# Expression profiling of lncRNAs and mRNAs reveals regulation of muscle growth in the Pacific abalone, *Haliotis discus hannai*

**DOI:** 10.1038/s41598-018-35202-z

**Published:** 2018-11-15

**Authors:** Jianfang Huang, Xuan Luo, Liting Zeng, Zekun Huang, Miaoqin Huang, Weiwei You, Caihuan Ke

**Affiliations:** 1 State Key Laboratory of Marine Environmental Science, Xiamen University, Xiamen, 361102 China; 2 College of Ocean and Earth Sciences, Xiamen University, Xiamen, 361102 China; 3 Fujian Collaborative Innovation Center for Exploitation and Utilization of Marine Biological Resources, Xiamen University, Xiamen, 361102 China

## Abstract

Long non-coding RNAs (lncRNAs) are known to play a major role in the epigenetic regulation of muscle development. Unfortunately there is little understanding of the mechanisms with which they regulate muscle growth in abalone. Therefore, we used RNA-seq to study the muscle transcriptomes of six *Haliotis discus hannai* specimens: three large (L_HD group) and three small (S_HD group). We identified 2463 lncRNAs in abalone muscle belonging to two subtypes: 160 anti-sense lncRNAs and 2303 intergenic lncRNAs (lincRNAs). In the L_HD group, we identified 204 significantly differentially expressed lncRNAs (55 upregulated and 149 downregulated), and 2268 significantly differentially expressed mRNAs (994 upregulated and 1274 downregulated), as compared to the S_HD group. The bioinformatics analysis indicated that lncRNAs were relate to cell growth, regulation of growth, MAPK signaling pathway, TGF-β signaling pathway, PI3K-Akt and insulin signaling pathway, which involved in regulating muscle growth. These findings contribute to understanding the possible regulatory mechanisms of muscle growth in Pacific abalone.

## Introduction

Muscle growth in livestock is very important, as it directly affects meat production. The regulatory mechanisms of muscle growth are complex, and are affected by genetics, nutrition, and the environment^1^. Of these, genetic factors, including those growth hormone (GH), insulin-like growth factors (IGFs), myogenic regulatory factors (MRFs), myostatin (Mstn), and paired box proteins (Paxs), are the most important^1,2^. However, studies of the role of non-coding RNA, particularly long noncoding RNA (lncRNA), in the regulation of muscle growth remain scarce.

LncRNAs are RNA molecules longer than 200 nucleotides (nt) that have little or no open reading frame (ORF)^3^. Compared with mRNAs, lncRNAs are marked by lower expression levels, less conservation, and more variable expression among tissues^4,5^. Many researches have shown that lncRNAs are relate to various biological processes including cancer, apoptosis, immunity, and development^6–8^. Several studies have also indicated that lncRNAs play a vital role in muscle growth^9,10^. For example, *Lnc133* was highly expressed in the adductor muscle of *Pinctada martensii* and it could be involved in regulating the cell proliferation of adductor muscles by targeting *pm*-RhoA^11^. Most currently identified lncRNAs have been derived from mice and humans^12–14^. Several studies in chickens^9^, cattle^15^, pigs^16^, zebrafish^17^, and rainbow trout^18^ have enriched the datasets of animal lncRNA, but little is known about lncRNA in the abalone.

The Pacific abalone, is the most commonly cultivated abalone in China^19^. Here, we used Illumina HiSeqX sequencing to determine the lncRNA and mRNA expression profiles of two *H*. *discus hannai* phenotypes that differ with respect to muscle growth rate. We then used quantitative real-time polymerase chain reactions (qRT-PCR) to compare the expression levels of muscle growth-related genes between these phenotypes. These results increase our knowledge of the molecular mechanisms regulating muscle growth in the abalone.

## Results

### Identification of candidate lncRNAs

**Figure 1:**
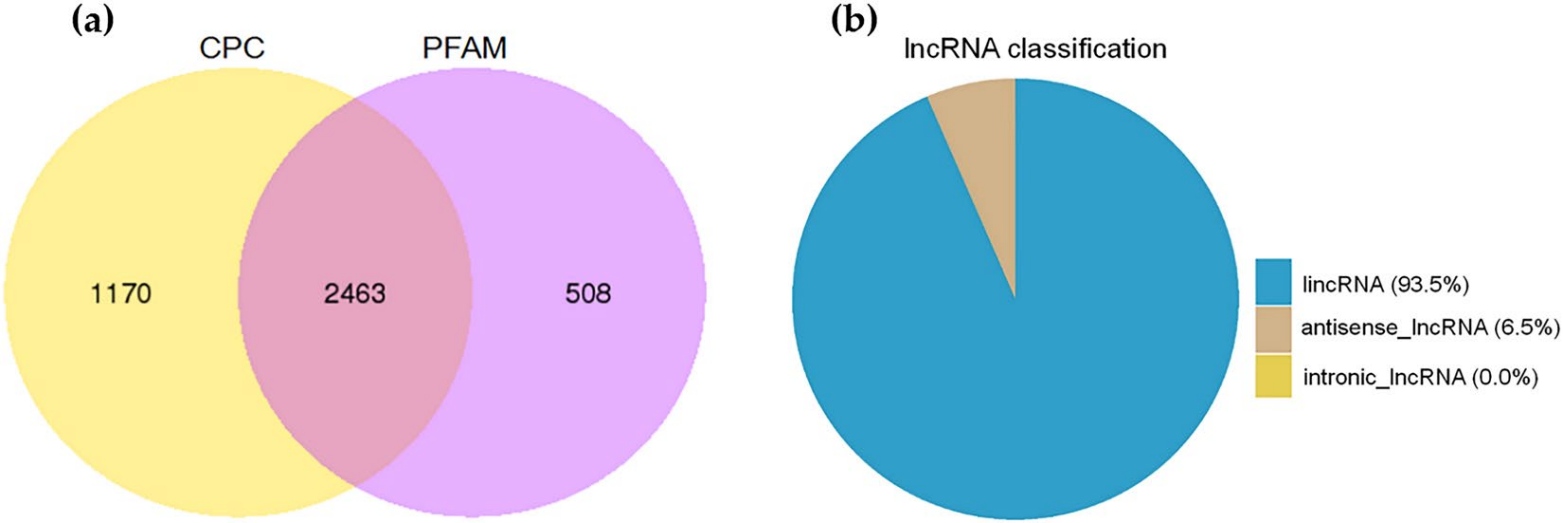
Screening and classification of predicted lncRNAs in the adductor muscle transcriptome. (**a**) The protein-coding potentials of lncRNAs were analyzed with CPC and PFAM. (**b**) The proportion of lncRNAs that were intergenic lncRNAs (lincRNAs), intronic lncRNAs, and anti-sense lncRNAs.

We generated 709,386,602 raw RNA-seq reads (NCBI accession no. SRP126378) from the adductor muscle samples of the three large (L_HD) and three small (S_HD) *H*. *discus hannai* specimens. The result of RNA quality was shown in Table 1. After discarding low-quality, adaptor, and poly-N sequences, 688,261,544 clean reads remained. We were able to map between 64.09% and 68.95% of the clean reads in each library to the *H*. *discus hannai* reference genome (Supplementary Table S1). Our coding potential analysis identified 2463 lncRNAs (Fig. 1): 2303 lincRNAs (93.5%) and 160 anti-sense lncRNAs (6.5%). We did not identify any intronic lncRNAs.

**Table 1:** The result of RNA quality.

Sample name	Raw reads	Clean reads	clean bases	Error rate(%)	Q20(%)	Q30(%)	GC content(%)
L_1	121394632	117028432	17.55G	0.02	95.31	88.73	47.42
L_2	109139940	105852548	15.88G	0.03	95.14	88.46	47.74
L_3	120542518	115621922	17.34G	0.02	95.29	88.75	48.83
S_1	129079606	126056030	18.91G	0.03	95.03	88.25	45.43
S_2	119736948	116934824	17.54G	0.02	95.17	88.49	45.93
S_3	109492958	106767788	16.02G	0.03	94.93	88.05	45.21

### Genomic characterization of the candidate lncRNAs

**Figure 2:**
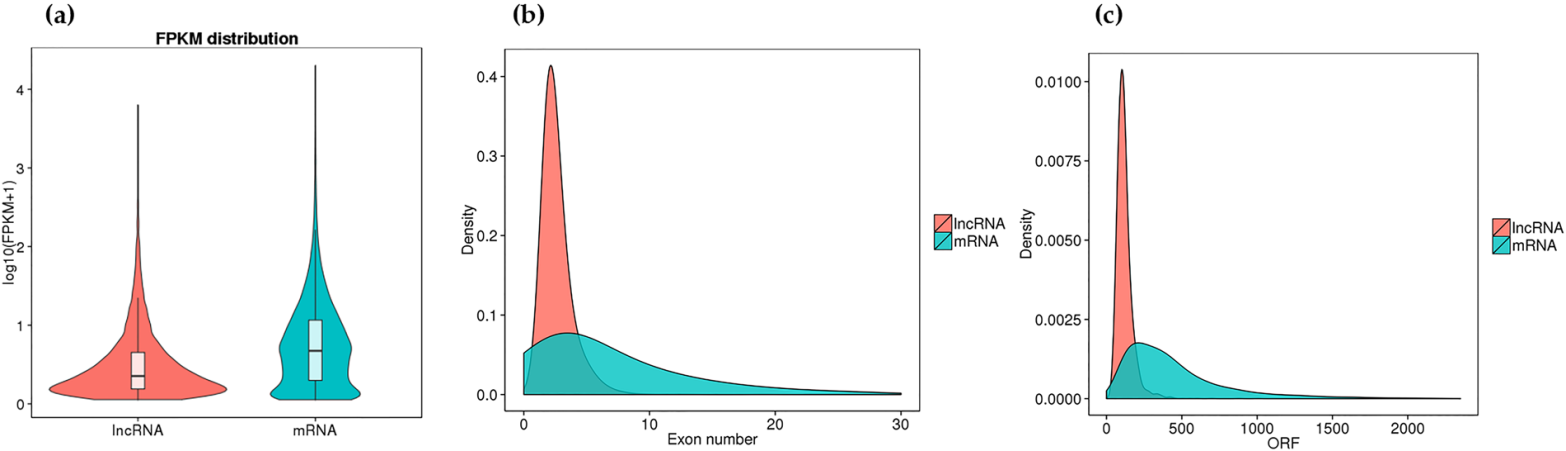
A comparison of candidate lncRNA and mRNA features. (**a**) Expression of lncRNAs and mRNAs. (**b**) Density distribution of the number of exons in lncRNAs and mRNAs. (**c**) Density distribution of the ORF length in lncRNAs and mRNAs.

We identified 23,847 mRNAs and 2463 lncRNAs in the adductor muscle samples from the six *H*. *discus hannai* specimens. We found that the lncRNAs were less expressed than the mRNAs (Fig. 2a), and the lncRNAs had fewer exons than the mRNAs (Fig. 2b). In addition, in comparison to the mRNAs, most lncRNAs were shorter ORF length (Fig. 2c).

### Differential expression (DE) cluster analysis

**Figure 3:**
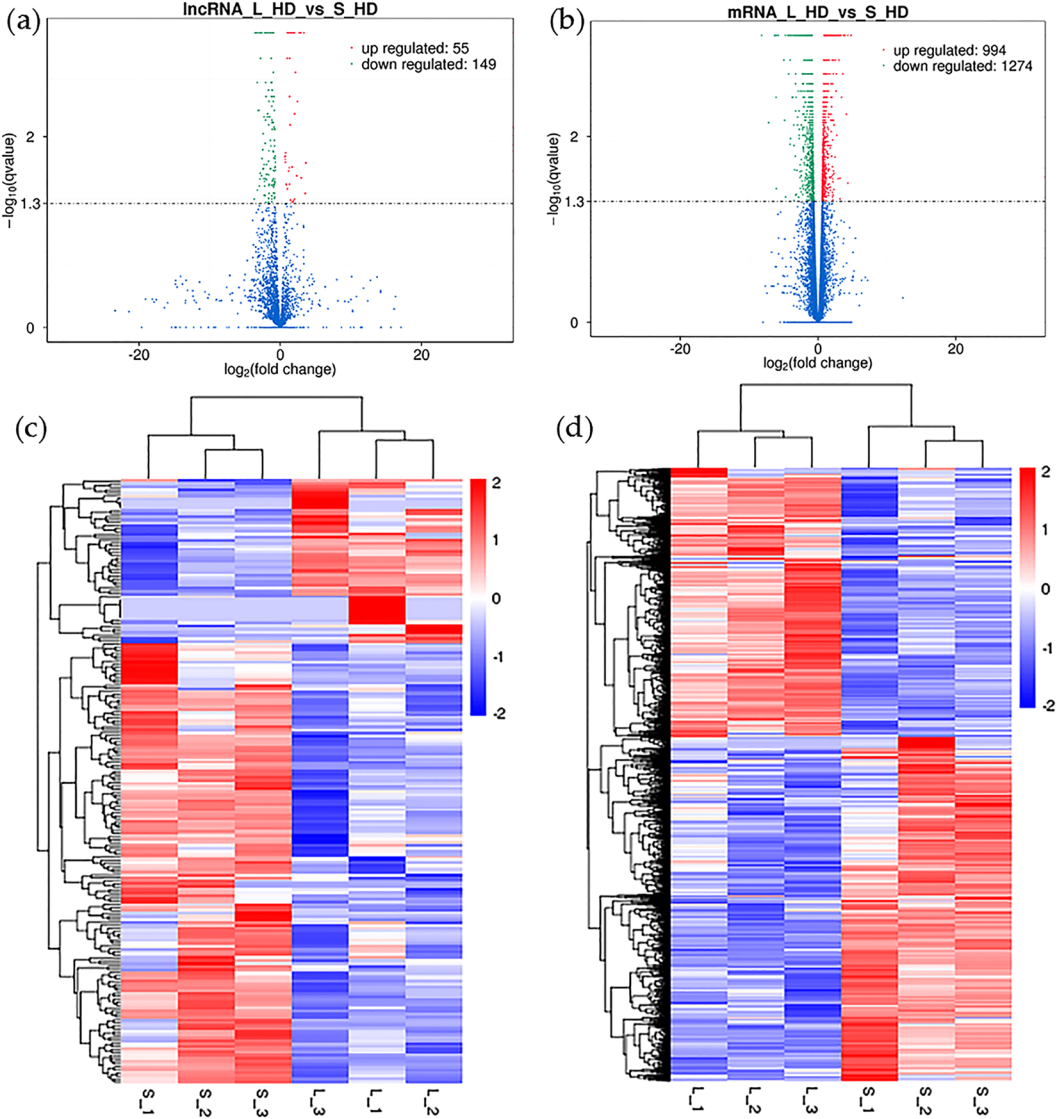
Volcano plots and heat maps of differentially expressed transcripts (*P* < 0.05). Expression of (**a**) lncRNAs and (**b**) mRNAs in large (L_HD) versus small (S_HD) specimens of abalone. Red and green dots indicate up- and down-regulated transcripts, respectively. Hierarchical clustering of differentially expressed (**c**) lncRNAs and (**d**) mRNAs. Red rectangles represent upregulated lncRNAs/mRNAs; blue rectangles represent downregulated lncRNAs/mRNAs.

We obtained 204 lncRNAs (DE-lncRNAs) and 2268 mRNAs (DE-mRNAs) that were significantly differentially expressed between the L_HD and S_HD specimens (*P* < 0.05; Supplementary Tables S2 and 3). In the L_HD specimens, 55 DE-lncRNAs and 994 DE-mRNAs were upregulated compared to the S_HD specimens, while 149 DE-lncRNAs and 1274 DE-mRNAs were downregulated (Fig. 3a,b). Our heat maps also suggested that lncRNAs (Fig. 3c) and mRNAs (Fig. 3d) were significant expression difference (*P* < 0.05) between the two groups.

### Prediction of the lncRNA target genes

LncRNAs can act in *cis* to regulate the neighboring genes; or they may function in *trans* to regulate the expression of genes located in distant domains^20^. To better understand the functional roles of our identified lncRNAs, we forecasted the targets of lncRNAs. We identified 1727 lncRNAs acting in *cis* with 5512 mRNAs. Interestingly, several muscle development-related genes including ras homolog family member A (RhoA) and cell division cycle 42 (Cdc42), were targeted by the lncRNAs XLOC_042193 and XLOC_020807, indicating that these muscle growth genes may be *cis*-regulated by lncRNAs. We identified 327,782 interactions in *trans* between 2464 lncRNAs and 16,676 mRNAs. Similarly, we observed that several DE-lncRNAs (such as XLOC_031278, XLOC_019246, XLOC_046403, XLOC_021050) acted in *trans* on muscle growth-related genes (Table 2).

**Table 2:** Long non-coding RNAs (lncRNAs) and lncRNA target genes that are associated with muscle growth.

Target genes	*Cis*-lncRNA	*Trans*-lncRNA
ras homolog family member A (RhoA)	XLOC_042193	XLOC_047918, XLOC_020199, XLOC_012389, XLOC_045008, XLOC_046195
multiple EGF like domains 10 (Megf10)	XLOC_001947	XLOC_007603, XLOC_009224, XLOC_009858, XLOC_008709, XLOC_036992, XLOC_050377, XLOC_012901, XLOC_041226, XLOC_022894, XLOC_002316
cell division cycle 42 (Cdc42)	XLOC_020807	XLOC_031278, XLOC_005639, XLOC_044588, XLOC_034979, XLOC_043665, XLOC_042193, XLOC_042273, XLOC_032617, XLOC_004306, XLOC_001947, XLOC_001947, XLOC_036419, XLOC_015393, XLOC_001333, XLOC_031494, XLOC_000853
growth differentiation factor 8 (Gdf8)		XLOC_019246, XLOC_047280
kruppel-like factor 5 (Klf5)		XLOC_016243, XLOC_019672, XLOC_046721, XLOC_044403, XLOC_020895, XLOC_030357, XLOC_041651, XLOC_032933, XLOC_045896
mothers against decapentaplegic homolog 3 (Smad3)		XLOC_008991, XLOC_007226, XLOC_002646, XLOC_046403, XLOC_014032, XLOC_019974, XLOC_045193, XLOC_036406
myocyte enhancer factor 2 A (Mef2A)		XLOC_046403, XLOC_032049, XLOC_002646, XLOC_021050, XLOC_014032
insulin like growth factor 2 receptor (Igf2R)		XLOC_044392, XLOC_018947, XLOC_026363, XLOC_019672, XLOC_028896, XLOC_047606, XLOC_046195, XLOC_017657, XLOC_042141, XLOC_020895, XLOC_030357, XLOC_009037, XLOC_043937, XLOC_047918, XLOC_041651, XLOC_032933, XLOC_045008
myosin heavy chain (Myh)		XLOC_021050, XLOC_039472, XLOC_020134, XLOC_013832, XLOC_042141, XLOC_002952, XLOC_027398, XLOC_047606, XLOC_002695, XLOC_036494, XLOC_043937, XLOC_011639, XLOC_015925, XLOC_036406, XLOC_025234, XLOC_026363, XLOC_033661, XLOC_032049, XLOC_028897, XLOC_046403, XLOC_044392
fibroblast growth factor receptor (Fgfr)		XLOC_050379, XLOC_050377, XLOC_003281, XLOC_001947, XLOC_047172, XLOC_000329
sirtuin 3 (Sirt3)		XLOC_014032, XLOC_019974, XLOC_021050, XLOC_039472, XLOC_029968, XLOC_020134, XLOC_013832, XLOC_034979, XLOC_045094, XLOC_045193, XLOC_043252, XLOC_002695, XLOC_001626, XLOC_043937, XLOC_011639, XLOC_015925, XLOC_016026, XLOC_000853, XLOC_036406, XLOC_011751, XLOC_017610, XLOC_033661, XLOC_002857, XLOC_007226, XLOC_002646, XLOC_046403, XLOC_005168, XLOC_044392

### Bioinformatics analysis

**Figure 4:**
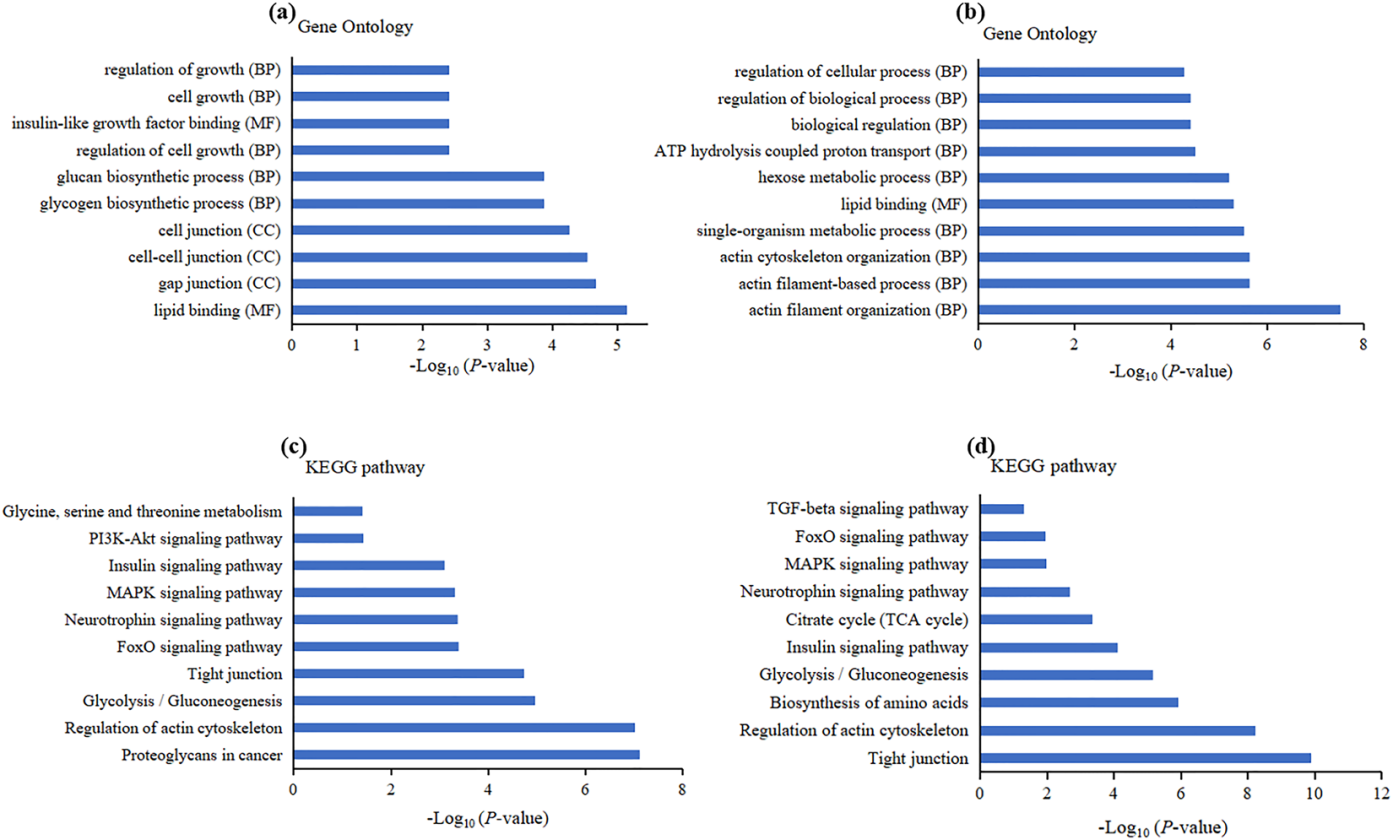
Analysis of significant GO terms and KEGG pathways for the predicted differentially expressed target mRNAs of our DE-lncRNAs. Significant GO terms for genes (**a**) *cis*-regulated and (**b**) *trans-*regulated by lncRNAs in L_HD specimens, as compared to S_HD specimens. BP: biological process; MF: molecular function; CC: cellular_component. Significant KEGG pathways for genes (**c**) *cis*-regulated and (**d**) *trans*-regulated by lncRNAs in L_HD specimens, as compared to S_HD specimens (*P* < 0.05 is recommended).

Our GO analysis of the DE-target mRNAs regulated in *cis* by DE-lncRNAs identified 120 significantly terms (*P* < 0.05). These terms were primarily involved in growth regulation and in biosynthetic-related functions such as glycogen biosynthetic process, regulation of cell growth, insulin-like growth factor binding, and regulation of growth (Fig. 4a). We identified 322 GO terms significantly enriched across the DE-target mRNAs regulated in *trans* by DE-lncRNAs (*P* < 0.05). These GO terms encompassed various biological processes, including actin cytoskeleton organization, hexose metabolic process, and regulation of biological process (Fig. 4b).

The DE-target mRNAs of the DE-lncRNAs regulated in *cis* were significantly enriched in 82 KEGG pathways. Some of these signaling pathways were concerned with muscle growth, including the MAPK, the FoxO, and the PI3K-Akt signaling pathway (Fig. 4c). Our results therefore indicated that lncRNAs may function in *cis* on neighboring genes to influence muscle development. Our functional analysis also indicated that DE-target mRNAs in *trans* were significantly enriched in 103 KEGG pathways. Several of these signaling pathways were associated with muscle growth, including the MAPK, the TGF-β, and the insulin signaling pathway (Fig. 4d).

### LncRNA-mRNA interaction network

**Figure 5:**
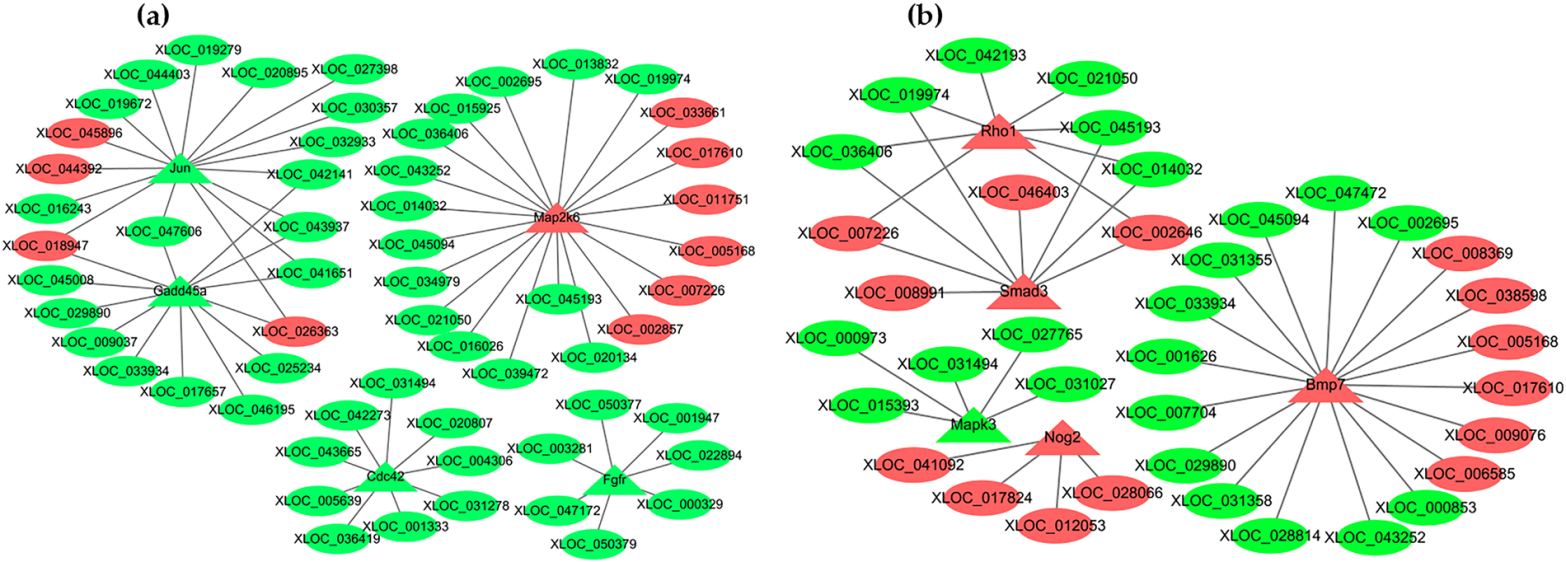
LncRNA-mRNA interaction networks. (**a**) The MAPK signaling pathway, showing 59 lncRNAs interacting with 5 mRNAs. (**b**) The TGF-β signaling pathway, showing 37 lncRNAs interacting with 5 mRNAs. All interactions show gene expression in large specimens, as compared to small specimens. Green ovals: downregulated lncRNAs; red ovals: upregulated lncRNAs; green triangles: downregulated genes; red triangles: upregulated genes.

Our lncRNA-mRNA interaction network results indicated that possible regulatory network interactions were linked to several signaling pathways, including the MAPK, the FoxO, the PI3K-Akt, and the TGF-β signaling pathway. Here, several DE-mRNAs and their corresponding DE-lncRNA regulators were constructed to assess their function with respect to abalone muscle growth (Fig. 5). We found that 59 lncRNAs interacted with five mRNAs in the MAPK signaling pathway (Fig. 5a), while 37 lncRNAs interacted with five mRNAs in the TGF-β signaling pathway (Fig. 5b).

### Specific expression of lncRNAs

We found 14 specific lncRNA expressions in the L_HD, particularly XLOC_007603, which has the lowest P value. Genes multiple EGF like domains 10 (Megf10) and bone morphogenetic protein 7 (Bmp7) were targeted by XLOC_007603. We also discovered nine specific lncRNA expressions in the S_HD samples, such as XLOC_004306. Growth hormone secretagogue receptor type 1 (Ghsr) and Actin, both related to growth, were targeted by XLOC_004306. These specific expressed lncRNAs perhaps play crucial roles in abalone muscle growth, although the underlying regulatory mechanisms require further study.

### Validation of the transcripts expression by qRT-PCR

**Figure 6:**
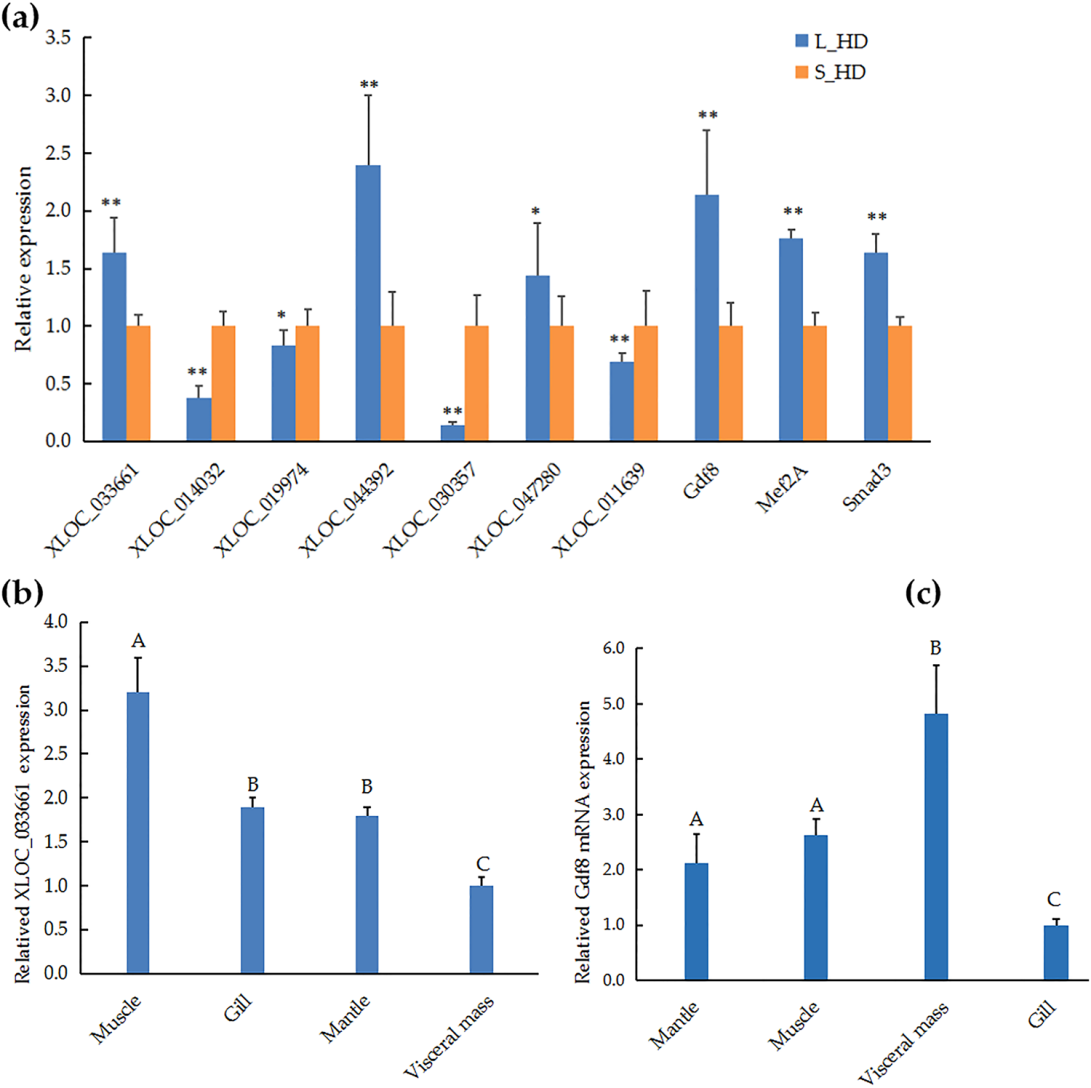
Relative expression of lncRNAs and mRNAs, quantified with qRT-PCR. (**a**) Some lncRNAs and mRNAs were tested in the muscle of *Haliotis discus hannai*. (**b**) Expression of XLOC_033661 in the mantle, muscle, visceral mass, and gill. (**c**) Expression of Gdf8 in the mantle, muscle, visceral mass, and gill. Asterisks indicate statistically significant differences between large (L_HD) and small (S_HD) specimens: *P < 0.05; **P < 0.01. Different capital letters indicate significant differences among tissues (*P* < 0.01).

To validate our sequencing results, we selected three upregulated DE-mRNAs, three upregulated DE-lncRNAs, and four downregulated DE-lncRNAs to analyse the expression levels using qRT-PCR. (Fig. 6a). The expression patterns of these DE-lncRNAs and DE-mRNAs were accordance with the sequencing data, suggesting that our RNA-seq data were accurate. Our analysis of the tissue expression patterns of XLOC_033661 and growth differentiation factor 8 (Gdf8) suggested that these were ubiquitously expressed in all examined tissues (Fig. 6b,c).

## Discussion

Muscle growth is a complex life activity regulated by the coordinated action of many biological processes. Abalone with different body weights have different growth rates: larger abalones grow faster and smaller abalones grow slower^21,22^. To clarify the mechanisms underlying muscle growth in Pacific abalone, we used RNA-seq to investigate the discrepancy in mRNA and lncRNA expression patterns between larger and smaller abalone specimens from the same family.

As far as we know, this is the first study of lncRNA expression data in *H*. *discus hannai*. Here, we identified 2463 lncRNAs and 23,847 mRNAs. We found that the lncRNAs had fewer exons and were shorter than the mRNAs, consistent with previous studies^18,23,24^. The average number of exons (mean: 2.6) found in the lncRNAs of *H*. *discus hannai* was less than that of zebrafish (mean: 2.8 exons), humans (mean: 2.9 exons), and mice (mean: 3.7 exons)^17,24^. LncRNAs were also less expressed than the mRNAs, again consistent with previous studies.

LncRNAs act as either *cis-* or *trans-*regulatory elements, with either co-localized or co-expressed protein-coding genes as targets^10^. For example, Linc-MD1, influences muscle development by targeting MAML1^25^. Here, we identified 204 DE-lncRNAs and 2268 DE-mRNAs between the L_HD group and the S_HD group. We also constructed interaction networks between the *cis*- and *trans*-acting DE-lncRNAs and their mRNA targets to estimate the function of DE-lncRNAs in the regulation of muscle growth. Some genes have been shown to be connection with muscle growth, including Gdf8^26,27^, kruppel-like factor 5 (Klf5)^28^, tuberous sclerosis-1 (Tsc1)^29^, sirtuin 3 (Sirt3)^30^, myocyte enhancer factor 2 A (Mef2A)^31^, insulin like growth factor 2 receptor (Igf2R)^32^, RhoA^33^, Cdc42^34^, Megf10^35^, and myosin heavy chain (Myh)^36^. Gdf8 (also known as Mtsn) is an important member of the TGF-β superfamily, and functions as a negative regulator of skeletal muscle development and growth^37^. Our expression analyses suggested that Gdf8 was ubiquitously expressed in all tested tissues, consistent with previous studies^27^. We found that Gdf8 mRNA was most highly expressed in the muscle and visceral mass, indicating that Gdf8 may play important roles in these tissues. We noticed the highest levels of XLOC_033661 expression in the muscle, indicating that this lncRNA perhaps play a vital role in muscle growth. Mef2A is known to be highly expressed in skeletal muscle, suggesting that it is valuable for skeletal muscle myoblast differentiation^38^. LncRNA-uc.167 is antisense to the Mef2C gene, and influences P19 cell proliferation and differentiation by regulating Mef2C^39^. Therefore, we speculate that the lncRNAs XLOC_046403, XLOC_032049, XLOC_002646, XLOC_021050, and XLOC_014032 regulate the muscle growth in *H*. *discus hannai* by targeting Mef2A. Similarly, other lncRNAs might affect muscle growth by targeting specific genes.

The results of GO and KEGG pathway analyses could help us understand the mechanisms underlying abalone muscle growth. Moreover, our lncRNA-mRNA interaction network indicated that 59 lncRNAs interacted with 5 mRNAs in the MAPK signaling pathway, and 37 lncRNAs interacted with 5 mRNAs in the TGF-β signaling pathway (Fig. 5a,b). Association of DE-mRNAs and DE-lncRNAs with pathways relevant to growth may partly explain the regulation of muscle development. The MAPK signaling pathway, which includes the p38 MAPK, the extracellular regulated kinase 1 and 2 (ERK1/2), and the Jun NH2-terminal kinase (JNK) pathways, plays a vital role in muscle development^40,41^. The p38 MAPK though regulating the sequential activation of MRFs and their transcriptional coactivators to control skeletal muscle differentiation^42^. Mothers against decapentaplegic homolog 3 (Smad3) acts downstream of TGF-β to repress the bHLH domain of MyoD, and thus control myoblast differentiation^43^. TGF-β/Smad3 stimulated smooth muscle cell (SMC) proliferation is controlled by the PI3K/Akt signaling pathway^44^. PI3K/Akt is one of the major pathways contributing to skeletal muscle differentiation^45^. Our results can elucidate key lncRNAs and provide leads to further understand the mechanisms of molluscan muscle growth.

In conclusion, we reported the first lncRNA expression profiles of *H*. *discus hannai* using Illumina HiSeqX sequencing technology and identified 2463 lncRNAs. We also found out DE-mRNAs and DE-lncRNAs in slow- and fast- growing specimens of *H*. *discus hannai*. We identified lncRNAs acting in *cis* and *trans* to target genes (mRNAs). Our bioinformatics analyses suggested that many DE-lncRNAs might influence the regulation of muscle growth in *H*. *discus hannai* by affecting target genes. All these findings may help to understand the biological mechanisms controlling muscle growth in the abalone. Nevertheless, the roles of lncRNAs and their target genes analyses need further experiental verification.

## Materials and Methods

### Experimental sample

A breeding population of *H*. *discus hannai* has produced pedigreed offspring; The six *H*. *discus hannai* abalones used in this research were obtained from Fuda Aquiculture in Jinjiang, Fujian province, China; all specimens were about 2 years old. Three of the samples were larger (“L_HD” group; mean weight, 95.1 ± 7.7 g; mean muscle weight, 45.5 ± 5.0 g), and three were smaller (“S_HD” group; mean weight, 16.5 ± 1.0 g; mean muscle weight, 7.3 ± 0.8 g). All six specimens of the adductor muscle, mantle, visceral mass, and gill were collected from each abalone, immediately snap-frozen in liquid nitrogen^46^.

The corresponding author declares that all the methods were approved and perform in agreement with the instructions of the Laboratory Animal Management and Ethics Committee of Xiamen University and that all experimental protocols about abalones were carried out in accordance with the Regulations for the Administration of Affairs Concerning Experimental Animals of Xiamen University. Moreover, all the researcher who perform the animal experiments are trained by attending specifc courses.

### RNA isolation and Illumina deep sequencing

The total RNA was isolated from adductor muscle samples taken from each *H*. *discus hannai* specimen using TRIzol reagent (Invitrogen, Carlsbad, CA, USA). Then, we checked the purity of the total RNA and assessed its integrity. Approximately 3 µg RNA per sample was used to construct a complementary (cDNA) library. We used a TruSeq PE Cluster Kit v3-cBot-HS with the cBot Cluster Generation System (Illumina, San Diego, CA, USA) to cluster the index-coded sample. The libraries were sequenced on an Illumina HiseqX platform and 150 bp paired-end reads were generated. Raw data were cleaned with in-house Perl scripts. Specifically, our script removed low quality reads, those containing adapter sequences, and those containing poly-N sequences to generate clean reads. At the same time, our script also calculated the Q20, Q30, and GC content of the clean data.

### Transcriptome assembly

We used previously generated reference genome and gene model annotation files for *H*. *discus hannai* (the files provided by Dr. Weiwei You, Xiamen University, Xiamen). The clean reads were mapped to the *H*. *discus hannai* reference genome using TopHat v2.0.9^47^ with default parameters. The mapped reads were assembled with both Scripture (beta2)^22^ and Cufflinks v2.1.1^48,49^.

### Quantification of gene expression level

We calculated the fragments per kilobase (kb) per million reads (FPKMs) for both the lncRNAs and the coding genes using Cuffdiff v2.1.1^50^. We considered transcripts or genes differentially expressed when expression levels were significantly different (adjusted *P* of <0.05) between the large and small specimens (L_HD and S_HD).

### Identification of lncRNAs

We used CPC (0.9-r2)^51^ and Pfam-scan (v1.3)^52^ to screen for candidate lncRNAs. Only those transcripts without predicted coding potential were retained. Finally, we selected the candidate lncRNAs predicted by both CPC and Pfam-scan as final lncRNAs for further analyses.

To investigate transcript conservation, we computed phylogenetic models in the Phast (v1.3) package^53^. Then, we computed the conservation scores of lncRNAs and coding genes using phastCons.

### Target gene prediction

LncRNAs acting in *cis* act on neighboring target genes^54,55^. To identify these, we searched mRNAs 10 k/100 k up- and down-stream of each lncRNA. LncRNAs acting in *trans* influence target genes at the expression level. We computed the Pearson’s correlation coefficients both the expression levels of mRNAs and lncRNAs with custom scripts (*r* > 0.95 or *r* < −0.95). The lncRNA-mRNA interaction networks of DE-lncRNAs and their corresponding DE-mRNAs were constructed using Cytoscape.

### Functional enrichment analysis

To evaluated the functions of the DE-lncRNA, we analyzed GO (Gene Ontology) with the GOseq R package^56^. We also performed KEGG (http://www.genome.jp/kegg/) analysis on DE-target mRNAs of the DE-lncRNAs using the hypergeometric test in KOBAS^57^. We considered functions with *P* < 0.05 significantly enriched.

### QRT-PCR

Several genes were chosen for qRT-PCR using gene-specific primers (Supplementary Table S4). Relative gene expression levels were quantified based on β-actin gene expression using the 2^−∆∆CT^ method^58^.

### Statistical analysis

All qRT-PCR data were presented as mean ± standard deviation (SD). The statistical significance was evaluated using SPSS 19.0.

## Electronic supplementary material

Expression profiling of lncRNAs and mRNAs reveals regulation of muscle growth in the Pacific abalone, Haliotis discus hannai Supplementary Information
