# Supplementary material for: Expression profiling of lncRNAs and mRNAs reveals regulation of muscle growth in the Pacific abalone, Haliotis discus hannai
Source: Sci Rep. 2018 Nov 15;8:16839. doi: 10.1038/s41598-018-35202-z (PMC6237873; doi:10.1038/s41598-018-35202-z)
Supplement: Supplementary file 1 — Expression profiling of lncRNAs and mRNAs reveals regulation of muscle growth in the Pacific abalone, Haliotis discus hannai Supplementary Information [file 41598_2018_35202_MOESM1_ESM.pdf]

Expression profiling of lncRNAs and mRNAs reveals regulation of muscle growth in the Pacific abalone, Haliotis discus hannai

Jianfang Huang, Xuan Luo, Liting Zeng, Zekun Huang, Miaoqin Huang, Weiwei You, and Caihuan Ke

This document contains Supplementary Tables S1-S4.

Table S1 The results of reads mappable to *H. discus hannai* reference genome

Sample name	L_1	L_2	L_3	S_1	S_2	S_3
Raw reads	121394632	109139940	120542518	129079606	119736948	109492958
Total reads	117028432	105852548	115621922	126056030	116934824	106767788
Total mapped	77448108 (66.18%)	69969625 (66.1%)	79723225 (68.95%)	80793369 (64.09%)	74985875 (64.13%)	69778281 (65.36%)
Multiple mapped	2137191 (1.83%)	1924097 (1.82%)	2770803 (2.4%)	3033292 (2.41%)	2307796 (1.97%)	2262043 (2.12%)
Uniquely mapped	75310917 (64.35%)	68045528 (64.28%)	76952422 (66.56%)	77760077 (61.69%)	72678079 (62.15%)	67516238 (63.24%)
Read-1	39266099 (33.55%)	35594382 (33.63%)	40393617 (34.94%)	40722717 (32.31%)	37738552 (32.27%)	35752324 (33.49%)
Read-2	36044818 (30.8%)	32451146 (30.66%)	36558805 (31.62%)	37037360 (29.38%)	34939527 (29.88%)	31763914 (29.75%)
Reads map to '+'	37779844 (32.28%)	34319847 (32.42%)	39019465 (33.75%)	38935554 (30.89%)	36114078 (30.88%)	34001514 (31.85%)
Reads map to '-'	37531073 (32.07%)	33725681 (31.86%)	37932957 (32.81%)	38824523 (30.8%)	36564001 (31.27%)	33514724 (31.39%)
Non-splice reads	48831577 (41.73%)	43520789 (41.11%)	44205571 (38.23%)	54488540 (43.23%)	49132264 (42.02%)	46864939 (43.89%)
Splice reads	26479340 (22.63%)	24524739 (23.17%)	32746851 (28.32%)	23271537 (18.46%)	23545815 (20.14%)	20651299 (19.34%)
Reads mapped in proper pairs	63391680 (54.17%)	56094084 (52.99%)	64416528 (55.71%)	63452244 (50.34%)	59567226 (50.94%)	54062922 (50.64%)

Table S2 Differentially expressed lncRNAs

gene_id	L_HD_FPKM	S_HD_FPKM	log2(foldchange)	*P*
XLOC_016243	0	0.657163	#NAME?	0.000820679
XLOC_025907	0	0.696107	#NAME?	0.000820679
XLOC_001567	0	129.006	#NAME?	0.000820679
XLOC_001376	0	1.45772	#NAME?	0.000820679
XLOC_031574	0	6.75832	#NAME?	0.000820679
XLOC_002412	0.871669	0	inf	0.000820679
XLOC_034008	7.40667	0	inf	0.000820679
XLOC_008213	0.958475	0	inf	0.000820679
XLOC_008991	1.47691	0	inf	0.000820679
XLOC_014829	7.54999	0	inf	0.000820679
XLOC_007603	48.6252	0	inf	0.000820679
XLOC_000952	38.8146	0	inf	0.000820679
XLOC_001841	7.40497	0	inf	0.000820679
XLOC_036689	0.599902	0	inf	0.000820679
XLOC_011751	224.856	21.849	3.36337	0.000820679
XLOC_005168	291.909	38.0501	2.93955	0.000820679
XLOC_012053	11435.6	1578.15	2.85722	0.000820679
XLOC_050345	7.87554	1.21692	2.69415	0.000820679
XLOC_044392	1042.34	262.475	1.98958	0.000820679
XLOC_007849	35.7181	10.0772	1.82556	0.000820679
XLOC_033661	541.505	175.435	1.62604	0.000820679
XLOC_018947	18.3638	6.89696	1.41283	0.000820679

XLOC_002857	135.138	52.3174	1.36907	0.000820679
XLOC_041819	2216.83	891.212	1.31466	0.000820679
XLOC_047280	50.8772	23.7196	1.10094	0.000820679
XLOC_032049	267.273	125.075	1.09552	0.000820679
XLOC_026363	11.4004	5.41364	1.07441	0.000820679
XLOC_017824	30.9543	15.5082	0.997113	0.000820679
XLOC_018930	3.63246	6.92411	-0.930681	0.000820679
XLOC_047606	5.97316	11.8287	-0.985721	0.000820679
XLOC_002952	150.148	301.377	-1.00518	0.000820679
XLOC_001441	78.6005	159.809	-1.02374	0.000820679
XLOC_008709	3.21785	6.60349	-1.03713	0.000820679
XLOC_034832	5.97318	12.8832	-1.10892	0.000820679
XLOC_029968	9.53356	21.1471	-1.14937	0.000820679
XLOC_034979	1.00255	2.25185	-1.16744	0.000820679
XLOC_014032	1.39471	3.13873	-1.17022	0.000820679
XLOC_036494	53.0362	125.19	-1.23907	0.000820679
XLOC_009858	3.32723	7.86259	-1.24068	0.000820679
XLOC_017657	8.6908	21.3553	-1.29703	0.000820679
XLOC_047299	3.48013	8.57363	-1.30077	0.000820679
XLOC_000329	13.1401	33.4446	-1.34779	0.000820679
XLOC_045094	1.66774	4.2684	-1.3558	0.000820679
XLOC_036992	15.7845	42.7901	-1.43876	0.000820679
XLOC_027758	1.38463	3.79536	-1.45474	0.000820679
XLOC_050377	6.51997	17.9304	-1.45947	0.000820679

XLOC_042141	58.6949	165.248	-1.49332	0.000820679
XLOC_027398	322.813	1064.39	-1.72126	0.000820679
XLOC_028929	26.3712	94.3394	-1.83889	0.000820679
XLOC_050379	6.83102	24.9653	-1.86975	0.000820679
XLOC_018938	2.3827	10.0168	-2.07175	0.000820679
XLOC_048553	35.4306	158.513	-2.16154	0.000820679
XLOC_002170	1.17497	5.37483	-2.1936	0.000820679
XLOC_041651	0.457595	2.73226	-2.57795	0.000820679
XLOC_021172	0.273007	1.68703	-2.62748	0.000820679
XLOC_021161	0.579934	3.85776	-2.7338	0.000820679
XLOC_003281	0.743264	5.04382	-2.76257	0.000820679
XLOC_021193	0.181397	1.40645	-2.95483	0.000820679
XLOC_005639	2.62719	24.1699	-3.20162	0.000820679
XLOC_032933	0.433701	4.38035	-3.33627	0.000820679
XLOC_007704	2.29517	25.5437	-3.4763	0.000820679
XLOC_045008	0.353799	4.22862	-3.57918	0.000820679
XLOC_004306	0	64.5734	#NAME?	0.00151774
XLOC_008905	51.7294	20.523	1.33375	0.00151774
XLOC_028897	2.71595	1.37109	0.986134	0.00151774
XLOC_047300	1.95343	4.69239	-1.26432	0.00151774
XLOC_020807	3.20358	7.75528	-1.27549	0.00151774
XLOC_021194	0.189693	1.30994	-2.78776	0.00151774
XLOC_014459	0	3.011	#NAME?	0.00213042
XLOC_017610	17.1453	3.82447	2.16448	0.00213042

XLOC_016026	155.38	304.144	-0.968954	0.00213042
XLOC_036548	0	14.138	#NAME?	0.00271512
XLOC_029392	1.43163	3.25967	-1.18707	0.00271512
XLOC_009224	0.814252	1.94523	-1.25639	0.00271512
XLOC_001947	6.29393	15.8345	-1.33104	0.00271512
XLOC_043937	0.324935	1.35814	-2.0634	0.00271512
XLOC_020199	0.101785	0.979748	-3.26688	0.00271512
XLOC_011639	295.082	646.413	-1.13134	0.00326205
XLOC_031355	1.18766	3.73878	-1.65445	0.00326205
XLOC_046403	12.3552	4.95215	1.31899	0.00380231
XLOC_000853	7.07238	13.3077	-0.911997	0.00380231
XLOC_044403	1.90827	4.46045	-1.22492	0.00380231
XLOC_044588	0.450348	1.79934	-1.99836	0.00380231
XLOC_000082	36.0869	6.65685	2.43857	0.00429787
XLOC_036406	1.0774	2.11564	-0.973542	0.00429787
XLOC_018686	2.01843	6.0767	-1.59005	0.00429787
XLOC_001947	7.71142	17.699	-1.1986	0.00480132
XLOC_013832	5.33036	9.52934	-0.838144	0.00532397
XLOC_020203	0.119813	0.959781	-3.00192	0.00532397
XLOC_001517	0.317001	2.84835	-3.16757	0.00532397
XLOC_033970	3.17828	0.783521	2.0202	0.00578348
XLOC_000869	2.03867	6.98795	-1.77724	0.00578348
XLOC_001791	1.69958	4.81192	-1.50143	0.00621905
XLOC_041226	0.351769	1.02916	-1.54876	0.00621905

XLOC_033934	0.359348	1.2787	-1.83123	0.00621905
XLOC_000973	7.8421	38.2318	-2.28546	0.00621905
XLOC_013102	1.68967	3.78905	-1.16509	0.00670086
XLOC_021288	0.345789	1.45357	-2.07163	0.00670086
XLOC_015925	2.4851	4.82367	-0.956828	0.00712329
XLOC_016318	3.35944	7.33906	-1.12737	0.00712329
XLOC_046195	0.871075	1.91523	-1.13665	0.00712329
XLOC_043252	0.665165	1.49122	-1.16471	0.00712329
XLOC_008369	50.5748	19.1466	1.40133	0.00755259
XLOC_036596	1.32908	4.15976	-1.64607	0.00755259
XLOC_038260	1.38397	0	inf	0.00800162
XLOC_009538	7.50073	13.8976	-0.889734	0.00800162
XLOC_019672	5.05974	8.41833	-0.734471	0.00842602
XLOC_002037	126.541	223.88	-0.823123	0.00842602
XLOC_027765	1.24588	2.70665	-1.11935	0.00842602
XLOC_012901	0.552461	2.96683	-2.42498	0.00933293
XLOC_008035	1.0767	0	inf	0.00972356
XLOC_022894	3.40777	6.06728	-0.832224	0.0109379
XLOC_032976	0.263908	0.690338	-1.38727	0.0109379
XLOC_044798	0.307124	1.33866	-2.1239	0.0117709
XLOC_035802	0.65912	0	inf	0.0121758
XLOC_043240	23.0298	39.9995	-0.79648	0.0125549
XLOC_019279	0.533376	1.33999	-1.329	0.0125549
XLOC_047918	0.133219	0.739257	-2.47227	0.0137473

XLOC_000063	0.643003	0	inf	0.0141107
XLOC_047472	0.531535	1.07828	-1.0205	0.0141107
XLOC_028814	3.00821	9.29357	-1.62733	0.0141107
XLOC_042622	2.67069	5.23258	-0.970306	0.014476
XLOC_032894	89.6951	53.0973	0.756392	0.0148208
XLOC_002646	39.1228	23.9214	0.70971	0.0159979
XLOC_020134	5.64747	9.33216	-0.724606	0.0159979
XLOC_045896	86.9366	54.5389	0.672678	0.0163478
XLOC_015193	1.67145	3.13605	-0.90785	0.016685
XLOC_011594	125.071	206.86	-0.725915	0.0169923
XLOC_012389	4.50987	26.9002	-2.57646	0.0169923
XLOC_041092	21.3438	12.9862	0.716834	0.0173453
XLOC_031358	0.528898	1.66924	-1.65813	0.0180929
XLOC_001719	243.75	146.053	0.738904	0.0184892
XLOC_019974	7.1461	11.4578	-0.681097	0.0184892
XLOC_038597	11.1718	0.909558	3.61855	0.0188471
XLOC_042273	1.16142	4.49379	-1.95204	0.0195535
XLOC_019912	0.739215	3.59359	-2.28136	0.0195535
XLOC_031027	20.3801	33.6185	-0.722096	0.0205228
XLOC_028066	16.1768	5.04452	1.68114	0.0208422
XLOC_016562	23.9695	9.82056	1.28732	0.0211683
XLOC_026032	6.31219	10.6582	-0.755749	0.0211683
XLOC_002316	4.32165	11.404	-1.39988	0.0215514
XLOC_021186	0.343306	1.78617	-2.3793	0.0215514

XLOC_031278	0.854854	2.18786	-1.35577	0.0218619
XLOC_001626	0.193708	1.03216	-2.41372	0.0218619
XLOC_025809	18.1385	28.5837	-0.656133	0.0225564
XLOC_007938	17.9508	35.3726	-0.978588	0.0225564
XLOC_014343	0.810649	0	inf	0.0228478
XLOC_003002	5.59909	2.44744	1.19392	0.0228478
XLOC_002695	1.79023	3.25087	-0.860684	0.0228478
XLOC_021184	0.191964	1.36188	-2.82669	0.0241881
XLOC_033807	0.205882	0.743725	-1.85296	0.0248731
XLOC_009037	34.7192	136.241	-1.97236	0.0248731
XLOC_034923	1.45004	4.46774	-1.62345	0.0251677
XLOC_048293	2.61451	0.51673	2.33906	0.0257834
XLOC_015393	0.678134	4.2081	-2.63353	0.0257834
XLOC_047569	28.2951	3.63053	2.9623	0.0271297
XLOC_002982	15.8072	31.9398	-1.01478	0.0276479
XLOC_007414	0.220133	0.94983	-2.1093	0.0276479
XLOC_036419	0.193626	0.970086	-2.32484	0.0276479
XLOC_042193	2.5314	4.27407	-0.755679	0.027885
XLOC_043665	0.166797	1.228	-2.88014	0.027885
XLOC_030357	0.650986	2.49694	-1.93946	0.0282044
XLOC_025234	0.348573	0.732869	-1.0721	0.0287519
XLOC_007421	2.52968	4.40077	-0.7988	0.029403
XLOC_036426	0.679907	1.46289	-1.10541	0.029403
XLOC_046111	0.145123	0.680076	-2.22842	0.029403

XLOC_009517	0	7.08757	#NAME?	0.0299214
XLOC_032617	1.04959	1.74271	-0.731504	0.0302156
XLOC_009121	6.00169	10.415	-0.795227	0.0311397
XLOC_006585	3.78775	1.52785	1.30984	0.0316212
XLOC_003152	14.2168	7.92767	0.842626	0.0316212
XLOC_039472	1.25896	2.38824	-0.923722	0.0316212
XLOC_008443	1.25429	3.70462	-1.56246	0.0316212
XLOC_034294	1.9484	1.06224	0.875176	0.0318917
XLOC_008788	1.2211	2.87967	-1.23772	0.032187
XLOC_045193	3.96366	7.15594	-0.85231	0.0324675
XLOC_021050	0.548459	1.17934	-1.10452	0.0324675
XLOC_001638	17.9829	40.7506	-1.1802	0.0324675
XLOC_031494	4.85346	14.5758	-1.58649	0.0327195
XLOC_002650	0.16913	0.769902	-2.18655	0.0329966
XLOC_007730	0.195957	1.51133	-2.94721	0.0329966
XLOC_040061	1.11646	2.14296	-0.940674	0.0339147
XLOC_025525	0.824712	0.409934	1.0085	0.0353697
XLOC_047172	0.111035	1.05041	-3.24186	0.0364617
XLOC_028896	2.13328	3.68825	-0.789864	0.0391497
XLOC_041480	1.684	3.29415	-0.968014	0.0391497
XLOC_019246	3.37664	0.292824	3.52748	0.039382
XLOC_030660	9.77073	29.0212	-1.57057	0.039382
XLOC_007267	1.96062	8.67164	-2.14499	0.0413639
XLOC_029890	0.322714	1.58186	-2.29329	0.0413639

XLOC_008928	2.49256	5.66425	-1.18426	0.0418385
XLOC_025976	1.36891	2.32754	-0.765771	0.0427039
XLOC_048597	0.368061	1.14947	-1.64296	0.0441781
XLOC_008026	12.1211	21.4857	-0.825861	0.0444534
XLOC_020895	1.24521	3.88568	-1.64177	0.044934
XLOC_006129	15.1867	3.78536	2.0043	0.0451727
XLOC_000835	0.682768	1.55468	-1.18715	0.0451727
XLOC_019629	0.035824	0.458044	-3.67649	0.0451727
XLOC_009076	1.48636	0.545406	1.44638	0.0463231
XLOC_038598	4.17457	1.1074	1.91445	0.0474104
XLOC_046721	0.755152	1.73043	-1.19629	0.0482225
XLOC_007226	1.46197	0.445808	1.71342	0.0484321
XLOC_001333	0.173921	0.551549	-1.66506	0.0484321
XLOC_009408	0.781021	3.2165	-2.04206	0.048902

Table S3 Differentially expressed mRNAs

transcript_id	L_HD_FPKM	S_HD_FPKM	log2(foldchange)	*P*	swissprot
evm.model.scaffold7467.14	4.11271	8.40597	-1.03133	0.000820679	sp|Q9Z0R9|FADS2_MOUSE Fatty acid desaturase 2 OS=Mus musculus GN=Fads2 PE=2 SV=1
evm.model.scaffold43895.5	1.89064	4.43068	-1.22865	0.00621905	sp|Q90WY4|ADA2A_DANRE Alpha-2A adrenergic receptor OS=Danio rerio GN=adra2a PE=3 SV=1
evm.model.scaffold85903.7	1.40858	4.125	-1.55016	0.0152063	--
evm.model.scaffold93059.1	1.02822	2.3523	-1.19392	0.00271512	sp|Q01484|ANK2_HUMAN Ankyrin-2 OS=Homo sapiens GN=ANK2 PE=1 SV=4
evm.model.scaffold197.42	13.9004	28.7789	-1.04988	0.0184892	sp|Q8R3S2|TSN33_MOUSE Tetraspanin-33 OS=Mus musculus GN=Tspan33 PE=1 SV=1
evm.model.scaffold166635.22	0.117766	1.04427	-3.14849	0.0418385	sp|A9JRD8|BTB6A_DANRE BTB/POZ domain-containing protein 6-A OS=Danio rerio GN=btbd6a PE=1 SV=1
evm.model.scaffold166635.21	10.5418	25.2781	-1.26177	0.000820679	--
evm.model.scaffold43539.3	0.313324	91.8687	-8.19577	0.000820679	sp|O75581|LRP6_HUMAN Low-density lipoprotein receptor-related protein 6 OS=Homo sapiens GN=LRP6 PE=1 SV=2
evm.model.scaffold43539.5	15.6639	25.6253	-0.710124	0.0205228	sp|Q6DIB5|MEG10_MOUSE Multiple epidermal growth factor-like domains

					protein 10 OS=Mus musculus GN=Megf10 PE=1 SV=1
evm.model.scaffold111921.1	23.6349	47.327	-1.00174	0.00151774	sp|C3YWU0|FUCO_BRAFL Alpha-L-fucosidase OS=Branchiostoma floridae GN=BRAFLDRAFT_56888 PE=3 SV=2
evm.model.scaffold111921.9	2.08343	4.85158	-1.21949	0.00151774	sp|Q9R0T8|IKKE_MOUSE Inhibitor of nuclear factor kappa-B kinase subunit epsilon OS=Mus musculus GN=Ikbke PE=1 SV=2
evm.model.scaffold104945.42	11.8734	19.5078	-0.716312	0.023485	sp|Q8BHH9|T179A_MOUSE Transmembrane protein 179 OS=Mus musculus GN=Tmem179 PE=2 SV=1
evm.model.scaffold17183.54	35.4668	55.736	-0.652139	0.020248	sp|A5A6H4|ROA1_PANTR Heterogeneous nuclear ribonucleoprotein A1 OS=Pan troglodytes GN=HNRNPA1 PE=2 SV=1
evm.model.scaffold148433.112	4.62257	8.66831	-0.907057	0.00621905	sp|P38024|PUR6_CHICK Multifunctional protein ADE2 OS=Gallus gallus GN=AIRC PE=2 SV=1
evm.model.scaffold64459.7	5.73439	17.2071	-1.58529	0.000820679	sp|A4II20|EGR1_XENTR Early growth response protein 1 OS=Xenopus tropicalis GN=egr1 PE=2

					SV=2
evm.model.scaffold116119.104	6.35395	10.8601	-0.77331	0.0241881	sp|Q2KJJ8|PLD3_BOVIN Phospholipase D3 OS=Bos taurus GN=PLD3 PE=2 SV=1
evm.model.scaffold53733.12	5.68903	11.721	-1.04284	0.000820679	sp|P86413|OSR1_XENLA Protein odd-skipped-related 1 OS=Xenopus laevis GN=osr1 PE=2 SV=1
evm.model.scaffold68863.4	2.70914	4.50253	-0.732897	0.0208422	sp|Q60847|COCA1_MOUSE Collagen alpha-1(XII) chain OS=Mus musculus GN=Col12a1 PE=2 SV=3
evm.model.scaffold151177.4	1.983	4.61369	-1.21823	0.000820679	sp|P16157|ANK1_HUMAN Ankyrin-1 OS=Homo sapiens GN=ANK1 PE=1 SV=3
evm.model.scaffold9813.13	1.02792	2.09455	-1.02692	0.0254597	sp|Q5DTL9|S4A10_MOUSE Sodium-driven chloride bicarbonate exchanger OS=Mus musculus GN=Slc4a10 PE=1 SV=2
evm.model.scaffold170623.3	0.843858	4.3825	-2.37668	0.000820679	sp|Q99758|ABCA3_HUMAN ATP-binding cassette sub-family A member 3 OS=Homo sapiens GN=ABCA3 PE=1 SV=2
evm.model.scaffold143723.10	34.9757	58.9691	-0.753605	0.00755259	sp|P79782|TCF15_CHICK Transcription factor 15 OS=Gallus gallus GN=TCF15 PE=2 SV=2
evm.model.scaffold148487.6	0.186883	1.22459	-2.71209	0.00842602	sp|Q9DD78|TLR21_CHICK Toll-like

					receptor 2 type-1 OS=Gallus gallus GN=TLR2-1 PE=2 SV=1
evm.model.scaffold174561.38	2.76099	6.1838	-1.16331	0.000820679	sp|P53355|DAPK1_HUMAN Death-associated protein kinase 1 OS=Homo sapiens GN=DAPK1 PE=1 SV=6
evm.model.scaffold159893.21	30.3444	99.5712	-1.7143	0.000820679	sp|Q5RB02|CATC_PONAB Dipeptidyl peptidase 1 OS=Pongo abelii GN=CTSC PE=2 SV=1
evm.model.scaffold82159.11	6.44292	16.0462	-1.31645	0.0152063	sp|Q01414|ERG_LYTVA Transcriptional regulator ERG homolog (Fragment) OS=Lytechinus variegatus GN=ERG PE=3 SV=1
evm.model.scaffold82159.12	3.18563	7.53278	-1.24161	0.000820679	sp|Q90837|ERG_CHICK Transcriptional regulator Erg OS=Gallus gallus GN=ERG PE=2 SV=1
evm.model.scaffold56755.162	75.8226	219.638	-1.53442	0.0441781	sp|Q9U1H8|FACE2_DROME CAAX prenyl protease 2 OS=Drosophila melanogaster GN=Sras PE=2 SV=3
evm.model.scaffold150767.22	2.22802	4.59315	-1.04372	0.0129481	sp|D2GXS7|TRIM2_AILME Tripartite motif-containing protein 2 OS=Ailuropoda melanoleuca GN=TRIM2 PE=3 SV=1
evm.model.scaffold150767.24	23.0311	44.4473	-0.948509	0.000820679	sp|Q4PLW0|PLIN2_PIG Perilipin-2 OS=Sus scrofa GN=PLIN2 PE=2 SV=1

evm.model.scaffold176621.60	9.17126	14.2721	-0.638002	0.039382	sp|Q4U2R1|HERC2_MOUSE E3 ubiquitin-protein ligase HERC2 OS=Mus musculus GN=Herc2 PE=1 SV=3
evm.model.scaffold151663.11.1	15.8623	43.142	-1.44349	0.000820679	sp|Q80TL4|K1045_MOUSE Protein KIAA1045 OS=Mus musculus GN=Kiaa1045 PE=2 SV=2
evm.model.scaffold153701.5	5.66429	9.13524	-0.689547	0.0188471	sp|Q460N5|PAR14_HUMAN Poly [ADP-ribose] polymerase 14 OS=Homo sapiens GN=PARP14 PE=1 SV=3
evm.model.scaffold64247.10	6.66836	14.1832	-1.08878	0.000820679	sp|P41969|ELK1_MOUSE ETS domain-containing protein Elk-1 OS=Mus musculus GN=Elk1 PE=2 SV=3
evm.model.scaffold169829.2	10.7844	20.4218	-0.921167	0.000820679	sp|O77210|IF4E_APLCA Eukaryotic translation initiation factor 4E OS=Aplysia californica PE=1 SV=1
evm.model.scaffold39673.14	2.90267	5.75614	-0.987721	0.0141107	--
evm.model.scaffold95039.39	0.0863035	5.79072	-6.06818	0.0364617	sp|A2VE04|FRRS1_BOVIN Ferric-chelate reductase 1 OS=Bos taurus GN=FRRS1 PE=2 SV=1
evm.model.scaffold162515.34	6.31196	13.9507	-1.14418	0.0429196	--
evm.model.scaffold146247.19	0.705878	2.80197	-1.98895	0.00151774	sp|Q6NUT3|MFS12_HUMAN Major facilitator superfamily domain-containing protein 12

					OS=Homo sapiens GN=MFSD12 PE=1 SV=2
evm.model.scaffold140847.44.1	6.20739	14.3407	-1.20806	0.000820679	sp|P48316|GA45A_MOUSE Growth arrest and DNA damage-inducible protein GADD45 alpha OS=Mus musculus GN=Gadd45a PE=1 SV=1
evm.model.scaffold120171.2	2.21746	5.08888	-1.19844	0.0215514	--
evm.model.scaffold169189.14.1	7.13195	13.1689	-0.884762	0.00213042	sp|Q8VI63|MOB2_MOUSE MOB kinase activator 2 OS=Mus musculus GN=Mob2 PE=2 SV=1
evm.model.scaffold140631.1	7.38978	29.5678	-2.00042	0.000820679	sp|P19477|FIBA_PARPA Fibrinogen-like protein A OS=Parastichopus parvimensis PE=2 SV=1
evm.model.scaffold140631.2	18.2225	100.83	-2.46814	0.000820679	sp|O18783|PLMN_MACEU Plasminogen OS=Macropus eugenii GN=PLG PE=2 SV=1
evm.model.scaffold154539.10	5.49217	14.9786	-1.44745	0.000820679	sp|Q2EMV9|PAR14_MOUSE Poly [ADP-ribose] polymerase 14 OS=Mus musculus GN=Parp14 PE=1 SV=3
evm.model.scaffold164773.53	1.05984	5.53233	-2.38404	0.000820679	--
evm.model.scaffold164773.55	2.19267	5.76744	-1.39524	0.013343	--
evm.model.scaffold164773.57	0.540951	3.76597	-2.79945	0.000820679	--
evm.model.scaffold164773.59	4.60109	17.3859	-1.91787	0.000820679	--
evm.model.scaffold164773.58	2.5127	6.1003	-1.27964	0.00429787	--

evm.model.scaffold133599.1	5.86886	12.516	-1.09262	0.000820679	sp|P25160|ARL1_DROME ADP-ribosylation factor-like protein 1 OS=Drosophila melanogaster GN=Arl1 PE=2 SV=5
evm.model.scaffold157403.2	6.31505	10.8381	-0.779244	0.0347943	sp|Q7T2P0|MX1_ICTPU Interferon-induced GTP-binding protein Mx1 OS=Ictalurus punctatus GN=mx1 PE=2 SV=1
evm.model.scaffold52059.19	11.5036	18.2477	-0.665626	0.0347943	sp|Q9DD78|TLR21_CHICK Toll-like receptor 2 type-1 OS=Gallus gallus GN=TLR2-1 PE=2 SV=1
evm.model.scaffold108875.3	0	163.299	#NAME?	0.0299214	--
evm.model.scaffold169745.13	44.8008	89.1305	-0.992394	0.000820679	sp|Q4V7C7|ARP3_RAT Actin-related protein 3 OS=Rattus norvegicus GN=Actr3 PE=1 SV=1
evm.model.scaffold123417.20.1	12.6389	22.3085	-0.819728	0.00326205	sp|Q8C4V1|RHG24_MOUSE Rho GTPase-activating protein 24 OS=Mus musculus GN=Arhgap24 PE=1 SV=2
evm.model.scaffold166811.19	5.3058	8.54943	-0.688259	0.0282044	sp|Q92625|ANS1A_HUMAN Ankyrin repeat and SAM domain-containing protein 1A OS=Homo sapiens GN=ANKS1A PE=1 SV=4
evm.model.scaffold107255.30	2.93756	6.65679	-1.18021	0.000820679	sp|O18956|ENTP1_BOVIN Ectonucleoside triphosphate

					diphosphohydrolase 1 OS=Bos taurus GN=ENTPD1 PE=1 SV=1
evm.model.scaffold94005.1	0.119213	0.84774	-2.83008	0.0451727	sp|Q80ZW0|STK35_MOUSE Serine/threonine-protein kinase 35 OS=Mus musculus GN=Stk35 PE=2 SV=3
evm.model.scaffold141439.3	4.88589	8.59725	-0.815254	0.0299214	sp|Q8I7X7|NHP2_BRABE H/ACA ribonucleoprotein complex subunit 2-like protein OS=Branchiostoma belcheri PE=2 SV=1
evm.model.scaffold146097.32	75.1255	115.981	-0.626514	0.0316212	sp|Q01518|CAP1_HUMAN Adenylyl cyclase-associated protein 1 OS=Homo sapiens GN=CAP1 PE=1 SV=5
evm.model.scaffold72763.63	10.6089	17.683	-0.737088	0.0373071	sp|A9JTJ0|SIM15_XENLA Small integral membrane protein 15 OS=Xenopus laevis GN=smim15 PE=3 SV=1
evm.model.scaffold161513.22	2.63196	6.11199	-1.21551	0.000820679	sp|Q24400|MLP2_DROME Muscle LIM protein Mlp84B OS=Drosophila melanogaster GN=Mlp84B PE=1 SV=1
evm.model.scaffold142971.19	4.23031	9.93056	-1.23111	0.000820679	sp|Q96D03|DDT4L_HUMAN DNA damage-inducible transcript 4-like protein OS=Homo sapiens GN=DDIT4L PE=1 SV=1
evm.model.scaffold166635.3	1.7062	3.4307	-1.00771	0.0427039	sp|Q01460|DIAC_RAT

					Di-N-acetylchitobiase OS=Rattus norvegicus GN=Ctbs PE=1 SV=1
evm.model.scaffold100143.29	2.27341	4.62124	-1.02342	0.0302156	sp|Q5E9H7|DHB12_BOVIN Very-long-chain 3-oxoacyl-CoA reductase OS=Bos taurus GN=HSD17B12 PE=2 SV=1
evm.model.scaffold61415.17	1.1412	2.41848	-1.08355	0.0370657	sp|Q9DGB6|TLR22_CHICK Toll-like receptor 2 type-2 OS=Gallus gallus GN=TLR2-2 PE=2 SV=1
evm.model.scaffold91157.12	1.39137	3.17498	-1.19024	0.00578348	--
evm.model.scaffold86191.16	98.0119	405.49	-2.04864	0.000820679	--
evm.model.scaffold138569.16	1.58891	3.23316	-1.02491	0.0271297	sp|O60494|CUBN_HUMAN Cubilin OS=Homo sapiens GN=CUBN PE=1 SV=5
evm.model.scaffold138569.15	1.57765	3.07538	-0.962986	0.00326205	sp|Q9TU53|CUBN_CANFA Cubilin OS=Canis familiaris GN=CUBN PE=1 SV=1
evm.model.scaffold171661.24	0.914535	3.46447	-1.92152	0.000820679	sp|P51869|CP4F4_RAT Cytochrome P450 4F4 OS=Rattus norvegicus GN=Cyp4f4 PE=2 SV=1
evm.model.scaffold147445.25	23.0215	42.016	-0.867959	0.00429787	sp|P30682|GNAI_LYMST Guanine nucleotide-binding protein G(i) subunit alpha OS=Lymnaea stagnalis PE=2 SV=3
evm.model.scaffold4477.3_evm	1.23519	2.5512	-1.04644	0.0463231	sp|P79401|CP3AT_PIG Cytochrome

.model.scaffold4477.4					P450 3A29 OS=Sus scrofa GN=CYP3A29 PE=2 SV=1
evm.model.scaffold65315.50	1.90367	6.49611	-1.7708	0.000820679	--
evm.model.scaffold95051.25	0.260528	0.974729	-1.90356	0.0299214	sp|Q9SS90|RGLG1_ARATH E3 ubiquitin-protein ligase RGLG1 OS=Arabidopsis thaliana GN=RGLG1 PE=1 SV=1
evm.model.scaffold64247.16	3.54631	8.9434	-1.3345	0.000820679	sp|Q8WWQ8|STAB2_HUMAN Stabilin-2 OS=Homo sapiens GN=STAB2 PE=1 SV=3
evm.model.scaffold142253.9	5.70205	13.074	-1.19714	0.000820679	--
evm.model.scaffold30917.4	0.611682	2.09369	-1.77519	0.0373071	--
evm.model.scaffold142253.12	34.2687	71.4705	-1.06046	0.000820679	sp|Q90617|LAMP2_CHICK Lysosome-associated membrane glycoprotein 2 OS=Gallus gallus GN=LAMP2 PE=2 SV=1
evm.model.scaffold139609.9	0.537084	3.64981	-2.7646	0.00532397	sp|Q9ULJ7|ANR50_HUMAN Ankyrin repeat domain-containing protein 50 OS=Homo sapiens GN=ANKRD50 PE=1 SV=4
evm.model.scaffold64407.83	18.4791	37.6657	-1.02736	0.000820679	sp|P38657|PDIA3_BOVIN Protein disulfide-isomerase A3 OS=Bos taurus GN=PDIA3 PE=2 SV=1
evm.model.scaffold169873.43	1.77609	4.22574	-1.2505	0.00151774	sp|P45447|E78C_DROME Ecdysone-induced protein 78C

					OS=Drosophila melanogaster GN=Eip78C PE=2 SV=4
evm.model.scaffold167055.38	0.266918	1.26261	-2.24194	0.00271512	sp|Q5FWH7|S39AC_MOUSE Zinc transporter ZIP12 OS=Mus musculus GN=Slc39a12 PE=2 SV=1
evm.model.scaffold176203.5	13.9177	30.8151	-1.14671	0.000820679	sp|P70677|CASP3_MOUSE Caspase-3 OS=Mus musculus GN=Casp3 PE=1 SV=1
evm.model.scaffold2427.53	18.4014	68.5865	-1.89811	0.000820679	sp|Q8JHV9|BIR7A_XENLA Baculoviral IAP repeat-containing protein 7-A OS=Xenopus laevis GN=birc7-a PE=1 SV=1
evm.model.scaffold176479.16	0.690395	2.01922	-1.5483	0.0180929	sp|Q8VI93|OAS3_MOUSE 2'-5'-oligoadenylate synthase 3 OS=Mus musculus GN=Oas3 PE=2 SV=1
evm.model.scaffold136775.1	2.44757	5.48812	-1.16496	0.000820679	--
evm.model.scaffold116119.87	18.7894	36.1713	-0.944923	0.00532397	sp|Q5RCS8|VATD_PONAB V-type proton ATPase subunit D OS=Pongo abelii GN=ATP6V1D PE=2 SV=1
evm.model.scaffold153405.1	240.837	1396.77	-2.53596	0.000820679	sp|Q26636|CATL_SARPE Cathepsin L OS=Sarcophaga peregrina PE=1 SV=1
evm.model.scaffold164395.1	0.121871	0.579736	-2.25004	0.00326205	sp|Q8R422|CD109_MOUSE CD109 antigen OS=Mus musculus GN=Cd109 PE=2 SV=1

evm.model.scaffold164395.2	7.81124	14.7787	-0.9199	0.00151774	sp|P42839|VNX1_YEAST Low affinity vacuolar monovalent cation/H(+) antiporter OS=Saccharomyces cerevisiae (strain ATCC 204508 / S288c) GN=VNX1 PE=1 SV=1
evm.model.scaffold108969.26	10.8511	22.1687	-1.03069	0.00151774	sp|O65198|SODCP_MEDSA Superoxide dismutase [Cu-Zn], chloroplastic OS=Medicago sativa GN=SODCP PE=2 SV=1
evm.model.scaffold159969.15	12.8258	27.1991	-1.08451	0.000820679	sp|Q9VCA2|ORCT_DROME Organic cation transporter protein OS=Drosophila melanogaster GN=Orct PE=1 SV=1
evm.model.scaffold81993.2	11.6336	21.4947	-0.885679	0.000820679	sp|P53356|HTK16_HYDVU Tyrosine-protein kinase HTK16 OS=Hydra vulgaris GN=HTK16 PE=2 SV=1
evm.model.scaffold81993.8	8.90696	16.6193	-0.899851	0.00271512	sp|Q6PFS7|ATG3_DANRE Ubiquitin-like-conjugating enzyme ATG3 OS=Danio rerio GN=atg3 PE=2 SV=1
evm.model.scaffold148011.13	10.331	16.4952	-0.675068	0.0441781	sp|A5PKG7|KCTD5_BOVIN BTB/POZ domain-containing protein KCTD5 OS=Bos taurus GN=KCTD5 PE=2 SV=1

evm.model.scaffold121775.10	7.43657	12.9545	-0.800749	0.00712329	sp|Q80TD3|FNIP2_MOUSE Folliculin-interacting protein 2 OS=Mus musculus GN=Fnip2 PE=1 SV=2
evm.model.scaffold140847.75.1	0.24315	1.50698	-2.63174	0.000820679	sp|Q24155|TRUNK_DROME Protein trunk OS=Drosophila melanogaster GN=trk PE=3 SV=1
evm.model.scaffold147865.10	9.71939	27.7077	-1.51135	0.000820679	--
evm.model.scaffold142971.90	3.40095	5.65289	-0.733052	0.0314536	sp|Q29451|MA2B1_BOVIN Lysosomal alpha-mannosidase OS=Bos taurus GN=MAN2B1 PE=1 SV=4
evm.model.scaffold142971.92	13.3495	65.8604	-2.30262	0.000820679	sp|C3YWU0|FUCO_BRAFL Alpha-L-fucosidase OS=Branchiostoma floridae GN=BRAFLDRAFT_56888 PE=3 SV=2
evm.model.scaffold157909.1	2.61732	5.98112	-1.19232	0.0191834	sp|O88447|KLC1_MOUSE Kinesin light chain 1 OS=Mus musculus GN=Klc1 PE=1 SV=3
evm.model.scaffold102425.2	2.83874	10.0248	-1.82025	0.000820679	sp|P22125|RAB1_DIPOM Ras-related protein ORAB-1 OS=Diplobatis ommata PE=2 SV=1
evm.model.scaffold120781.2	13.3418	41.8923	-1.65074	0.00532397	sp|Q86UW6|N4BP2_HUMAN NEDD4-binding protein 2 OS=Homo sapiens GN=N4BP2 PE=1 SV=2
evm.model.scaffold124269.42	9.26897	15.4854	-0.740427	0.020248	sp|O46404|P55G_BOVIN

					Phosphatidylinositol 3-kinase regulatory subunit gamma OS=Bos taurus GN=PIK3R3 PE=2 SV=1
evm.model.scaffold162515.8	110.673	168.544	-0.606826	0.0416339	sp|P49918|CDN1C_HUMAN Cyclin-dependent kinase inhibitor 1C OS=Homo sapiens GN=CDKN1C PE=1 SV=1
evm.model.scaffold33507.14	3.01606	6.38799	-1.0827	0.020248	sp|A1A5K6|TBC24_XENLA TBC1 domain family member 24 OS=Xenopus laevis GN=tbc1d24 PE=2 SV=1
evm.model.scaffold154705.23	15.3086	69.7453	-2.18775	0.000820679	sp|P28799|GRN_HUMAN Granulins OS=Homo sapiens GN=GRN PE=1 SV=2
evm.model.scaffold111921.55	1.15239	5.76322	-2.32224	0.000820679	sp|P52926|HMGA2_HUMAN High mobility group protein HMGI-C OS=Homo sapiens GN=HMGA2 PE=1 SV=1
evm.model.scaffold28061.9	14.3143	23.5564	-0.718658	0.00933293	sp|Q01664|TFAP4_HUMAN Transcription factor AP-4 OS=Homo sapiens GN=TFAP4 PE=1 SV=2
evm.model.scaffold123009.79.1	24.5242	45.7587	-0.899841	0.00151774	sp|Q9EQT3|RHOU_MOUSE Rho-related GTP-binding protein RhoU OS=Mus musculus GN=Rhou PE=2 SV=1

evm.model.scaffold143135.32	7.52529	14.5356	-0.949777	0.000820679	sp|Q9P2F6|RHG20_HUMAN Rho GTPase-activating protein 20 OS=Homo sapiens GN=ARHGAP20 PE=1 SV=2
evm.model.scaffold86191.27	3.85347	15.3087	-1.99013	0.000820679	--
evm.model.scaffold99125.1	3.71261	7.70094	-1.0526	0.000820679	sp|Q5ZJU3|ASNS_CHICK Asparagine synthetase [glutamine-hydrolyzing] OS=Gallus gallus GN=ASNS PE=2 SV=3
evm.model.scaffold79123.16	0.215493	6.71828	-4.96238	0.00151774	--
evm.model.scaffold98043.7	0.810027	2.0723	-1.35519	0.0318917	--
evm.model.scaffold77517.18.1	2.25389	4.21132	-0.901856	0.00429787	sp|P0C6B8|SVEP1_RAT Sushi, von Willebrand factor type A, EGF and pentraxin domain-containing protein 1 OS=Rattus norvegicus GN=Svep1 PE=1 SV=1
evm.model.scaffold175929.13	1.88998	6.24615	-1.72459	0.000820679	sp|Q5ZIJ9|MIB2_CHICK E3 ubiquitin-protein ligase MIB2 OS=Gallus gallus GN=MIB2 PE=2 SV=1
evm.model.scaffold141451.24	0.0885768	1.42781	-4.01073	0.0105331	--
evm.model.scaffold81773.84	173.578	329.165	-0.923228	0.00271512	sp|A4IIN5|TISD_XENTR Zinc finger protein 36, C3H1 type-like 2 OS=Xenopus tropicalis GN=zfp36l2 PE=2 SV=2
evm.model.scaffold88753.76	2.93603	5.6079	-0.933594	0.0359113	sp|Q8WNR0|COPT1_PIG High

					affinity copper uptake protein 1 OS=Sus scrofa GN=SLC31A1 PE=2 SV=1
evm.model.scaffold73223.26	0.0732001	0.69398	-3.24498	0.00532397	sp|Q06852|SLAP1_CLOTH Cell surface glycoprotein 1 OS=Clostridium thermocellum (strain ATCC 27405 / DSM 1237 / NBRC 103400 / NCIMB 10682 / NRRL B-4536 / VPI 7372) GN=olpB PE=3 SV=2
evm.model.scaffold71643.1	0.0927407	0.5662	-2.61004	0.000820679	sp|Q8BV79|TRNK1_MOUSE TPR and ankyrin repeat-containing protein 1 OS=Mus musculus GN=Trank1 PE=2 SV=3
evm.model.scaffold124473.10	3.13001	6.35858	-1.02254	0.00888556	sp|Q6B9X6|VWKA_DICDI Alpha-protein kinase vwkA OS=Dictyostelium discoideum GN=vwkA PE=1 SV=1
evm.model.scaffold134743.18	7.46981	28.1076	-1.91181	0.000820679	sp|O42342|SOX7_XENLA Transcription factor Sox-7 OS=Xenopus laevis GN=sox7 PE=2 SV=1
evm.model.scaffold164773.11	9.9789	20.2153	-1.01849	0.000820679	sp|P08953|TOLL_DROME Protein toll OS=Drosophila melanogaster GN=Tl PE=1 SV=1
evm.model.scaffold164773.12	0.47756	1.09986	-1.20356	0.0486765	sp|P08953|TOLL_DROME Protein toll

					OS=Drosophila melanogaster GN=Tl PE=1 SV=1
evm.model.scaffold128495.3	0.614853	9.62838	-3.96898	0.000820679	sp|P46436|GST1_ASCSU Glutathione S-transferase 1 OS=Ascaris suum GN=GST1 PE=1 SV=3
evm.model.scaffold103395.80	1.42285	3.90634	-1.45704	0.00151774	sp|P78417|GSTO1_HUMAN Glutathione S-transferase omega-1 OS=Homo sapiens GN=GSTO1 PE=1 SV=2
evm.model.scaffold165923.13	79.324	139.749	-0.817013	0.00213042	sp|P26043|RADI_MOUSE Radixin OS=Mus musculus GN=Rdx PE=1 SV=3
evm.model.scaffold82057.47	26.1032	65.8624	-1.33523	0.000820679	--
evm.model.scaffold168187.23	2.29834	5.57051	-1.27722	0.00888556	--
evm.model.scaffold142101.83	0.199301	1.06933	-2.42369	0.00755259	sp|Q01603|PERO_DROME Peroxidase OS=Drosophila melanogaster GN=Pxd PE=2 SV=2
evm.model.scaffold26189.8	44.2087	78.6006	-0.830209	0.0476583	sp|P60522|GBRL2_RAT Gamma-aminobutyric acid receptor-associated protein-like 2 OS=Rattus norvegicus GN=Gabarapl2 PE=3 SV=1
evm.model.scaffold64247.1	1.79763	3.30221	-0.877334	0.0327195	sp|Q24118|PIGE_DROME Protein pigeon OS=Drosophila melanogaster GN=pigeon PE=2 SV=2

evm.model.scaffold107225.70	5.10505	8.71392	-0.771395	0.00842602	--
evm.model.scaffold142329.10	27.8216	49.8093	-0.840209	0.00151774	--
evm.model.scaffold73677.15	5.20834	16.1076	-1.62884	0.000820679	--
evm.model.scaffold43385.6	4.61319	10.2383	-1.15014	0.000820679	sp|Q91XS8|ST17B_RAT Serine/threonine-protein kinase 17B OS=Rattus norvegicus GN=Stk17b PE=1 SV=1
evm.model.scaffold52321.23	25.1458	39.8859	-0.665562	0.0195535	--
evm.model.scaffold174343.38	11.6838	22.2369	-0.928454	0.00429787	sp|Q9U5N1|VATC_MANSE V-type proton ATPase subunit C OS=Manduca sexta PE=2 SV=1
evm.model.scaffold156791.22	0.674089	1.80794	-1.42333	0.0316212	sp|Q5F4B8|S46A3_CHICK Solute carrier family 46 member 3 OS=Gallus gallus GN=SLC46A3 PE=2 SV=1
evm.model.scaffold58729.9	2.93088	5.51329	-0.91158	0.0418385	sp|Q6NT55|CP4FN_HUMAN Cytochrome P450 4F22 OS=Homo sapiens GN=CYP4F22 PE=2 SV=1
evm.model.scaffold154529.14	5.51562	12.3024	-1.15735	0.000820679	sp|Q56H79|NPSR1_MACMU Neuropeptide S receptor OS=Macaca mulatta GN=NPSR1 PE=2 SV=1
evm.model.scaffold154529.19	0.334233	9.01246	-4.75299	0.000820679	--
evm.model.scaffold174219.7	1.7642	5.21427	-1.56345	0.00842602	--
evm.model.scaffold138629.34	10.6509	20.0358	-0.911608	0.00429787	sp|Q4LDE5|SVEP1_HUMAN Sushi, von Willebrand factor type A, EGF and pentraxin domain-containing protein 1

					OS=Homo sapiens GN=SVEP1 PE=1 SV=3
evm.model.scaffold128325.25	7.44824	15.6455	-1.07077	0.0173453	--
evm.model.scaffold128325.20	11.7891	46.7576	-1.98775	0.000820679	--
evm.model.scaffold120453.12	4.92316	15.6751	-1.67082	0.000820679	--
evm.model.scaffold20967.17	0.645201	3.05074	-2.24134	0.000820679	--
evm.model.scaffold5015.16	3.30864	6.24775	-0.917099	0.0191834	sp|P45481|CBP_MOUSE CREB-binding protein OS=Mus musculus GN=Crebbp PE=1 SV=3
evm.model.scaffold5015.13	3.86353	9.77804	-1.33962	0.0156116	sp|Q63151|ACSL3_RAT Long-chain-fatty-acid--CoA ligase 3 OS=Rattus norvegicus GN=Acsl3 PE=1 SV=1
evm.model.scaffold159893.6	4.61103	8.61945	-0.902506	0.00480132	sp|O73853|CP17A_ICTPU Steroid 17-alpha-hydroxylase/17,20 lyase OS=Ictalurus punctatus GN=cyp17a1 PE=2 SV=1
evm.model.scaffold107973.1	0	4.42287	#NAME?	0.000820679	--
evm.model.scaffold158265.8	5.4074	11.482	-1.08636	0.0169923	sp|A2VCW9|AASS_RAT Alpha-aminoadipic semialdehyde synthase, mitochondrial OS=Rattus norvegicus GN=Aass PE=2 SV=1
evm.model.scaffold158265.9	4.34905	10.9876	-1.3371	0.000820679	sp|A8E657|AASS_BOVIN Alpha-aminoadipic semialdehyde synthase, mitochondrial OS=Bos

					taurus GN=AASS PE=2 SV=1
evm.model.scaffold27129.39	5.1301	9.51194	-0.890751	0.00326205	sp|Q2WEA5|TRPM1_RAT Transient receptor potential cation channel subfamily M member 1 OS=Rattus norvegicus GN=Trpm1 PE=2 SV=1
evm.model.scaffold27129.37	2.56042	16.9267	-2.72485	0.000820679	--
evm.model.scaffold27129.36	0.0960201	0.74878	-2.96313	0.00621905	--
evm.model.scaffold164773.43	2.63946	8.27721	-1.6489	0.00532397	--
evm.model.scaffold172453.31	2.27305	5.73581	-1.33537	0.000820679	sp|Q9TTK4|LYST_BOVIN Lysosomal-trafficking regulator OS=Bos taurus GN=LYST PE=1 SV=1
evm.model.scaffold60125.3	2.61976	8.95481	-1.77323	0.000820679	sp|Q9LQU4|PCR2_ARATH Protein PLANT CADMIUM RESISTANCE 2 OS=Arabidopsis thaliana GN=PCR2 PE=1 SV=1
evm.model.scaffold2059.40	0	0.768417	#NAME?	0.000820679	--
evm.model.scaffold62351.8	4.37188	7.28563	-0.7368	0.0305208	sp|P25210|NFYB_PETMA Nuclear transcription factor Y subunit beta OS=Petromyzon marinus GN=NFYB PE=2 SV=1
evm.model.scaffold823.35	1.42924	3.18016	-1.15385	0.0316212	sp|Q8BFR4|GNS_MOUSE N-acetylglucosamine-6-sulfatase OS=Mus musculus GN=Gns PE=2 SV=1
evm.model.scaffold88177.11	10.2219	18.6395	-0.866708	0.00213042	--

evm.model.scaffold48653.3	96.9824	162.607	-0.745593	0.00712329	sp|Q05974|RAB1A_LYMST Ras-related protein Rab-1A OS=Lymnaea stagnalis GN=RAB1A PE=2 SV=1
evm.model.scaffold175229.24	2.86262	5.58261	-0.963606	0.0311397	sp|P25911|LYN_MOUSE Tyrosine-protein kinase Lyn OS=Mus musculus GN=Lyn PE=1 SV=4
evm.model.scaffold47967.1	5.63884	13.3144	-1.23951	0.00621905	sp|Q5ZMC2|MYO1G_CHICK Unconventional myosin-Ig OS=Gallus gallus GN=MYO1G PE=2 SV=1
evm.model.scaffold126619.17	8.11846	17.5405	-1.11141	0.000820679	sp|Q5RDC9|S35D2_PONAB UDP-N-acetylglucosamine/UDP-glucose/GDP-mannose transporter OS=Pongo abelii GN=SLC35D2 PE=2 SV=1
evm.model.scaffold173885.34	7.1027	11.7345	-0.724312	0.0191834	sp|A0MTA1|APEX1_DANRE DNA-(apurinic or apyrimidinic site) lyase OS=Danio rerio GN=apex1 PE=1 SV=1
evm.model.scaffold153941.5	9.96258	16.14	-0.696046	0.0248731	sp|Q02874|H2AY_RAT Core histone macro-H2A.1 OS=Rattus norvegicus GN=H2afy PE=1 SV=4
evm.model.scaffold152681.36	1.25657	14.3913	-3.51763	0.000820679	sp|P15541|AMPN_RABIT Aminopeptidase N OS=Oryctolagus cuniculus GN=ANPEP PE=1 SV=4

evm.model.scaffold152681.35	0.570668	7.74363	-3.76229	0.00380231	sp|P15145|AMPN_PIG Aminopeptidase N OS=Sus scrofa GN=ANPEP PE=1 SV=4
evm.model.scaffold42935.34	6.18513	10.1705	-0.717515	0.0125549	sp|P11387|TOP1_HUMAN DNA topoisomerase 1 OS=Homo sapiens GN=TOP1 PE=1 SV=2
evm.model.scaffold112961.8	1.28471	8.64626	-2.75063	0.0105331	--
evm.model.scaffold144475.54	4.28843	83.4939	-4.28315	0.000820679	--
evm.model.scaffold72763.22	0.258667	0.778098	-1.58885	0.0264598	sp|Q5UR67|YR617_MIMIV Uncharacterized protein R617 OS=Acanthamoeba polyphaga mimivirus GN=MIMI_R617 PE=4 SV=1
evm.model.scaffold157527.3	1.97946	5.51943	-1.47941	0.000820679	sp|O70496|CLCN7_MOUSE H(+)/Cl(-) exchange transporter 7 OS=Mus musculus GN=Clcn7 PE=1 SV=1
evm.model.scaffold76817.20	18.8117	34.1244	-0.85917	0.00213042	sp|Q90972|RNF13_CHICK E3 ubiquitin-protein ligase RNF13 OS=Gallus gallus GN=RNF13 PE=1 SV=1
evm.model.scaffold135471.16	1.33237	2.85548	-1.09974	0.00480132	sp|Q6NUD7|ZN521_XENLA Zinc finger protein 521 OS=Xenopus laevis GN=znf521 PE=2 SV=1
evm.model.scaffold135471.13	2.79165	5.14228	-0.88129	0.0163478	sp|Q0VE82|CPNE7_MOUSE Copine-7 OS=Mus musculus GN=Cpne7 PE=2

					SV=1
evm.model.scaffold125695.1	1.96841	8.91967	-2.17996	0.0129481	sp|O35448|PPT2_MOUSE Lysosomal thioesterase PPT2 OS=Mus musculus GN=Ppt2 PE=2 SV=1
evm.model.scaffold72197.11	1.40571	4.85006	-1.7867	0.000820679	sp|Q4UMH6|Y381_RICFE Putative ankyrin repeat protein RF_0381 OS=Rickettsia felis (strain ATCC VR-1525 / URRWXCal2) GN=RF_0381 PE=3 SV=1
evm.model.scaffold146755.37	0.68455	1.98045	-1.5326	0.00532397	sp|Q9TSP2|TLR4_PAPAN Toll-like receptor 4 OS=Papio anubis GN=TLR4 PE=3 SV=1
evm.model.scaffold66661.30	36.3491	68.9294	-0.923202	0.00480132	sp|Q9VLM5|DAD1_DROME Dolichyl-diphosphooligosaccharide--protein glycosyltransferase subunit DAD1 OS=Drosophila melanogaster GN=l(2)k12914 PE=3 SV=1
evm.model.scaffold75523.2	8.74397	18.1605	-1.05444	0.000820679	--
evm.model.scaffold156305.14	4.83738	10.4642	-1.11317	0.00151774	--
evm.model.scaffold64407.25	0.736244	3.68667	-2.32406	0.000820679	sp|Q8NFW1|COMA1_HUMAN Collagen alpha-1(XXII) chain OS=Homo sapiens GN=COL22A1 PE=2 SV=2
evm.model.scaffold52869.1	44.5364	107.341	-1.26914	0.000820679	--
evm.model.scaffold52869.4	1.44986	8.43421	-2.54034	0.000820679	--

evm.model.scaffold90899.38_evm.model.scaffold90899.41	2.98699	6.25063	-1.06531	0.00213042	sp|Q6DN14|MCTP1_HUMAN Multiple C2 and transmembrane domain-containing protein 1 OS=Homo sapiens GN=MCTP1 PE=2 SV=2
evm.model.scaffold141451.46	1.09726	2.61184	-1.25116	0.0261282	sp|Q8H191|PAO4_ARATH Probable polyamine oxidase 4 OS=Arabidopsis thaliana GN=PAO4 PE=1 SV=1
evm.model.scaffold90803.8	26.0663	54.624	-1.06735	0.000820679	sp|Q2KJ16|PHKG2_BOVIN Phosphorylase b kinase gamma catalytic chain, liver/testis isoform OS=Bos taurus GN=PHKG2 PE=2 SV=1
evm.model.scaffold90803.6	0.172304	0.789639	-2.19623	0.0332993	sp|Q9BX70|BTBD2_HUMAN BTB/POZ domain-containing protein 2 OS=Homo sapiens GN=BTBD2 PE=1 SV=1
evm.model.scaffold160259.4	1.75385	5.2057	-1.56956	0.000820679	sp|O08623|SQSTM_RAT Sequestosome-1 OS=Rattus norvegicus GN=Sqstm1 PE=1 SV=1
evm.model.scaffold59055.1_evm.model.scaffold59055.2	0.267621	0.804755	-1.58836	0.020248	sp|Q9UGM3|DMBT1_HUMAN Deleted in malignant brain tumors 1 protein OS=Homo sapiens GN=DMBT1 PE=1 SV=2
evm.model.scaffold3243.16	1.47529	5.2305	-1.82595	0.000820679	--
evm.model.scaffold151467.170	1.62038	3.13237	-0.950921	0.0148208	sp|Q9CXB8|ALPK1_MOUSE

					Alpha-protein kinase 1 OS=Mus musculus GN=Alpk1 PE=2 SV=2
evm.model.scaffold32961.3	0.341221	2.10786	-2.627	0.0238645	sp|P82596|PLC_HALLA Perlucin OS=Haliotis laevigata PE=1 SV=3
evm.model.scaffold138569.96_evm.model.scaffold138569.97	10.1711	25.8814	-1.34743	0.000820679	sp|Q8K2A1|GULP1_MOUSE PTB domain-containing engulfment adapter protein 1 OS=Mus musculus GN=Gulp1 PE=1 SV=1
evm.model.scaffold72763.109	207.249	1064.77	-2.3611	0.000820679	sp|Q9U943|APLP_LOCMI Apolipophorins OS=Locusta migratoria PE=1 SV=2
evm.model.scaffold110559.25	8.22755	15.2029	-0.88581	0.00151774	sp|P11274|BCR_HUMAN Breakpoint cluster region protein OS=Homo sapiens GN=BCR PE=1 SV=2
evm.model.scaffold174343.14_evm.model.scaffold174343.15	1.4291	4.06314	-1.50749	0.000820679	sp|Q28039|SC6A9_BOVIN Sodium- and chloride-dependent glycine transporter 1 OS=Bos taurus GN=SLC6A9 PE=2 SV=1
evm.model.scaffold149395.3	1.09837	3.85161	-1.81009	0.00429787	--
evm.model.scaffold149395.6	43.2272	115.244	-1.41468	0.000820679	sp|Q95VF7|PRO1B_ACACA Profilin-1B OS=Acanthamoeba castellanii PE=1 SV=3
evm.model.scaffold64247.22	6.0345	13.5398	-1.1659	0.00213042	sp|Q8WWQ8|STAB2_HUMAN Stabilin-2 OS=Homo sapiens GN=STAB2 PE=1 SV=3

evm.model.scaffold133643.3	5.94302	13.4501	-1.17835	0.000820679	--
evm.model.scaffold14779.4	11.1741	37.0486	-1.72925	0.000820679	sp|Q24372|LACH_DROME Lachesin OS=Drosophila melanogaster GN=Lac PE=1 SV=2
evm.model.scaffold14779.1	7.69714	16.2916	-1.08173	0.0276479	--
evm.model.scaffold44571.27	2.80179	6.149	-1.134	0.000820679	--
evm.model.scaffold69945.30	5.29665	11.7537	-1.14997	0.000820679	sp|A3FEM2|FEV_DANRE Protein FEV OS=Danio rerio GN=fev PE=2 SV=1
evm.model.scaffold164875.8	10.6611	18.3092	-0.780218	0.00800162	sp|Q8BIE6|FRM4A_MOUSE FERM domain-containing protein 4A OS=Mus musculus GN=Frmd4a PE=1 SV=2
evm.model.scaffold71897.45	7.41095	12.5869	-0.764193	0.0299214	--
evm.model.scaffold125871.12	3.16514	8.75869	-1.46844	0.000820679	sp|P70031|CCKAR_XENLA Cholecystokinin receptor OS=Xenopus laevis GN=cckar PE=2 SV=1
evm.model.scaffold176479.13	5.58876	12.1732	-1.12311	0.00213042	--
evm.model.scaffold93679.82	57.1076	111.953	-0.971143	0.000820679	sp|Q6DDK3|DOXA1_XENLA Dual oxidase maturation factor 1 OS=Xenopus laevis GN=duoxa1 PE=2 SV=1
evm.model.scaffold149739.6	1.10104	3.06848	-1.47865	0.0208422	--
evm.model.scaffold149739.7	2.16373	5.94898	-1.45912	0.000820679	sp|P09917|LOX5_HUMAN Arachidonate 5-lipoxygenase

					OS=Homo sapiens GN=ALOX5 PE=1 SV=2
evm.model.scaffold65899.19	1.63034	3.99456	-1.29286	0.000820679	sp|Q99M80|PTPRT_MOUSE Receptor-type tyrosine-protein phosphatase T OS=Mus musculus GN=Ptprt PE=2 SV=2
evm.model.scaffold65899.18	1.68866	5.78516	-1.77648	0.000820679	sp|Q15262|PTPRK_HUMAN Receptor-type tyrosine-protein phosphatase kappa OS=Homo sapiens GN=PTPRK PE=1 SV=2
evm.model.scaffold172049.20	17.4529	32.4857	-0.896341	0.00213042	--
evm.model.scaffold161635.3_evm.model.scaffold161635.4	8.99952	18.2215	-1.01772	0.000820679	sp|Q7T2P0|MX1_ICTPU Interferon-induced GTP-binding protein Mx1 OS=Ictalurus punctatus GN=mx1 PE=2 SV=1
evm.model.scaffold176323.8	12.9222	36.2387	-1.48768	0.000820679	sp|O77460|IPYR_DROME Inorganic pyrophosphatase OS=Drosophila melanogaster GN=Nurf-38 PE=1 SV=3
evm.model.scaffold107255.61	2.22468	5.81317	-1.38573	0.000820679	sp|Q498K0|GALC_XENLA Galactocerebrosidase OS=Xenopus laevis GN=galc PE=2 SV=2
evm.model.scaffold26071.68	1.86477	7.52924	-2.01351	0.00480132	sp|Q9JID6|ACSL1_CAVPO Long-chain-fatty-acid--CoA ligase 1 OS=Cavia porcellus GN=ACSL1 PE=2 SV=1

evm.model.scaffold163753.6	15.8295	38.1117	-1.26762	0.000820679	sp|Q9JM83|CALM4_MOUSE Calmodulin-4 OS=Mus musculus GN=Calm4 PE=2 SV=2
evm.model.scaffold163753.5	9.40358	28.5513	-1.60227	0.000820679	sp|P10246|TNNC2_MELGA Troponin C, skeletal muscle OS=Meleagris gallopavo GN=TNNC2 PE=1 SV=2
evm.model.scaffold146097.43	15.7234	29.8233	-0.923528	0.00213042	sp|Q13637|RAB32_HUMAN Ras-related protein Rab-32 OS=Homo sapiens GN=RAB32 PE=1 SV=3
evm.model.scaffold108831.4	13.6412	36.817	-1.4324	0.000820679	sp|P55859|PNPH_BOVIN Purine nucleoside phosphorylase OS=Bos taurus GN=PNP PE=1 SV=3
evm.model.scaffold65971.100	24.7413	107.269	-2.11624	0.00380231	sp|P50430|ARSB_RAT Arylsulfatase B OS=Rattus norvegicus GN=Arsb PE=2 SV=2
evm.model.scaffold26071.25_evm.model.scaffold26071.26	13.0484	19.5939	-0.586529	0.0404844	sp|Q5E9X2|MP2K6_BOVIN Dual specificity mitogen-activated protein kinase kinase 6 OS=Bos taurus GN=MAP2K6 PE=2 SV=1
evm.model.scaffold26693.14	2.11808	5.12109	-1.2737	0.048902	sp|Q8K211|COPT1_MOUSE High affinity copper uptake protein 1 OS=Mus musculus GN=Slc31a1 PE=2 SV=1
evm.model.scaffold115693.16	24.4968	59.4989	-1.28027	0.0342138	sp|Q6CGG3|FKBP2_YARLI FK506-binding protein 2 OS=Yarrowia

					lipolytica (strain CLIB 122 / E 150) GN=FPR2 PE=3 SV=1
evm.model.scaffold150495.2	1.80514	4.80561	-1.41261	0.00429787	sp|Q689D1|TLR2_CANFA Toll-like receptor 2 OS=Canis familiaris GN=TLR2 PE=2 SV=1
evm.model.scaffold154529.30	25.0179	55.2365	-1.14266	0.000820679	sp|Q2TBI0|LBP_BOVIN Lipopolysaccharide-binding protein OS=Bos taurus GN=LBP PE=2 SV=1
evm.model.scaffold3565.15	0.799798	2.47659	-1.63065	0.000820679	sp|Q5F478|ANR44_CHICK Serine/threonine-protein phosphatase 6 regulatory ankyrin repeat subunit B OS=Gallus gallus GN=ANKRD44 PE=2 SV=1
evm.model.scaffold126661.5	25.5726	66.499	-1.37873	0.000820679	sp|P58307|OX1R_MOUSE Orexin receptor type 1 OS=Mus musculus GN=Hcrtr1 PE=2 SV=3
evm.model.scaffold144337.10	14.0767	37.1469	-1.39993	0.000820679	sp|Q24799|MYPH_ECHGR Myophilin OS=Echinococcus granulosus PE=2 SV=1
evm.model.scaffold3099.9	2.68835	13.0344	-2.27753	0.000820679	--
evm.model.scaffold147433.22	51.8343	103.372	-0.995862	0.000820679	sp|P28491|CALR_PIG Calreticulin OS=Sus scrofa GN=CALR PE=1 SV=3
evm.model.scaffold147433.26	2.21952	3.45168	-0.637048	0.0437576	sp|Q54KA7|SECG_DICDI Ankyrin repeat, PH and SEC7 domain containing protein secG

					OS=Dictyostelium discoideum GN=secG PE=2 SV=1
evm.model.scaffold61991.9	4.69594	15.117	-1.68669	0.000820679	sp|P54802|ANAG_HUMAN Alpha-N-acetylglucosaminidase OS=Homo sapiens GN=NAGLU PE=1 SV=2
evm.model.scaffold155585.5	37.0887	69.7477	-0.911168	0.00151774	sp|Q9V4X2|PGSC2_DROME Peptidoglycan-recognition protein SC2 OS=Drosophila melanogaster GN=PGRP-SC2 PE=2 SV=1
evm.model.scaffold90713.19	0.719784	6.19132	-3.10461	0.000820679	--
evm.model.scaffold72861.2	1.00555	2.97137	-1.56314	0.000820679	sp|Q9UA35|S28A3_EPTST Solute carrier family 28 member 3 OS=Eptatretus stoutii GN=SLC28A3 PE=2 SV=1
evm.model.scaffold72861.4	0.801601	65.1284	-6.34426	0.000820679	--
evm.model.scaffold51453.38	4.83563	10.0215	-1.05132	0.00972356	sp|Q9NZC3|GDE1_HUMAN Glycerophosphodiester phosphodiesterase 1 OS=Homo sapiens GN=GDE1 PE=1 SV=1
evm.model.scaffold100263.3	6.21173	15.3036	-1.30081	0.048902	sp|Q2V897|TLR2_BOSTR Toll-like receptor 2 OS=Boselaphus tragocamelus GN=TLR2 PE=2 SV=1
evm.model.scaffold100263.2	4.18809	9.60093	-1.19688	0.00712329	sp|B3Y613|TLR2_PANTR Toll-like receptor 2 OS=Pan troglodytes

					GN=TLR2 PE=2 SV=1
evm.model.scaffold70117.2	0.710525	3.59913	-2.34069	0.000820679	sp|Q5DW34|EHMT1_MOUSE Histone-lysine N-methyltransferase EHMT1 OS=Mus musculus GN=Ehmt1 PE=1 SV=2
evm.model.scaffold176379.1	1.63765	7.61515	-2.21724	0.00972356	--
evm.model.scaffold7521.15	1.2039	5.70658	-2.24492	0.000820679	sp|C3YWU0|FUCO_BRAFL Alpha-L-fucosidase OS=Branchiostoma floridae GN=BRAFLDRAFT_56888 PE=3 SV=2
evm.model.scaffold7521.11	4.1581	14.5379	-1.80582	0.000820679	sp|P04066|FUCO_HUMAN Tissue alpha-L-fucosidase OS=Homo sapiens GN=FUCA1 PE=1 SV=4
evm.model.scaffold59345.5	1.93466	3.47267	-0.843966	0.0311397	sp|O95294|RASL1_HUMAN RasGAP-activating-like protein 1 OS=Homo sapiens GN=RASAL1 PE=1 SV=3
evm.model.scaffold174483.4	5.07047	11.0618	-1.12539	0.000820679	sp|Q9TUP7|OX2R_CANFA Orexin receptor type 2 OS=Canis familiaris GN=HCRTR2 PE=1 SV=1
evm.model.scaffold174483.1	4.62193	9.06169	-0.971285	0.0109379	sp|Q7M456|RNOY_CRAGI Ribonuclease Oy OS=Crassostrea gigas PE=1 SV=1
evm.model.scaffold89891.20	1.97074	5.48373	-1.47642	0.000820679	sp|Q5XGD7|TM45B_XENTR Transmembrane protein 45B

					OS=Xenopus tropicalis GN=tmem45b PE=2 SV=1
evm.model.scaffold83847.5	1.94739	3.80646	-0.966912	0.0254597	sp|Q8TDG4|HELQ_HUMAN Helicase POLQ-like OS=Homo sapiens GN=HELQ PE=1 SV=2
evm.model.scaffold55821.15	1.55293	6.33922	-2.02932	0.000820679	sp|Q8NHV1|GIMA7_HUMAN GTPase IMAP family member 7 OS=Homo sapiens GN=GIMAP7 PE=1 SV=1
evm.model.scaffold61991.13	10.686	23.1739	-1.11678	0.000820679	sp|Q99JY8|LPP3_MOUSE Lipid phosphate phosphohydrolase 3 OS=Mus musculus GN=Ppap2b PE=1 SV=1
evm.model.scaffold65971.2	0.735838	2.35222	-1.67656	0.000820679	sp|Q90419|TWHH_DANRE Tiggy-winkle hedgehog protein OS=Danio rerio GN=shhb PE=1 SV=1
evm.model.scaffold166837.89	3.52794	6.15347	-0.802576	0.0324675	--
evm.model.scaffold166837.78_evm.model.scaffold166837.80	1.30694	2.36642	-0.856511	0.014476	sp|Q2KJY2|KI26B_HUMAN Kinesin-like protein KIF26B OS=Homo sapiens GN=KIF26B PE=2 SV=1
evm.model.scaffold109973.2	1.94777	6.01315	-1.6263	0.00271512	--
evm.model.scaffold128461.1	2.16446	4.07844	-0.91401	0.00755259	sp|Q99M80|PTPRT_MOUSE Receptor-type tyrosine-protein phosphatase T OS=Mus musculus GN=Ptprt PE=2 SV=2

evm.model.scaffold104929.19	2.38171	6.43892	-1.43482	0.000820679	sp|P21525|FOSLA_DROME Transcription factor kayak, isoforms A/B/F OS=Drosophila melanogaster GN=kay PE=1 SV=4
evm.model.scaffold168485.6	3.38388	6.53352	-0.949181	0.0101381	sp|Q3UDW8|HGNAT_MOUSE Heparan-alpha-glucosaminide N-acetyltransferase OS=Mus musculus GN=Hgsnat PE=1 SV=2
evm.model.scaffold88753.15.1	23.5929	36.8572	-0.643593	0.027885	sp|Q32PX7|FUBP1_RAT Far upstream element-binding protein 1 OS=Rattus norvegicus GN=Fubp1 PE=1 SV=1
evm.model.scaffold170495.15	10.5213	16.6305	-0.660518	0.0231466	sp|P08953|TOLL_DROME Protein toll OS=Drosophila melanogaster GN=Tl PE=1 SV=1
evm.model.scaffold170495.12	4.5502	12.4573	-1.45299	0.000820679	sp|P08953|TOLL_DROME Protein toll OS=Drosophila melanogaster GN=Tl PE=1 SV=1
evm.model.scaffold30179.4	1.57074	3.46085	-1.13968	0.0105331	sp|Q28927|ADRB1_SHEEP Beta-1 adrenergic receptor OS=Ovis aries GN=ADRB1 PE=2 SV=2
evm.model.scaffold9813.34.1	1.6369	11.5294	-2.81628	0.000820679	--
evm.model.scaffold26355.2	9.92605	26.9118	-1.43895	0.000820679	--
evm.model.scaffold90899.19	8.92541	14.5027	-0.700329	0.0159979	sp|P08928|LAM0_DROME Lamin Dm0 OS=Drosophila melanogaster GN=Lam PE=1 SV=4

evm.model.scaffold140847.17	49.648	87.3044	-0.814318	0.00213042	sp|Q2KIT4|DNJB4_BOVIN DnaJ homolog subfamily B member 4 OS=Bos taurus GN=DNAJB4 PE=2 SV=1
evm.model.scaffold146755.7	1.48067	3.27838	-1.14673	0.0218619	--
evm.model.scaffold4129.6	1.1094	2.29599	-1.04934	0.0191834	sp|P23385|GRM1_RAT Metabotropic glutamate receptor 1 OS=Rattus norvegicus GN=Grm1 PE=1 SV=1
evm.model.scaffold68793.1	17.086	28.3874	-0.732433	0.0152063	sp|Q9BRS8|LARP6_HUMAN La-related protein 6 OS=Homo sapiens GN=LARP6 PE=1 SV=1
evm.model.scaffold162457.8	3.73218	22.782	-2.60981	0.0276479	--
evm.model.scaffold29275.13	7.19351	13.6438	-0.923476	0.000820679	sp|A0JNA3|IMDH1_BOVIN Inosine-5'-monophosphate dehydrogenase 1 OS=Bos taurus GN=IMPDH1 PE=2 SV=2
evm.model.scaffold160305.2	8.16854	32.6314	-1.99811	0.000820679	sp|Q964E1|ACTC_BIOOB Actin, cytoplasmic OS=Biomphalaria obstructa PE=3 SV=1
evm.model.scaffold160305.3	23.1715	56.3029	-1.28086	0.000820679	sp|Q93131|ACTC_BRAFL Actin, cytoplasmic OS=Branchiostoma floridae PE=2 SV=1
evm.model.scaffold52321.73	2.03492	5.8975	-1.53513	0.000820679	sp|Q9EQD2|NPFF2_RAT Neuropeptide FF receptor 2 OS=Rattus norvegicus GN=Npffr2 PE=2 SV=1

evm.model.scaffold52321.72	28.0158	49.7517	-0.828504	0.0241881	sp|O95236|APOL3_HUMAN Apolipoprotein L3 OS=Homo sapiens GN=APOL3 PE=1 SV=3
evm.model.scaffold52321.75	0.774579	2.99382	-1.9505	0.00380231	sp|O95236|APOL3_HUMAN Apolipoprotein L3 OS=Homo sapiens GN=APOL3 PE=1 SV=3
evm.model.scaffold52321.74	0.641688	2.05201	-1.67709	0.0173453	--
evm.model.scaffold159927.22	1.02736	3.75444	-1.86966	0.00429787	sp|Q13951|PEBB_HUMAN Core-binding factor subunit beta OS=Homo sapiens GN=CBFB PE=1 SV=2
evm.model.scaffold107255.38	7.09379	11.8511	-0.740387	0.0494585	sp|Q49GP5|P4K2B_DANRE Phosphatidylinositol 4-kinase type 2-beta OS=Danio rerio GN=pi4k2b PE=2 SV=1
evm.model.scaffold107255.33	55.0959	125.544	-1.18818	0.000820679	sp|Q9VC61|CRERF_DROME Protein CREBRF homolog OS=Drosophila melanogaster GN=CG13624 PE=2 SV=2
evm.model.scaffold7521.5	2.13524	5.81693	-1.44586	0.00151774	sp|P48039|MTR1A_HUMAN Melatonin receptor type 1A OS=Homo sapiens GN=MTNR1A PE=1 SV=1
evm.model.scaffold126727.31	0.11627	6.29092	-5.75772	0.000820679	sp|P29228|VLPA_MYCHR Variant surface antigen A OS=Mycoplasma hyorhinis GN=vlpA PE=4 SV=2
evm.model.scaffold154529.43	14.8486	37.2403	-1.32653	0.000820679	sp|Q91766|HNF4A_XENLA

					Hepatocyte nuclear factor 4-alpha OS=Xenopus laevis GN=hnf4a PE=2 SV=2
evm.model.scaffold154529.44	2.29076	5.0156	-1.13059	0.0451727	--
evm.model.scaffold124071.3	6.58559	11.0173	-0.742391	0.0476583	sp|Q5ZKI4|CCD93_CHICK Coiled-coil domain-containing protein 93 OS=Gallus gallus GN=CCDC93 PE=2 SV=1
evm.model.scaffold154027.23	137.62	246.532	-0.841083	0.00380231	--
evm.model.scaffold138629.43	33.0085	176.231	-2.41656	0.000820679	sp|Q8HXX6|SAP3_MACFA Ganglioside GM2 activator OS=Macaca fascicularis GN=GM2A PE=2 SV=2
evm.model.scaffold136919.1	10.6006	29.8536	-1.49376	0.000820679	sp|Q4FZY0|EFHD2_RAT EF-hand domain-containing protein D2 OS=Rattus norvegicus GN=Efhd2 PE=1 SV=1
evm.model.scaffold71989.28	5.24854	9.22247	-0.813236	0.0208422	--
evm.model.scaffold42227.10	20.5203	34.4324	-0.746711	0.00972356	sp|Q9VR07|INE_DROME Sodium- and chloride-dependent GABA transporter ine OS=Drosophila melanogaster GN=ine PE=1 SV=1
evm.model.scaffold72185.19	3.03205	5.11735	-0.755102	0.0305208	sp|Q9NVA4|T184C_HUMAN Transmembrane protein 184C OS=Homo sapiens GN=TMEM184C PE=2 SV=2

evm.model.scaffold16445.3	3.85336	12.7231	-1.72326	0.000820679	sp|Q6DHN0|TMM53_DANRE Transmembrane protein 53 OS=Danio rerio GN=tmem53 PE=2 SV=1
evm.model.scaffold35045.1	9.36722	17.5829	-0.908479	0.0137473	sp|P58058|NADK_MOUSE NAD kinase OS=Mus musculus GN=Nadk PE=1 SV=2
evm.model.scaffold66475.21	0	1.1131	#NAME?	0.000820679	--
evm.model.scaffold66475.20	0.420766	2.23688	-2.4104	0.0177206	--
evm.model.scaffold58523.1	0.831648	3.47296	-2.06212	0.00151774	--
evm.model.scaffold93729.11	15.8802	43.0333	-1.43822	0.000820679	sp|D9HP27|CNR11_MAIZE Cell number regulator 11 OS=Zea mays GN=CNR11 PE=2 SV=1
evm.model.scaffold33543.3	2.2399	6.66246	-1.57262	0.000820679	sp|Q9DD78|TLR21_CHICK Toll-like receptor 2 type-1 OS=Gallus gallus GN=TLR2-1 PE=2 SV=1
evm.model.scaffold26189.34	5.98249	12.9842	-1.11794	0.000820679	--
evm.model.scaffold127065.4	15.7589	26.7064	-0.76102	0.0156116	sp|Q2WGK2|JAM1_FELCA Junctional adhesion molecule A OS=Felis catus GN=F11R PE=1 SV=1
evm.model.scaffold122901.4	11.6747	38.6543	-1.72725	0.000820679	sp|O43934|MFS11_HUMAN UNC93-like protein MFSD11 OS=Homo sapiens GN=MFSD11 PE=2 SV=2
evm.model.scaffold149573.12	14.2533	22.4274	-0.653973	0.0460364	sp|Q9V3H2|PSDE_DROME 26S proteasome non-ATPase regulatory

					subunit 14 OS=Drosophila melanogaster GN=Rpn11 PE=1 SV=1
evm.model.scaffold98335.59	22.7838	98.5385	-2.11268	0.0368081	--
evm.model.scaffold170995.61.1	4.16974	8.51134	-1.02943	0.00151774	sp|Q9NFT7|HXK2_DROME Hexokinase type 2 OS=Drosophila melanogaster GN=Hex-t2 PE=2 SV=4
evm.model.scaffold3649.1	0.707645	4.3849	-2.63145	0.000820679	--
evm.model.scaffold175229.10	0.397112	1.40674	-1.82474	0.00429787	sp|P17713|STK_HYDVU Tyrosine-protein kinase STK OS=Hydra vulgaris GN=STK PE=2 SV=1
evm.model.scaffold141451.2	2.25279	6.39886	-1.5061	0.000820679	sp|E1C3S7|TDRD7_CHICK Tudor domain-containing protein 7 OS=Gallus gallus GN=TDRD7 PE=2 SV=1
evm.model.scaffold141451.1	5.26922	11.6591	-1.1458	0.00380231	sp|Q28808|IFI6_PANTR Interferon alpha-inducible protein 6 OS=Pan troglodytes GN=IFI6 PE=2 SV=1
evm.model.scaffold142869.25	0.507855	1.56923	-1.62757	0.0479762	--
evm.model.scaffold51533.21	8.41197	17.3052	-1.04069	0.000820679	sp|Q91ZR3|ZHANG_MOUSE CREB/ATF bZIP transcription factor OS=Mus musculus GN=Crebzf PE=1 SV=2
evm.model.scaffold132315.29	3.78799	6.74941	-0.83333	0.00578348	sp|Q5F478|ANR44_CHICK Serine/threonine-protein phosphatase

					6 regulatory ankyrin repeat subunit B OS=Gallus gallus GN=ANKRD44 PE=2 SV=1
evm.model.scaffold166837.35	2.89289	8.4593	-1.54803	0.000820679	--
evm.model.scaffold145793.13	12.4804	37.1933	-1.57537	0.000820679	sp|Q96RW7|HMCN1_HUMAN Hemicentin-1 OS=Homo sapiens GN=HMCN1 PE=1 SV=2
evm.model.scaffold65307.25	74.0573	152.835	-1.04526	0.000820679	--
evm.model.scaffold65307.28	3.7833	8.0334	-1.08637	0.000820679	sp|Q9Z148|EHMT2_MOUSE Histone-lysine N-methyltransferase EHMT2 OS=Mus musculus GN=Ehmt2 PE=1 SV=2
evm.model.scaffold43975.4.1	101.63	304.265	-1.582	0.000820679	sp|Q03168|ASPP_AEDAE Lysosomal aspartic protease OS=Aedes aegypti GN=AAEL006169 PE=1 SV=2
evm.model.scaffold148433.3	1.82232	16.1698	-3.14945	0.000820679	sp|Q70LM4|LGRD_BREPA Linear gramicidin synthase subunit D OS=Brevibacillus parabrevis GN=lgrD PE=1 SV=1
evm.model.scaffold104023.20	0.573561	1.70391	-1.57083	0.016685	sp|Q8JHV9|BIR7A_XENLA Baculoviral IAP repeat-containing protein 7-A OS=Xenopus laevis GN=birc7-a PE=1 SV=1
evm.model.scaffold109409.1	1.63879	3.23072	-0.979227	0.0264598	sp|Q6R5N8|TLR13_MOUSE Toll-like receptor 13 OS=Mus musculus

					GN=Tlr13 PE=1 SV=1
evm.model.scaffold2735.17	9.03055	23.2232	-1.36268	0.000820679	--
evm.model.scaffold73857.4	10.1295	31.8523	-1.65284	0.000820679	sp|Q8CHN6|SGPL1_RAT Sphingosine-1-phosphate lyase 1 OS=Rattus norvegicus GN=Sgpl1 PE=2 SV=1
evm.model.scaffold144571.12	0.404415	1.9088	-2.23876	0.00800162	sp|P28039|AOAH_HUMAN Acyloxyacyl hydrolase OS=Homo sapiens GN=AOAH PE=1 SV=1
evm.model.scaffold9813.38	0.668438	3.0577	-2.19358	0.000820679	sp|O01393|UNC9_CAEEL Innexin unc-9 OS=Caenorhabditis elegans GN=unc-9 PE=2 SV=1
evm.model.scaffold9813.36	15.0306	61.4639	-2.03184	0.000820679	sp|O01393|UNC9_CAEEL Innexin unc-9 OS=Caenorhabditis elegans GN=unc-9 PE=2 SV=1
evm.model.scaffold9813.30	8.5083	25.409	-1.5784	0.000820679	sp|O01393|UNC9_CAEEL Innexin unc-9 OS=Caenorhabditis elegans GN=unc-9 PE=2 SV=1
evm.model.scaffold9813.31	1.09385	4.24746	-1.95719	0.000820679	sp|O01393|UNC9_CAEEL Innexin unc-9 OS=Caenorhabditis elegans GN=unc-9 PE=2 SV=1
evm.model.scaffold168231.28	1.61186	3.39554	-1.07491	0.0411246	sp|Q9NUV9|GIMA4_HUMAN GTPase IMAP family member 4 OS=Homo sapiens GN=GIMAP4 PE=1 SV=1

evm.model.scaffold168231.26	5.77251	13.5983	-1.23615	0.00213042	sp|Q9NUV9|GIMA4_HUMAN GTPase IMAP family member 4 OS=Homo sapiens GN=GIMAP4 PE=1 SV=1
evm.model.scaffold152525.9	3.77743	8.28967	-1.13391	0.000820679	sp|A2T737|EHF_PANTR ETS homologous factor OS=Pan troglodytes GN=EHF PE=3 SV=1
evm.model.scaffold152525.8	3.0127	8.71304	-1.53212	0.000820679	sp|Q32LN0|EHF_BOVIN ETS homologous factor OS=Bos taurus GN=EHF PE=2 SV=1
evm.model.scaffold152525.7	0.371983	1.31721	-1.82417	0.00972356	sp|Q20930|MIG17_CAEEL ADAM family mig-17 OS=Caenorhabditis elegans GN=mig-17 PE=1 SV=3
evm.model.scaffold152525.6	0.564453	6.51407	-3.52863	0.000820679	sp|Q9UKP5|ATS6_HUMAN A disintegrin and metalloproteinase with thrombospondin motifs 6 OS=Homo sapiens GN=ADAMTS6 PE=2 SV=2
evm.model.scaffold59861.12	15.6773	26.5913	-0.762274	0.00842602	sp|P74897|YQA3_THEAQ Universal stress protein in QAH/OAS sulfhydrylase 3'region OS=Thermus aquaticus PE=3 SV=1
evm.model.scaffold171195.6	0.997009	3.43125	-1.78306	0.00429787	sp|Q8K2J9|BTBD6_MOUSE BTB/POZ domain-containing protein 6 OS=Mus musculus GN=Btbd6 PE=2 SV=2
evm.model.scaffold98447.46	3.40023	5.44384	-0.678993	0.0457321	sp|Q3U0J8|TBD2B_MOUSE TBC1

					domain family member 2B OS=Mus musculus GN=Tbc1d2b PE=1 SV=2
evm.model.scaffold121145.34	6.88563	20.6073	-1.5815	0.000820679	sp|Q8VEE0|RPE_MOUSE Ribulose-phosphate 3-epimerase OS=Mus musculus GN=Rpe PE=2 SV=1
evm.model.scaffold96251.2	8.68788	14.1431	-0.703024	0.0129481	sp|Q505D1|ANR28_MOUSE Serine/threonine-protein phosphatase 6 regulatory ankyrin repeat subunit A OS=Mus musculus GN=Ankrd28 PE=1 SV=1
evm.model.scaffold167531.1	104.285	256.847	-1.30038	0.000820679	sp|P82596|PLC_HALLA Perlucin OS=Haliotis laevigata PE=1 SV=3
evm.model.scaffold168171.11	0.770703	2.0227	-1.39204	0.0276479	sp|Q8BGC3|MOT12_MOUSE Monocarboxylate transporter 12 OS=Mus musculus GN=Slc16a12 PE=2 SV=1
evm.model.scaffold168171.12	0.293524	4.83903	-4.04317	0.000820679	sp|Q8R0M8|MOT5_MOUSE Monocarboxylate transporter 5 OS=Mus musculus GN=Slc16a4 PE=2 SV=1
evm.model.scaffold160093.2	1.75684	4.55747	-1.37525	0.00271512	sp|Q8TDB6|DTX3L_HUMAN E3 ubiquitin-protein ligase DTX3L OS=Homo sapiens GN=DTX3L PE=1 SV=1
evm.model.scaffold139475.12	0.803811	2.94927	-1.87543	0.000820679	sp|Q0PV50|TLR3_BOSTR Toll-like

					receptor 3 OS=Boselaphus tragocamelus GN=TLR3 PE=2 SV=1
evm.model.scaffold29081.2	10.5333	17.6378	-0.743711	0.0231466	sp|Q8R2E9|ERO1B_MOUSE ERO1-like protein beta OS=Mus musculus GN=Ero1lb PE=1 SV=1
evm.model.scaffold51225.2	5.49974	17.3849	-1.6604	0.000820679	sp|Q6IMZ0|NFIL3_RAT Nuclear factor interleukin-3-regulated protein OS=Rattus norvegicus GN=Nfil3 PE=2 SV=1
evm.model.scaffold172049.6	0.789275	50.223	-5.99168	0.000820679	sp|P82595|PLS_HALLA Perlustrin OS=Haliotis laevigata PE=1 SV=2
evm.model.scaffold172049.7	5.10883	9.85277	-0.947537	0.00380231	sp|Q6R5N8|TLR13_MOUSE Toll-like receptor 13 OS=Mus musculus GN=Tlr13 PE=1 SV=1
evm.model.scaffold168725.9	2.11604	4.61412	-1.12469	0.00151774	sp|Q5RE52|APC5_PONAB Anaphase-promoting complex subunit 5 OS=Pongo abelii GN=ANAPC5 PE=2 SV=1
evm.model.scaffold95039.14	9.2775	16.5259	-0.832917	0.0191834	sp|Q6DV14|PRDX1_GECJA Peroxiredoxin-1 OS=Gecko japonicus GN=PRDX1 PE=2 SV=1
evm.model.scaffold59345.18	7.31866	13.5038	-0.883714	0.00213042	sp|P0CE10|Y4102_ARATH Putative uncharacterized protein At4g01020, chloroplastic OS=Arabidopsis thaliana GN=At4g01020 PE=3 SV=1

evm.model.scaffold76375.1	4.51349	11.3966	-1.33629	0.00380231	sp|Q9ET66|PI16_MOUSE Peptidase inhibitor 16 OS=Mus musculus GN=Pi16 PE=2 SV=1
evm.model.scaffold37487.5	5.33168	26.0017	-2.28595	0.000820679	sp|O88279|SLIT1_RAT Slit homolog 1 protein OS=Rattus norvegicus GN=Slit1 PE=1 SV=1
evm.model.scaffold161075.4	16.1558	38.5472	-1.25457	0.000820679	sp|Q96GP6|SREC2_HUMAN Scavenger receptor class F member 2 OS=Homo sapiens GN=SCARF2 PE=1 SV=4
evm.model.scaffold161075.3	1.09134	3.83226	-1.81209	0.00213042	sp|F1QBY1|NIPLB_DANRE Nipped-B-like protein B OS=Danio rerio GN=nipblb PE=2 SV=1
evm.model.scaffold110569.12	10.6136	24.107	-1.18354	0.000820679	sp|O88281|MEGF6_RAT Multiple epidermal growth factor-like domains protein 6 OS=Rattus norvegicus GN=Megf6 PE=1 SV=1
evm.model.scaffold110569.18	8.85466	23.8071	-1.42689	0.000820679	--
evm.model.scaffold5015.10_evm.model.scaffold5015.14_evm.model.scaffold5015.15	3.19775	5.99182	-0.905937	0.0482225	sp|Q13263|TIF1B_HUMAN Transcription intermediary factor 1-beta OS=Homo sapiens GN=TRIM28 PE=1 SV=5
evm.model.scaffold103395.59	4.02265	11.688	-1.53881	0.035577	--
evm.model.scaffold30547.5	45.2217	98.7602	-1.12691	0.000820679	sp|Q5FVR2|TYPH_RAT Thymidine phosphorylase OS=Rattus norvegicus

					GN=Tymp PE=2 SV=1
evm.model.scaffold53139.17	10.9132	16.8579	-0.627347	0.0444534	sp|P79145|CREM_CANFA cAMP-responsive element modulator OS=Canis familiaris GN=CREM PE=2 SV=2
evm.model.scaffold123037.7	6.99493	17.0195	-1.28281	0.000820679	sp|Q8R4V0|SGK3_RAT Serine/threonine-protein kinase Sgk3 OS=Rattus norvegicus GN=Sgk3 PE=1 SV=2
evm.model.scaffold123037.4	2.82827	7.3256	-1.37303	0.00213042	--
evm.model.scaffold88257.23	21.0981	87.8941	-2.05865	0.000820679	sp|Q9H4G4|GAPR1_HUMAN Golgi-associated plant pathogenesis-related protein 1 OS=Homo sapiens GN=GLIPR2 PE=1 SV=3
evm.model.scaffold174791.17	0.321297	0.961683	-1.58165	0.0287519	sp|D2GXS7|TRIM2_AILME Tripartite motif-containing protein 2 OS=Ailuropoda melanoleuca GN=TRIM2 PE=3 SV=1
evm.model.scaffold79203.4	19.3113	33.8138	-0.808166	0.00380231	sp|Q8HXX6|SAP3_MACFA Ganglioside GM2 activator OS=Macaca fascicularis GN=GM2A PE=2 SV=2
evm.model.scaffold171147.1	8.3087	18.1077	-1.12391	0.0129481	sp|P38571|LICH_HUMAN Lysosomal acid lipase/cholesteryl ester hydrolase OS=Homo sapiens GN=LIPA PE=1

					SV=2
evm.model.scaffold109263.42	2.7436	5.93838	-1.114	0.00800162	sp|P27465|PISD_CRIGR Phosphatidylserine decarboxylase proenzyme OS=Cricetulus griseus GN=PISD PE=1 SV=2
evm.model.scaffold107255.51	2.66169	5.88793	-1.14542	0.00800162	--
evm.model.scaffold26071.56	1.43949	4.49229	-1.64189	0.0257834	--
evm.model.scaffold37199.9	2.33176	4.9903	-1.09771	0.0105331	sp|Q24307|DIAP2_DROME Death-associated inhibitor of apoptosis 2 OS=Drosophila melanogaster GN=Diap2 PE=1 SV=3
evm.model.scaffold146097.16	8.70063	17.5803	-1.01476	0.000820679	sp|P56941|NPC1_PIG Niemann-Pick C1 protein OS=Sus scrofa GN=NPC1 PE=2 SV=1
evm.model.scaffold176307.27	1.61687	3.0438	-0.912674	0.0491986	--
evm.model.scaffold127921.31	3.29782	10.6909	-1.6968	0.000820679	sp|Q9JM83|CALM4_MOUSE Calmodulin-4 OS=Mus musculus GN=Calm4 PE=2 SV=2
evm.model.scaffold175113.16	2.82078	4.96512	-0.815733	0.00888556	sp|F1QBY1|NIPLB_DANRE Nipped-B-like protein B OS=Danio rerio GN=nipblb PE=2 SV=1
evm.model.scaffold175113.15	2.21518	4.51622	-1.02769	0.00151774	sp|Q9UK61|F208A_HUMAN Protein FAM208A OS=Homo sapiens GN=FAM208A PE=1 SV=3
evm.model.scaffold5015.9	24.6848	50.2205	-1.02465	0.000820679	sp|Q5R668|ACSL3_PONAB

					Long-chain-fatty-acid--CoA ligase 3 OS=Pongo abelii GN=ACSL3 PE=2 SV=2
evm.model.scaffold43539.12	30.1609	47.1877	-0.645732	0.0241881	sp|P49419|AL7A1_HUMAN Alpha-aminoadipic semialdehyde dehydrogenase OS=Homo sapiens GN=ALDH7A1 PE=1 SV=5
evm.model.scaffold102061.3	4.81409	8.97846	-0.899204	0.0482225	sp|Q5TCZ1|SPD2A_HUMAN SH3 and PX domain-containing protein 2A OS=Homo sapiens GN=SH3PXD2A PE=1 SV=1
evm.model.scaffold27307.18	0.0355016	0.4776	-3.74985	0.000820679	sp|Q8WXR4|MYO3B_HUMAN Myosin-IIIb OS=Homo sapiens GN=MYO3B PE=2 SV=4
evm.model.scaffold172313.11	13.1253	37.7693	-1.52487	0.000820679	sp|Q58A42|DD3_DICDI Protein DD3-3 OS=Dictyostelium discoideum GN=DD3-3 PE=2 SV=1
evm.model.scaffold51453.60	1.75579	5.37292	-1.61359	0.00578348	sp|Q70FG7|DODA_BETVU 4,5-DOPA dioxygenase extradiol OS=Beta vulgaris GN=DODA PE=1 SV=1
evm.model.scaffold51453.63	7.44103	20.5069	-1.46253	0.000820679	sp|Q865F1|MTP_PIG Microsomal triglyceride transfer protein large subunit OS=Sus scrofa GN=MTTP PE=2 SV=1
evm.model.scaffold150821.27	5.65909	12.8525	-1.18341	0.000820679	sp|O96785|HUNB_CLOAL Protein

					hunchback (Fragment) OS=Clogmia albipunctata GN=hb PE=2 SV=1
evm.model.scaffold169545.5	1.38893	4.49244	-1.69352	0.0404844	sp|Q4AEE3|DNAS1_HORSE Deoxyribonuclease-1 OS=Equus caballus GN=DNASE1 PE=2 SV=1
evm.model.scaffold931.12	1.08874	3.04199	-1.48235	0.00933293	sp|Q7ZWG6|PCFT_DANRE Proton-coupled folate transporter OS=Danio rerio GN=slc46a1 PE=2 SV=1
evm.model.scaffold112779.6	6.27274	12.2649	-0.967365	0.0205228	--
evm.model.scaffold112779.5	4.7184	11.1994	-1.24706	0.0241881	--
evm.model.scaffold15567.21	11.4895	33.1392	-1.52823	0.000820679	sp|P11707|CP3A6_RABIT Cytochrome P450 3A6 OS=Oryctolagus cuniculus GN=CYP3A6 PE=2 SV=2
evm.model.scaffold168217.4	18.6638	30.8995	-0.727339	0.0109379	sp|P97384|ANX11_MOUSE Annexin A11 OS=Mus musculus GN=Anxa11 PE=2 SV=2
evm.model.scaffold51139.3	1.29153	3.6332	-1.49216	0.00213042	--
evm.model.scaffold90415.3	10.3703	16.9949	-0.712647	0.0180929	sp|Q92J02|GLRX1_RICCN Glutaredoxin-1 OS=Rickettsia conorii (strain ATCC VR-613 / Malish 7) GN=grxC1 PE=3 SV=1
evm.model.scaffold23177.8	2.73662	5.89288	-1.10658	0.00213042	--
evm.model.scaffold67471.1	2.11998	6.22697	-1.55448	0.000820679	sp|Q54KZ0|TM2D3_DICDI TM2 domain-containing protein DDB_G0287015 OS=Dictyostelium

					discoideum GN=DDB_G0287015 PE=3 SV=1
evm.model.scaffold122901.19	2.12312	5.51973	-1.37841	0.000820679	sp|Q9Z2M7|PMM2_MOUSE Phosphomannomutase 2 OS=Mus musculus GN=Pmm2 PE=1 SV=1
evm.model.scaffold119793.4	0	1.26741	#NAME?	0.000820679	sp|P86731|UP1_HALAI Uncharacterized protein 1 OS=Haliotis asinina PE=1 SV=1
evm.model.scaffold130831.7	8.38701	27.6356	-1.7203	0.000820679	--
evm.model.scaffold65971.60	0.990179	2.02457	-1.03186	0.0101381	sp|A6BM72|MEG11_HUMAN Multiple epidermal growth factor-like domains protein 11 OS=Homo sapiens GN=MEGF11 PE=2 SV=3
evm.model.scaffold62683.10	3.79259	6.87415	-0.857998	0.00712329	sp|B1H1P9|PPAL_XENLA Lysosomal acid phosphatase OS=Xenopus laevis GN=acp2 PE=2 SV=1
evm.model.scaffold38775.7.1	2.79181	6.28462	-1.17063	0.00800162	sp|P13277|CYSP1_HOMAM Digestive cysteine proteinase 1 OS=Homarus americanus GN=LCP1 PE=1 SV=2
evm.model.scaffold104929.23	2.4566	6.4321	-1.38863	0.00326205	sp|P29176|FOSX_MSVFR Transforming protein v-Fos/v-Fox OS=FBR murine osteosarcoma virus GN=FOS-FOX PE=3 SV=1
evm.model.scaffold160813.39	8.84774	31.8648	-1.84858	0.0113727	--
evm.model.scaffold145793.23	2.83773	6.0854	-1.10061	0.00480132	sp|Q90218|GLRK_ANAPL Probable

					glutamate receptor OS=Anas platyrhynchos GN=KBP PE=2 SV=1
evm.model.scaffold27973.127	37.3696	66.8356	-0.83875	0.00271512	sp|P56470|LEG4_HUMAN Galectin-4 OS=Homo sapiens GN=LGALS4 PE=1 SV=1
evm.model.scaffold70561.35	6.81795	12.1924	-0.838567	0.0188471	sp|Q9UIU6|SIX4_HUMAN Homeobox protein SIX4 OS=Homo sapiens GN=SIX4 PE=1 SV=2
evm.model.scaffold70561.39	5.72358	20.0955	-1.81189	0.000820679	sp|Q91496|FOS_TETFL Proto-oncogene c-Fos OS=Tetraodon fluviatilis GN=fos PE=3 SV=1
evm.model.scaffold73429.20	2.02219	16.3639	-3.01652	0.000820679	sp|A6NK06|IRG1_HUMAN Cis-aconitate decarboxylase OS=Homo sapiens GN=IRG1 PE=1 SV=1
evm.model.scaffold10643.1	0.417389	1.634	-1.96895	0.020248	sp|P98160|PGBM_HUMAN Basement membrane-specific heparan sulfate proteoglycan core protein OS=Homo sapiens GN=HSPG2 PE=1 SV=4
evm.model.scaffold123407.38	11.3435	33.4841	-1.56161	0.000820679	--
evm.model.scaffold64247.15	1.51935	4.61669	-1.60341	0.00213042	sp|Q08BT9|P20D1_XENTR Probable carboxypeptidase PM20D1 OS=Xenopus tropicalis GN=pm20d1 PE=2 SV=1
evm.model.scaffold168117.27	4.08386	6.66382	-0.706416	0.044934	sp|Q13231|CHIT1_HUMAN Chitotriosidase-1 OS=Homo sapiens

					GN=CHIT1 PE=1 SV=1
evm.model.scaffold175591.3	11.8167	25.6519	-1.11823	0.0141107	--
evm.model.scaffold103525.28	1.51832	2.83096	-0.898821	0.0347943	sp|Q5U4T9|MBOA7_XENLA Lysophospholipid acyltransferase 7 OS=Xenopus laevis GN=mboat7 PE=2 SV=1
evm.model.scaffold174343.9	7.4299	18.1404	-1.28779	0.000820679	sp|Q5R432|CNDP2_PONAB Cytosolic non-specific dipeptidase OS=Pongo abelii GN=CNDP2 PE=2 SV=1
evm.model.scaffold135399.10	1.58314	3.38576	-1.09669	0.0109379	sp|Q04499|PROD_DROME Proline dehydrogenase 1, mitochondrial OS=Drosophila melanogaster GN=slgA PE=1 SV=2
evm.model.scaffold152681.18	0.101739	6.42253	-5.98019	0.000820679	sp|Q7Z5P9|MUC19_HUMAN Mucin-19 OS=Homo sapiens GN=MUC19 PE=1 SV=2
evm.model.scaffold52321.81	0.827581	5.57739	-2.75262	0.000820679	sp|Q5BIZ0|MFSD4_XENTR Major facilitator superfamily domain-containing protein 4 OS=Xenopus tropicalis GN=mfsd4 PE=2 SV=1
evm.model.scaffold3377.8	25.1786	43.8819	-0.80143	0.00213042	sp|P48809|RB27C_DROME Heterogeneous nuclear ribonucleoprotein 27C OS=Drosophila

					melanogaster GN=Hrb27C PE=1 SV=2
evm.model.scaffold170775.31	1.90915	4.48978	-1.23372	0.000820679	sp|P16157|ANK1_HUMAN Ankyrin-1 OS=Homo sapiens GN=ANK1 PE=1 SV=3
evm.model.scaffold162731.70	5.46041	35.5404	-2.70238	0.000820679	sp|Q90744|NAGAB_CHICK Alpha-N-acetylgalactosaminidase OS=Gallus gallus GN=NAGA PE=1 SV=1
evm.model.scaffold73857.11	11.594	22.1169	-0.931775	0.0152063	sp|Q9FMR5|VA714_ARATH Vesicle-associated membrane protein 714 OS=Arabidopsis thaliana GN=VAMP714 PE=1 SV=1
evm.model.scaffold109085.30	0.51508	1.64741	-1.67733	0.00532397	--
evm.model.scaffold111939.11	8.9312	14.7816	-0.726873	0.0420901	sp|P51538|CP3A9_RAT Cytochrome P450 3A9 OS=Rattus norvegicus GN=Cyp3a9 PE=2 SV=2
evm.model.scaffold136189.8	7.17202	12.7149	-0.826072	0.00888556	sp|P97538|RASM_RAT Ras-related protein M-Ras OS=Rattus norvegicus GN=Mras PE=1 SV=2
evm.model.scaffold138629.94	7.52628	14.1773	-0.913571	0.000820679	sp|Q4LDD4|ARAP1_MOUSE Arf-GAP with Rho-GAP domain, ANK repeat and PH domain-containing protein 1 OS=Mus musculus GN=Arap1 PE=2 SV=2
evm.model.scaffold157929.19.1	0.499681	1.98489	-1.98998	0.000820679	sp|Q01196|RUNX1_HUMAN

					Runt-related transcription factor 1 OS=Homo sapiens GN=RUNX1 PE=1 SV=3
evm.model.scaffold13361.24	10.1539	24.4441	-1.26746	0.000820679	sp|Q9R0M6|RAB9A_MOUSE Ras-related protein Rab-9A OS=Mus musculus GN=Rab9a PE=1 SV=1
evm.model.scaffold171689.32	10.3084	19.4361	-0.914919	0.000820679	sp|Q92542|NICA_HUMAN Nicastrin OS=Homo sapiens GN=NCSTN PE=1 SV=2
evm.model.scaffold176119.50	5.90381	9.14923	-0.632004	0.0335734	sp|E9Q555|RN213_MOUSE E3 ubiquitin-protein ligase RNF213 OS=Mus musculus GN=Rnf213 PE=2 SV=1
evm.model.scaffold142051.7	0.66018	2.58567	-1.96961	0.00326205	sp|Q8JFG3|TNFA_SPAAU Tumor necrosis factor OS=Sparus aurata GN=tnf PE=3 SV=1
evm.model.scaffold136555.3	330.506	1102.77	-1.73838	0.000820679	sp|P54611|VATE_DROME V-type proton ATPase subunit E OS=Drosophila melanogaster GN=Vha26 PE=2 SV=1
evm.model.scaffold61991.49	3.07816	5.65877	-0.878419	0.00621905	sp|Q9UGC6|RGS17_HUMAN Regulator of G-protein signaling 17 OS=Homo sapiens GN=RGS17 PE=1 SV=2
evm.model.scaffold171681.3	5.0707	13.6594	-1.42964	0.000820679	--

evm.model.scaffold176027.43	6.08195	11.5389	-0.923902	0.0148208	--
evm.model.scaffold110817.26	14.0553	25.7149	-0.871488	0.00621905	sp|P07686|HEXB_HUMAN Beta-hexosaminidase subunit beta OS=Homo sapiens GN=HEXB PE=1 SV=3
evm.model.scaffold126679.1	7.11779	13.9807	-0.973938	0.0191834	sp|P86789|GIGA6_CRAGI Gigasin-6 OS=Crassostrea gigas PE=1 SV=1
evm.model.scaffold141451.45	2.07605	3.7338	-0.846805	0.0460364	sp|B6RSP1|PKHA7_DANRE Pleckstrin homology domain-containing family A member 7 OS=Danio rerio GN=plekha7 PE=2 SV=2
evm.model.scaffold67769.24	0.248434	0.598784	-1.26917	0.0347943	sp|Q9HBA0|TRPV4_HUMAN Transient receptor potential cation channel subfamily V member 4 OS=Homo sapiens GN=TRPV4 PE=1 SV=2
evm.model.scaffold142051.18	9.18348	21.461	-1.2246	0.000820679	sp|Q8JFG3|TNFA_SPAAU Tumor necrosis factor OS=Sparus aurata GN=tnf PE=3 SV=1
evm.model.scaffold15515.13	20.5969	38.6568	-0.908296	0.000820679	sp|P42577|FRIS_LYMST Soma ferritin OS=Lymnaea stagnalis PE=2 SV=2
evm.model.scaffold50579.16	2.04926	3.79601	-0.889383	0.0388548	sp|Q14534|ERG1_HUMAN Squalene monooxygenase OS=Homo sapiens GN=SQLE PE=1 SV=3

evm.model.scaffold7483.14_evm.model.scaffold7483.15	2.59059	5.27211	-1.0251	0.00380231	sp|Q8BZ20|PAR12_MOUSE Poly [ADP-ribose] polymerase 12 OS=Mus musculus GN=Parp12 PE=2 SV=3
evm.model.scaffold106529.17	9.02949	15.8592	-0.812603	0.00888556	sp|O70469|DOK2_MOUSE Docking protein 2 OS=Mus musculus GN=Dok2 PE=1 SV=1
evm.model.scaffold171673.33	13.9623	21.6783	-0.634709	0.0361439	sp|Q80U96|XPO1_RAT Exportin-1 OS=Rattus norvegicus GN=Xpo1 PE=1 SV=1
evm.model.scaffold76817.2	3.47792	12.3316	-1.82606	0.000820679	sp|Q9R0Q9|MPU1_MOUSE Mannose-P-dolichol utilization defect 1 protein OS=Mus musculus GN=Mpdu1 PE=2 SV=1
evm.model.scaffold85829.2	7.71241	15.4132	-0.998911	0.000820679	--
evm.model.scaffold73127.18	26.9823	45.0449	-0.739352	0.00326205	sp|P41239|CSK_CHICK Tyrosine-protein kinase CSK OS=Gallus gallus GN=CSK PE=2 SV=1
evm.model.scaffold140451.22	3.64318	7.91222	-1.11889	0.000820679	sp|Q28DL4|TRAF6_XENTR TNF receptor-associated factor 6 OS=Xenopus tropicalis GN=traf6 PE=2 SV=1
evm.model.scaffold98447.5	2.5061	4.74558	-0.921139	0.0188471	sp|Q8VCN5|CGL_MOUSE Cystathionine gamma-lyase OS=Mus musculus GN=Cth PE=1 SV=1
evm.model.scaffold99537.22	2.03265	15.0489	-2.88822	0.000820679	sp|P23174|MDR3_CRIGR Multidrug

					resistance protein 3 OS=Cricetulus griseus GN=ABCB4 PE=2 SV=1
evm.model.scaffold57357.12	0.0742504	0.83209	-3.48627	0.048902	sp|B3A0S3|ELDP2_LOTGI EGF-like domain-containing protein 2 OS=Lottia gigantea PE=1 SV=1
evm.model.scaffold57357.11	1.03693	3.27165	-1.65769	0.000820679	--
evm.model.scaffold72861.10	14.9747	36.3631	-1.27995	0.000820679	sp|Q9JM83|CALM4_MOUSE Calmodulin-4 OS=Mus musculus GN=Calm4 PE=2 SV=2
evm.model.scaffold176621.52	9.70564	24.6085	-1.34226	0.00151774	sp|A4IF78|TFIP8_BOVIN Tumor necrosis factor alpha-induced protein 8 OS=Bos taurus GN=TNFAIP8 PE=2 SV=1
evm.model.scaffold128139.87	3.6188	8.0268	-1.14931	0.00151774	sp|Q8WV28|BLNK_HUMAN B-cell linker protein OS=Homo sapiens GN=BLNK PE=1 SV=2
evm.model.scaffold50375.10_evm.model.scaffold50375.9	11.9704	25.1103	-1.06881	0.000820679	--
evm.model.scaffold50579.8	0.954929	4.77851	-2.3231	0.000820679	sp|Q3T0J1|FBX4_BOVIN F-box only protein 4 OS=Bos taurus GN=FBXO4 PE=2 SV=1
evm.model.scaffold135929.32	4.33376	13.6212	-1.65216	0.000820679	--
evm.model.scaffold135929.34	0.912433	2.51077	-1.46034	0.027885	--
evm.model.scaffold135929.37	11.4407	32.8597	-1.52215	0.000820679	--
evm.model.scaffold135929.38	1.32828	3.14352	-1.24282	0.0318917	sp|Q26474|LACH_SCHAM Lachesin

					OS=Schistocerca americana GN=LAC PE=1 SV=1
evm.model.scaffold99441.15	0	1.45436	#NAME?	0.0211683	sp|Q57997|Y577_METJA Universal stress protein MJ0577 OS=Methanocaldococcus jannaschii (strain ATCC 43067 / DSM 2661 / JAL-1 / JCM 10045 / NBRC 100440) GN=MJ0577 PE=1 SV=1
evm.model.scaffold93729.57	4.90461	8.77683	-0.839564	0.0296885	sp|Q17103|MYC_ASTRU Myc protein (Fragment) OS=Asterias rubens GN=MYC PE=2 SV=1
evm.model.scaffold73677.6	6.13364	11.7687	-0.940134	0.00151774	sp|Q06852|SLAP1_CLOTH Cell surface glycoprotein 1 OS=Clostridium thermocellum (strain ATCC 27405 / DSM 1237 / NBRC 103400 / NCIMB 10682 / NRRL B-4536 / VPI 7372) GN=olpB PE=3 SV=2
evm.model.scaffold73677.1	8.08169	13.7626	-0.768025	0.029403	sp|Q2IBF2|MET_GORGO Hepatocyte growth factor receptor OS=Gorilla gorilla gorilla GN=MET PE=3 SV=1
evm.model.scaffold66661.51	17.6629	60.0594	-1.76567	0.000820679	sp|Q9R1T3|CATZ_RAT Cathepsin Z OS=Rattus norvegicus GN=Ctsz PE=1 SV=2
evm.model.scaffold69129.4	3.40976	9.60498	-1.49411	0.00213042	sp|Q58CV5|SPX2_BOVIN Sugar phosphate exchanger 2 OS=Bos taurus

					GN=SLC37A2 PE=2 SV=1
evm.model.scaffold69129.3	2.10674	4.62792	-1.13535	0.0276479	sp|Q7SY29|SPX2_DANRE Sugar phosphate exchanger 2 OS=Danio rerio GN=slc37a2 PE=2 SV=1
evm.model.scaffold69129.2	2.98531	8.12291	-1.44412	0.000820679	--
evm.model.scaffold78787.19	2.46408	9.434	-1.93682	0.000820679	sp|Q86WA9|S2611_HUMAN Sodium-independent sulfate anion transporter OS=Homo sapiens GN=SLC26A11 PE=2 SV=2
evm.model.scaffold175113.12	3.68978	9.62659	-1.38349	0.000820679	sp|Q6NSJ0|K1161_HUMAN Uncharacterized family 31 glucosidase KIAA1161 OS=Homo sapiens GN=KIAA1161 PE=1 SV=2
evm.model.scaffold132635.25	3.35945	6.29922	-0.906947	0.0471091	--
evm.model.scaffold132635.26	2.39181	12.3988	-2.37402	0.000820679	--
evm.model.scaffold132635.27	1.87085	5.74591	-1.61884	0.000820679	--
evm.model.scaffold21181.23_evm.model.scaffold21181.24	1.28433	3.93492	-1.61532	0.00933293	sp|Q5R792|ATG5_PONAB Autophagy protein 5 OS=Pongo abelii GN=ATG5 PE=2 SV=1
evm.model.scaffold53871.1	10.0514	16.0483	-0.675031	0.0311397	sp|Q09YN5|MET_RABIT Hepatocyte growth factor receptor OS=Oryctolagus cuniculus GN=MET PE=3 SV=1
evm.model.scaffold53871.2	1.10425	2.55088	-1.20792	0.0418385	sp|Q2QLH6|MET_OTOGA Hepatocyte growth factor receptor

					OS=Otolemur garnettii GN=MET PE=3 SV=1
evm.model.scaffold64345.3	1.98299	5.0606	-1.35163	0.000820679	sp|P97864|CASP7_MOUSE Caspase-7 OS=Mus musculus GN=Casp7 PE=1 SV=2
evm.model.scaffold51139.28	3.48361	38.5564	-3.46832	0.000820679	sp|O62640|PIAP_PIG Putative inhibitor of apoptosis OS=Sus scrofa GN=PIAP PE=2 SV=1
evm.model.scaffold123417.25	2.59404	5.46279	-1.07444	0.0141107	--
evm.model.scaffold66257.89	1.11068	2.33295	-1.07071	0.0218619	sp|Q9PWF7|CATA_GLARU Catalase OS=Glandirana rugosa GN=cat PE=2 SV=3
evm.model.scaffold823.36_evm.model.scaffold823.37	2.8132	9.80358	-1.8011	0.000820679	sp|Q8BFR4|GNS_MOUSE N-acetylglucosamine-6-sulfatase OS=Mus musculus GN=Gns PE=2 SV=1
evm.model.scaffold3293.12	0.948599	2.72268	-1.52116	0.00480132	sp|P00763|TRY2_RAT Anionic trypsin-2 OS=Rattus norvegicus GN=Prss2 PE=1 SV=2
evm.model.scaffold99537.20	1.28214	9.17559	-2.83924	0.000820679	sp|P08183|MDR1_HUMAN Multidrug resistance protein 1 OS=Homo sapiens GN=ABCB1 PE=1 SV=3
evm.model.scaffold154521.5	0	1.23334	#NAME?	0.00755259	sp|Q3U0Y2|ZMYNB_MOUSE Uncharacterized protein ZMYM6NB

					OS=Mus musculus GN=Zmym6nb PE=2 SV=2
evm.model.scaffold150821.76	3.39831	8.29495	-1.28741	0.00151774	--
evm.model.scaffold147663.4	1.24697	3.19746	-1.3585	0.014476	sp|Q924H0|NPFF2_MOUSE Neuropeptide FF receptor 2 OS=Mus musculus GN=Npffr2 PE=2 SV=2
evm.model.scaffold176361.97	4.5773	7.54669	-0.721349	0.0271297	sp|P18433|PTPRA_HUMAN Receptor-type tyrosine-protein phosphatase alpha OS=Homo sapiens GN=PTPRA PE=1 SV=2
evm.model.scaffold176361.98	2.22822	5.53281	-1.31212	0.00380231	sp|A0JM12|MEG10_XENTR Multiple epidermal growth factor-like domains protein 10 OS=Xenopus tropicalis GN=megf10 PE=2 SV=1
evm.model.scaffold109967.5	2.00487	6.54773	-1.70748	0.000820679	sp|A1L1W9|MOT10_DANRE Monocarboxylate transporter 10 OS=Danio rerio GN=slc16a10 PE=2 SV=1
evm.model.scaffold132179.27	1.06043	3.70701	-1.80561	0.000820679	sp|Q9Y6I7|WSB1_HUMAN WD repeat and SOCS box-containing protein 1 OS=Homo sapiens GN=WSB1 PE=1 SV=1
evm.model.scaffold132179.21	5.10109	13.2051	-1.37222	0.000820679	sp|Q3TCN2|PLBL2_MOUSE Putative phospholipase B-like 2 OS=Mus musculus GN=Plbd2 PE=1 SV=2

evm.model.scaffold174791.20	3.64752	50.5731	-3.79338	0.000820679	sp|P59222|SREC2_MOUSE Scavenger receptor class F member 2 OS=Mus musculus GN=Scarf2 PE=1 SV=1
evm.model.scaffold174791.27	2.48389	5.49291	-1.14497	0.00670086	sp|Q96GP6|SREC2_HUMAN Scavenger receptor class F member 2 OS=Homo sapiens GN=SCARF2 PE=1 SV=4
evm.model.scaffold174791.24	1.7857	10.0975	-2.49943	0.000820679	sp|Q80V70|MEGF6_MOUSE Multiple epidermal growth factor-like domains protein 6 OS=Mus musculus GN=Megf6 PE=2 SV=3
evm.model.scaffold67769.7	6.52458	15.9494	-1.28955	0.000820679	sp|Q6DFJ6|TBK1_XENLA Serine/threonine-protein kinase TBK1 OS=Xenopus laevis GN=tbk1 PE=2 SV=1
evm.model.scaffold67769.6	0.289356	0.802842	-1.47227	0.0494585	--
evm.model.scaffold109207.3	1.57027	10.8759	-2.79204	0.000820679	--
evm.model.scaffold39283.2	18.0121	49.4165	-1.45603	0.000820679	sp|Q26636|CATL_SARPE Cathepsin L OS=Sarcophaga peregrina PE=1 SV=1
evm.model.scaffold30711.17	1.14199	2.5724	-1.17157	0.0205228	sp|Q80V70|MEGF6_MOUSE Multiple epidermal growth factor-like domains protein 6 OS=Mus musculus GN=Megf6 PE=2 SV=3
evm.model.scaffold26349.3	2.28399	3.7191	-0.703397	0.0364617	sp|Q5ZKD7|MOV10_CHICK Putative helicase MOV-10 OS=Gallus gallus

					GN=MOV10 PE=2 SV=1
evm.model.scaffold99267.10	35.5827	93.8613	-1.39936	0.000820679	sp|P40423|SQH_DROME Myosin regulatory light chain sqh OS=Drosophila melanogaster GN=sqh PE=1 SV=1
evm.model.scaffold136165.23	0.797611	2.39254	-1.58479	0.0291155	sp|Q5ND28|SREC_MOUSE Scavenger receptor class F member 1 OS=Mus musculus GN=Scarf1 PE=1 SV=1
evm.model.scaffold146097.23	3.98211	6.78229	-0.76824	0.0159979	--
evm.model.scaffold115227.48	1.58973	5.04659	-1.66653	0.000820679	sp|Q14527|HLTF_HUMAN Helicase-like transcription factor OS=Homo sapiens GN=HLTF PE=1 SV=2
evm.model.scaffold171673.27	3.08718	6.11292	-0.985573	0.000820679	sp|Q9BX95|SGPP1_HUMAN Sphingosine-1-phosphate phosphatase 1 OS=Homo sapiens GN=SGPP1 PE=1 SV=2
evm.model.scaffold176307.15	0.825283	2.73954	-1.73097	0.000820679	sp|P40189|IL6RB_HUMAN Interleukin-6 receptor subunit beta OS=Homo sapiens GN=IL6ST PE=1 SV=2
evm.model.scaffold167793.1	10.5524	33.0258	-1.64602	0.000820679	sp|C3YWU0|FUCO_BRAFL Alpha-L-fucosidase OS=Branchiostoma floridae GN=BRAFLDRAFT_56888 PE=3 SV=2

evm.model.scaffold118787.26	8.11258	13.6245	-0.747973	0.0117709	--
evm.model.scaffold146755.14	10.5633	35.3567	-1.74292	0.000820679	--
evm.model.scaffold167793.71_evm.model.scaffold167793.70	1.90757	5.02657	-1.39784	0.000820679	sp|Q8IY33|MILK2_HUMAN MICAL-like protein 2 OS=Homo sapiens GN=MICALL2 PE=1 SV=1
evm.model.scaffold51533.17.1	49.163	78.3937	-0.673164	0.0215514	sp|Q5ZL67|NF2L1_CHICK Nuclear factor erythroid 2-related factor 1 OS=Gallus gallus GN=NFE2L1 PE=2 SV=1
evm.model.scaffold37069.2	0.225728	1.22922	-2.44508	0.0264598	sp|Q61469|LPP1_MOUSE Lipid phosphate phosphohydrolase 1 OS=Mus musculus GN=Ppap2a PE=1 SV=1
evm.model.scaffold19527.6	7.47113	15.0286	-1.00831	0.000820679	sp|Q58DJ0|IRF5_BOVIN Interferon regulatory factor 5 OS=Bos taurus GN=IRF5 PE=2 SV=1
evm.model.scaffold56755.119	0.764651	2.0467	-1.42043	0.0314536	--
evm.model.scaffold52659.1	7.33424	12.7726	-0.800333	0.00429787	sp|A1X150|MET_ECHTE Hepatocyte growth factor receptor OS=Echinops telfairi GN=MET PE=3 SV=1
evm.model.scaffold64565.44	8.39468	14.5793	-0.796375	0.00621905	sp|Q9Z207|DIAP3_MOUSE Protein diaphanous homolog 3 OS=Mus musculus GN=Diaph3 PE=1 SV=1
evm.model.scaffold169745.9.4	3.29421	6.59823	-1.00214	0.00326205	sp|P31327|CPSM_HUMAN Carbamoyl-phosphate synthase

					[ammonia], mitochondrial OS=Homo sapiens GN=CPS1 PE=1 SV=2
evm.model.scaffold72763.129	6.61821	12.7058	-0.940977	0.000820679	sp|Q8CHR6|DPYD_MOUSE Dihydropyrimidine dehydrogenase [NADP(+)] OS=Mus musculus GN=Dpyd PE=1 SV=1
evm.model.scaffold39835.1	4.11467	10.3595	-1.33211	0.0484321	--
evm.model.scaffold38207.10	37.8456	58.6485	-0.631969	0.032187	sp|Q8CFN2|CDC42_RAT Cell division control protein 42 homolog OS=Rattus norvegicus GN=Cdc42 PE=1 SV=2
evm.model.scaffold73429.19	0.665774	3.86337	-2.53676	0.000820679	sp|Q5U3U0|PHYD1_DANRE Phytanoyl-CoA dioxygenase domain-containing protein 1 OS=Danio rerio GN=phyhd1 PE=2 SV=1
evm.model.scaffold150821.79	1.91468	3.44177	-0.846046	0.0276479	sp|Q92543|SNX19_HUMAN Sorting nexin-19 OS=Homo sapiens GN=SNX19 PE=1 SV=2
evm.model.scaffold79079.3	27.6955	64.3355	-1.21596	0.000820679	sp|P59999|ARPC4_MOUSE Actin-related protein 2/3 complex subunit 4 OS=Mus musculus GN=Arpc4 PE=1 SV=3
evm.model.scaffold13425.5	0.498935	1.53141	-1.61794	0.00326205	sp|Q5NBX1|COBL_MOUSE Protein cordon-bleu OS=Mus musculus

					GN=Cobl PE=1 SV=1
evm.model.scaffold82395.4	1.52894	2.82096	-0.883658	0.029403	sp|Q9VT65|CANB_DROME Calpain-B OS=Drosophila melanogaster GN=CalpB PE=1 SV=2
evm.model.scaffold158265.30	1.17123	3.07482	-1.39248	0.00933293	sp|A0JM12|MEG10_XENTR Multiple epidermal growth factor-like domains protein 10 OS=Xenopus tropicalis GN=megf10 PE=2 SV=1
evm.model.scaffold164773.68	0.836274	3.94368	-2.2375	0.00842602	sp|Q66H59|NPL_RAT N-acetylneuraminate lyase OS=Rattus norvegicus GN=Npl PE=2 SV=1
evm.model.scaffold137529.3	9.88575	16.9965	-0.781814	0.0208422	sp|Q6INX1|TMM98_XENLA Transmembrane protein 98 OS=Xenopus laevis GN=tmem98 PE=2 SV=1
evm.model.scaffold68735.1	1.31987	3.01986	-1.19409	0.00670086	sp|Q6QNU9|TLR12_MOUSE Toll-like receptor 12 OS=Mus musculus GN=Tlr12 PE=2 SV=1
evm.model.scaffold104629.5	16.2979	25.3911	-0.639635	0.0271297	sp|Q7ZVZ7|AB17C_DANRE Alpha/beta hydrolase domain-containing protein 17C OS=Danio rerio GN=abhd17c PE=2 SV=1
evm.model.scaffold17291.1	5.25316	8.96437	-0.771018	0.0248731	sp|P28227|MAOX_ANAPL NADP-dependent malic enzyme

					OS=Anas platyrhynchos GN=ME1 PE=1 SV=1
evm.model.scaffold8351.3	2.71339	6.59387	-1.28103	0.000820679	sp|Q8N957|ANKF1_HUMAN Ankyrin repeat and fibronectin type-III domain-containing protein 1 OS=Homo sapiens GN=ANKFN1 PE=2 SV=2
evm.model.scaffold8351.1	163.43	404.523	-1.30755	0.000820679	sp|P0CAT2|S238B_DANRE Solute carrier family 25 member 38-B OS=Danio rerio GN=slc25a38b PE=3 SV=2
evm.model.scaffold160813.40	3.86656	10.1746	-1.39585	0.00213042	sp|Q6MZQ0|PRR5L_HUMAN Proline-rich protein 5-like OS=Homo sapiens GN=PRR5L PE=1 SV=2
evm.model.scaffold160813.42	96.5843	214.892	-1.15375	0.000820679	sp|Q9BQI0|AIF1L_HUMAN Allograft inflammatory factor 1-like OS=Homo sapiens GN=AIF1L PE=1 SV=1
evm.model.scaffold57017.6	0.387936	1.47404	-1.92589	0.00621905	sp|Q8JZL1|I17RD_MOUSE Interleukin-17 receptor D OS=Mus musculus GN=Il17rd PE=1 SV=1
evm.model.scaffold176361.27_evm.model.scaffold176361.28	3.74398	7.07814	-0.918798	0.00151774	sp|Q12768|STRUM_HUMAN WASH complex subunit strumpellin OS=Homo sapiens GN=KIAA0196 PE=1 SV=1
evm.model.scaffold83723.2	3.90811	8.60809	-1.13922	0.000820679	sp|Q95ZJ1|GALT5_CAEEL

					Polypeptide N-acetylgalactosaminyltransferase 5 OS=Caenorhabditis elegans GN=gly-5 PE=2 SV=2
evm.model.scaffold83723.1	3.22939	7.83291	-1.27829	0.00480132	sp|Q9H3G5|CPVL_HUMAN Probable serine carboxypeptidase CPVL OS=Homo sapiens GN=CPVL PE=1 SV=2
evm.model.scaffold114775.18	17.3906	34.4474	-0.986088	0.000820679	sp|Q9UJU6|DBNL_HUMAN Drebrin-like protein OS=Homo sapiens GN=DBNL PE=1 SV=1
evm.model.scaffold123009.12.1	1.71305	4.1107	-1.26281	0.000820679	--
evm.model.scaffold170775.1	1.16032	14.3836	-3.63183	0.00380231	--
evm.model.scaffold152525.11	25.8289	59.0784	-1.19364	0.000820679	sp|Q9UKW6|ELF5_HUMAN ETS-related transcription factor Elf-5 OS=Homo sapiens GN=ELF5 PE=1 SV=2
evm.model.scaffold154825.19	2.04971	3.69422	-0.849853	0.0476583	--
evm.model.scaffold169005.9	14.0675	28.5503	-1.02114	0.00271512	--
evm.model.scaffold140331.2	0.551855	1.53424	-1.47517	0.00326205	sp|Q01484|ANK2_HUMAN Ankyrin-2 OS=Homo sapiens GN=ANK2 PE=1 SV=4
evm.model.scaffold115693.38	3.8647	6.75187	-0.80493	0.00380231	sp|Q9H2Y9|SO5A1_HUMAN Solute carrier organic anion transporter family member 5A1 OS=Homo sapiens

					GN=SLCO5A1 PE=2 SV=2
evm.model.scaffold164279.1	7.05154	16.9204	-1.26275	0.000820679	sp|Q8K3Y7|TNF15_RAT Tumor necrosis factor ligand superfamily member 15 OS=Rattus norvegicus GN=Tnfsf15 PE=2 SV=1
evm.model.scaffold128139.31	5.01105	9.82398	-0.971195	0.0163478	sp|B2LU20|SFXN1_SHEEP Sideroflexin-1 OS=Ovis aries GN=SFXN1 PE=2 SV=1
evm.model.scaffold90031.20	0	1.33634	#NAME?	0.0373071	sp|P48450|ERG7_RAT Lanosterol synthase OS=Rattus norvegicus GN=Lss PE=1 SV=2
evm.model.scaffold4289.12	2.70368	4.46064	-0.722325	0.014476	sp|Q68CJ6|SLIP_HUMAN Nuclear GTPase SLIP-GC OS=Homo sapiens GN=NUGGC PE=2 SV=3
evm.model.scaffold121031.31	33.1978	66.8471	-1.00978	0.000820679	sp|Q6NVA9|ACTB_XENTR Actin, cytoplasmic 1 OS=Xenopus tropicalis GN=actb PE=2 SV=1
evm.model.scaffold121031.33	4.55381	13.6968	-1.58869	0.000820679	sp|P45886|ACT3_BACDO Actin-3, muscle-specific OS=Bactrocera dorsalis PE=2 SV=1
evm.model.scaffold121031.32	0.42085	4.53213	-3.42881	0.000820679	sp|Q93131|ACTC_BRAFL Actin, cytoplasmic OS=Branchiostoma floridae PE=2 SV=1
evm.model.scaffold104507.16	5.61121	10.3511	-0.883399	0.00213042	sp|O70511|ANK3_RAT Ankyrin-3 OS=Rattus norvegicus GN=Ank3 PE=1

					SV=3
evm.model.scaffold175229.7	1.26599	2.70727	-1.09657	0.0245648	sp|A1A5H8|YES_DANRE Tyrosine-protein kinase yes OS=Danio rerio GN=yes1 PE=1 SV=1
evm.model.scaffold175229.5	29.2994	55.1173	-0.911635	0.00151774	sp|Q1JPZ3|SRC_DANRE Proto-oncogene tyrosine-protein kinase Src OS=Danio rerio GN=src PE=1 SV=2
evm.model.scaffold112415.3.1	1.88721	2.97093	-0.65466	0.0396592	sp|B1H349|SOX6_XENTR Transcription factor Sox-6 OS=Xenopus tropicalis GN=sox6 PE=2 SV=1
evm.model.scaffold90825.2	6.49479	16.5379	-1.34842	0.000820679	--
evm.model.scaffold90825.3	5.07811	13.4353	-1.40366	0.000820679	--
evm.model.scaffold57357.6	22.0313	45.2054	-1.03694	0.000820679	sp|Q17029|VATF_ANOGA V-type proton ATPase subunit F OS=Anopheles gambiae GN=Vha14 PE=2 SV=2
evm.model.scaffold69685.8	0	3.07865	#NAME?	0.00271512	--
evm.model.scaffold106789.12	0.920829	8.6887	-3.23813	0.00213042	sp|Q96KQ7|EHMT2_HUMAN Histone-lysine N-methyltransferase EHMT2 OS=Homo sapiens GN=EHMT2 PE=1 SV=3
evm.model.scaffold111921.46	0	0.785235	#NAME?	0.000820679	sp|P31331|GLB_NASMU Globin OS=Nassarius mutabilis PE=1 SV=1

evm.model.scaffold51453.19	12.5297	24.7809	-0.98388	0.00933293	sp|Q6P8E9|NH2L1_XENTR NHP2-like protein 1 OS=Xenopus tropicalis GN=nhp2l1 PE=2 SV=1
evm.model.scaffold154539.8	4.42644	7.16456	-0.694733	0.0276479	sp|Q2EMV9|PAR14_MOUSE Poly [ADP-ribose] polymerase 14 OS=Mus musculus GN=Parp14 PE=1 SV=3
evm.model.scaffold155289.1	11.0646	46.0642	-2.0577	0.00151774	sp|Q9UHL4|DPP2_HUMAN Dipeptidyl peptidase 2 OS=Homo sapiens GN=DPP7 PE=1 SV=3
evm.model.scaffold133259.29	3.63628	7.93793	-1.1263	0.000820679	sp|A3KFU9|PTHD2_MOUSE Patched domain-containing protein 2 OS=Mus musculus GN=Ptchd2 PE=2 SV=1
evm.model.scaffold98043.21	9.9233	15.6126	-0.653824	0.0335734	sp|Q96P53|WDFY2_HUMAN WD repeat and FYVE domain-containing protein 2 OS=Homo sapiens GN=WDFY2 PE=2 SV=2
evm.model.scaffold169321.4	42.7665	66.927	-0.646108	0.0299214	sp|P26801|CEBPG_RAT CCAAT/enhancer-binding protein gamma OS=Rattus norvegicus GN=Cebpg PE=2 SV=2
evm.model.scaffold169321.3	22.8337	51.3915	-1.17036	0.000820679	sp|Q05826|CEBPB_CHICK CCAAT/enhancer-binding protein beta OS=Gallus gallus GN=CEBPB PE=1 SV=1
evm.model.scaffold78661.8	2.52457	4.45187	-0.818374	0.0376361	sp|Q8T2Q0|ZDHC6_DICDI Putative

					ZDHHC-type palmitoyltransferase 6 OS=Dictyostelium discoideum GN=DDB_G0275149 PE=2 SV=1
evm.model.scaffold136529.8	1.19341	7.05549	-2.56365	0.000820679	--
evm.model.scaffold127189.26	2.06596	5.11928	-1.30913	0.00380231	--
evm.model.scaffold51225.71	3.64241	9.78698	-1.42597	0.000820679	--
evm.model.scaffold82531.7	0.284014	1.68007	-2.56449	0.00326205	--
evm.model.scaffold81773.93	0.71022	2.76157	-1.95915	0.00213042	sp|Q969Z3|MARC2_HUMAN Mitochondrial amidoxime reducing component 2 OS=Homo sapiens GN=MARC2 PE=1 SV=1
evm.model.scaffold73223.10	0.106969	2.05023	-4.26053	0.00213042	sp|Q2KIR7|GLYAT_BOVIN Glycine N-acyltransferase OS=Bos taurus GN=GLYAT PE=1 SV=2
evm.model.scaffold26189.59	1.90289	4.05618	-1.09193	0.00326205	sp|Q09128|CP24A_RAT 1,25-dihydroxyvitamin D(3) 24-hydroxylase, mitochondrial OS=Rattus norvegicus GN=Cyp24a1 PE=1 SV=1
evm.model.scaffold47427.2	7.09937	12.7332	-0.842831	0.0429196	--
evm.model.scaffold134743.27	42.5566	305.267	-2.84261	0.000820679	sp|Q54TR1|CFAD_DICDI Counting factor associated protein D OS=Dictyostelium discoideum GN=cfaD PE=1 SV=1
evm.model.scaffold27973.176	4.88676	7.76426	-0.667969	0.0457321	sp|Q9ULM0|PKHH1_HUMAN

					Pleckstrin homology domain-containing family H member 1 OS=Homo sapiens GN=PLEKHH1 PE=2 SV=2
evm.model.scaffold27973.175	6.88566	12.1757	-0.822335	0.00578348	sp|P97479|MYO7A_MOUSE Unconventional myosin-VIIa OS=Mus musculus GN=Myo7a PE=1 SV=2
evm.model.scaffold65283.13	2.26406	4.05974	-0.842478	0.029403	sp|P25107|PTH1R_DIDVI Parathyroid hormone/parathyroid hormone-related peptide receptor OS=Didelphis virginiana GN=PTH1R PE=2 SV=2
evm.model.scaffold169113.43	2.94162	5.66863	-0.946389	0.00429787	sp|O95352|ATG7_HUMAN Ubiquitin-like modifier-activating enzyme ATG7 OS=Homo sapiens GN=ATG7 PE=1 SV=1
evm.model.scaffold15937.5	9.2791	17.0537	-0.878029	0.00621905	sp|A2SWM2|SPNS2_DANRE Protein spinster homolog 2 OS=Danio rerio GN=spns2 PE=1 SV=2
evm.model.scaffold115633.52	2.4404	5.08078	-1.05793	0.0468595	sp|Q0V9U8|PHIPL_XENTR Phytanoyl-CoA hydroxylase-interacting protein-like OS=Xenopus tropicalis GN=phyhipl PE=2 SV=1
evm.model.scaffold115633.53	3.36339	8.86107	-1.39757	0.00429787	sp|Q0V9U8|PHIPL_XENTR Phytanoyl-CoA

					hydroxylase-interacting protein-like OS=Xenopus tropicalis GN=phyhipl PE=2 SV=1
evm.model.scaffold115633.51	11.5356	41.9588	-1.86288	0.000820679	sp|Q0V9U8|PHIPL_XENTR Phytanoyl-CoA hydroxylase-interacting protein-like OS=Xenopus tropicalis GN=phyhipl PE=2 SV=1
evm.model.scaffold128139.28	0.396625	2.30078	-2.53628	0.00326205	sp|Q91790|DUS1A_XENLA Dual specificity protein phosphatase 1-A OS=Xenopus laevis GN=dusp1-a PE=1 SV=1
evm.model.scaffold175399.2	1.96506	4.06506	-1.0487	0.000820679	sp|A2AJ76|HMCN2_MOUSE Hemicentin-2 OS=Mus musculus GN=Hmcn2 PE=2 SV=1
evm.model.scaffold167889.17	10.9219	19.0483	-0.802435	0.00972356	sp|Q5R6K8|PP4C_PONAB Serine/threonine-protein phosphatase 4 catalytic subunit OS=Pongo abelii GN=PPP4C PE=2 SV=1
evm.model.scaffold175495.46	27.4438	40.8051	-0.572271	0.0484321	sp|Q6NYT3|IER5L_DANRE Immediate early response gene 5-like protein OS=Danio rerio GN=ier5l PE=2 SV=2
evm.model.scaffold174445.1	1.61329	3.82469	-1.24533	0.000820679	sp|P16157|ANK1_HUMAN Ankyrin-1 OS=Homo sapiens

					GN=ANK1 PE=1 SV=3
evm.model.scaffold116119.149	2.39368	6.12695	-1.35594	0.000820679	sp|Q923X1|ELTD1_MOUSE EGF, latrophilin seven transmembrane domain-containing protein 1 OS=Mus musculus GN=Eltd1 PE=2 SV=3
evm.model.scaffold134743.8	8.71405	16.3785	-0.910392	0.0218619	sp|Q8R4F0|MCLN3_MOUSE Mucolipin-3 OS=Mus musculus GN=Mcoln3 PE=1 SV=1
evm.model.scaffold134743.5	1.07152	3.10777	-1.53622	0.0156116	sp|Q28658|SPRR3_RABIT Small proline-rich protein 3 OS=Oryctolagus cuniculus GN=SPRR3 PE=2 SV=1
evm.model.scaffold134743.6	5.77661	11.6091	-1.00696	0.0245648	sp|Q8R4F0|MCLN3_MOUSE Mucolipin-3 OS=Mus musculus GN=Mcoln3 PE=1 SV=1
evm.model.scaffold141143.8	1.91733	5.69554	-1.57073	0.000820679	sp|Q8NBS3|S4A11_HUMAN Sodium bicarbonate transporter-like protein 11 OS=Homo sapiens GN=SLC4A11 PE=1 SV=2
evm.model.scaffold124911.20	1.38116	2.74971	-0.993393	0.0434957	sp|P12348|PER_DROPS Period circadian protein OS=Drosophila pseudoobscura pseudoobscura GN=per PE=3 SV=3
evm.model.scaffold52321.51	3.02381	6.95937	-1.20259	0.00755259	sp|P24329|THTR_RAT Thiosulfate sulfurtransferase OS=Rattus norvegicus GN=Tst PE=1 SV=3

evm.model.scaffold95641.51	16.6906	36.4335	-1.12623	0.000820679	sp|Q28619|NHRF1_RABIT Na(+)/H(+) exchange regulatory cofactor NHE-RF1 OS=Oryctolagus cuniculus GN=SLC9A3R1 PE=1 SV=3
evm.model.scaffold103151.3	15.314	33.3088	-1.12105	0.000820679	sp|Q32LN0|EHF_BOVIN ETS homologous factor OS=Bos taurus GN=EHF PE=2 SV=1
evm.model.scaffold78787.20	9.00603	14.8835	-0.724749	0.0191834	sp|P51688|SPHM_HUMAN N-sulphoglucosamine sulphohydrolase OS=Homo sapiens GN=SGSH PE=1 SV=1
evm.model.scaffold132039.1	30.6242	61.8701	-1.01457	0.00271512	sp|Q24799|MYPH_ECHGR Myophilin OS=Echinococcus granulosus PE=2 SV=1
evm.model.scaffold94347.8	221.662	374.094	-0.75504	0.0296885	sp|Q26534|CATL_SCHMA Cathepsin L OS=Schistosoma mansoni GN=CL1 PE=2 SV=1
evm.model.scaffold138629.26	25.9209	44.2217	-0.770639	0.0370657	sp|Q4LDE5|SVEP1_HUMAN Sushi, von Willebrand factor type A, EGF and pentraxin domain-containing protein 1 OS=Homo sapiens GN=SVEP1 PE=1 SV=3
evm.model.scaffold138629.25	0.34993	1.81352	-2.37365	0.023485	sp|Q9WUU7|CATZ_MOUSE Cathepsin Z OS=Mus musculus GN=Ctsz PE=2 SV=1

evm.model.scaffold167227.8	4.76595	10.0018	-1.06942	0.00326205	sp|Q9UHL4|DPP2_HUMAN Dipeptidyl peptidase 2 OS=Homo sapiens GN=DPP7 PE=1 SV=3
evm.model.scaffold167227.6	1.54193	4.68721	-1.60399	0.000820679	sp|O95999|BCL10_HUMAN B-cell lymphoma/leukemia 10 OS=Homo sapiens GN=BCL10 PE=1 SV=1
evm.model.scaffold158547.84_evm.model.scaffold158547.85	24.3479	80.2421	-1.72056	0.000820679	sp|A1E295|CATB_PIG Cathepsin B OS=Sus scrofa GN=CTSB PE=1 SV=1
evm.model.scaffold154475.7	2.0193	4.39645	-1.12249	0.00271512	sp|P22105|TENX_HUMAN Tenascin-X OS=Homo sapiens GN=TNXB PE=1 SV=4
evm.model.scaffold24751.36	8.35544	24.7682	-1.5677	0.0361439	--
evm.model.scaffold174215.1	2.47394	5.23024	-1.08007	0.0427039	--
evm.model.scaffold94547.9	5.98098	35.7859	-2.58094	0.000820679	--
evm.model.scaffold165645.23	3.77093	16.0383	-2.08853	0.000820679	--
evm.model.scaffold21299.6	2.68066	6.8668	-1.35705	0.000820679	sp|P59694|TNFA_LAMGL Tumor necrosis factor OS=Lama glama GN=TNF PE=2 SV=1
evm.model.scaffold90031.61	13.8882	24.0856	-0.794318	0.00578348	sp|P11584|ITBX_DROME Integrin beta-PS OS=Drosophila melanogaster GN=mys PE=1 SV=3
evm.model.scaffold166273.4	11.0778	59.2725	-2.41969	0.000820679	--
evm.model.scaffold112415.1	2.74949	6.06598	-1.14158	0.0332993	sp|O54804|CHKA_MOUSE Choline kinase alpha OS=Mus musculus GN=Chka PE=1 SV=3

evm.model.scaffold72179.2	20.387	42.2791	-1.0523	0.000820679	--
evm.model.scaffold72179.3	36.1421	61.9979	-0.778538	0.00429787	sp|Q8HZK2|DUOX2_PIG Dual oxidase 2 OS=Sus scrofa GN=DUOX2 PE=1 SV=2
evm.model.scaffold72179.7	22.3264	38.6518	-0.791788	0.00213042	sp|O60749|SNX2_HUMAN Sorting nexin-2 OS=Homo sapiens GN=SNX2 PE=1 SV=2
evm.model.scaffold38291.38	3.41922	7.04942	-1.04384	0.00532397	sp|P18433|PTPRA_HUMAN Receptor-type tyrosine-protein phosphatase alpha OS=Homo sapiens GN=PTPRA PE=1 SV=2
evm.model.scaffold83415.2	4.74809	9.51405	-1.00271	0.00972356	sp|Q5XGK0|SPNS1_XENLA Protein spinster homolog 1 OS=Xenopus laevis GN=spns1 PE=2 SV=1
evm.model.scaffold5455.42	11.1234	23.5091	-1.07962	0.000820679	sp|Q0VGW6|S12A9_XENLA Solute carrier family 12 member 9 OS=Xenopus laevis GN=slc12a9 PE=2 SV=1
evm.model.scaffold6943.2	1.57171	3.6671	-1.22231	0.0416339	sp|Q96CD0|FBXL8_HUMAN F-box/LRR-repeat protein 8 OS=Homo sapiens GN=FBXL8 PE=1 SV=1
evm.model.scaffold52869.11	7.86936	20.9545	-1.41294	0.000820679	sp|P82450|SIAE_RAT Sialate O-acetylesterase OS=Rattus norvegicus GN=Siae PE=1 SV=2
evm.model.scaffold176475.8	21.7072	39.8842	-0.877644	0.000820679	sp|P81134|RENR_BOVIN Renin

					receptor OS=Bos taurus GN=ATP6AP2 PE=1 SV=2
evm.model.scaffold91157.5	0	2.02117	#NAME?	0.000820679	--
evm.model.scaffold136165.53	7.27109	21.0878	-1.53616	0.000820679	sp|Q14162|SREC_HUMAN Scavenger receptor class F member 1 OS=Homo sapiens GN=SCARF1 PE=1 SV=3
evm.model.scaffold176213.1	11.722	23.7229	-1.01706	0.000820679	sp|P42674|BP10_PARLI Blastula protease 10 OS=Paracentrotus lividus GN=BP10 PE=2 SV=1
evm.model.scaffold105815.20	0.548963	2.2271	-2.02039	0.00578348	--
evm.model.scaffold176323.9.1	1.90548	2.99323	-0.651552	0.0396592	sp|P10079|FBP1_STRPU Fibropellin-1 OS=Strongylocentrotus purpuratus GN=EGF1 PE=1 SV=2
evm.model.scaffold16799.3	3.11259	5.26938	-0.759521	0.0264598	sp|Q9W534|MOODY_DROME G-protein coupled receptor moody OS=Drosophila melanogaster GN=moody PE=2 SV=2
evm.model.scaffold135929.49	0.884542	2.00693	-1.18199	0.0205228	sp|Q149C3|LIGO4_MOUSE Leucine-rich repeat and immunoglobulin-like domain containing-NOGO receptor-interacting protein 4 OS=Mus musculus GN=Lingo4 PE=2 SV=2
evm.model.scaffold175329.2	0.853006	2.23774	-1.39142	0.00712329	sp|P58151|YCXC_EUGLO Uncharacterized 51.9 kDa protein in

					rps4-rps11 intergenic region OS=Euglena longa PE=4 SV=1
evm.model.scaffold148655.9	0.539597	2.53094	-2.22972	0.000820679	--
evm.model.scaffold49899.3	0.235812	1.06132	-2.17015	0.00380231	sp|Q20363|SIP1_CAEEL Stress-induced protein 1 OS=Caenorhabditis elegans GN=sip-1 PE=1 SV=1
evm.model.scaffold36941.13	4.81528	11.0206	-1.19451	0.00800162	sp|Q32KJ8|ARSI_RAT Arylsulfatase I OS=Rattus norvegicus GN=Arsi PE=2 SV=1
evm.model.scaffold36941.12	1.34842	7.33982	-2.44448	0.000820679	sp|Q5FYB0|ARSJ_HUMAN Arylsulfatase J OS=Homo sapiens GN=ARSJ PE=2 SV=1
evm.model.scaffold27189.42_evm.model.scaffold27189.43	4.54541	10.2787	-1.17717	0.000820679	sp|Q9NZJ5|E2AK3_HUMAN Eukaryotic translation initiation factor 2-alpha kinase 3 OS=Homo sapiens GN=EIF2AK3 PE=1 SV=3
evm.model.scaffold56755.125	7.79335	17.6306	-1.17777	0.000820679	sp|Q8BX37|PAPL_MOUSE Iron/zinc purple acid phosphatase-like protein OS=Mus musculus GN=Papl PE=2 SV=2
evm.model.scaffold140913.8	6.58671	10.6488	-0.693063	0.0413639	sp|O14522|PTPRT_HUMAN Receptor-type tyrosine-protein phosphatase T OS=Homo sapiens GN=PTPRT PE=1 SV=6

evm.model.scaffold140913.3	3.58157	6.07186	-0.761546	0.0282044	sp|Q5REG4|DTX3_PONAB Probable E3 ubiquitin-protein ligase DTX3 OS=Pongo abelii GN=DTX3 PE=2 SV=1
evm.model.scaffold9945.43.1	12.2648	25.6753	-1.06586	0.000820679	sp|Q98892|OBCAM_CHICK Opioid-binding protein/cell adhesion molecule homolog OS=Gallus gallus GN=OPCML PE=1 SV=2
evm.model.scaffold136409.13	104.495	501.88	-2.26391	0.000820679	sp|Q5R5J1|PCKGC_PONAB Phosphoenolpyruvate carboxykinase, cytosolic [GTP] OS=Pongo abelii GN=PCK1 PE=2 SV=1
evm.model.scaffold157537.10	0.167563	0.576396	-1.78235	0.0382321	--
evm.model.scaffold124269.84	246.855	637.437	-1.36862	0.000820679	sp|P10984|ACT2_CAEEL Actin-2 OS=Caenorhabditis elegans GN=act-2 PE=3 SV=3
evm.model.scaffold157537.19	0.134206	0.942719	-2.81238	0.000820679	sp|Q0S7V5|FAD3_RHOJR 3-[(3aS,4S,7aS)-7a-methyl-1, 5-dioxo-octahydro-1H-inden-4-yl]propanoyl:CoA ligase OS=Rhodococcus jostii (strain RHA1) GN=fadD3 PE=1 SV=1
evm.model.scaffold72763.113	5.71632	9.55428	-0.74106	0.0351039	sp|Q6GQN4|FA32A_DANRE Protein FAM32A-like OS=Danio rerio GN=fam32al PE=3 SV=1
evm.model.scaffold72763.110	32.5811	136.163	-2.06323	0.000820679	sp|Q9U943|APLP_LOCMI

					Apolipophorins OS=Locusta migratoria PE=1 SV=2
evm.model.scaffold247.17	1.5875	2.9986	-0.917527	0.027885	--
evm.model.scaffold42375.20	1.69635	3.07116	-0.856347	0.0332993	sp|Q92609|TBCD5_HUMAN TBC1 domain family member 5 OS=Homo sapiens GN=TBC1D5 PE=1 SV=1
evm.model.scaffold98983.3	27.8642	43.6294	-0.64689	0.0274321	sp|P78356|PI42B_HUMAN Phosphatidylinositol 5-phosphate 4-kinase type-2 beta OS=Homo sapiens GN=PIP4K2B PE=1 SV=1
evm.model.scaffold176307.3	8.44622	15.2134	-0.848962	0.00380231	sp|O89026|ROBO1_MOUSE Roundabout homolog 1 OS=Mus musculus GN=Robo1 PE=1 SV=1
evm.model.scaffold176307.2	0.0946548	1.00383	-3.4067	0.000820679	sp|Q13332|PTPRS_HUMAN Receptor-type tyrosine-protein phosphatase S OS=Homo sapiens GN=PTPRS PE=1 SV=3
evm.model.scaffold166837.39	0.462703	1.47834	-1.67582	0.00621905	--
evm.model.scaffold73127.2.1	1.26715	2.71281	-1.0982	0.0101381	sp|P24043|LAMA2_HUMAN Laminin subunit alpha-2 OS=Homo sapiens GN=LAMA2 PE=1 SV=4
evm.model.scaffold146247.55	12.3676	19.656	-0.668401	0.0218619	sp|Q641Z2|PTN9_RAT Tyrosine-protein phosphatase non-receptor type 9 OS=Rattus norvegicus GN=Ptpn9 PE=2 SV=1

evm.model.scaffold174263.11_evm.model.scaffold174263.10	16.5602	30.755	-0.893099	0.000820679	sp|A2X6E6|SUT5_ORYSI Sucrose transport protein SUT5 OS=Oryza sativa subsp. indica GN=SUT5 PE=3 SV=1
evm.model.scaffold20575.31	5.86342	12.9428	-1.14234	0.00532397	--
evm.model.scaffold149093.9	13.3881	35.4894	-1.40644	0.000820679	sp|Q9WTR0|MMP16_MOUSE Matrix metalloproteinase-16 OS=Mus musculus GN=Mmp16 PE=2 SV=3
evm.model.scaffold56279.9	7.45141	18.607	-1.32026	0.000820679	sp|Q9I8C7|ACH10_CHICK Neuronal acetylcholine receptor subunit alpha-10 OS=Gallus gallus GN=CHRNA10 PE=3 SV=1
evm.model.scaffold167403.16	20.802	66.3324	-1.67299	0.000820679	sp|Q6GM59|MOT12_XENLA Monocarboxylate transporter 12 OS=Xenopus laevis GN=slc16a12 PE=2 SV=1
evm.model.scaffold171689.48	2.30947	11.3479	-2.29679	0.000820679	--
evm.model.scaffold9075.23	7.19622	34.1693	-2.24739	0.000820679	sp|Q92982|NINJ1_HUMAN Ninjurin-1 OS=Homo sapiens GN=NINJ1 PE=1 SV=2
evm.model.scaffold9075.25	5.24466	15.8133	-1.59222	0.000820679	sp|P20933|ASPG_HUMAN N(4)-(beta-N-acetylglucosaminyl)-L-asparaginase OS=Homo sapiens GN=AGA PE=1 SV=2
evm.model.scaffold141143.12	3.95406	13.6218	-1.78451	0.000820679	sp|Q60HH4|ASAH1_MACFA Acid

					ceramidase OS=Macaca fascicularis GN=ASAH1 PE=2 SV=1
evm.model.scaffold176231.4	4.01595	21.0404	-2.38935	0.000820679	--
evm.model.scaffold6571.45	1.29276	2.87518	-1.1532	0.0117709	sp|Q9NQ36|SCUB2_HUMAN Signal peptide, CUB and EGF-like domain-containing protein 2 OS=Homo sapiens GN=SCUBE2 PE=2 SV=2
evm.model.scaffold109197.1	4.48474	13.9096	-1.63298	0.000820679	--
evm.model.scaffold109197.2	8.98402	23.9628	-1.41536	0.000820679	--
evm.model.scaffold133871.1	0.348594	1.19466	-1.77698	0.0370657	sp|B3A0P4|USP26_LOTGI Uncharacterized shell protein 26 (Fragment) OS=Lottia gigantea PE=1 SV=1
evm.model.scaffold146869.13	2.94824	5.92673	-1.00738	0.014476	--
evm.model.scaffold146869.12	1.87105	3.53275	-0.916942	0.0437576	sp|Q9JLL3|TNR19_MOUSE Tumor necrosis factor receptor superfamily member 19 OS=Mus musculus GN=Tnfrsf19 PE=2 SV=2
evm.model.scaffold148273.3	5.29327	13.029	-1.29949	0.000820679	sp|P12276|FAS_CHICK Fatty acid synthase OS=Gallus gallus GN=FASN PE=1 SV=5
evm.model.scaffold80269.1	2.37305	6.15826	-1.37578	0.000820679	sp|Q99758|ABCA3_HUMAN ATP-binding cassette sub-family A member 3 OS=Homo sapiens

					GN=ABCA3 PE=1 SV=2
evm.model.scaffold84343.1	3.04655	6.33473	-1.05611	0.000820679	sp|Q9NZJ4|SACS_HUMAN Sacsin OS=Homo sapiens GN=SACS PE=1 SV=2
evm.model.scaffold84343.2	0.0925613	0.434463	-2.23075	0.0420901	--
evm.model.scaffold67769.23	6.4248	20.1887	-1.65183	0.000820679	sp|P09960|LKHA4_HUMAN Leukotriene A-4 hydrolase OS=Homo sapiens GN=LTA4H PE=1 SV=2
evm.model.scaffold176609.7	5.65865	13.5556	-1.26036	0.000820679	sp|Q8BS03|PI15_MOUSE Peptidase inhibitor 15 OS=Mus musculus GN=Pi15 PE=2 SV=2
evm.model.scaffold26693.27	0.373975	1.2998	-1.79728	0.0274321	--
evm.model.scaffold31897.1	67.8735	201.107	-1.56704	0.00888556	sp|Q8BG94|COMD7_MOUSE COMM domain-containing protein 7 OS=Mus musculus GN=Commd7 PE=2 SV=1
evm.model.scaffold71915.1	12.499	23.4446	-0.907446	0.000820679	sp|Q8K1N1|PLPL8_MOUSE Calcium-independent phospholipase A2-gamma OS=Mus musculus GN=Pnpla8 PE=1 SV=1
evm.model.scaffold51139.27	2.21602	12.5969	-2.50703	0.00326205	sp|A1E2V0|BIRC3_CANFA Baculoviral IAP repeat-containing protein 3 OS=Canis familiaris GN=BIRC3 PE=2 SV=1
evm.model.scaffold51139.24	0.645799	2.58105	-1.9988	0.00800162	sp|Q60989|XIAP_MOUSE E3 ubiquitin-protein ligase XIAP OS=Mus

					musculus GN=Xiap PE=1 SV=2
evm.model.scaffold82159.3	17.1121	29.6935	-0.795128	0.0169923	sp|Q6W5P4|NPSR1_HUMAN Neuropeptide S receptor OS=Homo sapiens GN=NPSR1 PE=1 SV=1
evm.model.scaffold82159.5	4.6914	10.6339	-1.18058	0.000820679	sp|O15973|OPSD1_MIZYE Rhodopsin, GQ-coupled OS=Mizuhopecten yessoensis GN=SCOP1 PE=1 SV=1
evm.model.scaffold82159.7	0.921163	2.44595	-1.40887	0.0105331	sp|Q01414|ERG_LYTVA Transcriptional regulator ERG homolog (Fragment) OS=Lytechinus variegatus GN=ERG PE=3 SV=1
evm.model.scaffold142385.6	10.0327	19.6208	-0.967665	0.000820679	sp|P23469|PTPRE_HUMAN Receptor-type tyrosine-protein phosphatase epsilon OS=Homo sapiens GN=PTPRE PE=1 SV=1
evm.model.scaffold109207.6	0.460257	1.60314	-1.80039	0.000820679	sp|Q8CG09|MRP1_RAT Multidrug resistance-associated protein 1 OS=Rattus norvegicus GN=Abcc1 PE=1 SV=2
evm.model.scaffold138799.2.1	13.0996	25.3254	-0.95106	0.0268133	sp|Q55FM5|MTPN_DICDI Myotrophin homolog OS=Dictyostelium discoideum GN=mtpn PE=3 SV=1
evm.model.scaffold136775.21	6.97675	12.1842	-0.804385	0.0497358	sp|Q91VN0|LRP5_MOUSE

					Low-density lipoprotein receptor-related protein 5 OS=Mus musculus GN=Lrp5 PE=1 SV=3
evm.model.scaffold91053.1	8.24046	25.1125	-1.60761	0.00800162	sp|A8WGS4|TLCD2_DANRE TLC domain-containing protein 2 OS=Danio rerio GN=tlcd2 PE=2 SV=1
evm.model.scaffold147433.32	1.81742	3.80051	-1.0643	0.0141107	sp|A0JM12|MEG10_XENTR Multiple epidermal growth factor-like domains protein 10 OS=Xenopus tropicalis GN=megf10 PE=2 SV=1
evm.model.scaffold147433.31	0.539913	2.05535	-1.92858	0.0169923	sp|Q14162|SREC_HUMAN Scavenger receptor class F member 1 OS=Homo sapiens GN=SCARF1 PE=1 SV=3
evm.model.scaffold6943.54	5.91722	9.54646	-0.690045	0.0353697	sp|Q6P6Y1|K1468_DANRE LisH domain and HEAT repeat-containing protein KIAA1468 homolog OS=Danio rerio GN=zgc:66014 PE=2 SV=1
evm.model.scaffold157355.7	30.7095	52.7612	-0.780795	0.00712329	sp|Q7Z0Q2|RNKB_CERCA Ribonuclease kappa-B OS=Ceratitis capitata PE=3 SV=1
evm.model.scaffold157355.9	23.5963	85.9016	-1.86412	0.000820679	--
evm.model.scaffold157355.8	23.5474	62.4995	-1.40828	0.000820679	sp|Q7ZUV1|PKHF2_DANRE Pleckstrin homology domain-containing family F member 2 OS=Danio rerio GN=plekhf2 PE=2

					SV=1
evm.model.scaffold51453.40	2.08542	5.10328	-1.29109	0.00151774	sp|P58727|TLR4_FELCA Toll-like receptor 4 OS=Felis catus GN=TLR4 PE=2 SV=1
evm.model.scaffold20843.3	0.488087	1.26075	-1.36907	0.00712329	sp|Q69Z37|SAM9L_MOUSE Sterile alpha motif domain-containing protein 9-like OS=Mus musculus GN=Samd9l PE=1 SV=2
evm.model.scaffold176475.11	27.8093	43.3214	-0.639513	0.0335734	--
evm.model.scaffold1304202.1	49.0472	161.533	-1.71959	0.0101381	sp|Q63HN8|RN213_HUMAN E3 ubiquitin-protein ligase RNF213 OS=Homo sapiens GN=RNF213 PE=1 SV=3
evm.model.scaffold95039.20	0.146596	0.812731	-2.47093	0.0396592	--
evm.model.scaffold176027.33	5.25147	11.4009	-1.11836	0.000820679	--
evm.model.scaffold133567.11	8.7771	26.7069	-1.6054	0.000820679	sp|Q6V289|ABRAL_COTCO Costars family protein ABRACL OS=Coturnix coturnix PE=3 SV=2
evm.model.scaffold61991.23	1.12855	2.16552	-0.94025	0.00326205	sp|Q4LDE5|SVEP1_HUMAN Sushi, von Willebrand factor type A, EGF and pentraxin domain-containing protein 1 OS=Homo sapiens GN=SVEP1 PE=1 SV=3
evm.model.scaffold137173.4	0.574805	5.25639	-3.19293	0.00380231	sp|P55006|RDH7_RAT Retinol dehydrogenase 7 OS=Rattus

					norvegicus GN=Rdh7 PE=2 SV=1
evm.model.scaffold123079.4	27.3043	41.63	-0.608497	0.0413639	sp|Q9R1S4|XBP1_RAT X-box-binding protein 1 OS=Rattus norvegicus GN=Xbp1 PE=2 SV=1
evm.model.scaffold174263.1	2.02074	6.74219	-1.73833	0.000820679	sp|Q7Z2H8|S36A1_HUMAN Proton-coupled amino acid transporter 1 OS=Homo sapiens GN=SLC36A1 PE=1 SV=1
evm.model.scaffold151467.134	3.41244	5.59674	-0.713784	0.0129481	sp|O43345|ZN208_HUMAN Zinc finger protein 208 OS=Homo sapiens GN=ZNF208 PE=2 SV=2
evm.model.scaffold71175.1	5.17719	10.4666	-1.01556	0.00429787	sp|O09175|AMPB_RAT Aminopeptidase B OS=Rattus norvegicus GN=Rnpep PE=1 SV=2
evm.model.scaffold71175.2	6.26797	46.6734	-2.89653	0.000820679	--
evm.model.scaffold84455.14_evm.model.scaffold84455.15	5.84755	10.3819	-0.828161	0.00578348	sp|Q07553|GCY3E_DROME Guanylate cyclase 32E OS=Drosophila melanogaster GN=Gyc32E PE=1 SV=4
evm.model.scaffold53733.30	12.3216	25.6715	-1.05898	0.000820679	sp|Q9V3J1|VATH_DROME V-type proton ATPase subunit H OS=Drosophila melanogaster GN=VhaSFD PE=2 SV=2
evm.model.scaffold30559.11	1.05425	4.93396	-2.22653	0.00271512	--
evm.model.scaffold64407.41	33.4749	50.5736	-0.595304	0.0471091	sp|C3KHG1|CR032_ANOFI UPF0729 protein C18orf32 homolog

					OS=Anoplopoma fimbria PE=3 SV=1
evm.model.scaffold44553.20	2.92419	5.6465	-0.94932	0.00380231	sp|P51945|CCNG1_MOUSE Cyclin-G1 OS=Mus musculus GN=Ccng1 PE=2 SV=2
evm.model.scaffold166055.19.3	12.0252	20.7084	-0.784148	0.0117709	sp|Q4QQM4|P5I11_MOUSE Tumor protein p53-inducible protein 11 OS=Mus musculus GN=Trp53i11 PE=2 SV=1
evm.model.scaffold2427.13	4.4716	7.71928	-0.787674	0.0447111	sp|O55196|ENAM_MOUSE Enamelin OS=Mus musculus GN=Enam PE=1 SV=1
evm.model.scaffold146053.1	7.16807	14.4991	-1.01631	0.000820679	sp|Q5RAJ6|DJB11_PONAB DnaJ homolog subfamily B member 11 OS=Pongo abelii GN=DNAJB11 PE=2 SV=1
evm.model.scaffold95051.18.2	1.01453	2.53649	-1.32202	0.0241881	sp|P79110|TXTP_BOVIN Tricarboxylate transport protein, mitochondrial OS=Bos taurus GN=SLC25A1 PE=2 SV=1
evm.model.scaffold107255.17	7.48673	14.7836	-0.981594	0.000820679	sp|Q498K0|GALC_XENLA Galactocerebrosidase OS=Xenopus laevis GN=galc PE=2 SV=2
evm.model.scaffold81473.67	1.18479	3.58699	-1.59814	0.00213042	sp|Q9LJN4|BXL5_ARATH Probable beta-D-xylosidase 5 OS=Arabidopsis thaliana GN=BXL5 PE=2 SV=2

evm.model.scaffold27973.146	7.58645	16.8507	-1.15131	0.000820679	sp|Q6R5N8|TLR13_MOUSE Toll-like receptor 13 OS=Mus musculus GN=Tlr13 PE=1 SV=1
evm.model.scaffold20791.6	1.23914	2.50258	-1.01408	0.0117709	sp|Q66T02|PKHG5_MOUSE Pleckstrin homology domain-containing family G member 5 OS=Mus musculus GN=Plekhg5 PE=1 SV=1
evm.model.scaffold169113.17	10.118	20.7612	-1.03697	0.00151774	sp|P46437|GST_MUSDO Glutathione S-transferase OS=Musca domestica PE=2 SV=1
evm.model.scaffold116119.175	2.98763	6.24308	-1.06325	0.00326205	sp|Q0PV50|TLR3_BOSTR Toll-like receptor 3 OS=Boselaphus tragocamelus GN=TLR3 PE=2 SV=1
evm.model.scaffold159271.6	2.91367	4.50181	-0.62767	0.0318917	sp|Q7Z333|SETX_HUMAN Probable helicase senataxin OS=Homo sapiens GN=SETX PE=1 SV=4
evm.model.scaffold129895.10	10.0616	17.9162	-0.8324	0.00271512	sp|Q4U2R1|HERC2_MOUSE E3 ubiquitin-protein ligase HERC2 OS=Mus musculus GN=Herc2 PE=1 SV=3
evm.model.scaffold73127.74	21.0795	36.8313	-0.805092	0.020248	--
evm.model.scaffold45699.1	5.21787	10.5009	-1.00898	0.000820679	sp|A4IF63|TRIM2_BOVIN Tripartite motif-containing protein 2 OS=Bos taurus GN=TRIM2 PE=2 SV=1

evm.model.scaffold107039.4	22.1231	41.5209	-0.908288	0.000820679	sp|Q8WY64|MYLIP_HUMAN E3 ubiquitin-protein ligase MYLIP OS=Homo sapiens GN=MYLIP PE=1 SV=2
evm.model.scaffold11673.5	0.45664	1.83283	-2.00494	0.0191834	--
evm.model.scaffold23155.13_evm.model.scaffold23155.12	32.2445	54.0582	-0.745461	0.00621905	sp|Q96QK1|VPS35_HUMAN Vacuolar protein sorting-associated protein 35 OS=Homo sapiens GN=VPS35 PE=1 SV=2
evm.model.scaffold40687.5	22.9912	76.2006	-1.72872	0.000820679	sp|Q3MI05|PPGB_BOVIN Lysosomal protective protein OS=Bos taurus GN=CTSA PE=2 SV=1
evm.model.scaffold69023.3	48.7883	93.9214	-0.944919	0.000820679	sp|Q9W2N0|CAPZA_DROME F-actin-capping protein subunit alpha OS=Drosophila melanogaster GN=cpa PE=2 SV=1
evm.model.scaffold173383.37	35.0288	59.7382	-0.770111	0.00670086	sp|Q14140|SRTD2_HUMAN SERTA domain-containing protein 2 OS=Homo sapiens GN=SERTAD2 PE=1 SV=1
evm.model.scaffold52831.27	10.4482	16.3816	-0.648823	0.0284609	sp|Q8BGG7|UBS3B_MOUSE Ubiquitin-associated and SH3 domain-containing protein B OS=Mus musculus GN=Ubash3b PE=1 SV=1
evm.model.scaffold100379.5	5.73922	18.5794	-1.69478	0.000820679	sp|Q920A5|RISC_MOUSE

					Retinoid-inducible serine carboxypeptidase OS=Mus musculus GN=Scpep1 PE=2 SV=2
evm.model.scaffold89149.1	2.81644	6.81344	-1.27451	0.000820679	--
evm.model.scaffold90899.75_evm.model.scaffold90899.74	2.4211	5.06666	-1.06537	0.000820679	sp|P55013|S12A2_SQUAC Solute carrier family 12 member 2 OS=Squalus acanthias GN=SLC12A2 PE=1 SV=1
evm.model.scaffold100379.68	14.2153	22.8758	-0.686382	0.0457321	sp|C4A0P0|CID2B_BRAFL CDGSH iron-sulfur domain-containing protein 2 homolog B OS=Branchiostoma floridae GN=BRAFLDRAFT_274541 PE=3 SV=1
evm.model.scaffold79079.14	7.3039	12.8542	-0.815502	0.0180929	sp|P05099|MATN1_CHICK Cartilage matrix protein OS=Gallus gallus GN=MATN1 PE=1 SV=2
evm.model.scaffold47331.7	22.609	45.9697	-1.02378	0.000820679	sp|P34121|COAA_DICDI Coactosin OS=Dictyostelium discoideum GN=coaA PE=1 SV=1
evm.model.scaffold7425.16	0.12045	0.572616	-2.24913	0.0347943	sp|E1BD59|TRI56_BOVIN E3 ubiquitin-protein ligase TRIM56 OS=Bos taurus GN=TRIM56 PE=3 SV=1
evm.model.scaffold136117.24	26.7316	42.8886	-0.682048	0.0228478	sp|Q5ZIK9|COPE_CHICK Coatomer subunit epsilon OS=Gallus gallus GN=COPE PE=2 SV=1

evm.model.scaffold150821.87	9.10996	18.566	-1.02714	0.000820679	sp|Q922R8|PDIA6_MOUSE Protein disulfide-isomerase A6 OS=Mus musculus GN=Pdia6 PE=1 SV=3
evm.model.scaffold67097.34	11.2276	21.3196	-0.925133	0.000820679	sp|Q5FWK3|RHG01_MOUSE Rho GTPase-activating protein 1 OS=Mus musculus GN=Arhgap1 PE=1 SV=1
evm.model.scaffold49049.21	261.106	494.617	-0.921678	0.00842602	sp|P28648|CD63_RAT CD63 antigen OS=Rattus norvegicus GN=Cd63 PE=1 SV=2
evm.model.scaffold27973.83	0.584949	1.89765	-1.69783	0.0205228	--
evm.model.scaffold124841.17	125.098	196.944	-0.65472	0.0248731	sp|Q92747|ARC1A_HUMAN Actin-related protein 2/3 complex subunit 1A OS=Homo sapiens GN=ARPC1A PE=1 SV=2
evm.model.scaffold124841.18	14.2248	30.5167	-1.10119	0.000820679	sp|Q2L6K8|CNPY4_DANRE Protein canopy 4 OS=Danio rerio GN=cnpy4 PE=2 SV=1
evm.model.scaffold77239.11	0.913631	2.2812	-1.32011	0.00712329	sp|Q9BRZ2|TRI56_HUMAN E3 ubiquitin-protein ligase TRIM56 OS=Homo sapiens GN=TRIM56 PE=1 SV=3
evm.model.scaffold98447.43	13.6671	24.7533	-0.856915	0.00480132	sp|Q5BK46|RNH1_RAT Ribonuclease H1 OS=Rattus norvegicus GN=Rnaseh1 PE=2 SV=1
evm.model.scaffold14517.19	39.1005	89.5579	-1.19563	0.000820679	sp|P24367|PPIB_CHICK

					Peptidyl-prolyl cis-trans isomerase B OS=Gallus gallus GN=PPIB PE=2 SV=1
evm.model.scaffold14517.10	4.77639	13.3112	-1.47865	0.000820679	sp|P70031|CCKAR_XENLA Cholecystokinin receptor OS=Xenopus laevis GN=cckar PE=2 SV=1
evm.model.scaffold150767.5	5.27526	14.662	-1.47477	0.000820679	sp|C0HBB2|GLMP_SALSA Glycosylated lysosomal membrane protein OS=Salmo salar GN=glmp PE=2 SV=1
evm.model.scaffold93729.61	97.9298	181.91	-0.893403	0.00151774	sp|Q98SV0|SELPB_DANRE Selenoprotein Pb OS=Danio rerio GN=sepp1b PE=2 SV=3
evm.model.scaffold105705.5	7.69486	21.2472	-1.4653	0.000820679	sp|Q9DC37|MFSD1_MOUSE Major facilitator superfamily domain-containing protein 1 OS=Mus musculus GN=Mfsd1 PE=1 SV=1
evm.model.scaffold86385.3	1.21315	12.184	-3.32816	0.000820679	sp|Q9JMA7|CP341_MOUSE Cytochrome P450 3A41 OS=Mus musculus GN=Cyp3a41a PE=2 SV=2
evm.model.scaffold89799.1	2.18775	4.48462	-1.03554	0.00842602	sp|Q17938|DAF36_CAEEL Cholesterol desaturase daf-36 OS=Caenorhabditis elegans GN=daf-36 PE=1 SV=2
evm.model.scaffold169005.10	12.3685	20.7623	-0.747292	0.0284609	--
evm.model.scaffold146109.2	15.5523	36.2395	-1.22044	0.000820679	sp|Q75WF2|DNA2_ACAPL

					Plancitoxin-1 OS=Acanthaster planci PE=1 SV=1
evm.model.scaffold126893.1	0.391014	1.6341	-2.0632	0.00213042	sp|Q0PV50|TLR3_BOSTR Toll-like receptor 3 OS=Boselaphus tragocamelus GN=TLR3 PE=2 SV=1
evm.model.scaffold134743.59	4.56717	15.8625	-1.79625	0.000820679	sp|Q2KIR8|TDH_BOVIN L-threonine 3-dehydrogenase, mitochondrial OS=Bos taurus GN=TDH PE=2 SV=1
evm.model.scaffold134891.14	17.5869	65.4544	-1.89599	0.00621905	sp|Q78P75|DYL2_RAT Dynein light chain 2, cytoplasmic OS=Rattus norvegicus GN=Dynll2 PE=1 SV=1
evm.model.scaffold134891.10	39.8193	70.2618	-0.819272	0.00429787	sp|Q78P75|DYL2_RAT Dynein light chain 2, cytoplasmic OS=Rattus norvegicus GN=Dynll2 PE=1 SV=1
evm.model.scaffold95939.5_evm.model.scaffold95939.6	22.3031	40.3297	-0.854596	0.00429787	sp|Q5RBM1|RPN2_PONAB Dolichyl-diphosphooligosaccharide--protein glycosyltransferase subunit 2 OS=Pongo abelii GN=RPN2 PE=2 SV=1
evm.model.scaffold87549.17	4.7877	28.9702	-2.59716	0.000820679	sp|O35548|MMP16_RAT Matrix metalloproteinase-16 OS=Rattus norvegicus GN=Mmp16 PE=2 SV=1
evm.model.scaffold90899.23.1	7.87775	51.1425	-2.69867	0.000820679	sp|Q7ZYC4|ACBG2_XENLA Long-chain-fatty-acid--CoA ligase ACSBG2 OS=Xenopus laevis GN=acsbg2 PE=2 SV=1

evm.model.scaffold136585.6	6.84374	69.2804	-3.33959	0.000820679	sp|O34363|YOBN_BACSU Putative L-amino-acid oxidase YobN OS=Bacillus subtilis (strain 168) GN=yobN PE=3 SV=3
evm.model.scaffold146155.3	5.47774	34.4085	-2.65111	0.000820679	sp|Q7Z5S9|TM144_HUMAN Transmembrane protein 144 OS=Homo sapiens GN=TMEM144 PE=2 SV=1
evm.model.scaffold35963.7	2.81241	4.73481	-0.7515	0.0276479	sp|P16157|ANK1_HUMAN Ankyrin-1 OS=Homo sapiens GN=ANK1 PE=1 SV=3
evm.model.scaffold169965.12	6.15742	16.9223	-1.45853	0.00213042	--
evm.model.scaffold169965.19	5.37811	9.31109	-0.791852	0.0248731	sp|Q9UK39|NOCT_HUMAN Nocturnin OS=Homo sapiens GN=CCRN4L PE=2 SV=2
evm.model.scaffold147331.13	1.32185	3.03228	-1.19784	0.0302156	sp|A0PJN4|U2QL1_MOUSE Ubiquitin-conjugating enzyme E2Q-like protein 1 OS=Mus musculus GN=Ube2ql1 PE=2 SV=2
evm.model.scaffold53733.20	2.21899	5.81771	-1.39055	0.000820679	sp|A1L2F6|TM205_DANRE Transmembrane protein 205 OS=Danio rerio GN=tmem205 PE=2 SV=1
evm.model.scaffold124143.1	8.67739	19.1387	-1.14116	0.0208422	sp|Q3TLP5|ECHD2_MOUSE Enoyl-CoA hydratase domain-containing protein 2, mitochondrial OS=Mus musculus

					GN=Echdc2 PE=1 SV=2
evm.model.scaffold124143.7	62.1632	276.293	-2.15206	0.00213042	--
evm.model.scaffold133351.15	2.76744	10.0333	-1.85817	0.000820679	sp|Q9NRA2|S17A5_HUMAN Sialin OS=Homo sapiens GN=SLC17A5 PE=1 SV=2
evm.model.scaffold84915.44	1.62412	3.88425	-1.25798	0.000820679	--
evm.model.scaffold84915.49	2.05093	3.64637	-0.830184	0.0109379	sp|Q9HCF6|TRPM3_HUMAN Transient receptor potential cation channel subfamily M member 3 OS=Homo sapiens GN=TRPM3 PE=2 SV=4
evm.model.scaffold152681.9	15.5492	39.4421	-1.3429	0.000820679	--
evm.model.scaffold167477.85	1.12392	3.64284	-1.69653	0.000820679	sp|Q9DD78|TLR21_CHICK Toll-like receptor 2 type-1 OS=Gallus gallus GN=TLR2-1 PE=2 SV=1
evm.model.scaffold39493.4	1.65177	4.04445	-1.29194	0.000820679	sp|P18433|PTPRA_HUMAN Receptor-type tyrosine-protein phosphatase alpha OS=Homo sapiens GN=PTPRA PE=1 SV=2
evm.model.scaffold66661.70	2.1854	5.00826	-1.19641	0.0169923	sp|Q32KH5|GALNS_CANFA N-acetylgalactosamine-6-sulfatase OS=Canis familiaris GN=GALNS PE=2 SV=1
evm.model.scaffold71897.39	2.6133	9.32154	-1.83469	0.000820679	sp|Q9VT65|CANB_DROME Calpain-B OS=Drosophila

					melanogaster GN=CalpB PE=1 SV=2
evm.model.scaffold141823.10	1.91934	17.839	-3.21636	0.000820679	--
evm.model.scaffold159893.33	0.121579	1.38475	-3.50966	0.00755259	sp|P37064|ASO_CUCPM L-ascorbate oxidase OS=Cucurbita pepo var. melopepo PE=1 SV=1
evm.model.scaffold124907.16	4.74773	9.63508	-1.02106	0.000820679	sp|O13356|CHS1_CRYNH Chitin synthase 1 OS=Cryptococcus neoformans var. grubii serotype A (strain H99 / ATCC 208821 / CBS 10515 / FGSC 9487) GN=CHS1 PE=3 SV=2
evm.model.scaffold124907.17	4.78163	9.49584	-0.989792	0.0129481	sp|Q92625|ANS1A_HUMAN Ankyrin repeat and SAM domain-containing protein 1A OS=Homo sapiens GN=ANKS1A PE=1 SV=4
evm.model.scaffold82159.20	31.7687	56.739	-0.836737	0.00326205	sp|Q9N1Q0|RSMB_MACEU Small nuclear ribonucleoprotein-associated protein B' OS=Macropus eugenii GN=SNRPB PE=2 SV=1
evm.model.scaffold168171.24	0.395659	1.22963	-1.63589	0.000820679	sp|Q8R151|ZNFX1_MOUSE NFX1-type zinc finger-containing protein 1 OS=Mus musculus GN=Znfx1 PE=2 SV=3
evm.model.scaffold168171.25	4.38466	7.46191	-0.767081	0.0121758	sp|Q9P2E3|ZNFX1_HUMAN NFX1-type zinc finger-containing

					protein 1 OS=Homo sapiens GN=ZNFX1 PE=2 SV=2
evm.model.scaffold168171.22	2.58593	4.431	-0.77695	0.0156116	sp|Q8R151|ZNFX1_MOUSE NFX1-type zinc finger-containing protein 1 OS=Mus musculus GN=Znfx1 PE=2 SV=3
evm.model.scaffold168355.17	638.421	1036.22	-0.698746	0.0284609	sp|P42577|FRIS_LYMST Soma ferritin OS=Lymnaea stagnalis PE=2 SV=2
evm.model.scaffold8273.8	0.520782	2.06602	-1.9881	0.000820679	sp|Q90705|EF2_CHICK Elongation factor 2 OS=Gallus gallus GN=EEF2 PE=1 SV=3
evm.model.scaffold162457.10	10.3123	18.0792	-0.809968	0.014476	--
evm.model.scaffold150525.5	24.7527	42.4385	-0.777783	0.00480132	sp|Q80X72|LRC15_MOUSE Leucine-rich repeat-containing protein 15 OS=Mus musculus GN=Lrrc15 PE=2 SV=1
evm.model.scaffold150525.6	3.8091	11.5172	-1.59627	0.00151774	sp|Q498D6|FGFR4_RAT Fibroblast growth factor receptor 4 OS=Rattus norvegicus GN=Fgfr4 PE=1 SV=1
evm.model.scaffold176119.88	0.638614	5.02496	-2.9761	0.000820679	sp|Q3ZBG9|PLS2_BOVIN Phospholipid scramblase 2 OS=Bos taurus GN=PLSCR2 PE=2 SV=1
evm.model.scaffold74759.2	25.1813	43.1471	-0.776912	0.00271512	sp|F1N9Y5|KSYK_CHICK Tyrosine-protein kinase SYK OS=Gallus gallus GN=SYK PE=1 SV=2

evm.model.scaffold65899.1	0.310972	2.11159	-2.76347	0.00972356	sp|Q58EX2|SDK2_HUMAN Protein sidekick-2 OS=Homo sapiens GN=SDK2 PE=1 SV=3
evm.model.scaffold160103.12	2.62459	4.61071	-0.812897	0.0396592	sp|Q91WU4|TMCO4_MOUSE Transmembrane and coiled-coil domain-containing protein 4 OS=Mus musculus GN=Tmco4 PE=2 SV=2
evm.model.scaffold163411.6	12.5111	24.2792	-0.956514	0.000820679	sp|P07898|PGCA_CHICK Aggrecan core protein OS=Gallus gallus GN=ACAN PE=1 SV=2
evm.model.scaffold163411.5	31.0908	47.7664	-0.619507	0.0302156	sp|O00291|HIP1_HUMAN Huntingtin-interacting protein 1 OS=Homo sapiens GN=HIP1 PE=1 SV=5
evm.model.scaffold163895.4.1	1.51149	4.26418	-1.4963	0.000820679	sp|P22105|TENX_HUMAN Tenascin-X OS=Homo sapiens GN=TNXB PE=1 SV=4
evm.model.scaffold151227.10	11.1906	35.1767	-1.65233	0.000820679	sp|O88958|GNPI1_MOUSE Glucosamine-6-phosphate isomerase 1 OS=Mus musculus GN=Gnpda1 PE=2 SV=3
evm.model.scaffold172609.6	4.45523	14.9499	-1.74656	0.000820679	sp|Q06367|CP1A1_CAVPO Cytochrome P450 1A1 OS=Cavia porcellus GN=CYP1A1 PE=1 SV=1
evm.model.scaffold176323.15	11.6029	18.9614	-0.708588	0.0159979	sp|Q9Z1Z3|EPN2_RAT Epsin-2

					OS=Rattus norvegicus GN=Epn2 PE=1 SV=1
evm.model.scaffold84953.19	18.0488	39.9245	-1.14538	0.000820679	sp|Q9W4P5|VA0D1_DROME V-type proton ATPase subunit d 1 OS=Drosophila melanogaster GN=VhaAC39-1 PE=2 SV=1
evm.model.scaffold166837.49	26.1041	50.6529	-0.956369	0.000820679	sp|Q9ESS0|DUS10_MOUSE Dual specificity protein phosphatase 10 OS=Mus musculus GN=Dusp10 PE=2 SV=2
evm.model.scaffold74183.6	2.80822	6.38466	-1.18495	0.00213042	--
evm.model.scaffold74183.3	4.15148	10.1074	-1.28372	0.0299214	--
evm.model.scaffold73013.7	1.19277	2.54502	-1.09336	0.00712329	sp|Q7G192|ALDO2_ARATH Indole-3-acetaldehyde oxidase OS=Arabidopsis thaliana GN=AAO2 PE=1 SV=2
evm.model.scaffold50277.2	1.46349	3.31374	-1.17905	0.0276479	sp|Q9JHX4|CASP8_RAT Caspase-8 OS=Rattus norvegicus GN=Casp8 PE=1 SV=1
evm.model.scaffold142101.2	3.01312	5.48611	-0.864526	0.00429787	sp|Q460N5|PAR14_HUMAN Poly [ADP-ribose] polymerase 14 OS=Homo sapiens GN=PARP14 PE=1 SV=3
evm.model.scaffold7723.1_evm.model.scaffold7723.2	3.40031	8.49657	-1.32121	0.000820679	sp|F1QN74|ZMY10_DANRE Zinc finger MYND domain-containing protein 10 OS=Danio rerio

					GN=zmynd10 PE=2 SV=1
evm.model.scaffold140631.24	2.44093	8.31304	-1.76795	0.0251677	--
evm.model.scaffold104669.15	1.10296	5.59514	-2.3428	0.000820679	sp|Q9R0G6|COMP_MOUSE Cartilage oligomeric matrix protein OS=Mus musculus GN=Comp PE=1 SV=2
evm.model.scaffold109527.29	59.5254	95.3006	-0.678979	0.0191834	sp|Q08874|MITF_MOUSE Microphthalmia-associated transcription factor OS=Mus musculus GN=Mitf PE=1 SV=3
evm.model.scaffold41199.13	33.0409	76.8191	-1.21721	0.000820679	sp|Q5BJU6|CRBL2_RAT cAMP-responsive element-binding protein-like 2 OS=Rattus norvegicus GN=Crebl2 PE=2 SV=1
evm.model.scaffold41199.14	2.81046	6.67011	-1.2469	0.00271512	--
evm.model.scaffold164773.46	0.396535	3.32663	-3.06854	0.0231466	--
evm.model.scaffold164773.47	4.68931	18.509	-1.98078	0.000820679	--
evm.model.scaffold164773.44	0.330224	6.04242	-4.19361	0.000820679	--
evm.model.scaffold164773.45	0.314152	5.98203	-4.2511	0.000820679	--
evm.model.scaffold164773.42	1.13856	3.57561	-1.65098	0.0228478	--
evm.model.scaffold176535.5	0.526843	1.34514	-1.35231	0.048902	sp|A6NDV4|TMM8B_HUMAN Transmembrane protein 8B OS=Homo sapiens GN=TMEM8B PE=1 SV=2
evm.model.scaffold176535.3	2.10941	5.25751	-1.31754	0.0109379	--
evm.model.scaffold138489.42	9.61321	16.7304	-0.799383	0.00480132	sp|Q8CJH6|NPAS4_RAT Neuronal PAS domain-containing protein 4

					OS=Rattus norvegicus GN=Npas4 PE=2 SV=1
evm.model.scaffold69023.18	2.67222	4.68056	-0.808641	0.0173453	sp|P10079|FBP1_STRPU Fibropellin-1 OS=Strongylocentrotus purpuratus GN=EGF1 PE=1 SV=2
evm.model.scaffold2487.6	18.713	49.12	-1.39227	0.000820679	sp|Q8NDA2|HMCN2_HUMAN Hemicentin-2 OS=Homo sapiens GN=HMCN2 PE=2 SV=2
evm.model.scaffold2487.7	1.86032	6.13512	-1.72154	0.00151774	sp|F7J220|SRCR1_PATPE Scavenger receptor cysteine-rich domain superfamily protein OS=Patiria pectinifera GN=SRCR1 PE=1 SV=1
evm.model.scaffold4919.15	1.85087	5.67647	-1.61679	0.000820679	sp|O35744|CHIL3_MOUSE Chitinase-like protein 3 OS=Mus musculus GN=Chil3 PE=1 SV=2
evm.model.scaffold92151.27	2.60719	5.32828	-1.03118	0.00213042	sp|P35289|RAB15_RAT Ras-related protein Rab-15 OS=Rattus norvegicus GN=Rab15 PE=2 SV=1
evm.model.scaffold90371.13	32.1427	123.207	-1.93852	0.000820679	--
evm.model.scaffold90371.15	1.20011	4.68704	-1.96551	0.000820679	--
evm.model.scaffold2393.4	0.881827	3.33694	-1.91996	0.00670086	sp|O00338|ST1C2_HUMAN Sulfotransferase 1C2 OS=Homo sapiens GN=SULT1C2 PE=1 SV=1
evm.model.scaffold162341.4	2.74219	8.81245	-1.68421	0.000820679	sp|Q24940|CATLL_FASHE Cathepsin L-like proteinase OS=Fasciola hepatica

					GN=Cat-1 PE=1 SV=1
evm.model.scaffold170799.1	0	0.645931	#NAME?	0.000820679	sp|Q9VUL9|FUCTA_DROME Glycoprotein 3-alpha-L-fucosyltransferase A OS=Drosophila melanogaster GN=FucTA PE=2 SV=2
evm.model.scaffold107255.13	1.30862	4.13916	-1.66129	0.000820679	sp|B5X3C1|GALC_SALSA Galactocerebrosidase OS=Salmo salar GN=galc PE=2 SV=1
evm.model.scaffold86191.22	5.8408	18.8779	-1.69246	0.000820679	--
evm.model.scaffold86191.23	0.760107	1.74822	-1.20161	0.027885	sp|Q8R0M8|MOT5_MOUSE Monocarboxylate transporter 5 OS=Mus musculus GN=Slc16a4 PE=2 SV=1
evm.model.scaffold151467.138	17.6496	28.7887	-0.705864	0.0163478	sp|Q8N8U2|CDYL2_HUMAN Chromodomain Y-like protein 2 OS=Homo sapiens GN=CDYL2 PE=1 SV=2
evm.model.scaffold127037.5	0.397644	1.35175	-1.76528	0.0163478	sp|D9IQ16|GXN_ACRMI Galaxin OS=Acropora millepora PE=1 SV=1
evm.model.scaffold127037.2	0.335381	2.444	-2.86538	0.000820679	sp|D9IQ16|GXN_ACRMI Galaxin OS=Acropora millepora PE=1 SV=1
evm.model.scaffold80273.11	0.749649	2.36306	-1.65637	0.0109379	sp|Q8K337|I5P2_MOUSE Type II inositol 1,4,5-trisphosphate 5-phosphatase OS=Mus musculus

					GN=Inpp5b PE=1 SV=1
evm.model.scaffold146097.8	17.5464	41.3068	-1.2352	0.000820679	sp|Q66I21|AL8A1_DANRE Aldehyde dehydrogenase family 8 member A1 OS=Danio rerio GN=aldh8a1 PE=2 SV=1
evm.model.scaffold92151.19.1	1.88746	3.44059	-0.866211	0.0418385	sp|O95861|BPNT1_HUMAN 3'(2'),5'-bisphosphate nucleotidase 1 OS=Homo sapiens GN=BPNT1 PE=1 SV=1
evm.model.scaffold99021.9	10.1744	19.2223	-0.917833	0.00271512	sp|Q13094|LCP2_HUMAN Lymphocyte cytosolic protein 2 OS=Homo sapiens GN=LCP2 PE=1 SV=1
evm.model.scaffold174851.7	309.307	734.358	-1.24744	0.00380231	sp|Q2KJG3|SYNC_BOVIN Asparagine--tRNA ligase, cytoplasmic OS=Bos taurus GN=NARS PE=2 SV=3
evm.model.scaffold174851.5	32.6097	124.64	-1.9344	0.000820679	sp|A0JNU3|LPP60_MOUSE 60 kDa lysophospholipase OS=Mus musculus GN=Aspg PE=2 SV=1
evm.model.scaffold174851.2	0.669356	1.61164	-1.26768	0.0109379	--
evm.model.scaffold153897.1	14.3525	27.7098	-0.949097	0.00271512	sp|Q7T2P0|MX1_ICTPU Interferon-induced GTP-binding protein Mx1 OS=Ictalurus punctatus GN=mx1 PE=2 SV=1
evm.model.scaffold57245.2	8.71135	24.1391	-1.47041	0.000820679	--

evm.model.scaffold75453.17	4.44165	7.30009	-0.716818	0.0218619	sp|Q8NFZ0|FBX18_HUMAN F-box only protein 18 OS=Homo sapiens GN=FBXO18 PE=1 SV=2
evm.model.scaffold136165.2	41.5956	95.924	-1.20546	0.000820679	sp|P27447|YES_XIPHE Tyrosine-protein kinase Yes OS=Xiphophorus helleri GN=yes PE=2 SV=3
evm.model.scaffold142253.22	32.5409	50.3352	-0.629314	0.0316212	sp|Q86GC8|ACES_CULPI Acetylcholinesterase OS=Culex pipiens GN=ACHE1 PE=2 SV=2
evm.model.scaffold172729.1	7.14979	17.0429	-1.2532	0.000820679	sp|Q4H3K6|FGFR_CIOIN Fibroblast growth factor receptor OS=Ciona intestinalis GN=FGFR PE=2 SV=1
evm.model.scaffold119639.1	4.64555	8.254	-0.829246	0.013343	sp|Q9UBH6|XPR1_HUMAN Xenotropic and polytropic retrovirus receptor 1 OS=Homo sapiens GN=XPR1 PE=1 SV=1
evm.model.scaffold92151.1	5.07606	8.7561	-0.78658	0.00532397	sp|Q6ZQ12|NINL_MOUSE Ninein-like protein OS=Mus musculus GN=Ninl PE=2 SV=3
evm.model.scaffold14517.17	0.785815	2.05098	-1.38405	0.0308116	--
evm.model.scaffold128325.27.1	0.34663	1.52934	-2.14144	0.0137473	--
evm.model.scaffold21299.9	18.8699	40.7162	-1.10951	0.000820679	sp|O35235|TNF11_MOUSE Tumor necrosis factor ligand superfamily member 11 OS=Mus musculus

					GN=Tnfsf11 PE=1 SV=2
evm.model.scaffold21299.2	1.79771	3.91063	-1.12125	0.00755259	sp|Q6GM59|MOT12_XENLA Monocarboxylate transporter 12 OS=Xenopus laevis GN=slc16a12 PE=2 SV=1
evm.model.scaffold21299.5	0.229375	3.8117	-4.05465	0.00151774	sp|Q5UBV8|TNF15_MOUSE Tumor necrosis factor ligand superfamily member 15 OS=Mus musculus GN=Tnfsf15 PE=2 SV=2
evm.model.scaffold174377.3	5.11748	9.83608	-0.94265	0.000820679	sp|Q9DD78|TLR21_CHICK Toll-like receptor 2 type-1 OS=Gallus gallus GN=TLR2-1 PE=2 SV=1
evm.model.scaffold66257.8	1.18981	2.64317	-1.15154	0.00213042	sp|Q8QFV0|KCNT1_CHICK Potassium channel subfamily T member 1 OS=Gallus gallus GN=KCNT1 PE=2 SV=1
evm.model.scaffold160813.29	5.34641	9.42046	-0.817227	0.0251677	sp|P83425|HIP_MYTED Heavy metal-binding protein HIP OS=Mytilus edulis PE=1 SV=1
evm.model.scaffold116119.93	65.3904	189.162	-1.53247	0.000820679	sp|P61917|NPC2_PANTR Epididymal secretory protein E1 OS=Pan troglodytes GN=NPC2 PE=2 SV=1
evm.model.scaffold142101.104	35.868	54.9176	-0.614573	0.040273	sp|Q8MSU3|FRRS1_DROME Putative ferric-chelate reductase 1 homolog OS=Drosophila melanogaster

					GN=CG8399 PE=2 SV=1
evm.model.scaffold53733.35.1	8.44104	13.5034	-0.677829	0.0388548	sp|P11627|L1CAM_MOUSE Neural cell adhesion molecule L1 OS=Mus musculus GN=L1cam PE=1 SV=1
evm.model.scaffold128461.13	6.14251	12.7205	-1.05026	0.0109379	sp|Q8BUM3|PTN7_MOUSE Tyrosine-protein phosphatase non-receptor type 7 OS=Mus musculus GN=Ptpn7 PE=1 SV=1
evm.model.scaffold144587.1	1.07528	3.36228	-1.64473	0.000820679	sp|P97685|NFASC_RAT Neurofascin OS=Rattus norvegicus GN=Nfasc PE=1 SV=2
evm.model.scaffold5677.4	21.5216	51.8605	-1.26885	0.000820679	sp|Q7SXW6|ARP2A_DANRE Actin-related protein 2-A OS=Danio rerio GN=actr2a PE=2 SV=1
evm.model.scaffold176621.9	1.70631	3.43901	-1.01111	0.0199213	sp|Q8SPN1|PKR2_BOVIN Prokineticin receptor 2 OS=Bos taurus GN=PROKR2 PE=2 SV=1
evm.model.scaffold140451.9	3.94188	7.22162	-0.873439	0.00429787	sp|Q3UR85|MRF_MOUSE Myelin regulatory factor OS=Mus musculus GN=Myrf PE=1 SV=2
evm.model.scaffold138629.172	11.9754	32.4062	-1.43619	0.000820679	sp|O61363|HCYG_ENTDO Hemocyanin G-type, units Oda to Odg OS=Enteroctopus dofleini GN=ODHCY PE=1 SV=1
evm.model.scaffold86385.1	4.21771	21.8713	-2.37451	0.000820679	sp|P33268|CP3A8_MACFA

					Cytochrome P450 3A8 OS=Macaca fascicularis GN=CYP3A8 PE=1 SV=1
evm.model.scaffold142971.86	102.519	189.877	-0.889181	0.00213042	sp|Q95218|DMBT1_RABIT Deleted in malignant brain tumors 1 protein OS=Oryctolagus cuniculus GN=Dmbt1 PE=1 SV=2
evm.model.scaffold100379.15	51.5111	190.929	-1.89008	0.000820679	sp|O88582|SOCS2_RAT Suppressor of cytokine signaling 2 OS=Rattus norvegicus GN=Socs2 PE=2 SV=1
evm.model.scaffold100379.10	17.1724	51.485	-1.58406	0.000820679	sp|Q2KJC3|MPEG1_BOVIN Macrophage-expressed gene 1 protein OS=Bos taurus GN=MPEG1 PE=2 SV=2
evm.model.scaffold133351.4	0.0677293	0.662683	-3.29047	0.0163478	sp|Q9VN14|CONT_DROME Contactin OS=Drosophila melanogaster GN=Cont PE=1 SV=2
evm.model.scaffold154441.41	2.63131	6.36863	-1.2752	0.000820679	sp|Q99571|P2RX4_HUMAN P2X purinoceptor 4 OS=Homo sapiens GN=P2RX4 PE=1 SV=2
evm.model.scaffold138569.81	4.05179	14.1776	-1.80698	0.000820679	sp|Q5XGP7|SKA2A_XENLA Src kinase-associated phosphoprotein 2-A OS=Xenopus laevis GN=skap2-a PE=2 SV=1
evm.model.scaffold140611.1	25.2397	40.9145	-0.696914	0.023485	sp|O73817|PSB3_ONCMY Proteasome subunit beta type-3 OS=Oncorhynchus mykiss GN=psmb3

					PE=2 SV=1
evm.model.scaffold155263.5	0	0.960818	#NAME?	0.000820679	--
evm.model.scaffold172313.12	10.8412	38.893	-1.84299	0.000820679	sp|Q58A42|DD3_DICDI Protein DD3-3 OS=Dictyostelium discoideum GN=DD3-3 PE=2 SV=1
evm.model.scaffold172313.10	4.29254	8.83308	-1.04108	0.00151774	sp|A4IGF3|ATP23_DANRE Mitochondrial inner membrane protease ATP23 homolog OS=Danio rerio GN=zgc:162885 PE=2 SV=1
evm.model.scaffold169587.8	2.01609	4.46872	-1.1483	0.0105331	sp|Q4R6N0|TPTE2_MACFA Phosphatidylinositol 3,4,5-trisphosphate 3-phosphatase TPTE2 OS=Macaca fascicularis GN=TPTE2 PE=2 SV=1
evm.model.scaffold163753.15	84.982	129.603	-0.60887	0.0296885	sp|Q9JLS3|TAOK2_RAT Serine/threonine-protein kinase TAO2 OS=Rattus norvegicus GN=Taok2 PE=1 SV=1
evm.model.scaffold43171.2	12.4907	19.7576	-0.66155	0.0282044	sp|Q8NI37|PPTC7_HUMAN Protein phosphatase PTC7 homolog OS=Homo sapiens GN=PPTC7 PE=2 SV=1
evm.model.scaffold167793.10_evm.model.scaffold167793.9	0.603875	1.70485	-1.49733	0.0205228	sp|Q5U2V4|PLBL1_RAT Phospholipase B-like 1 OS=Rattus norvegicus GN=Plbd1 PE=2 SV=1
evm.model.scaffold176119.54	15.2691	27.0879	-0.827038	0.00429787	sp|Q63HN8|RN213_HUMAN E3

					ubiquitin-protein ligase RNF213 OS=Homo sapiens GN=RNF213 PE=1 SV=3
evm.model.scaffold34455.25	66.2942	129.996	-0.971511	0.000820679	sp|O75581|LRP6_HUMAN Low-density lipoprotein receptor-related protein 6 OS=Homo sapiens GN=LRP6 PE=1 SV=2
evm.model.scaffold34455.27	16.8283	41.7384	-1.31049	0.000820679	sp|P98157|LRP1_CHICK Low-density lipoprotein receptor-related protein 1 OS=Gallus gallus GN=LRP1 PE=2 SV=1
evm.model.scaffold66879.16	5.98877	9.68407	-0.693355	0.0264598	sp|Q80TA9|EPG5_MOUSE Ectopic P granules protein 5 homolog OS=Mus musculus GN=Epg5 PE=2 SV=2
evm.model.scaffold176119.105	4.27015	8.13741	-0.930282	0.00670086	sp|Q08DY9|CASP3_BOVIN Caspase-3 OS=Bos taurus GN=CASP3 PE=2 SV=1
evm.model.scaffold18883.11	3.2043	5.95126	-0.893185	0.0101381	sp|Q5ZIJ9|MIB2_CHICK E3 ubiquitin-protein ligase MIB2 OS=Gallus gallus GN=MIB2 PE=2 SV=1
evm.model.scaffold80479.4	0	2.906	#NAME?	0.0373071	sp|Q4R532|SYNG1_MACFA Synapse differentiation-inducing gene protein 1 OS=Macaca fascicularis GN=SYNDIG1 PE=2 SV=1
evm.model.scaffold153701.26	3.07844	5.46802	-0.828818	0.0184892	sp|Q9UKK3|PARP4_HUMAN Poly [ADP-ribose] polymerase 4 OS=Homo

					sapiens GN=PARP4 PE=1 SV=3
evm.model.scaffold118407.4	1.24756	2.45336	-0.975653	0.00972356	sp|Q5F478|ANR44_CHICK Serine/threonine-protein phosphatase 6 regulatory ankyrin repeat subunit B OS=Gallus gallus GN=ANKRD44 PE=2 SV=1
evm.model.scaffold118407.5	1.38475	4.5667	-1.72153	0.000820679	--
evm.model.scaffold112961.5_evm.model.scaffold112961.7	6.76732	20.1968	-1.57747	0.000820679	sp|Q91WM2|CECR5_MOUSE Cat eye syndrome critical region protein 5 homolog OS=Mus musculus GN=Cecr5 PE=2 SV=1
evm.model.scaffold49089.6	5.50406	10.8608	-0.980558	0.0148208	sp|Q8CIQ7|DOCK3_MOUSE Dedicator of cytokinesis protein 3 OS=Mus musculus GN=Dock3 PE=1 SV=1
evm.model.scaffold99537.1	2.20802	4.99525	-1.1778	0.00326205	sp|Q5I2M4|TLR9_SHEEP Toll-like receptor 9 OS=Ovis aries GN=TLR9 PE=2 SV=1
evm.model.scaffold142101.94	2.40882	5.34463	-1.14977	0.000820679	sp|P50429|ARSB_MOUSE Arylsulfatase B OS=Mus musculus GN=Arsb PE=2 SV=3
evm.model.scaffold73127.20	33.9099	61.4439	-0.857563	0.000820679	sp|O75581|LRP6_HUMAN Low-density lipoprotein receptor-related protein 6 OS=Homo sapiens GN=LRP6 PE=1 SV=2

evm.model.scaffold7467.31	5.56871	10.2656	-0.882404	0.00213042	sp|Q8C8R3|ANK2_MOUSE Ankyrin-2 OS=Mus musculus GN=Ank2 PE=1 SV=2
evm.model.scaffold9647.10	2.72882	6.5835	-1.27058	0.00213042	sp|P23228|HMCS1_CHICK Hydroxymethylglutaryl-CoA synthase, cytoplasmic OS=Gallus gallus GN=HMGCS1 PE=1 SV=1
evm.model.scaffold91387.9	4.18802	10.6195	-1.34237	0.000820679	sp|Q8C033|ARHGA_MOUSE Rho guanine nucleotide exchange factor 10 OS=Mus musculus GN=Arhgef10 PE=2 SV=2
evm.model.scaffold82339.8	3.45439	7.49656	-1.1178	0.00271512	sp|Q19425|S38A9_CAEEL Sodium-coupled neutral amino acid transporter 9 homolog OS=Caenorhabditis elegans GN=F13H10.3 PE=3 SV=2
evm.model.scaffold19577.4	0.153653	10.5262	-6.09817	0.00380231	sp|Q92000|SHH_XENLA Sonic hedgehog protein OS=Xenopus laevis GN=shh PE=2 SV=1
evm.model.scaffold19577.3	0.257859	18.6667	-6.17774	0.000820679	sp|Q91610|DHHA_XENLA Desert hedgehog protein A OS=Xenopus laevis GN=dhh-a PE=2 SV=1
evm.model.scaffold19577.2	0.166522	23.9566	-7.16856	0.00712329	--
evm.model.scaffold75085.3	17.0386	34.7776	-1.02936	0.000820679	sp|O75165|DJC13_HUMAN DnaJ homolog subfamily C member 13

					OS=Homo sapiens GN=DNAJC13 PE=1 SV=5
evm.model.scaffold104967.1	32.1136	101.236	-1.65646	0.000820679	sp|P70699|LYAG_MOUSE Lysosomal alpha-glucosidase OS=Mus musculus GN=Gaa PE=1 SV=2
evm.model.scaffold174971.10	12.7634	21.2412	-0.734844	0.0101381	sp|Q9BYI3|HYCCI_HUMAN Hyccin OS=Homo sapiens GN=FAM126A PE=1 SV=2
evm.model.scaffold21181.11	0.531417	1.32169	-1.31447	0.0109379	sp|Q7Z408|CSMD2_HUMAN CUB and sushi domain-containing protein 2 OS=Homo sapiens GN=CSMD2 PE=1 SV=2
evm.model.scaffold30425.11	10.2822	37.4302	-1.86405	0.000820679	--
evm.model.scaffold23155.19	11.1267	17.0616	-0.61673	0.035577	sp|Q924T7|RNF31_MOUSE E3 ubiquitin-protein ligase RNF31 OS=Mus musculus GN=Rnf31 PE=1 SV=2
evm.model.scaffold134307.4	8.40878	20.7117	-1.30048	0.000820679	sp|A2AAJ9|OBSCN_MOUSE Obscurin OS=Mus musculus GN=Obscn PE=2 SV=2
evm.model.scaffold134307.3	1.10631	8.03153	-2.85992	0.000820679	--
evm.model.scaffold52321.34	8.23751	26.7166	-1.69746	0.000820679	--
evm.model.scaffold52321.31	21.5634	79.8303	-1.88835	0.000820679	--
evm.model.scaffold132893.19	5.31348	11.0267	-1.05328	0.0316212	sp|Q9NUP1|BL1S4_HUMAN Biogenesis of lysosome-related

					organelles complex 1 subunit 4 OS=Homo sapiens GN=BLOC1S4 PE=1 SV=1
evm.model.scaffold124769.6	11.6908	27.0879	-1.21228	0.000820679	sp|Q92820|GGH_HUMAN Gamma-glutamyl hydrolase OS=Homo sapiens GN=GGH PE=1 SV=2
evm.model.scaffold124769.8	5.85867	11.5695	-0.981681	0.0205228	sp|Q62867|GGH_RAT Gamma-glutamyl hydrolase OS=Rattus norvegicus GN=Ggh PE=1 SV=1
evm.model.scaffold78605.18	3.17866	7.96641	-1.32551	0.000820679	sp|Q7ZWE6|DTB1A_DANRE Dysbindin-A OS=Danio rerio GN=dtnbp1a PE=2 SV=1
evm.model.scaffold76053.19	9.91989	16.5885	-0.741786	0.0382321	sp|Q9HB90|RRAGC_HUMAN Ras-related GTP-binding protein C OS=Homo sapiens GN=RRAGC PE=1 SV=1
evm.model.scaffold76053.18	45.9843	148.78	-1.69396	0.000820679	sp|Q9BQK8|LPIN3_HUMAN Phosphatidate phosphatase LPIN3 OS=Homo sapiens GN=LPIN3 PE=1 SV=3
evm.model.scaffold176599.1	5.07111	9.82403	-0.954014	0.00429787	sp|P35610|SOAT1_HUMAN Sterol O-acyltransferase 1 OS=Homo sapiens GN=SOAT1 PE=1 SV=3

evm.model.scaffold125.5	0.191055	0.692015	-1.85682	0.0335734	sp|P05549|AP2A_HUMAN Transcription factor AP-2-alpha OS=Homo sapiens GN=TFAP2A PE=1 SV=1
evm.model.scaffold125.8	1.2027	7.25418	-2.59253	0.020248	--
evm.model.scaffold86669.25	4.73333	14.5724	-1.62231	0.000820679	sp|Q9D8B3|CHM4B_MOUSE Charged multivesicular body protein 4b OS=Mus musculus GN=Chmp4b PE=1 SV=2
evm.model.scaffold18583.66	11.4712	24.8225	-1.11364	0.000820679	sp|P61023|CHP1_RAT Calcineurin B homologous protein 1 OS=Rattus norvegicus GN=Chp1 PE=1 SV=2
evm.model.scaffold75107.14	11.9592	20.191	-0.755589	0.00933293	sp|Q740Y5|Y1207_MYCPA UPF0353 protein MAP_1207 OS=Mycobacterium paratuberculosis (strain ATCC BAA-968 / K-10) GN=MAP_1207 PE=3 SV=1
evm.model.scaffold43895.17	0	1.3785	#NAME?	0.000820679	--
evm.model.scaffold102645.6	1.42149	3.99063	-1.48921	0.0205228	sp|Q9BSE2|TMM79_HUMAN Transmembrane protein 79 OS=Homo sapiens GN=TMEM79 PE=1 SV=1
evm.model.scaffold50375.49	0.940243	8.50933	-3.17794	0.000820679	sp|Q5M9B1|SPSB3_XENLA SPRY domain-containing SOCS box protein 3 OS=Xenopus laevis GN=spsb3 PE=2 SV=1

evm.model.scaffold165499.1	4.4548	8.29973	-0.897703	0.0173453	sp|Q9EP71|RAI14_MOUSE Ankycorbin OS=Mus musculus GN=Rai14 PE=1 SV=1
evm.model.scaffold172453.22	12.8841	28.9886	-1.1699	0.00271512	sp|P62744|AP2S1_RAT AP-2 complex subunit sigma OS=Rattus norvegicus GN=Ap2s1 PE=1 SV=1
evm.model.scaffold55829.1.1	69.0165	104.33	-0.596144	0.0416339	sp|Q5ZMH1|SEPT2_CHICK Septin-2 OS=Gallus gallus GN=SEPT2 PE=2 SV=1
evm.model.scaffold147877.9	3.52967	10.94	-1.63201	0.000820679	--
evm.model.scaffold176027.35	1.68053	3.63867	-1.1145	0.0173453	sp|Q99LJ8|NGBR_MOUSE Dehydrodolichyl diphosphate syntase complex subunit Nus1 OS=Mus musculus GN=Nus1 PE=2 SV=1
evm.model.scaffold176027.32	8.00719	15.5052	-0.953388	0.00380231	sp|Q14247|SRC8_HUMAN Src substrate cortactin OS=Homo sapiens GN=CTTN PE=1 SV=2
evm.model.scaffold123417.30	0.838919	9.29385	-3.46967	0.000820679	sp|Q6RY07|CHIA_RAT Acidic mammalian chitinase OS=Rattus norvegicus GN=Chia PE=2 SV=1
evm.model.scaffold145623.46	7.64664	13.5135	-0.821509	0.0222103	sp|P11620|YPT1_SCHPO GTP-binding protein ypt1 OS=Schizosaccharomyces pombe (strain 972 / ATCC 24843) GN=ypt1 PE=1 SV=2

evm.model.scaffold145623.40	4.94	21.4232	-2.1166	0.000820679	sp|P12955|PEPD_HUMAN Xaa-Pro dipeptidase OS=Homo sapiens GN=PEPD PE=1 SV=3
evm.model.scaffold66257.98	1.77401	5.82622	-1.71554	0.000820679	sp|Q38SD2|LRRK1_HUMAN Leucine-rich repeat serine/threonine-protein kinase 1 OS=Homo sapiens GN=LRRK1 PE=1 SV=3
evm.model.scaffold54263.30	28.0695	53.9027	-0.941354	0.000820679	--
evm.model.scaffold54263.36	9.42823	17.6077	-0.901148	0.00429787	sp|P58557|YBEY_HUMAN Putative ribonuclease OS=Homo sapiens GN=YBEY PE=1 SV=2
evm.model.scaffold159713.25	8.48819	18.3506	-1.1123	0.0173453	sp|P26885|FKBP2_HUMAN Peptidyl-prolyl cis-trans isomerase FKBP2 OS=Homo sapiens GN=FKBP2 PE=1 SV=2
evm.model.scaffold156763.34	1.96578	3.99721	-1.02389	0.0284609	sp|Q8K4K6|PANK1_MOUSE Pantothenate kinase 1 OS=Mus musculus GN=Pank1 PE=1 SV=1
evm.model.scaffold90899.82	0.0365437	1.05696	-4.85415	0.00670086	--
evm.model.scaffold162999.26	0.0788288	0.389551	-2.30502	0.0302156	sp|Q8HZK2|DUOX2_PIG Dual oxidase 2 OS=Sus scrofa GN=DUOX2 PE=1 SV=2
evm.model.scaffold146623.67	152.181	251.278	-0.723497	0.0117709	sp|Q3T0F5|RAB7A_BOVIN Ras-related protein Rab-7a OS=Bos

					taurus GN=RAB7A PE=2 SV=1
evm.model.scaffold152335.6	3.86688	10.0737	-1.38136	0.00151774	sp|Q9QXM0|ABHD2_MOUSE Abhydrolase domain-containing protein 2 OS=Mus musculus GN=Abhd2 PE=2 SV=1
evm.model.scaffold73127.6	4.0065	9.5406	-1.25174	0.000820679	sp|P59889|S39A1_DANRE Zinc transporter ZIP1 OS=Danio rerio GN=slc39a1 PE=2 SV=1
evm.model.scaffold73127.4	2.29708	10.8384	-2.23828	0.000820679	sp|A4FV52|VGLU1_BOVIN Vesicular glutamate transporter 1 OS=Bos taurus GN=SLC17A7 PE=2 SV=1
evm.model.scaffold73127.3	3.55512	8.93854	-1.33014	0.000820679	sp|Q1L8X9|VGLU3_DANRE Vesicular glutamate transporter 3 OS=Danio rerio GN=slc17a8 PE=3 SV=2
evm.model.scaffold33507.8	8.40224	16.3997	-0.964824	0.00151774	sp|Q8K2K6|AGFG1_MOUSE Arf-GAP domain and FG repeat-containing protein 1 OS=Mus musculus GN=Agfg1 PE=1 SV=2
evm.model.scaffold38291.12	36.6774	65.8255	-0.843754	0.00578348	sp|Q91V37|VATO_MOUSE V-type proton ATPase 21 kDa proteolipid subunit OS=Mus musculus GN=Atp6v0b PE=1 SV=1
evm.model.scaffold5455.25	8.92594	15.8621	-0.829508	0.0117709	sp|Q9Y251|HPSE_HUMAN Heparanase OS=Homo sapiens GN=HPSE PE=1 SV=2

evm.model.scaffold132179.17	0.143274	3.0841	-4.428	0.000820679	sp|Q6BZA0|ALO_DEBHA D-arabinono-1,4-lactone oxidase OS=Debaryomyces hansenii (strain ATCC 36239 / CBS 767 / JCM 1990 / NBRC 0083 / IGC 2968) GN=ALO1 PE=3 SV=2
evm.model.scaffold5677.19	152.4	262.257	-0.783119	0.0248731	sp|Q9QZH4|AAKB2_RAT 5'-AMP-activated protein kinase subunit beta-2 OS=Rattus norvegicus GN=Prkab2 PE=1 SV=1
evm.model.scaffold173785.14	3.41331	7.88359	-1.20768	0.0159979	--
evm.model.scaffold2163.5	3.40224	6.97696	-1.03612	0.000820679	--
evm.model.scaffold197.24	9.32259	18.5606	-0.993438	0.00888556	sp|P42583|NKX25_XENLA Homeobox protein Nkx-2.5 OS=Xenopus laevis GN=nkx-2.5 PE=2 SV=1
evm.model.scaffold124027.1	271.288	564.552	-1.05728	0.000820679	sp|A2AV25|FBCD1_MOUSE Fibrinogen C domain-containing protein 1 OS=Mus musculus GN=Fibcd1 PE=2 SV=1
evm.model.scaffold30711.24	1.77163	3.53846	-0.998045	0.00712329	--
evm.model.scaffold30711.21	2.18947	5.54377	-1.34029	0.000820679	--
evm.model.scaffold30711.22	13.3417	22.2785	-0.739714	0.00712329	sp|Q15262|PTPRK_HUMAN Receptor-type tyrosine-protein phosphatase kappa OS=Homo sapiens

					GN=PTPRK PE=1 SV=2
evm.model.scaffold136165.35	2.45295	5.47047	-1.15715	0.0113727	--
evm.model.scaffold176645.12	6.52136	24.1586	-1.88929	0.000820679	--
evm.model.scaffold176645.10	46.2981	147.753	-1.67416	0.000820679	--
evm.model.scaffold176645.18	3.50059	9.3286	-1.41406	0.000820679	--
evm.model.scaffold176645.19	5.70838	9.99086	-0.807528	0.00532397	sp|Q9UIW2|PLXA1_HUMAN Plexin-A1 OS=Homo sapiens GN=PLXNA1 PE=1 SV=3
evm.model.scaffold107225.80	2.17532	15.6131	-2.84346	0.000820679	sp|Q9QY94|GLNA_ACOCA Glutamine synthetase OS=Acomys cahirinus GN=GLUL PE=2 SV=3
evm.model.scaffold124683.45	2.54765	180.309	-6.14516	0.000820679	sp|P86732|GAAP_HALAI Glycine, alanine and asparagine-rich protein OS=Haliotis asinina PE=1 SV=1
evm.model.scaffold106947.15.1	1.71607	5.15783	-1.58765	0.000820679	sp|Q98ST7|MOXD1_CHICK DBH-like monooxygenase protein 1 OS=Gallus gallus GN=MOXD1 PE=2 SV=1
evm.model.scaffold95039.50	0.764562	8.61128	-3.49352	0.0125549	sp|Q7T3T8|ZAR1_DANRE Zygote arrest protein 1 OS=Danio rerio GN=zar1 PE=2 SV=1
evm.model.scaffold7483.5.3	0.184443	1.621	-3.13564	0.000820679	sp|O95235|KI20A_HUMAN Kinesin-like protein KIF20A OS=Homo sapiens GN=KIF20A PE=1 SV=1
evm.model.scaffold146755.20	9.26646	15.9477	-0.783256	0.016685	sp|Q1LZ74|OARD1_BOVIN O-acetyl-ADP-ribose deacetylase 1

					OS=Bos taurus GN=OARD1 PE=2 SV=1
evm.model.scaffold65307.19	0.27232	0.747895	-1.45753	0.0345537	sp|Q9ULJ7|ANR50_HUMAN Ankyrin repeat domain-containing protein 50 OS=Homo sapiens GN=ANKRD50 PE=1 SV=4
evm.model.scaffold175641.2	0.156636	1.47098	-3.23129	0.0429196	sp|O77404|TYPX_TRYBB Tryparedoxin OS=Trypanosoma brucei brucei PE=1 SV=1
evm.model.scaffold56755.109	2.21133	4.96805	-1.16777	0.0101381	--
evm.model.scaffold136409.33	7.26776	30.8965	-2.08786	0.000820679	--
evm.model.scaffold136409.35	12.2202	20.6398	-0.75616	0.0129481	sp|Q9BX84|TRPM6_HUMAN Transient receptor potential cation channel subfamily M member 6 OS=Homo sapiens GN=TRPM6 PE=1 SV=2
evm.model.scaffold176645.8	0.340251	0.976015	-1.5203	0.039382	--
evm.model.scaffold176645.9	5.0359	11.0176	-1.12949	0.00429787	--
evm.model.scaffold72763.134	16.8223	30.5995	-0.86313	0.00151774	sp|Q7ZWN4|MIDNA_XENLA Midnolin-A OS=Xenopus laevis GN=midn-a PE=2 SV=1
evm.model.scaffold27307.53	8.08087	14.7699	-0.870077	0.00271512	--
evm.model.scaffold26687.3	27.1234	65.2178	-1.26573	0.000820679	sp|Q5TTG1|VATA_ANOGA V-type proton ATPase catalytic subunit A OS=Anopheles gambiae GN=Vha68-2 PE=3 SV=1

evm.model.scaffold26687.4	46.4367	198.094	-2.09284	0.00380231	sp|Q2TJ56|VATA_AEDAL V-type proton ATPase catalytic subunit A OS=Aedes albopictus GN=VhaA PE=2 SV=1
evm.model.scaffold110867.17	0.564958	1.42146	-1.33115	0.0441781	--
evm.model.scaffold154027.3	10.0377	20.3972	-1.02295	0.000820679	sp|Q05209|PTN12_HUMAN Tyrosine-protein phosphatase non-receptor type 12 OS=Homo sapiens GN=PTPN12 PE=1 SV=3
evm.model.scaffold7483.10	23.3782	47.9034	-1.03496	0.000820679	sp|O54750|CP2J6_MOUSE Cytochrome P450 2J6 OS=Mus musculus GN=Cyp2j6 PE=2 SV=2
evm.model.scaffold64247.36	11.2487	17.9993	-0.678184	0.0347943	sp|Q5XFW8|SEC13_RAT Protein SEC13 homolog OS=Rattus norvegicus GN=Sec13 PE=1 SV=1
evm.model.scaffold95039.52	0.587595	2.49773	-2.08772	0.014476	sp|Q7T3T8|ZAR1_DANRE Zygote arrest protein 1 OS=Danio rerio GN=zar1 PE=2 SV=1
evm.model.scaffold84455.5	9.32182	22.3083	-1.2589	0.000820679	sp|P29355|SEM5_CAEEL Sex muscle abnormal protein 5 OS=Caenorhabditis elegans GN=sem-5 PE=1 SV=1
evm.model.scaffold56259.2	5.79222	11.6376	-1.0066	0.000820679	sp|Q5MB13|ABCG2_MACMU ATP-binding cassette sub-family G member 2 OS=Macaca mulatta GN=ABCG2 PE=2 SV=1

evm.model.scaffold56259.1	7.58082	13.1182	-0.791144	0.0177206	sp|Q9UNQ0|ABCG2_HUMAN ATP-binding cassette sub-family G member 2 OS=Homo sapiens GN=ABCG2 PE=1 SV=3
evm.model.scaffold133775.6	83.3448	128.611	-0.625855	0.0342138	sp|Q9WVL2|STAT2_MOUSE Signal transducer and activator of transcription 2 OS=Mus musculus GN=Stat2 PE=1 SV=1
evm.model.scaffold133775.4	7.4428	25.1187	-1.75484	0.000820679	sp|P41826|HSP72_ANOAL Heat shock protein 70 A2 OS=Anopheles albimanus GN=HSP70A2 PE=3 SV=1
evm.model.scaffold148433.14.3	1.28835	2.42402	-0.911871	0.0205228	sp|Q0IHV1|INF2_XENTR Inverted formin-2 OS=Xenopus tropicalis GN=inf2 PE=2 SV=1
evm.model.scaffold59861.33	2.06254	3.52338	-0.772536	0.0101381	--
evm.model.scaffold90611.5	3.40421	6.39039	-0.908581	0.000820679	--
evm.model.scaffold91437.28	155.447	269.055	-0.791478	0.0177206	sp|Q9UT59|YKJ7_SCHPO Putative uncharacterized oxidoreductase C513.07 OS=Schizosaccharomyces pombe (strain 972 / ATCC 24843) GN=SPAC513.07 PE=3 SV=1
evm.model.scaffold161075.29	8.93823	15.1947	-0.765506	0.0148208	sp|Q5CZR5|NATTL_DANRE Natterin-like protein OS=Danio rerio GN=zgc:113413 PE=2 SV=1
evm.model.scaffold151631.10	6.65175	12.3822	-0.896462	0.00670086	sp|Q5RBU7|PCP_PONAB Lysosomal

					Pro-X carboxypeptidase OS=Pongo abelii GN=PRCP PE=2 SV=1
evm.model.scaffold104849.14	8.68298	22.3181	-1.36195	0.000820679	sp|Q9D1Q1|MPH6_MOUSE M-phase phosphoprotein 6 OS=Mus musculus GN=Mphosph6 PE=1 SV=1
evm.model.scaffold118949.3	5.45733	10.506	-0.94495	0.000820679	sp|Q2M389|WASH7_HUMAN WASH complex subunit 7 OS=Homo sapiens GN=KIAA1033 PE=1 SV=2
evm.model.scaffold77121.3	15.8504	38.7097	-1.28818	0.000820679	sp|P31401|VATB_MANSE V-type proton ATPase subunit B OS=Manduca sexta GN=VHA55 PE=2 SV=1
evm.model.scaffold6521.1	7.35566	19.4246	-1.40096	0.000820679	sp|A2AJ76|HMCN2_MOUSE Hemicentin-2 OS=Mus musculus GN=Hmcn2 PE=2 SV=1
evm.model.scaffold102645.12	0	0.441996	#NAME?	0.000820679	sp|Q9BSE2|TMM79_HUMAN Transmembrane protein 79 OS=Homo sapiens GN=TMEM79 PE=1 SV=1
evm.model.scaffold48963.6	0.741745	1.86909	-1.33334	0.00621905	sp|Q19269|NAS14_CAEEL Zinc metalloproteinase nas-14 OS=Caenorhabditis elegans GN=nas-14 PE=2 SV=2
evm.model.scaffold48963.1	4.94337	7.97439	-0.689879	0.0411246	sp|Q9JLA3|UGGG1_RAT UDP-glucose:glycoprotein glucosyltransferase 1 OS=Rattus

					norvegicus GN=Uggt1 PE=1 SV=2
evm.model.scaffold68265.1	13.8495	58.3005	-2.07368	0.000820679	sp|Q26636|CATL_SARPE Cathepsin L OS=Sarcophaga peregrina PE=1 SV=1
evm.model.scaffold135929.31	0.333006	1.83531	-2.4624	0.000820679	--
evm.model.scaffold84455.10	5.09378	8.20814	-0.688319	0.0231466	sp|Q07553|GCY3E_DROME Guanylate cyclase 32E OS=Drosophila melanogaster GN=Gyc32E PE=1 SV=4
evm.model.scaffold26071.15	0.102658	1.08086	-3.39626	0.0228478	sp|Q9BQS2|SYT15_HUMAN Synaptotagmin-15 OS=Homo sapiens GN=SYT15 PE=2 SV=3
evm.model.scaffold24751.8	9.85505	77.5853	-2.97685	0.000820679	sp|P15287|AGSA_APLCA Adenosine deaminase AGSA OS=Aplysia californica PE=1 SV=2
evm.model.scaffold69241.12	4.1628	8.46403	-1.02379	0.00326205	sp|O15439|MRP4_HUMAN Multidrug resistance-associated protein 4 OS=Homo sapiens GN=ABCC4 PE=1 SV=3
evm.model.scaffold138569.2	6.4841	11.2586	-0.796045	0.00755259	sp|Q28620|NPT2A_RABIT Sodium-dependent phosphate transport protein 2A OS=Oryctolagus cuniculus GN=SLC34A1 PE=2 SV=1
evm.model.scaffold115971.8_evm.model.scaffold115971.7	3.38642	5.38971	-0.670448	0.035577	sp|A5PF44|GARL3_DANRE GTPase-activating Rap/Ran-GAP domain-like protein 3 OS=Danio rerio GN=garnl3 PE=3 SV=1

evm.model.scaffold121775.36	12.9926	56.4671	-2.11972	0.000820679	sp|P02587|TNNC2_PIG Troponin C, skeletal muscle OS=Sus scrofa GN=TNNC2 PE=1 SV=2
evm.model.scaffold54713.33	1.59718	3.2587	-1.02877	0.0218619	sp|Q9V785|3BP5H_DROME SH3 domain-binding protein 5 homolog OS=Drosophila melanogaster GN=pcs PE=1 SV=4
evm.model.scaffold24569.10_evm.model.scaffold24569.11	6.5011	12.3668	-0.927717	0.00429787	sp|Q96RW7|HMCN1_HUMAN Hemicentin-1 OS=Homo sapiens GN=HMCN1 PE=1 SV=2
evm.model.scaffold49617.1	38.8902	111.875	-1.52441	0.000820679	sp|Q91VN0|LRP5_MOUSE Low-density lipoprotein receptor-related protein 5 OS=Mus musculus GN=Lrp5 PE=1 SV=3
evm.model.scaffold27475.7	2.49094	4.8471	-0.960432	0.0427039	sp|C3YWU0|FUCO_BRAFL Alpha-L-fucosidase OS=Branchiostoma floridae GN=BRAFLDRAFT_56888 PE=3 SV=2
evm.model.scaffold146623.19.1	12.1497	21.8367	-0.845832	0.00213042	sp|Q5RBU8|ROA2_PONAB Heterogeneous nuclear ribonucleoproteins A2/B1 OS=Pongo abelii GN=HNRNPA2B1 PE=2 SV=1
evm.model.scaffold15107.12	0.443227	1.8316	-2.04699	0.00972356	sp|Q15043|S39AE_HUMAN Zinc transporter ZIP14 OS=Homo sapiens GN=SLC39A14 PE=1 SV=3

evm.model.scaffold39611.2	3.15565	12.6463	-2.00271	0.000820679	sp|Q2HJ10|ZNT2_MOUSE Zinc transporter 2 OS=Mus musculus GN=Slc30a2 PE=2 SV=1
evm.model.scaffold110569.6	0.276763	2.66795	-3.26901	0.00271512	--
evm.model.scaffold147433.19	4.01169	7.09734	-0.823068	0.014476	sp|P80109|PHLD_BOVIN Phosphatidylinositol-glycan-specific phospholipase D OS=Bos taurus GN=GPLD1 PE=1 SV=1
evm.model.scaffold160657.3	1.26791	2.30365	-0.861475	0.0305208	sp|Q5F478|ANR44_CHICK Serine/threonine-protein phosphatase 6 regulatory ankyrin repeat subunit B OS=Gallus gallus GN=ANKRD44 PE=2 SV=1
evm.model.scaffold147663.11.2	10.8548	21.5615	-0.990129	0.000820679	sp|Q6PD21|SHB_MOUSE SH2 domain-containing adapter protein B OS=Mus musculus GN=Shb PE=1 SV=2
evm.model.scaffold138569.79	9.57985	14.4539	-0.593384	0.0497358	sp|Q1KKZ1|SKAP2_TAKRU Src kinase-associated phosphoprotein 2 OS=Takifugu rubripes GN=skap2 PE=3 SV=1
evm.model.scaffold23201.5	0.337094	1.44831	-2.10315	0.013343	sp|A3FEM2|FEV_DANRE Protein FEV OS=Danio rerio GN=fev PE=2 SV=1
evm.model.scaffold176555.39	17.5128	33.1539	-0.920764	0.000820679	sp|Q9VQG2|APH1_DROME Gamma-secretase subunit Aph-1

					OS=Drosophila melanogaster GN=aph-1 PE=1 SV=1
evm.model.scaffold132197.1	1.34653	3.48877	-1.37347	0.00271512	sp|Q6T752|TLR2_HORSE Toll-like receptor 2 OS=Equus caballus GN=TLR2 PE=2 SV=1
evm.model.scaffold65315.31	5.01317	10.6881	-1.09221	0.00151774	sp|P83088|FUCTC_DROME Alpha-(1,3)-fucosyltransferase C OS=Drosophila melanogaster GN=FucTC PE=2 SV=4
evm.model.scaffold65315.34	620.677	1676.46	-1.4335	0.000820679	sp|Q90YT1|RL37_ICTPU 60S ribosomal protein L37 OS=Ictalurus punctatus GN=rpl37 PE=3 SV=3
evm.model.scaffold39493.1	2.37061	5.67522	-1.25942	0.00842602	--
evm.model.scaffold39493.2	6.97805	17.217	-1.30294	0.000820679	--
evm.model.scaffold143135.12	1.88425	3.61844	-0.941379	0.00480132	sp|A7J1T0|M313B_XENLA Mitogen-activated protein kinase kinase kinase 13-B OS=Xenopus laevis GN=map3k13-b PE=2 SV=1
evm.model.scaffold78215.28	13.0578	27.4612	-1.07248	0.000820679	sp|Q90744|NAGAB_CHICK Alpha-N-acetylgalactosaminidase OS=Gallus gallus GN=NAGA PE=1 SV=1
evm.model.scaffold78215.20	8.84001	13.9147	-0.654495	0.0342138	sp|Q5T197|DCST1_HUMAN DC-STAMP domain-containing protein 1 OS=Homo sapiens

					GN=DCST1 PE=2 SV=1
evm.model.scaffold78215.21	14.1474	33.6674	-1.25082	0.000820679	sp|Q5T1A1|DCST2_HUMAN DC-STAMP domain-containing protein 2 OS=Homo sapiens GN=DCST2 PE=2 SV=2
evm.model.scaffold176559.14	12.9245	22.2167	-0.78153	0.0121758	sp|Q7TQF7|AMPH_MOUSE Amphiphysin OS=Mus musculus GN=Amph PE=1 SV=1
evm.model.scaffold18073.20	4.7571	24.4623	-2.36241	0.000820679	sp|Q338P8|CML8_ORYSJ Probable calcium-binding protein CML8 OS=Oryza sativa subsp. japonica GN=CML8 PE=2 SV=1
evm.model.scaffold14517.2_evm.model.scaffold14517.3	0.275111	0.699031	-1.34534	0.0373071	sp|Q54I71|AARA_DICDI Protein aardvark OS=Dictyostelium discoideum GN=aarA PE=2 SV=1
evm.model.scaffold153393.3	1.16444	4.04653	-1.79705	0.00670086	--
evm.model.scaffold153393.4	2.72711	5.761	-1.07894	0.0169923	sp|Q08890|IDS_MOUSE Iduronate 2-sulfatase OS=Mus musculus GN=Ids PE=2 SV=3
evm.model.scaffold171673.6_evm.model.scaffold171673.7	1.3429	3.5487	-1.40194	0.000820679	sp|Q5ZIJ9|MIB2_CHICK E3 ubiquitin-protein ligase MIB2 OS=Gallus gallus GN=MIB2 PE=2 SV=1
evm.model.scaffold93729.81	67.1961	139.404	-1.05282	0.000820679	sp|O00560|SDCB1_HUMAN Syntenin-1 OS=Homo sapiens GN=SDCBP PE=1 SV=1

evm.model.scaffold51225.49	59.631	229.716	-1.94572	0.000820679	--
evm.model.scaffold122055.2	0.435588	1.64225	-1.91464	0.00326205	sp|Q4UMH6|Y381_RICFE Putative ankyrin repeat protein RF_0381 OS=Rickettsia felis (strain ATCC VR-1525 / URRWXCal2) GN=RF_0381 PE=3 SV=1
evm.model.scaffold80507.7	0.683486	3.35148	-2.29382	0.000820679	sp|Q6NU98|PDK1B_XENLA Serine/threonine-protein kinase pdik1l-B OS=Xenopus laevis GN=pdik1-b PE=2 SV=1
evm.model.scaffold171167.15	2.96917	6.90745	-1.2181	0.00213042	sp|Q99M80|PTPRT_MOUSE Receptor-type tyrosine-protein phosphatase T OS=Mus musculus GN=Ptprt PE=2 SV=2
evm.model.scaffold150821.115	3.93954	8.92396	-1.17965	0.00151774	--
evm.model.scaffold10643.10	2.43868	4.14652	-0.765802	0.0254597	sp|Q6UVM3|KCNT2_HUMAN Potassium channel subfamily T member 2 OS=Homo sapiens GN=KCNT2 PE=1 SV=1
evm.model.scaffold129981.23	2.88527	8.1643	-1.50062	0.0199213	--
evm.model.scaffold129981.21	5.11688	8.88678	-0.796397	0.0195535	sp|P06197|PIS_YEAST CDP-diacylglycerol--inositol 3-phosphatidyltransferase OS=Saccharomyces cerevisiae (strain ATCC 204508 / S288c) GN=PIS1 PE=1

					SV=1
evm.model.scaffold27189.35	64.7813	109.415	-0.756163	0.014476	--
evm.model.scaffold27189.36	8.9803	28.2764	-1.65476	0.000820679	--
evm.model.scaffold170623.10	3.93377	6.89514	-0.809667	0.0441781	sp|P56652|ITIH3_BOVIN Inter-alpha-trypsin inhibitor heavy chain H3 OS=Bos taurus GN=ITIH3 PE=1 SV=2
evm.model.scaffold159887.11	114.994	223.349	-0.957737	0.000820679	sp|P54357|MLC2_DROME Myosin-2 essential light chain OS=Drosophila melanogaster GN=Mlc-c PE=1 SV=1
evm.model.scaffold35915.14	0	12.3259	#NAME?	0.020248	--
evm.model.scaffold115633.47	0.505113	2.23726	-2.14705	0.00972356	sp|Q8BFZ4|FXL21_MOUSE F-box/LRR-repeat protein 21 OS=Mus musculus GN=Fbxl21 PE=1 SV=1
evm.model.scaffold74267.1	3.09823	5.66141	-0.869716	0.020248	sp|Q9WYX8|Y508_THEMA Uncharacterized protein TM_0508 OS=Thermotoga maritima (strain ATCC 43589 / MSB8 / DSM 3109 / JCM 10099) GN=TM_0508 PE=3 SV=1
evm.model.scaffold132131.5.1	9.66773	19.2913	-0.996699	0.000820679	sp|Q9NWM0|SMOX_HUMAN Spermine oxidase OS=Homo sapiens GN=SMOX PE=1 SV=1
evm.model.scaffold103373.1	0.93984	2.8533	-1.60214	0.0105331	sp|P63012|RAB3A_RAT Ras-related protein Rab-3A OS=Rattus norvegicus GN=Rab3a PE=1 SV=1

evm.model.scaffold103373.7	6.1894	11.1683	-0.851545	0.000820679	--
evm.model.scaffold154441.7	74.6515	272.18	-1.86632	0.0284609	sp|P21708|MK03_RAT Mitogen-activated protein kinase 3 OS=Rattus norvegicus GN=Mapk3 PE=1 SV=5
evm.model.scaffold154441.5	53.2706	100.833	-0.920553	0.00151774	sp|Q9WUM4|COR1C_MOUSE Coronin-1C OS=Mus musculus GN=Coro1c PE=1 SV=2
evm.model.scaffold116119.153	1.29071	2.81819	-1.1266	0.00933293	sp|O35136|NCAM2_MOUSE Neural cell adhesion molecule 2 OS=Mus musculus GN=Ncam2 PE=2 SV=1
evm.model.scaffold156687.6	1.10475	4.76321	-2.10822	0.00271512	sp|Q8IWR1|TRI59_HUMAN Tripartite motif-containing protein 59 OS=Homo sapiens GN=TRIM59 PE=1 SV=1
evm.model.scaffold9813.41	1540.77	5487.87	-1.83259	0.00429787	--
evm.model.scaffold84915.28	146.595	267.457	-0.867475	0.00326205	sp|P63081|VATL_RAT V-type proton ATPase 16 kDa proteolipid subunit OS=Rattus norvegicus GN=Atp6v0c PE=2 SV=1
evm.model.scaffold52321.49	1.34597	3.7095	-1.46259	0.0121758	sp|P46635|THTR_CRIGR Thiosulfate sulfurtransferase OS=Cricetulus griseus GN=TST PE=2 SV=2
evm.model.scaffold41199.7	0.140208	3.66117	-4.70667	0.0447111	--
evm.model.scaffold146869.9	0.976116	5.39254	-2.46584	0.000820679	sp|Q9I8F9|HSP71_ORYLA Heat shock

					70 kDa protein 1 OS=Oryzias latipes PE=3 SV=1
evm.model.scaffold60761.15	12.0208	20.1763	-0.747129	0.0105331	sp|Q96MC6|HIAT1_HUMAN Hippocampus abundant transcript 1 protein OS=Homo sapiens GN=HIAT1 PE=2 SV=2
evm.model.scaffold60761.12	66.1257	142.176	-1.1044	0.000820679	sp|P18870|JUN_CHICK Transcription factor AP-1 OS=Gallus gallus GN=JUN PE=1 SV=2
evm.model.scaffold8823.9	1.17487	2.96097	-1.33356	0.0148208	sp|Q9BYV6|TRI55_HUMAN Tripartite motif-containing protein 55 OS=Homo sapiens GN=TRIM55 PE=1 SV=2
evm.model.scaffold28609.7	5.08902	9.86311	-0.954655	0.00578348	sp|Q8WZ42|TITIN_HUMAN Titin OS=Homo sapiens GN=TTN PE=1 SV=4
evm.model.scaffold25407.8	7.68773	13.9833	-0.863073	0.00213042	sp|Q9W6I1|RBPMS_CHICK RNA-binding protein with multiple splicing OS=Gallus gallus GN=RBPMS PE=2 SV=1
evm.model.scaffold161697.23	1.15812	2.42035	-1.06343	0.0413639	sp|Q5F4B8|S46A3_CHICK Solute carrier family 46 member 3 OS=Gallus gallus GN=SLC46A3 PE=2 SV=1
evm.model.scaffold154027.30	33.4266	72.0201	-1.1074	0.000820679	sp|P07746|HMGT_ONCMY High mobility group-T protein

					OS=Oncorhynchus mykiss PE=2 SV=2
evm.model.scaffold152083.1	3.67268	10.3621	-1.49641	0.000820679	sp|Q99758|ABCA3_HUMAN ATP-binding cassette sub-family A member 3 OS=Homo sapiens GN=ABCA3 PE=1 SV=2
evm.model.scaffold67097.11	13.9782	34.0876	-1.28607	0.000820679	sp|O00468|AGRIN_HUMAN Agrin OS=Homo sapiens GN=AGRN PE=1 SV=5
evm.model.scaffold60387.3	0.615088	1.80802	-1.55555	0.0329966	sp|Q9ULX7|CAH14_HUMAN Carbonic anhydrase 14 OS=Homo sapiens GN=CA14 PE=1 SV=1
evm.model.scaffold43895.28	3.95904	7.93706	-1.00345	0.00842602	sp|Q1LXE6|TT39C_DANRE Tetratricopeptide repeat protein 39C OS=Danio rerio GN=ttc39c PE=2 SV=1
evm.model.scaffold30637.5.1	4.46244	8.1554	-0.869921	0.0173453	sp|Q8TEW6|DOK4_HUMAN Docking protein 4 OS=Homo sapiens GN=DOK4 PE=1 SV=2
evm.model.scaffold168485.24	9.74089	20.9504	-1.10485	0.00429787	sp|Q9JHJ7|TEST_MOUSE Testisin OS=Mus musculus GN=Prss21 PE=2 SV=2
evm.model.scaffold71119.30	38.9131	66.2805	-0.768331	0.00480132	sp|Q62059|CSPG2_MOUSE Versican core protein OS=Mus musculus GN=Vcan PE=1 SV=2
evm.model.scaffold23781.5	10.9237	28.7958	-1.3984	0.000820679	sp|Q8JZL1|I17RD_MOUSE Interleukin-17 receptor D OS=Mus

					musculus GN=Il17rd PE=1 SV=1
evm.model.scaffold14517.31	1.2689	3.64023	-1.52045	0.00151774	sp|O00566|MPP10_HUMAN U3 small nucleolar ribonucleoprotein protein MPP10 OS=Homo sapiens GN=MPHOSPH10 PE=1 SV=2
evm.model.scaffold123009.14	2.20602	6.51663	-1.56268	0.000820679	--
evm.model.scaffold54713.41	8.94092	15.422	-0.786492	0.0125549	sp|Q00651|ITA4_MOUSE Integrin alpha-4 OS=Mus musculus GN=Itga4 PE=1 SV=1
evm.model.scaffold119493.1	8.63597	13.87	-0.683539	0.027885	sp|F1Q4S1|ATP9B_DANRE Probable phospholipid-transporting ATPase IIB OS=Danio rerio GN=atp9b PE=3 SV=1
evm.model.scaffold931.2	5.19071	9.73474	-0.907212	0.0101381	sp|Q7ZWG6|PCFT_DANRE Proton-coupled folate transporter OS=Danio rerio GN=slc46a1 PE=2 SV=1
evm.model.scaffold146623.55	11.3266	21.8739	-0.949499	0.000820679	--
evm.model.scaffold38291.26	3.28431	13.4302	-2.03182	0.000820679	sp|Q74FW6|TSAL_GEOSL L-threonine ammonia-lyase OS=Geobacter sulfurreducens (strain ATCC 51573 / DSM 12127 / PCA) GN=tdcB PE=1 SV=1
evm.model.scaffold98335.42	2.21759	5.09855	-1.2011	0.000820679	sp|Q9DDT2|BCAP_CHICK Phosphoinositide 3-kinase adapter protein 1 OS=Gallus gallus GN=PIK3AP1 PE=1 SV=1

evm.model.scaffold98335.47	8.56143	19.6267	-1.1969	0.000820679	--
evm.model.scaffold87549.39	4.89217	9.40113	-0.942359	0.0454783	sp|Q8AVF4|MLECB_XENLA Malectin-B OS=Xenopus laevis GN=mlec-b PE=2 SV=2
evm.model.scaffold90463.14	41.6961	69.2348	-0.731585	0.0148208	sp|Q5R5Z5|ARPC2_PONAB Actin-related protein 2/3 complex subunit 2 OS=Pongo abelii GN=ARPC2 PE=2 SV=1
evm.model.scaffold7723.13	7.04428	21.0222	-1.57739	0.000820679	sp|Q9NPF2|CHSTB_HUMAN Carbohydrate sulfotransferase 11 OS=Homo sapiens GN=CHST11 PE=1 SV=1
evm.model.scaffold25135.22	0.505563	1.46552	-1.53544	0.000820679	sp|Q8C8H8|KY_MOUSE Kyphoscoliosis peptidase OS=Mus musculus GN=Ky PE=1 SV=1
evm.model.scaffold136529.9	0.46274	2.27041	-2.29468	0.00213042	--
evm.model.scaffold72185.48_evm.model.scaffold72185.49	3.53811	6.39959	-0.855	0.00972356	sp|Q4KL91|S36A4_XENLA Proton-coupled amino acid transporter 4 OS=Xenopus laevis GN=slc36a4 PE=2 SV=1
evm.model.scaffold62683.12	8.33039	24.9083	-1.58017	0.000820679	sp|Q3ZBF8|CERS2_BOVIN Ceramide synthase 2 OS=Bos taurus GN=CERS2 PE=2 SV=1
evm.model.scaffold145793.22	0.586508	1.74814	-1.5756	0.0287519	sp|P23819|GRIA2_MOUSE Glutamate receptor 2 OS=Mus musculus

					GN=Gria2 PE=1 SV=3
evm.model.scaffold145793.24	5.36587	10.0526	-0.905685	0.00480132	sp|P19492|GRIA3_RAT Glutamate receptor 3 OS=Rattus norvegicus GN=Gria3 PE=1 SV=1
evm.model.scaffold164773.28	0.461195	3.41202	-2.88718	0.00326205	sp|Q17043|APLY_APLKU Aplysianin-A OS=Aplysia kurodai PE=1 SV=1
evm.model.scaffold47967.11	12.1086	21.1372	-0.803755	0.013343	sp|Q95M17|CHIA_BOVIN Acidic mammalian chitinase OS=Bos taurus GN=CHIA PE=1 SV=1
evm.model.scaffold142393.21	1.74358	3.53542	-1.01983	0.0228478	sp|Q12996|CSTF3_HUMAN Cleavage stimulation factor subunit 3 OS=Homo sapiens GN=CSTF3 PE=1 SV=1
evm.model.scaffold160933.14	5.53222	15.7961	-1.51364	0.000820679	sp|P29775|ETS4_DROME DNA-binding protein D-ETS-4 OS=Drosophila melanogaster GN=Ets98B PE=2 SV=3
evm.model.scaffold96499.17	5.68321	10.7878	-0.924625	0.000820679	sp|O94921|CDK14_HUMAN Cyclin-dependent kinase 14 OS=Homo sapiens GN=CDK14 PE=1 SV=3
evm.model.scaffold29275.8	32.0025	54.1659	-0.759197	0.00621905	sp|Q3SZF2|ARF4_BOVIN ADP-ribosylation factor 4 OS=Bos taurus GN=ARF4 PE=2 SV=3
evm.model.scaffold173895.11	92.6683	187.937	-1.0201	0.000820679	--
evm.model.scaffold173895.12	35.0173	54.4524	-0.636927	0.0308116	sp|P22648|FAS2_SCHAM Fasciclin-2

					OS=Schistocerca americana GN=FAS2 PE=1 SV=2
evm.model.scaffold136165.66	2.99703	9.76444	-1.704	0.000820679	sp|A0JM12|MEG10_XENTR Multiple epidermal growth factor-like domains protein 10 OS=Xenopus tropicalis GN=megf10 PE=2 SV=1
evm.model.scaffold144475.33	12.2064	49.2572	-2.0127	0.000820679	sp|O01991|EF2K_CAEEL Eukaryotic elongation factor 2 kinase OS=Caenorhabditis elegans GN=efk-1 PE=2 SV=1
evm.model.scaffold144475.34	2.05572	9.16272	-2.15613	0.000820679	sp|Q54DK4|AK1_DICDI Alpha-protein kinase 1 OS=Dictyostelium discoideum GN=ak1 PE=3 SV=1
evm.model.scaffold144475.35	8.32167	15.241	-0.873011	0.0447111	sp|Q6B9X6|VWKA_DICDI Alpha-protein kinase vwkA OS=Dictyostelium discoideum GN=vwkA PE=1 SV=1
evm.model.scaffold152681.30	0	0.461141	#NAME?	0.000820679	--
evm.model.scaffold121669.10	1.96105	7.86442	-2.00372	0.000820679	sp|Q8TAD2|IL17D_HUMAN Interleukin-17D OS=Homo sapiens GN=IL17D PE=2 SV=1
evm.model.scaffold121669.14	0.959885	2.3026	-1.26233	0.013343	sp|Q8SWR3|SPR_DROME Sex peptide receptor OS=Drosophila melanogaster GN=SPR PE=1 SV=1

evm.model.scaffold121669.15	2.29713	6.72427	-1.54955	0.000820679	sp|Q8SWR3|SPR_DROME Sex peptide receptor OS=Drosophila melanogaster GN=SPR PE=1 SV=1
evm.model.scaffold176495.81	4.00827	6.91183	-0.786087	0.0231466	sp|Q2VPU4|MLXIP_MOUSE MLX-interacting protein OS=Mus musculus GN=Mlxip PE=1 SV=1
evm.model.scaffold100923.11	0.961404	2.68266	-1.48045	0.0404844	sp|A4IIC5|S39A3_XENTR Zinc transporter ZIP3 OS=Xenopus tropicalis GN=slc39a3 PE=2 SV=1
evm.model.scaffold168231.31	7.59706	12.1529	-0.677789	0.0486765	sp|Q9NUV9|GIMA4_HUMAN GTPase IMAP family member 4 OS=Homo sapiens GN=GIMAP4 PE=1 SV=1
evm.model.scaffold168231.34	1.37767	6.41317	-2.21881	0.00578348	sp|Q9NUV9|GIMA4_HUMAN GTPase IMAP family member 4 OS=Homo sapiens GN=GIMAP4 PE=1 SV=1
evm.model.scaffold168231.35	4.73722	7.37686	-0.638966	0.0418385	sp|Q69ZL1|FGD6_MOUSE FYVE, RhoGEF and PH domain-containing protein 6 OS=Mus musculus GN=Fgd6 PE=1 SV=2
evm.model.scaffold161513.58	7.42433	16.5256	-1.15437	0.000820679	sp|Q5RBW6|STX12_PONAB Syntaxin-12 OS=Pongo abelii GN=STX12 PE=2 SV=1
evm.model.scaffold23155.5	15.6902	47.1441	-1.58721	0.000820679	--

evm.model.scaffold23155.8	0.637952	2.56239	-2.00597	0.000820679	sp|Q6DDL7|UN93A_XENLA Protein unc-93 homolog A OS=Xenopus laevis GN=unc93a PE=2 SV=1
evm.model.scaffold91157.18.1	2.54205	7.72126	-1.60284	0.000820679	sp|P37662|YHJX_ECOLI Uncharacterized MFS-type transporter YhjX OS=Escherichia coli (strain K12) GN=yhjX PE=1 SV=1
evm.model.scaffold159893.18	127.082	220.449	-0.794686	0.00621905	sp|P15626|GSTM2_MOUSE Glutathione S-transferase Mu 2 OS=Mus musculus GN=Gstm2 PE=1 SV=2
evm.model.scaffold124907.35	8.61341	22.7122	-1.39881	0.000820679	sp|Q99M15|PPIP2_MOUSE Proline-serine-threonine phosphatase-interacting protein 2 OS=Mus musculus GN=Pstpip2 PE=1 SV=4
evm.model.scaffold167793.18	9.52578	16.7507	-0.814312	0.00755259	sp|F8J2D3|PLB_DRYCN Phospholipase-B 81 OS=Drysdalia coronoides PE=1 SV=1
evm.model.scaffold99437.24	62.5754	219.167	-1.80837	0.000820679	sp|Q13887|KLF5_HUMAN Krueppel-like factor 5 OS=Homo sapiens GN=KLF5 PE=1 SV=2
evm.model.scaffold81801.33	2.72771	9.6787	-1.82712	0.000820679	sp|P08953|TOLL_DROME Protein toll OS=Drosophila melanogaster GN=Tl PE=1 SV=1

evm.model.scaffold81149.6	3.13229	9.66102	-1.62496	0.000820679	sp|O94673|YG75_SCHPO Uncharacterized membrane protein C776.05 OS=Schizosaccharomyces pombe (strain 972 / ATCC 24843) GN=SPBC776.05 PE=4 SV=2
evm.model.scaffold175645.16	0	0.720011	#NAME?	0.00532397	sp|Q5R8H3|BAP31_PONAB B-cell receptor-associated protein 31 OS=Pongo abelii GN=BCAP31 PE=2 SV=3
evm.model.scaffold32389.2	2.16375	5.8953	-1.44603	0.0329966	sp|Q8HXX6|SAP3_MACFA Ganglioside GM2 activator OS=Macaca fascicularis GN=GM2A PE=2 SV=2
evm.model.scaffold32389.4	0.295511	0.933299	-1.65913	0.0335734	sp|Q9ULJ7|ANR50_HUMAN Ankyrin repeat domain-containing protein 50 OS=Homo sapiens GN=ANKRD50 PE=1 SV=4
evm.model.scaffold157537.23	2.7924	6.94547	-1.31456	0.0180929	sp|P53817|HRSL3_RAT HRAS-like suppressor 3 OS=Rattus norvegicus GN=Pla2g16 PE=2 SV=2
evm.model.scaffold139565.4	30.0298	45.9928	-0.615012	0.0468595	--
evm.model.scaffold38817.20	5.4119	11.1057	-1.03709	0.00213042	sp|Q02858|TIE2_MOUSE Angiopoietin-1 receptor OS=Mus musculus GN=Tek PE=1 SV=2
evm.model.scaffold176361.47	6.44402	17.4451	-1.43679	0.000820679	sp|Q6DHN0|TMM53_DANRE Transmembrane protein 53 OS=Danio

					rerio GN=tmem53 PE=2 SV=1
evm.model.scaffold89891.26_evm.model.scaffold89891.27	7.6285	18.1807	-1.25294	0.000820679	sp|P11717|MPRI_HUMAN Cation-independent mannose-6-phosphate receptor OS=Homo sapiens GN=IGF2R PE=1 SV=3
evm.model.scaffold1272532.1	2714.87	6521.03	-1.26422	0.00480132	sp|Q9Y4G6|TLN2_HUMAN Talin-2 OS=Homo sapiens GN=TLN2 PE=1 SV=4
evm.model.scaffold78161.1	12.1946	28.6865	-1.23413	0.000820679	sp|B2RU80|PTPRB_MOUSE Receptor-type tyrosine-protein phosphatase beta OS=Mus musculus GN=Ptprb PE=1 SV=1
evm.model.scaffold176235.8	6.13716	12.6938	-1.04848	0.00213042	sp|Q9NU02|ANKE1_HUMAN Ankyrin repeat and EF-hand domain-containing protein 1 OS=Homo sapiens GN=ANKEF1 PE=2 SV=2
evm.model.scaffold112763.38	6.97371	30.4053	-2.12432	0.000820679	sp|P34528|YM67_CAEEL Putative serine protease K12H4.7 OS=Caenorhabditis elegans GN=K12H4.7 PE=3 SV=2
evm.model.scaffold12381.1	7.96208	27.1551	-1.77001	0.000820679	--
evm.model.scaffold97101.11	3.22518	7.16194	-1.15097	0.000820679	sp|Q10982|FUT2_PIG Galactoside 2-alpha-L-fucosyltransferase 2 OS=Sus

					scrofa GN=FUT2 PE=1 SV=3
evm.model.scaffold91387.10	3.94102	8.23472	-1.06315	0.00151774	sp|O15013|ARHGA_HUMAN Rho guanine nucleotide exchange factor 10 OS=Homo sapiens GN=ARHGEF10 PE=1 SV=4
evm.model.scaffold24751.32	60.4008	229.334	-1.92481	0.000820679	sp|Q6P698|PLSL_DANRE Plastin-2 OS=Danio rerio GN=lcp1 PE=2 SV=1
evm.model.scaffold150821.1	2.32848	7.64102	-1.71437	0.000820679	sp|A6QPN6|GILT_BOVIN Gamma-interferon-inducible lysosomal thiol reductase OS=Bos taurus GN=IFI30 PE=2 SV=1
evm.model.scaffold98335.60.1	21.3055	42.6114	-1.00001	0.000820679	sp|P49802|RGS7_HUMAN Regulator of G-protein signaling 7 OS=Homo sapiens GN=RGS7 PE=1 SV=3
evm.model.scaffold20575.22	3.63011	8.81391	-1.27977	0.000820679	sp|P10686|PLCG1_RAT 1-phosphatidylinositol 4,5-bisphosphate phosphodiesterase gamma-1 OS=Rattus norvegicus GN=Plcg1 PE=1 SV=1
evm.model.scaffold20575.23	5.64072	10.431	-0.886923	0.0231466	sp|P08487|PLCG1_BOVIN 1-phosphatidylinositol 4,5-bisphosphate phosphodiesterase gamma-1 OS=Bos taurus GN=PLCG1 PE=1 SV=1
evm.model.scaffold20575.25	7.86004	15.666	-0.995026	0.000820679	sp|P08487|PLCG1_BOVIN

					1-phosphatidylinositol 4,5-bisphosphate phosphodiesterase gamma-1 OS=Bos taurus GN=PLCG1 PE=1 SV=1
evm.model.scaffold154539.49	2.69294	8.11613	-1.59161	0.000820679	sp|Q8N6F8|WBS27_HUMAN Williams-Beuren syndrome chromosomal region 27 protein OS=Homo sapiens GN=WBSCR27 PE=1 SV=2
evm.model.scaffold73893.2	7.53025	14.3128	-0.926534	0.00213042	--
evm.model.scaffold112725.1	5.80335	11.6452	-1.00478	0.0117709	sp|Q6AYF9|IIGP5_RAT Interferon-inducible GTPase 5 OS=Rattus norvegicus GN=Irgc PE=2 SV=1
evm.model.scaffold170995.65	2.44111	6.33851	-1.37661	0.000820679	sp|Q76NI1|VKIND_HUMAN Protein very KIND OS=Homo sapiens GN=KNDC1 PE=2 SV=2
evm.model.scaffold40311.83	6.234	10.3315	-0.728826	0.035577	sp|Q9TRY0|FKBP4_BOVIN Peptidyl-prolyl cis-trans isomerase FKBP4 OS=Bos taurus GN=FKBP4 PE=1 SV=4
evm.model.scaffold34197.16_evm.model.scaffold34197.17	17.7727	98.9942	-2.47768	0.000820679	sp|Q27245|YH24_CAEEL Putative aminopeptidase W07G4.4 OS=Caenorhabditis elegans GN=lap-2 PE=3 SV=1

evm.model.scaffold127417.4	6.09142	10.8797	-0.836788	0.0468595	--
evm.model.scaffold116687.6	6.00145	13.6951	-1.19027	0.000820679	--
evm.model.scaffold116687.7	8.72838	13.4424	-0.62301	0.0497358	sp|Q91Y25|FAM21_CRIGR WASH complex subunit FAM21 OS=Cricetulus griseus GN=Fam21 PE=2 SV=1
evm.model.scaffold167477.23	2.77445	5.8524	-1.07683	0.000820679	sp|Q3U132|TESP1_MOUSE Protein TESPA1 OS=Mus musculus GN=Tespa1 PE=1 SV=1
evm.model.scaffold170927.15	41.2578	141.799	-1.78111	0.000820679	sp|Q60674|NR1D2_MOUSE Nuclear receptor subfamily 1 group D member 2 OS=Mus musculus GN=Nr1d2 PE=1 SV=1
evm.model.scaffold58729.36	1.98759	10.1491	-2.35225	0.000820679	sp|C3YWU0|FUCO_BRAFL Alpha-L-fucosidase OS=Branchiostoma floridae GN=BRAFLDRAFT_56888 PE=3 SV=2
evm.model.scaffold176007.27	22.9812	35.7376	-0.636986	0.0353697	sp|Q8NCC3|PAG15_HUMAN Group XV phospholipase A2 OS=Homo sapiens GN=PLA2G15 PE=1 SV=2
evm.model.scaffold136291.2	9.49146	35.8478	-1.91718	0.000820679	--
evm.model.scaffold124269.14	0.747693	4.02793	-2.42952	0.000820679	sp|Q6TLF6|RGN_DANRE Regucalcin OS=Danio rerio GN=rgn PE=2 SV=1
evm.model.scaffold45799.9	3.0728	8.19301	-1.41484	0.000820679	sp|Q9Y619|ORNT1_HUMAN Mitochondrial ornithine transporter 1

					OS=Homo sapiens GN=SLC25A15 PE=1 SV=1
evm.model.scaffold161771.22	1.25467	3.01084	-1.26286	0.0129481	sp|Q8K0U4|HS12A_MOUSE Heat shock 70 kDa protein 12A OS=Mus musculus GN=Hspa12a PE=1 SV=1
evm.model.scaffold161771.20	193.603	342.415	-0.822646	0.0101381	--
evm.model.scaffold168151.17	0.162149	2.72047	-4.06847	0.0121758	sp|B4IXJ2|CUE_DROGR Protein cueball OS=Drosophila grimshawi GN=cue PE=3 SV=1
evm.model.scaffold148433.140	119.61	220.183	-0.880365	0.0101381	sp|P58295|SC6A5_RAT Sodium- and chloride-dependent glycine transporter 2 OS=Rattus norvegicus GN=Slc6a5 PE=2 SV=1
evm.model.scaffold15567.15	8.45012	13.9077	-0.718843	0.0188471	sp|Q8TCJ2|STT3B_HUMAN Dolichyl-diphosphooligosaccharide--protein glycosyltransferase subunit STT3B OS=Homo sapiens GN=STT3B PE=1 SV=1
evm.model.scaffold122901.22	3.39693	6.10405	-0.845537	0.0465917	sp|P35859|ALS_RAT Insulin-like growth factor-binding protein complex acid labile subunit OS=Rattus norvegicus GN=Igfals PE=1 SV=1
evm.model.scaffold823.8	0.266055	1.28935	-2.27684	0.00842602	sp|P41212|ETV6_HUMAN Transcription factor ETV6 OS=Homo sapiens GN=ETV6 PE=1 SV=1

evm.model.scaffold78161.32	7.72108	22.8097	-1.56277	0.000820679	sp|Q9JMK0|B4GT5_MOUSE Beta-1,4-galactosyltransferase 5 OS=Mus musculus GN=B4galt5 PE=2 SV=2
evm.model.scaffold103395.7.1	3.72695	12.2287	-1.71421	0.000820679	sp|Q6DDL7|UN93A_XENLA Protein unc-93 homolog A OS=Xenopus laevis GN=unc93a PE=2 SV=1
evm.model.scaffold124387.6	19.5002	34.6807	-0.830646	0.00271512	sp|Q5EAJ7|MVP_STRPU Major vault protein OS=Strongylocentrotus purpuratus GN=MVP PE=1 SV=1
evm.model.scaffold65971.19	44.7637	69.7074	-0.638985	0.0439851	sp|P60897|DSS1_MOUSE 26S proteasome complex subunit DSS1 OS=Mus musculus GN=Shfm1 PE=3 SV=1
evm.model.scaffold52321.59	59.5329	112.14	-0.913538	0.00151774	sp|P49415|SDC_DROME Syndecan OS=Drosophila melanogaster GN=Sdc PE=2 SV=2
evm.model.scaffold167055.44	1.08692	5.12869	-2.23834	0.000820679	sp|Q8WTT0|CLC4C_HUMAN C-type lectin domain family 4 member C OS=Homo sapiens GN=CLEC4C PE=1 SV=1
evm.model.scaffold141215.11	4.35673	15.448	-1.82611	0.000820679	--
evm.model.scaffold142393.4	0	1.00675	#NAME?	0.000820679	--
evm.model.scaffold103373.10	1.25105	3.62038	-1.53301	0.000820679	--
evm.model.scaffold141215.9	1.06109	5.56728	-2.39143	0.000820679	--

evm.model.scaffold172527.1	2.18538	14.6079	-2.74079	0.000820679	--
evm.model.scaffold73825.6	17.0782	33.0064	-0.95059	0.000820679	sp|Q90ZE4|PSN2_DANRE Presenilin-2 OS=Danio rerio GN=psen2 PE=2 SV=2
evm.model.scaffold71897.53	26.6975	44.606	-0.740531	0.00578348	sp|P26443|DHE3_MOUSE Glutamate dehydrogenase 1, mitochondrial OS=Mus musculus GN=Glud1 PE=1 SV=1
evm.model.scaffold60761.1	1.27748	2.37555	-0.894967	0.0361439	--
evm.model.scaffold70561.45	2.57547	7.46307	-1.53493	0.000820679	sp|Q2KII1|ATF3_BOVIN Cyclic AMP-dependent transcription factor ATF-3 OS=Bos taurus GN=ATF3 PE=2 SV=1
evm.model.scaffold43975.8	0.089665	1.72961	-4.26975	0.000820679	--
evm.model.scaffold54817.7	3.77028	16.885	-2.163	0.000820679	sp|P43090|HEM0_OPSTA 5-aminolevulinate synthase, erythroid-specific, mitochondrial OS=Opsanus tau GN=alas2 PE=2 SV=1
evm.model.scaffold124723.3	9.36494	22.8494	-1.28681	0.000820679	sp|Q9QYH9|TNF14_MOUSE Tumor necrosis factor ligand superfamily member 14 OS=Mus musculus GN=Tnfsf14 PE=2 SV=1
evm.model.scaffold1294640.1	319.826	732.255	-1.19506	0.00326205	sp|P16056|MET_MOUSE Hepatocyte growth factor receptor OS=Mus musculus GN=Met PE=1 SV=1

evm.model.scaffold165855.26	0	10.9399	#NAME?	0.0109379	--
evm.model.scaffold165855.27	0	0.95869	#NAME?	0.000820679	--
evm.model.scaffold165855.23	0	2.04016	#NAME?	0.000820679	sp|P86727|QRP_HALAI Glutamine-rich protein OS=Haliotis asinina PE=1 SV=1
evm.model.scaffold165855.29	0	0.827209	#NAME?	0.0418385	--
evm.model.scaffold84179.8	193.683	537.714	-1.47314	0.000820679	sp|O13035|SAP_CHICK Proactivator polypeptide OS=Gallus gallus GN=PSAP PE=1 SV=1
evm.model.scaffold26913.5	6.46444	27.4763	-2.08759	0.00480132	--
evm.model.scaffold26913.4	7.30804	33.9425	-2.21554	0.000820679	sp|O35548|MMP16_RAT Matrix metalloproteinase-16 OS=Rattus norvegicus GN=Mmp16 PE=2 SV=1
evm.model.scaffold26913.3	3.03026	16.5878	-2.45261	0.000820679	sp|Q9Y5R2|MMP24_HUMAN Matrix metalloproteinase-24 OS=Homo sapiens GN=MMP24 PE=2 SV=1
evm.model.scaffold103525.14	2.51497	3.95627	-0.653601	0.0299214	sp|Q9TZM3|LRK1_CAEEL Leucine-rich repeat serine/threonine-protein kinase 1 OS=Caenorhabditis elegans GN=lrk-1 PE=1 SV=6
evm.model.scaffold27307.2	17.3435	47.3068	-1.44765	0.000820679	sp|A1A4M6|STAR5_BOVIN StAR-related lipid transfer protein 5 OS=Bos taurus GN=STARD5 PE=2 SV=1

evm.model.scaffold16007.8	197.675	430.527	-1.12297	0.000820679	sp|Q9R0R1|TRFM_MOUSE Melanotransferrin OS=Mus musculus GN=Mfi2 PE=2 SV=1
evm.model.scaffold16007.9	117.36	256.412	-1.12753	0.000820679	sp|P08582|TRFM_HUMAN Melanotransferrin OS=Homo sapiens GN=MFI2 PE=1 SV=2
evm.model.scaffold4289.56	0.722781	2.70962	-1.90646	0.00151774	--
evm.model.scaffold150685.3	11.1708	18.6716	-0.741106	0.0169923	sp|Q96JK4|HIPL1_HUMAN HHIP-like protein 1 OS=Homo sapiens GN=HHIPL1 PE=2 SV=2
evm.model.scaffold171299.1	0.41829	1.35252	-1.69307	0.0109379	sp|P18433|PTPRA_HUMAN Receptor-type tyrosine-protein phosphatase alpha OS=Homo sapiens GN=PTPRA PE=1 SV=2
evm.model.scaffold2221.17	6.03711	9.47768	-0.650677	0.0370657	sp|Q803C9|PTSS1_DANRE Phosphatidylserine synthase 1 OS=Danio rerio GN=ptdss1 PE=2 SV=2
evm.model.scaffold140847.46	9.39776	23.2513	-1.30693	0.000820679	sp|Q9WTQ7|GA45G_RAT Growth arrest and DNA damage-inducible protein GADD45 gamma OS=Rattus norvegicus GN=Gadd45g PE=2 SV=1
evm.model.scaffold140847.42	14.4841	24.403	-0.75259	0.013343	sp|P48316|GA45A_MOUSE Growth arrest and DNA damage-inducible protein GADD45 alpha OS=Mus musculus GN=Gadd45a PE=1 SV=1

evm.model.scaffold56755.92	10.9929	24.862	-1.17738	0.000820679	sp|Q8CFN2|CDC42_RAT Cell division control protein 42 homolog OS=Rattus norvegicus GN=Cdc42 PE=1 SV=2
evm.model.scaffold146665.18	2.68948	5.81253	-1.11184	0.00429787	sp|Q3U145|TMM64_MOUSE Transmembrane protein 64 OS=Mus musculus GN=Tmem64 PE=2 SV=1
evm.model.scaffold154529.62	2.59418	10.4056	-2.00401	0.000820679	--
evm.model.scaffold115363.24	1.64036	2.82448	-0.783974	0.0287519	sp|Q8IWY4|SCUB1_HUMAN Signal peptide, CUB and EGF-like domain-containing protein 1 OS=Homo sapiens GN=SCUBE1 PE=1 SV=3
evm.model.scaffold162731.69	6.34082	30.0744	-2.24579	0.000820679	sp|Q90744|NAGAB_CHICK Alpha-N-acetylgalactosaminidase OS=Gallus gallus GN=NAGA PE=1 SV=1
evm.model.scaffold162731.67	1.6737	5.19292	-1.63351	0.000820679	sp|A4IF63|TRIM2_BOVIN Tripartite motif-containing protein 2 OS=Bos taurus GN=TRIM2 PE=2 SV=1
evm.model.scaffold106897.1.1	2.76504	6.12847	-1.14823	0.000820679	sp|Q92113|CP17A_SQUAC Steroid 17-alpha-hydroxylase/17,20 lyase OS=Squalus acanthias GN=CYP17A1 PE=2 SV=1
evm.model.scaffold163753.33	1.25587	2.96054	-1.23718	0.00842602	sp|Q9VCA8|ANKHM_DROME

					Ankyrin repeat and KH domain-containing protein mask OS=Drosophila melanogaster GN=mask PE=1 SV=2
evm.model.scaffold148461.2	14.5382	23.2518	-0.677493	0.0254597	sp|Q8BTI9|PK3CB_MOUSE Phosphatidylinositol 4,5-bisphosphate 3-kinase catalytic subunit beta isoform OS=Mus musculus GN=Pik3cb PE=1 SV=2
evm.model.scaffold31507.2	10.622	25.9383	-1.28803	0.000820679	--
evm.model.scaffold79179.6.1	18.5833	37.0141	-0.994067	0.000820679	sp|Q5RFT1|S35F6_PONAB Solute carrier family 35 member F6 OS=Pongo abelii GN=SLC35F6 PE=2 SV=1
evm.model.scaffold176119.32	15.4996	39.2461	-1.34031	0.000820679	sp|Q8AVM5|VPP1_XENLA V-type proton ATPase 116 kDa subunit a isoform 1 OS=Xenopus laevis GN=atp6v0a1 PE=2 SV=1
evm.model.scaffold99021.19	6.5496	16.3189	-1.31706	0.000820679	sp|Q2KIR7|GLYAT_BOVIN Glycine N-acyltransferase OS=Bos taurus GN=GLYAT PE=1 SV=2
evm.model.scaffold171655.10	14.51	43.1986	-1.57394	0.0129481	--
evm.model.scaffold60387.11	0.252941	1.49678	-2.56498	0.0361439	sp|O43614|OX2R_HUMAN Orexin receptor type 2 OS=Homo sapiens GN=HCRTR2 PE=1 SV=2

evm.model.scaffold60387.13	1.96617	4.48697	-1.19036	0.0177206	--
evm.model.scaffold65971.96	1.52708	3.50555	-1.19887	0.0211683	sp|P50429|ARSB_MOUSE Arylsulfatase B OS=Mus musculus GN=Arsb PE=2 SV=3
evm.model.scaffold146247.92	25.3703	39.9507	-0.655081	0.0248731	sp|P46940|IQGA1_HUMAN Ras GTPase-activating-like protein IQGAP1 OS=Homo sapiens GN=IQGAP1 PE=1 SV=1
evm.model.scaffold161771.15	12.4639	23.7252	-0.928666	0.00151774	sp|O95757|HS74L_HUMAN Heat shock 70 kDa protein 4L OS=Homo sapiens GN=HSPA4L PE=1 SV=3
evm.model.scaffold1487.29	3.19523	17.8302	-2.48033	0.000820679	sp|Q9Y6R7|FCGBP_HUMAN IgGFc-binding protein OS=Homo sapiens GN=FCGBP PE=1 SV=3
evm.model.scaffold1487.26	10.2714	24.5407	-1.25654	0.000820679	sp|P17405|ASM_HUMAN Sphingomyelin phosphodiesterase OS=Homo sapiens GN=SMPD1 PE=1 SV=4
evm.model.scaffold1487.27	0.48931	1.79184	-1.87262	0.0121758	sp|Q9R044|NPHN_RAT Nephrin OS=Rattus norvegicus GN=Nphs1 PE=1 SV=2
evm.model.scaffold171579.5	10.3718	49.9855	-2.26884	0.000820679	sp|Q9JJL4|RHOQ_RAT Rho-related GTP-binding protein RhoQ OS=Rattus norvegicus GN=Rhoq PE=2 SV=1
evm.model.scaffold64407.21	1.01887	4.58308	-2.16934	0.000820679	sp|A6NMZ7|CO6A6_HUMAN

					Collagen alpha-6(VI) chain OS=Homo sapiens GN=COL6A6 PE=1 SV=2
evm.model.scaffold148487.32	0.192466	1.52007	-2.98146	0.00755259	--
evm.model.scaffold148487.31	12.4889	28.4206	-1.18629	0.000820679	sp|Q86WC4|OSTM1_HUMAN Osteopetrosis-associated transmembrane protein 1 OS=Homo sapiens GN=OSTM1 PE=1 SV=1
evm.model.scaffold176609.13	2.54861	4.8983	-0.94257	0.00578348	sp|Q9EQ32|BCAP_MOUSE Phosphoinositide 3-kinase adapter protein 1 OS=Mus musculus GN=Pik3ap1 PE=1 SV=1
evm.model.scaffold82057.12	2.48319	9.07468	-1.86965	0.00213042	sp|Q9LFT9|RAH1E_ARATH Ras-related protein RABH1e OS=Arabidopsis thaliana GN=RABH1E PE=2 SV=1
evm.model.scaffold82057.14	4.41234	8.80115	-0.996148	0.0225564	sp|P51147|RAB5C_CANFA Ras-related protein Rab-5C OS=Canis familiaris GN=RAB5C PE=2 SV=1
evm.model.scaffold115633.10	0.563027	1.33779	-1.24857	0.038559	sp|Q9QX05|TLR4_RAT Toll-like receptor 4 OS=Rattus norvegicus GN=Tlr4 PE=2 SV=1
evm.model.scaffold166523.14_evm.model.scaffold166523.15	41.379	22.8236	0.858375	0.00271512	--
evm.model.scaffold107519.13	14.6161	4.70726	1.6346	0.000820679	sp|Q5QYG2|UBIE_IDILO Ubiquinone/menaquinone

					biosynthesis C-methyltransferase UbiE OS=Idiomarina loihiensis (strain ATCC BAA-735 / DSM 15497 / L2-TR) GN=ubiE PE=3 SV=1
evm.model.scaffold53733.19	1.41671	0.325054	2.1238	0.00271512	sp|Q8BXB6|SO2B1_MOUSE Solute carrier organic anion transporter family member 2B1 OS=Mus musculus GN=Slco2b1 PE=1 SV=1
evm.model.scaffold68863.6	51.2765	13.0946	1.96933	0.000820679	sp|Q9Y6K8|KAD5_HUMAN Adenylate kinase isoenzyme 5 OS=Homo sapiens GN=AK5 PE=1 SV=2
evm.model.scaffold68863.2	12.4776	6.66853	0.903896	0.0439851	sp|Q3TQI7|CI078_MOUSE Uncharacterized protein C9orf78 homolog OS=Mus musculus PE=1 SV=2
evm.model.scaffold4397.27	12.6293	7.00939	0.849416	0.00429787	sp|P58215|LOXL3_HUMAN Lysyl oxidase homolog 3 OS=Homo sapiens GN=LOXL3 PE=2 SV=1
evm.model.scaffold82339.11	0.699739	0	inf	0.00621905	sp|Q6AYN4|PHIPL_RAT Phytanoyl-CoA hydroxylase-interacting protein-like OS=Rattus norvegicus GN=Phyhipl PE=2 SV=2
evm.model.scaffold88619.8	0.845091	0.246797	1.77578	0.0351039	sp|Q8ITC7|CAPAR_DROME

					Neuropeptides capa receptor OS=Drosophila melanogaster GN=CapaR PE=2 SV=3
evm.model.scaffold23251.31	187.931	108.659	0.790396	0.00755259	sp|Q9VZI3|UN112_DROME Unc-112-related protein OS=Drosophila melanogaster GN=Fit1 PE=1 SV=1
evm.model.scaffold18583.46	15.4117	5.86846	1.39297	0.00151774	sp|Q9VAI1|CIA30_DROME Complex I intermediate-associated protein 30, mitochondrial OS=Drosophila melanogaster GN=CIA30 PE=2 SV=1
evm.model.scaffold88753.64.1	128.059	64.339	0.993037	0.00151774	sp|P13395|SPTCA_DROME Spectrin alpha chain OS=Drosophila melanogaster GN=alpha-Spec PE=1 SV=2
evm.model.scaffold142905.63	11.1333	5.08477	1.13063	0.000820679	sp|Q9VCA2|ORCT_DROME Organic cation transporter protein OS=Drosophila melanogaster GN=Orct PE=1 SV=1
evm.model.scaffold172453.47	8.85423	4.76186	0.894844	0.00480132	sp|Q4JIJ3|METH_BOVIN Methionine synthase OS=Bos taurus GN=MTR PE=2 SV=1
evm.model.scaffold123417.16	1555.16	682.907	1.1873	0.000820679	--
evm.model.scaffold16669.11.1	24.6684	16.0918	0.616339	0.0347943	sp|Q06725|N2F1A_DANRE Nuclear receptor subfamily 2 group F member

					1-A OS=Danio rerio GN=nr2f1a PE=2 SV=1
evm.model.scaffold166837.70	523.666	236.86	1.14461	0.0101381	sp|Q24498|RY44_DROME Ryanodine receptor 44F OS=Drosophila melanogaster GN=Rya-r44F PE=1 SV=3
evm.model.scaffold169965.25	16.62	10.0598	0.724322	0.0382321	sp|Q1LZ79|GEMI8_BOVIN Gem-associated protein 8 OS=Bos taurus GN=GEMIN8 PE=2 SV=1
evm.model.scaffold17291.22	18.8346	8.64825	1.1229	0.000820679	sp|Q9JLY7|DUS14_MOUSE Dual specificity protein phosphatase 14 OS=Mus musculus GN=Dusp14 PE=2 SV=2
evm.model.scaffold77239.33	84.4401	37.0814	1.18723	0.000820679	sp|P27080|ADT_CHLRE ADP,ATP carrier protein OS=Chlamydomonas reinhardtii GN=ABT PE=2 SV=1
evm.model.scaffold88177.42	534.053	68.2587	2.9679	0.000820679	sp|Q8NBH2|KY_HUMAN Kyphoscoliosis peptidase OS=Homo sapiens GN=KY PE=1 SV=2
evm.model.scaffold160103.4	5.9727	1.83163	1.70526	0.000820679	--
evm.model.scaffold103395.35	83.4381	22.2386	1.90764	0.000820679	sp|Q8TB36|GDAP1_HUMAN Ganglioside-induced differentiation-associated protein 1 OS=Homo sapiens GN=GDAP1 PE=1 SV=3
evm.model.scaffold129981.42	109.153	62.0799	0.814158	0.00326205	sp|Q9VXK0|NIPSN_DROME Protein

					NipSnap OS=Drosophila melanogaster GN=Nipsnap PE=2 SV=2
evm.model.scaffold135.15	664.108	445.558	0.575804	0.048902	sp|Q3ZBI7|USMG5_BOVIN Up-regulated during skeletal muscle growth protein 5 OS=Bos taurus GN=USMG5 PE=1 SV=1
evm.model.scaffold160043.9	76.6965	38.3474	1.00003	0.000820679	sp|Q9Z218|DPP6_MOUSE Dipeptidyl aminopeptidase-like protein 6 OS=Mus musculus GN=Dpp6 PE=1 SV=1
evm.model.scaffold71777.9	16.0763	9.76387	0.719411	0.0434957	sp|Q6GPQ4|SMYD5_XENLA SET and MYND domain-containing protein 5 OS=Xenopus laevis GN=smyd5 PE=2 SV=1
evm.model.scaffold71777.6	10.4952	5.80388	0.854633	0.0361439	sp|Q0VD50|NMNA1_BOVIN Nicotinamide/nicotinic acid mononucleotide adenylyltransferase 1 OS=Bos taurus GN=NMNAT1 PE=2 SV=1
evm.model.scaffold34197.21	3.35923	1.90326	0.819659	0.0482225	sp|Q9Z110|P5CS_MOUSE Delta-1-pyrroline-5-carboxylate synthase OS=Mus musculus GN=Aldh18a1 PE=1 SV=2
evm.model.scaffold34197.23	42.047	11.6242	1.85487	0.000820679	sp|B3EWZ6|MLRP2_ACRMI MAM and LDL-receptor class A domain-containing protein 2

					(Fragment) OS=Acropora millepora PE=1 SV=1
evm.model.scaffold52059.10	2.62873	1.22408	1.10267	0.0353697	sp|Q9LRV8|PIRL2_ARATH Plant intracellular Ras-group-related LRR protein 2 OS=Arabidopsis thaliana GN=PIRL2 PE=2 SV=1
evm.model.scaffold72763.72	44.6392	28.686	0.637968	0.0329966	sp|Q9P0S9|TM14C_HUMAN Transmembrane protein 14C OS=Homo sapiens GN=TMEM14C PE=1 SV=1
evm.model.scaffold142393.96	43.6057	20.6495	1.07841	0.000820679	sp|Q68ER9|EAF6_XENTR Chromatin modification-related protein MEAF6 OS=Xenopus tropicalis GN=meaf6 PE=2 SV=1
evm.model.scaffold142393.93	35.2971	19.9103	0.826035	0.0208422	sp|Q68FG0|ZC4H2_MOUSE Zinc finger C4H2 domain-containing protein OS=Mus musculus GN=Zc4h2 PE=2 SV=1
evm.model.scaffold95809.17	8.8999	5.13059	0.794665	0.0276479	sp|Q9JHX4|CASP8_RAT Caspase-8 OS=Rattus norvegicus GN=Casp8 PE=1 SV=1
evm.model.scaffold95809.10	88.3149	43.9804	1.0058	0.000820679	--
evm.model.scaffold9463.1	1.94705	0.943506	1.04519	0.0251677	sp|Q8CDN9|LRRC9_MOUSE Leucine-rich repeat-containing protein 9 OS=Mus musculus GN=Lrrc9 PE=1

					SV=2
evm.model.scaffold176119.66_evm.model.scaffold176119.67_evm.model.scaffold176119.69	31.6173	20.2273	0.644413	0.0324675	sp|Q27421|OSP_DROME Protein outspread OS=Drosophila melanogaster GN=osp PE=2 SV=5
evm.model.scaffold107939.6	265.674	146.559	0.858174	0.00480132	--
evm.model.scaffold144071.11	23.5769	15.3239	0.621586	0.0418385	sp|P59759|MKL2_MOUSE MKL/myocardin-like protein 2 OS=Mus musculus GN=Mkl2 PE=1 SV=1
evm.model.scaffold39645.3	147.683	64.4671	1.19587	0.000820679	sp|O88799|ZAN_MOUSE Zonadhesin OS=Mus musculus GN=Zan PE=2 SV=1
evm.model.scaffold22223.18	32.1046	21.0797	0.606923	0.0376361	sp|A7Y2W8|SC6A9_XENLA Sodium- and chloride-dependent glycine transporter 1 OS=Xenopus laevis GN=slc6a9 PE=2 SV=1
evm.model.scaffold130319.2	10.742	0.534651	4.32852	0.0318917	sp|Q8R508|FAT3_RAT Protocadherin Fat 3 OS=Rattus norvegicus GN=Fat3 PE=1 SV=1
evm.model.scaffold61415.15	47.0653	27.0319	0.800001	0.00578348	sp|P16617|PGK1_RAT Phosphoglycerate kinase 1 OS=Rattus norvegicus GN=Pgk1 PE=1 SV=2
evm.model.scaffold161853.37	21.441	12.8983	0.733192	0.0484321	sp|P21912|SDHB_HUMAN Succinate dehydrogenase [ubiquinone] iron-sulfur subunit, mitochondrial OS=Homo sapiens GN=SDHB PE=1

					SV=3
evm.model.scaffold171661.26	631.521	104.683	2.5928	0.000820679	sp|P54714|TPIS_CANFA Triosephosphate isomerase OS=Canis familiaris GN=TPI1 PE=1 SV=3
evm.model.scaffold110559.13	58.2565	17.7236	1.71675	0.000820679	--
evm.model.scaffold128661.12	19.6681	11.776	0.740003	0.00933293	sp|Q9BZA8|PC11Y_HUMAN Protocadherin-11 Y-linked OS=Homo sapiens GN=PCDH11Y PE=1 SV=1
evm.model.scaffold40303.22	109.384	33.1456	1.72252	0.000820679	--
evm.model.scaffold142393.77.1	5.4029	2.68793	1.00724	0.00670086	sp|A9JTP3|BIRC7_XENTR Baculoviral IAP repeat-containing protein 7 OS=Xenopus tropicalis GN=birc7 PE=2 SV=1
evm.model.scaffold89811.1	99.1152	59.7029	0.731305	0.00888556	sp|Q8N7N5|DCAF8_MOUSE DDB1- and CUL4-associated factor 8 OS=Mus musculus GN=Dcaf8 PE=1 SV=1
evm.model.scaffold125871.28	29.7595	15.378	0.952483	0.000820679	sp|O57460|TLL1_DANRE Dorsal-ventral patterning tolloid-like protein 1 OS=Danio rerio GN=tll1 PE=2 SV=1
evm.model.scaffold58357.4	16.6038	8.41977	0.979662	0.00326205	sp|Q7SYI5|C1GTB_DANRE Glycoprotein-N-acetylgalactosamine 3-beta-galactosyltransferase 1-B OS=Danio rerio GN=c1galt1b PE=2 SV=1

evm.model.scaffold139609.1	88.6045	57.3365	0.627927	0.0318917	sp|P78333|GPC5_HUMAN Glypican-5 OS=Homo sapiens GN=GPC5 PE=2 SV=1
evm.model.scaffold139609.3	50.4041	31.0195	0.700365	0.0241881	--
evm.model.scaffold95641.11	1193.9	232.003	2.36347	0.000820679	sp|P07291|MLE_ARGIR Myosin essential light chain, striated adductor muscle OS=Argopecten irradians PE=1 SV=2
evm.model.scaffold2427.50	7.64571	4.58008	0.739277	0.0444534	sp|P41437|IAP3_NPVOP E3 ubiquitin-protein ligase IAP-3 OS=Orgyia pseudotsugata multicapsid polyhedrosis virus GN=IAP3 PE=1 SV=1
evm.model.scaffold145865.21	18.979	9.93264	0.934157	0.00271512	--
evm.model.scaffold136775.9	17.1448	10.2556	0.741366	0.0228478	sp|Q9Y6G5|COMDA_HUMAN COMM domain-containing protein 10 OS=Homo sapiens GN=COMMD10 PE=1 SV=1
evm.model.scaffold116119.88	76.7054	40.9968	0.903816	0.00213042	--
evm.model.scaffold70561.18	1.03623	0.370417	1.48412	0.0225564	--
evm.model.scaffold153405.6	87.8546	53.3988	0.718311	0.0169923	sp|Q6P0D0|QKIA_DANRE Protein quaking-A OS=Danio rerio GN=qkia PE=2 SV=2
evm.model.scaffold123407.15	16.888	8.47502	0.99471	0.000820679	sp|Q503L9|NXN_DANRE Nucleoredoxin OS=Danio rerio

					GN=nxn PE=2 SV=1
evm.model.scaffold166523.16	12.1069	6.51376	0.894263	0.00800162	sp|Q12680|GLT1_YEAST Glutamate synthase [NADH] OS=Saccharomyces cerevisiae (strain ATCC 204508 / S288c) GN=GLT1 PE=1 SV=2
evm.model.scaffold166523.17	15.5095	8.18875	0.921436	0.000820679	sp|Q9C102|GLT1_SCHPO Putative glutamate synthase [NADPH] OS=Schizosaccharomyces pombe (strain 972 / ATCC 24843) GN=glt1 PE=2 SV=1
evm.model.scaffold26071.33	15.0141	9.26181	0.696954	0.0476583	sp|Q8BVY0|RL1D1_MOUSE Ribosomal L1 domain-containing protein 1 OS=Mus musculus GN=Rsl1d1 PE=2 SV=1
evm.model.scaffold133775.37	0.457459	0.0930199	2.29803	0.0373071	sp|A6H782|TEKT3_BOVIN Tektin-3 OS=Bos taurus GN=TEKT3 PE=2 SV=1
evm.model.scaffold140941.12	17.6939	3.80437	2.21752	0.000820679	sp|Q5BIM1|TRI45_BOVIN Tripartite motif-containing protein 45 OS=Bos taurus GN=TRIM45 PE=2 SV=1
evm.model.scaffold88219.2	59.8055	23.2768	1.36138	0.000820679	sp|Q687X5|STEA4_HUMAN Metalloreductase STEAP4 OS=Homo sapiens GN=STEAP4 PE=1 SV=1
evm.model.scaffold81993.6	4.01317	1.88878	1.08729	0.016685	--
evm.model.scaffold148011.14	312.748	198.68	0.654555	0.0180929	sp|Q24251|ATP5H_DROME ATP synthase subunit d, mitochondrial

					OS=Drosophila melanogaster GN=ATPsyn-d PE=2 SV=2
evm.model.scaffold167793.36.1	1494.33	209.533	2.83425	0.000820679	sp|O75112|LDB3_HUMAN LIM domain-binding protein 3 OS=Homo sapiens GN=LDB3 PE=1 SV=2
evm.model.scaffold130069.13	11.3851	6.15746	0.886743	0.0156116	sp|Q9R044|NPHN_RAT Nephrin OS=Rattus norvegicus GN=Nphs1 PE=1 SV=2
evm.model.scaffold125621.5	9.16427	2.09074	2.132	0.000820679	--
evm.model.scaffold90031.38	1.52792	0.507035	1.59141	0.0324675	sp|Q8NEG5|ZSWM2_HUMAN E3 ubiquitin-protein ligase ZSWIM2 OS=Homo sapiens GN=ZSWIM2 PE=1 SV=2
evm.model.scaffold81473.34_evm.model.scaffold81473.33	86.8886	53.5539	0.698176	0.0276479	sp|Q9DBR7|MYPT1_MOUSE Protein phosphatase 1 regulatory subunit 12A OS=Mus musculus GN=Ppp1r12a PE=1 SV=2
evm.model.scaffold47331.11	562.921	194.52	1.53301	0.000820679	sp|Q27433|MEC2_CAEEL Mechanosensory protein 2 OS=Caenorhabditis elegans GN=mec-2 PE=1 SV=1
evm.model.scaffold95641.6	7929.57	2658.85	1.57644	0.00578348	sp|P07291|MLE_ARGIR Myosin essential light chain, striated adductor muscle OS=Argopecten irradians PE=1 SV=2

evm.model.scaffold154441.34	21.6301	10.3532	1.06296	0.000820679	sp|Q61382|TRAF4_MOUSE TNF receptor-associated factor 4 OS=Mus musculus GN=Traf4 PE=1 SV=2
evm.model.scaffold161065.1	0.688441	0.294933	1.22295	0.0434957	sp|A2AVA0|SVEP1_MOUSE Sushi, von Willebrand factor type A, EGF and pentraxin domain-containing protein 1 OS=Mus musculus GN=Svep1 PE=1 SV=1
evm.model.scaffold176555.18	68.7121	31.037	1.14657	0.000820679	sp|P42669|PURA_MOUSE Transcriptional activator protein Pur-alpha OS=Mus musculus GN=Pura PE=1 SV=1
evm.model.scaffold65971.38	481.734	254.784	0.918963	0.000820679	sp|Q0MQ97|NDUA4_PONPY Cytochrome c oxidase subunit NDUFA4 OS=Pongo pygmaeus GN=NDUFA4 PE=3 SV=1
evm.model.scaffold37199.21	56.8818	31.265	0.863419	0.000820679	sp|Q92628|K0232_HUMAN Uncharacterized protein KIAA0232 OS=Homo sapiens GN=KIAA0232 PE=1 SV=5
evm.model.scaffold37199.23	103.632	27.0044	1.9402	0.000820679	sp|A6QNP3|PPR3B_BOVIN Protein phosphatase 1 regulatory subunit 3B OS=Bos taurus GN=PPP1R3B PE=2 SV=1
evm.model.scaffold148433.139	13.521	4.24911	1.66997	0.000820679	sp|Q9Y345|SC6A5_HUMAN Sodium-

					and chloride-dependent glycine transporter 2 OS=Homo sapiens GN=SLC6A5 PE=1 SV=3
evm.model.scaffold61991.66	3.07281	1.65941	0.888887	0.0361439	--
evm.model.scaffold63441.39	9.26406	4.36838	1.08454	0.0101381	sp|Q2TBW5|ZNHI2_BOVIN Zinc finger HIT domain-containing protein 2 OS=Bos taurus GN=ZNHIT2 PE=2 SV=1
evm.model.scaffold98043.10	10.3817	6.31331	0.717575	0.027885	sp|Q9UL36|ZN236_HUMAN Zinc finger protein 236 OS=Homo sapiens GN=ZNF236 PE=2 SV=2
evm.model.scaffold150821.41_evm.model.scaffold150821.44	10.1097	2.80055	1.85196	0.000820679	sp|Q60997|DMBT1_MOUSE Deleted in malignant brain tumors 1 protein OS=Mus musculus GN=Dmbt1 PE=1 SV=2
evm.model.scaffold152865.5	5.49208	0.857348	2.6794	0.000820679	sp|Q93RV9|ECTD_STRCO Ectoine dioxygenase OS=Streptomyces coelicolor (strain ATCC BAA-471 / A3(2) / M145) GN=ectD PE=3 SV=2
evm.model.scaffold167489.27	0.632138	0.200493	1.65669	0.000820679	sp|P23098|DYHC_TRIGR Dynein beta chain, ciliary OS=Tripneustes gratilla PE=1 SV=1
evm.model.scaffold167489.20	413.148	142.813	1.53254	0.000820679	sp|Q5R454|AR6P1_PONAB ADP-ribosylation factor-like protein 6-interacting protein 1 OS=Pongo

					abelii GN=ARL6IP1 PE=2 SV=1
evm.model.scaffold125411.6	104.652	30.9694	1.75669	0.000820679	sp|Q9VVY3|GBS76_DROME Glycogen-binding subunit 76A OS=Drosophila melanogaster GN=Gbs-76A PE=1 SV=1
evm.model.scaffold99437.5	76.904	34.0697	1.17457	0.000820679	sp|O54834|RHG06_MOUSE Rho GTPase-activating protein 6 OS=Mus musculus GN=Arhgap6 PE=1 SV=3
evm.model.scaffold160093.12	2.88046	1.29303	1.15554	0.00712329	sp|P15122|ALDR_RABIT Aldose reductase OS=Oryctolagus cuniculus GN=AKR1B1 PE=2 SV=3
evm.model.scaffold26189.41	4.21152	1.55064	1.44148	0.00213042	sp|Q7TNC8|GLRA2_MOUSE Glycine receptor subunit alpha-2 OS=Mus musculus GN=Glra2 PE=2 SV=1
evm.model.scaffold2169.9	10.3221	6.2637	0.720657	0.0152063	sp|Q08AN1|ZN616_HUMAN Zinc finger protein 616 OS=Homo sapiens GN=ZNF616 PE=2 SV=2
evm.model.scaffold132065.8	17.027	9.43234	0.852135	0.0105331	sp|Q02978|M2OM_HUMAN Mitochondrial 2-oxoglutarate/malate carrier protein OS=Homo sapiens GN=SLC25A11 PE=1 SV=3
evm.model.scaffold132065.3	39.1985	18.1074	1.11421	0.000820679	sp|Q9UBQ7|GRHPR_HUMAN Glyoxylate reductase/hydroxypyruvate reductase OS=Homo sapiens GN=GRHPR PE=1

					SV=1
evm.model.scaffold132065.7	79.1894	30.2252	1.38955	0.000820679	sp|Q5XJ36|PARK7_DANRE Protein deglycase DJ-1zDJ-1 OS=Danio rerio GN=park7 PE=2 SV=1
evm.model.scaffold103395.83	162.985	98.4991	0.726558	0.0169923	sp|P55040|GEM_HUMAN GTP-binding protein GEM OS=Homo sapiens GN=GEM PE=1 SV=1
evm.model.scaffold103395.81	8.35814	3.73507	1.16205	0.00271512	sp|P78417|GSTO1_HUMAN Glutathione S-transferase omega-1 OS=Homo sapiens GN=GSTO1 PE=1 SV=2
evm.model.scaffold165923.17	96.5188	29.5859	1.7059	0.000820679	sp|P28227|MAOX_ANAPL NADP-dependent malic enzyme OS=Anas platyrhynchos GN=ME1 PE=1 SV=1
evm.model.scaffold165923.15	294.129	123.308	1.25418	0.000820679	sp|P41976|SODM_BOVIN Superoxide dismutase [Mn], mitochondrial OS=Bos taurus GN=SOD2 PE=2 SV=1
evm.model.scaffold81473.20	198.258	120.745	0.715417	0.013343	sp|Q9VCA2|ORCT_DROME Organic cation transporter protein OS=Drosophila melanogaster GN=Orct PE=1 SV=1
evm.model.scaffold170495.20	1068.56	389.19	1.45712	0.000820679	--
evm.model.scaffold6325.5	3988.83	699.917	2.51071	0.000820679	sp|Q9GP32|ALF_ECHMU Fructose-bisphosphate aldolase

					OS=Echinococcus multilocularis GN=FBPA PE=2 SV=1
evm.model.scaffold78875.6	0.546551	0.207896	1.3945	0.0370657	sp|Q8IVF4|DYH10_HUMAN Dynein heavy chain 10, axonemal OS=Homo sapiens GN=DNAH10 PE=1 SV=4
evm.model.scaffold78875.8	1628.65	794.555	1.03546	0.00326205	sp|P30175|ADF_LILLO Actin-depolymerizing factor OS=Lilium longiflorum PE=2 SV=1
evm.model.scaffold167419.12	158.234	95.4551	0.72917	0.0191834	sp|Q5UQ13|COLL2_MIMIV Collagen-like protein 2 OS=Acanthamoeba polyphaga mimivirus GN=MIMI_R196 PE=4 SV=1
evm.model.scaffold20897.20	84.687	16.7854	2.33493	0.000820679	--
evm.model.scaffold115633.28	30.1429	17.8461	0.75621	0.0152063	sp|Q14432|PDE3A_HUMAN cGMP-inhibited 3',5'-cyclic phosphodiesterase A OS=Homo sapiens GN=PDE3A PE=1 SV=3
evm.model.scaffold54277.11	53.0897	34.5068	0.621552	0.0274321	sp|O00409|FOXN3_HUMAN Forkhead box protein N3 OS=Homo sapiens GN=FOXN3 PE=1 SV=1
evm.model.scaffold157029.21.1	127.327	53.3768	1.25425	0.000820679	sp|P74897|YQA3_THEAQ Universal stress protein in QAH/OAS sulfhydrylase 3'region OS=Thermus aquaticus PE=3 SV=1
evm.model.scaffold39835.10	120.95	60.586	0.997347	0.000820679	sp|P15146|MTAP2_RAT

					Microtubule-associated protein 2 OS=Rattus norvegicus GN=Map2 PE=1 SV=3
evm.model.scaffold55821.12	36.696	19.3674	0.921991	0.0437576	--
evm.model.scaffold163319.8	15.2978	4.76404	1.68307	0.000820679	sp|C9J069|CI172_HUMAN Uncharacterized protein C9orf172 OS=Homo sapiens GN=C9orf172 PE=3 SV=1
evm.model.scaffold163319.9	1.84271	1.03241	0.835812	0.0177206	sp|Q01456|OAM_ASCSU Ovarian abundant message protein OS=Ascaris suum GN=OAM PE=2 SV=1
evm.model.scaffold23897.4	0.742844	0	inf	0.000820679	sp|Q9TU53|CUBN_CANFA Cubilin OS=Canis familiaris GN=CUBN PE=1 SV=1
evm.model.scaffold116119.132	36.7854	21.4157	0.780463	0.0195535	sp|Q9NZV1|CRIM1_HUMAN Cysteine-rich motor neuron 1 protein OS=Homo sapiens GN=CRIM1 PE=1 SV=1
evm.model.scaffold116119.133	24.0851	14.5864	0.723517	0.0287519	sp|Q8AWW5|CRIM1_CHICK Cysteine-rich motor neuron 1 protein OS=Gallus gallus GN=CRIM1 PE=2 SV=1
evm.model.scaffold107225.78	773.907	382.736	1.01581	0.000820679	sp|P51121|GLNA_XENLA Glutamine synthetetase OS=Xenopus laevis GN=glul PE=2 SV=1

evm.model.scaffold108061.6	4567.4	606.921	2.91179	0.000820679	sp|Q16527|CSRP2_HUMAN Cysteine and glycine-rich protein 2 OS=Homo sapiens GN=CSRP2 PE=1 SV=3
evm.model.scaffold58729.4	13.2843	7.19321	0.88502	0.00480132	sp|Q647I9|NALP5_BOVIN NACHT, LRR and PYD domains-containing protein 5 OS=Bos taurus GN=NLRP5 PE=2 SV=1
evm.model.scaffold29855.1	5.68378	2.35693	1.26994	0.00933293	sp|Q9X248|FABG_THEMA 3-oxoacyl-[acyl-carrier-protein] reductase FabG OS=Thermotoga maritima (strain ATCC 43589 / MSB8 / DSM 3109 / JCM 10099) GN=fabG PE=3 SV=1
evm.model.scaffold78787.33	222.982	115.282	0.951767	0.000820679	sp|O08796|EF2K_MOUSE Eukaryotic elongation factor 2 kinase OS=Mus musculus GN=Eef2k PE=1 SV=1
evm.model.scaffold20967.16	170.316	72.5594	1.23098	0.000820679	sp|Q7ZW47|STAU2_DANRE Double-stranded RNA-binding protein Staufen homolog 2 OS=Danio rerio GN=stau2 PE=2 SV=2
evm.model.scaffold113137.24.1	18.089	9.98174	0.857748	0.00151774	sp|Q9U1K1|SPIR_DROME Protein spire OS=Drosophila melanogaster GN=spir PE=1 SV=1
evm.model.scaffold148487.14.1	155.653	46.8596	1.73191	0.000820679	sp|P40222|TXLNA_HUMAN Alpha-taxilin OS=Homo sapiens

					GN=TXLNA PE=1 SV=3
evm.model.scaffold50375.58	3.13032	0.842524	1.89352	0.00888556	sp|Q8C0L6|PAOX_MOUSE Peroxisomal N(1)-acetyl-spermine/spermidine oxidase OS=Mus musculus GN=Paox PE=1 SV=3
evm.model.scaffold172453.33	9.92301	5.91975	0.745241	0.013343	sp|P14543|NID1_HUMAN Nidogen-1 OS=Homo sapiens GN=NID1 PE=1 SV=3
evm.model.scaffold72185.46	97.2323	37.2681	1.38349	0.000820679	sp|P14207|FOLR2_HUMAN Folate receptor beta OS=Homo sapiens GN=FOLR2 PE=1 SV=4
evm.model.scaffold22431.14	229.183	54.4841	2.07259	0.000820679	sp|P51163|HEM4_MOUSE Uroporphyrinogen-III synthase OS=Mus musculus GN=Uros PE=2 SV=1
evm.model.scaffold53391.14	22.2509	13.3286	0.739344	0.00429787	sp|Q80U22|RUSC2_MOUSE Iporin OS=Mus musculus GN=Rusc2 PE=2 SV=2
evm.model.scaffold17291.13	29.3139	16.6818	0.813307	0.00326205	sp|Q5RBZ2|MEP50_PONAB Methylosome protein 50 OS=Pongo abelii GN=WDR77 PE=2 SV=1
evm.model.scaffold99303.7	9.18452	6.04409	0.60368	0.0416339	--
evm.model.scaffold109263.20	787.451	168.41	2.22521	0.000820679	sp|Q7PPA5|ATC1_ANOGA Calcium-transporting ATPase

					sarcoplasmic/endoplasmic reticulum type OS=Anopheles gambiae GN=Ca-P60A PE=3 SV=5
evm.model.scaffold124577.1	29.6123	17.86	0.729465	0.00933293	sp|A2AJ76|HMCN2_MOUSE Hemicentin-2 OS=Mus musculus GN=Hmcn2 PE=2 SV=1
evm.model.scaffold151467.86	23.7483	11.7555	1.01448	0.000820679	sp|Q7TSH2|KPBB_MOUSE Phosphorylase b kinase regulatory subunit beta OS=Mus musculus GN=Phkb PE=1 SV=1
evm.model.scaffold151467.85	52.8237	16.4473	1.68334	0.000820679	sp|Q9QXE4|T53I1_MOUSE Tumor protein p53-inducible nuclear protein 1 OS=Mus musculus GN=Trp53inp1 PE=2 SV=1
evm.model.scaffold47967.7	12.7857	5.28207	1.27536	0.000820679	sp|P52183|ANNU_SCHAM Annulin OS=Schistocerca americana PE=2 SV=1
evm.model.scaffold74183.20	1.97344	0.734802	1.42528	0.0169923	--
evm.model.scaffold74183.22	6.52944	2.29216	1.51025	0.00578348	--
evm.model.scaffold29495.13	49.9836	28.5242	0.809267	0.00326205	sp|P12812|P40_SCHMA Major egg antigen OS=Schistosoma mansoni PE=2 SV=1
evm.model.scaffold80479.22	59.0172	16.2247	1.86295	0.000820679	sp|Q93YS4|AB22G_ARATH ABC transporter G family member 22 OS=Arabidopsis thaliana GN=ABCG22 PE=1 SV=1

evm.model.scaffold16143.1	2148.24	494.121	2.12022	0.000820679	--
evm.model.scaffold2169.13	44.5941	16.7529	1.41244	0.000820679	--
evm.model.scaffold104945.14	11.9328	5.70112	1.06561	0.0137473	sp|P80193|BODG_PSESK Gamma-butyrobetaine dioxygenase OS=Pseudomonas sp. (strain AK-1) PE=1 SV=1
evm.model.scaffold144475.52	60.0311	39.2144	0.614327	0.0439851	sp|Q4KTY1|KPSH1_PINFU Serine/threonine-protein kinase H1 homolog OS=Pinctada fucata GN=PSKH1 PE=2 SV=1
evm.model.scaffold33673.14	86.0693	33.647	1.35502	0.000820679	sp|P49951|CLH1_BOVIN Clathrin heavy chain 1 OS=Bos taurus GN=CLTC PE=1 SV=1
evm.model.scaffold76817.29	3.20699	1.19909	1.41928	0.035577	sp|O05389|YRBE_BACSU Uncharacterized oxidoreductase YrbE OS=Bacillus subtilis (strain 168) GN=yrbE PE=3 SV=2
evm.model.scaffold135471.10	87.7547	28.0929	1.64327	0.000820679	sp|Q5RAE3|LAT2_PONAB Large neutral amino acids transporter small subunit 2 OS=Pongo abelii GN=SLC7A8 PE=2 SV=2
evm.model.scaffold123037.36	7.41499	2.99639	1.30722	0.000820679	sp|Q7T141|PI15A_DANRE Peptidase inhibitor 15-A OS=Danio rerio GN=pi15a PE=3 SV=2
evm.model.scaffold98447.11	13.5185	8.87842	0.606556	0.0486765	sp|Q9HAR2|LPHN3_HUMAN

					Latrophilin-3 OS=Homo sapiens GN=LPHN3 PE=2 SV=2
evm.model.scaffold160425.2	23.2149	11.385	1.02792	0.000820679	--
evm.model.scaffold27307.47	9.52307	4.93633	0.947987	0.00326205	sp|Q9Z160|COG1_MOUSE Conserved oligomeric Golgi complex subunit 1 OS=Mus musculus GN=Cog1 PE=1 SV=3
evm.model.scaffold247.22	82.1367	36.1547	1.18384	0.000820679	--
evm.model.scaffold247.21	868.069	392.913	1.1436	0.00213042	--
evm.model.scaffold134645.3	242.98	113.06	1.10375	0.000820679	sp|P07291|MLE_ARGIR Myosin essential light chain, striated adductor muscle OS=Argopecten irradians PE=1 SV=2
evm.model.scaffold139039.16	0.928438	0.256353	1.85667	0.0125549	--
evm.model.scaffold146247.41	348.278	200.851	0.794115	0.0109379	sp|Q5RE33|REEP5_PONAB Receptor expression-enhancing protein 5 OS=Pongo abelii GN=REEP5 PE=2 SV=1
evm.model.scaffold18503.11	8.2007	3.97832	1.04359	0.0447111	sp|P83565|RM40_RAT 39S ribosomal protein L40, mitochondrial OS=Rattus norvegicus GN=Mrpl40 PE=1 SV=2
evm.model.scaffold175151.16	3.43819	0.502141	2.77549	0.000820679	sp|Q9P0Z9|SOX_HUMAN Peroxisomal sarcosine oxidase OS=Homo sapiens GN=PIPOX PE=1 SV=2

evm.model.scaffold69419.6.4	43.5938	27.9477	0.641396	0.0261282	sp|Q90XB6|SULF1_COTCO Extracellular sulfatase Sulf-1 OS=Coturnix coturnix GN=SULF1 PE=1 SV=1
evm.model.scaffold102677.2	36.2057	22.8166	0.666131	0.0205228	sp|P70372|ELAV1_MOUSE ELAV-like protein 1 OS=Mus musculus GN=Elavl1 PE=1 SV=2
evm.model.scaffold58847.5	19.7027	11.9002	0.727408	0.0305208	sp|Q6ZQ58|LARP1_MOUSE La-related protein 1 OS=Mus musculus GN=Larp1 PE=1 SV=3
evm.model.scaffold171603.7	14.8044	6.6775	1.14865	0.000820679	sp|Q6PCL0|TBX2B_XENLA T-box transcription factor TBX2-B OS=Xenopus laevis GN=tbx2-b PE=2 SV=2
evm.model.scaffold175457.26	8.05528	3.71411	1.11692	0.000820679	--
evm.model.scaffold3243.6	12.456	6.17196	1.01304	0.00151774	sp|P51590|CP2J3_RAT Cytochrome P450 2J3 OS=Rattus norvegicus GN=Cyp2j3 PE=2 SV=1
evm.model.scaffold141697.22	18.4244	10.7551	0.77659	0.0238645	sp|Q5XHH7|SYVNB_XENLA E3 ubiquitin-protein ligase synoviolin B OS=Xenopus laevis GN=syvn1-b PE=2 SV=1
evm.model.scaffold18073.9	13.0397	8.28811	0.653792	0.0441781	sp|Q92870|APBB2_HUMAN Amyloid beta A4 precursor protein-binding family B member 2 OS=Homo sapiens

					GN=APBB2 PE=1 SV=3
evm.model.scaffold150495.3	25.4859	12.847	0.988275	0.000820679	sp|A8MVX0|ARG33_HUMAN Rho guanine nucleotide exchange factor 33 OS=Homo sapiens GN=ARHGEF33 PE=2 SV=2
evm.model.scaffold84953.3	7.58759	2.24195	1.75889	0.000820679	sp|P31228|OXDD_BOVIN D-aspartate oxidase OS=Bos taurus GN=DDO PE=1 SV=2
evm.model.scaffold112249.6	2.48246	0.619964	2.00151	0.00271512	sp|Q9GZV3|SC5A7_HUMAN High affinity choline transporter 1 OS=Homo sapiens GN=SLC5A7 PE=1 SV=1
evm.model.scaffold124269.73	13.9956	7.42407	0.914689	0.00480132	sp|Q9VVY3|GBS76_DROME Glycogen-binding subunit 76A OS=Drosophila melanogaster GN=Gbs-76A PE=1 SV=1
evm.model.scaffold163569.24	14.7124	8.8499	0.7333	0.0329966	sp|P48612|PELO_DROME Protein pelota OS=Drosophila melanogaster GN=pelo PE=2 SV=2
evm.model.scaffold119983.69	153.362	80.0605	0.937777	0.000820679	sp|P19397|CD53_HUMAN Leukocyte surface antigen CD53 OS=Homo sapiens GN=CD53 PE=1 SV=1
evm.model.scaffold37487.25	32.3172	15.8158	1.03094	0.000820679	sp|Q9D3S9|CPVL_MOUSE Probable serine carboxypeptidase CPVL OS=Mus musculus GN=Cpvl PE=2

					SV=2
evm.model.scaffold173875.11.6	27.5256	13.68	1.0087	0.000820679	sp|Q16875|F263_HUMAN 6-phosphofructo-2-kinase/fructose-2,6-bisphosphatase 3 OS=Homo sapiens GN=PFKFB3 PE=1 SV=1
evm.model.scaffold6943.48	20.6688	10.7527	0.942754	0.000820679	sp|P82251|BAT1_HUMAN b(0,+)-type amino acid transporter 1 OS=Homo sapiens GN=SLC7A9 PE=1 SV=1
evm.model.scaffold61991.1	193.07	102.024	0.92022	0.000820679	--
evm.model.scaffold154705.51	38.3423	21.7439	0.818324	0.00380231	sp|Q8WTR2|DUS19_HUMAN Dual specificity protein phosphatase 19 OS=Homo sapiens GN=DUSP19 PE=1 SV=1
evm.model.scaffold90283.1	6.26184	3.17081	0.981735	0.000820679	sp|Q9Y6N5|SQRD_HUMAN Sulfide:quinone oxidoreductase, mitochondrial OS=Homo sapiens GN=SQRDL PE=1 SV=1
evm.model.scaffold150821.16	3.60257	0	inf	0.000820679	sp|Q5UQ35|YR811_MIMIV Putative ariadne-like RING finger protein R811 OS=Acanthamoeba polyphaga mimivirus GN=MIMI_R811 PE=3 SV=1
evm.model.scaffold7521.16	179.926	71.2706	1.33602	0.000820679	--
evm.model.scaffold83847.6	22.4872	14.9685	0.587173	0.0463231	sp|Q7LFX5|CHSTF_HUMAN Carbohydrate sulfotransferase 15 OS=Homo sapiens GN=CHST15 PE=1

					SV=1
evm.model.scaffold153865.1	774.706	201.624	1.94198	0.000820679	sp|A1ZA47|ZASP_DROME PDZ and LIM domain protein Zasp OS=Drosophila melanogaster GN=Zasp52 PE=1 SV=2
evm.model.scaffold162503.2	11.6902	7.17389	0.704477	0.0308116	sp|Q6INU8|TT30A_XENLA Tetratricopeptide repeat protein 30A OS=Xenopus laevis GN=ttc30a PE=2 SV=1
evm.model.scaffold55821.11	35.3918	19.2254	0.880397	0.00213042	sp|Q0KL02|TRIO_MOUSE Triple functional domain protein OS=Mus musculus GN=Trio PE=1 SV=3
evm.model.scaffold147293.13	19.5511	7.40103	1.40145	0.000820679	sp|Q08D11|PTSS2_XENTR Phosphatidylserine synthase 2 OS=Xenopus tropicalis GN=ptdss2 PE=2 SV=2
evm.model.scaffold147293.12	13.2235	3.07469	2.1046	0.044934	--
evm.model.scaffold147293.18	17.1481	11.062	0.632434	0.0351039	sp|O55201|SPT5H_MOUSE Transcription elongation factor SPT5 OS=Mus musculus GN=Supt5h PE=1 SV=1
evm.model.scaffold80573.7	15.7433	9.84873	0.676727	0.0370657	sp|Q9VA73|CMC_DROME Calcium-binding mitochondrial carrier protein Aralar1 OS=Drosophila melanogaster GN=aralar1 PE=2 SV=1

evm.model.scaffold61991.11	76.2872	43.3181	0.816472	0.00326205	sp|Q6P0Q8|MAST2_HUMAN Microtubule-associated serine/threonine-protein kinase 2 OS=Homo sapiens GN=MAST2 PE=1 SV=2
evm.model.scaffold136033.13	359.615	81.3566	2.14412	0.000820679	sp|Q8TC12|RDH11_HUMAN Retinol dehydrogenase 11 OS=Homo sapiens GN=RDH11 PE=1 SV=2
evm.model.scaffold103407.3	2.07561	0.579265	1.84124	0.00670086	sp|Q9UGM3|DMBT1_HUMAN Deleted in malignant brain tumors 1 protein OS=Homo sapiens GN=DMBT1 PE=1 SV=2
evm.model.scaffold70437.9	30.0234	17.2496	0.799524	0.00621905	sp|Q8Q0U0|Y045_METMA Putative ankyrin repeat protein MM_0045 OS=Methanosarcina mazei (strain ATCC BAA-159 / DSM 3647 / Goe1 / Go1 / JCM 11833 / OCM 88) GN=MM_0045 PE=3 SV=1
evm.model.scaffold66257.52	7.70156	3.16444	1.2832	0.000820679	sp|Q9S725|4CL2_ARATH 4-coumarate--CoA ligase 2 OS=Arabidopsis thaliana GN=4CL2 PE=1 SV=2
evm.model.scaffold90713.10	17.5468	8.71444	1.00973	0.0245648	--
evm.model.scaffold90825.1	86.7969	40.5044	1.09956	0.000820679	sp|Q3TZZ7|ESYT2_MOUSE Extended synaptotagmin-2 OS=Mus musculus

					GN=Esyt2 PE=1 SV=1
evm.model.scaffold141451.12	44.3628	16.2842	1.44588	0.000820679	sp|Q9W4Y2|PDFR_DROME PDF receptor OS=Drosophila melanogaster GN=Pdfr PE=1 SV=2
evm.model.scaffold141451.17	41.532	16.5249	1.32958	0.000820679	sp|Q19749|ODP2_CAEEL Dihydrolipoyllysine-residue acetyltransferase component of pyruvate dehydrogenase complex, mitochondrial OS=Caenorhabditis elegans GN=F23B12.5 PE=1 SV=1
evm.model.scaffold171497.15	42.8762	24.8998	0.784043	0.00480132	sp|Q8CCA0|DCNL4_MOUSE DCN1-like protein 4 OS=Mus musculus GN=Dcun1d4 PE=2 SV=1
evm.model.scaffold110259.4	5.14806	2.6172	0.976008	0.0342138	sp|Q96B70|LENG9_HUMAN Leukocyte receptor cluster member 9 OS=Homo sapiens GN=LENG9 PE=2 SV=2
evm.model.scaffold170495.19	3.12871	1.49283	1.06752	0.0388548	sp|P14730|WFD18_RAT WAP four-disulfide core domain protein 18 (Fragment) OS=Rattus norvegicus GN=Wfdc18 PE=2 SV=2
evm.model.scaffold58523.24	3.16577	0.974776	1.69941	0.000820679	sp|Q1JPD8|S43A3_BOVIN Solute carrier family 43 member 3 OS=Bos taurus GN=SLC43A3 PE=2 SV=1
evm.model.scaffold27189.28	31.4012	20.7561	0.597282	0.0434957	sp|P00504|AATC_CHICK Aspartate

					aminotransferase, cytoplasmic OS=Gallus gallus GN=GOT1 PE=1 SV=3
evm.model.scaffold157029.19	72.5762	38.6628	0.908551	0.00213042	sp|P17971|KCNAL_DROME Potassium voltage-gated channel protein Shal OS=Drosophila melanogaster GN=Shal PE=1 SV=2
evm.model.scaffold27129.18	33.0992	14.6423	1.17666	0.000820679	sp|O76840|PPN1_CAEEL Papilin OS=Caenorhabditis elegans GN=mig-6 PE=1 SV=1
evm.model.scaffold74253.19	4.4397	1.75876	1.3359	0.00271512	sp|Q54F25|CLN3_DICDI Battenin OS=Dictyostelium discoideum GN=cln3 PE=3 SV=1
evm.model.scaffold84915.9	54.2653	33.741	0.685526	0.0268133	sp|Q96LD1|SGCZ_HUMAN Zeta-sarcoglycan OS=Homo sapiens GN=SGCZ PE=2 SV=1
evm.model.scaffold17183.32	31.2161	20.456	0.609765	0.044934	--
evm.model.scaffold19571.32	18.302	7.81183	1.22827	0.000820679	--
evm.model.scaffold162457.6	5.17523	3.1456	0.71829	0.0429196	--
evm.model.scaffold146821.14	23.6527	11.7137	1.0138	0.000820679	sp|O08688|CAN5_MOUSE Calpain-5 OS=Mus musculus GN=Capn5 PE=2 SV=1
evm.model.scaffold29275.10	11.8139	6.17283	0.936485	0.00271512	sp|P48728|GCST_HUMAN Aminomethyltransferase, mitochondrial OS=Homo sapiens

					GN=AMT PE=1 SV=1
evm.model.scaffold115729.2	2.98107	1.18312	1.33324	0.0152063	sp|Q9TU53|CUBN_CANFA Cubilin OS=Canis familiaris GN=CUBN PE=1 SV=1
evm.model.scaffold4477.16	24.9772	14.9751	0.738051	0.0173453	sp|O94923|GLCE_HUMAN D-glucuronyl C5-epimerase OS=Homo sapiens GN=GLCE PE=1 SV=3
evm.model.scaffold174195.11	1308	256.094	2.35261	0.000820679	sp|P54985|PPIA_BLAGE Peptidyl-prolyl cis-trans isomerase OS=Blattella germanica GN=CYPA PE=2 SV=1
evm.model.scaffold49049.16	14.8588	7.04906	1.07582	0.000820679	sp|P58421|FZD5_XENLA Frizzled-5 OS=Xenopus laevis GN=fzd5 PE=2 SV=1
evm.model.scaffold18583.22	22.8072	8.88291	1.36039	0.000820679	sp|B0BM95|CS054_XENTR UPF0692 protein C19orf54 homolog OS=Xenopus tropicalis PE=2 SV=2
evm.model.scaffold102223.7_evm.model.scaffold102223.9	22.4254	12.8006	0.808931	0.00271512	sp|O08848|RO60_MOUSE 60 kDa SS-A/Ro ribonucleoprotein OS=Mus musculus GN=Trove2 PE=1 SV=1
evm.model.scaffold75915.5	2330.73	431.094	2.43471	0.000820679	sp|Q96UF2|G3P2_MUCCL Glyceraldehyde-3-phosphate dehydrogenase 2 OS=Mucor circinelloides f. lusitanicus GN=GPD2 PE=3 SV=1

evm.model.scaffold72185.16	101.061	60.1768	0.747954	0.0141107	--
evm.model.scaffold176503.2	241.394	100.694	1.26142	0.000820679	sp|P80049|FABPL_GINCI Fatty acid-binding protein, liver OS=Ginglymostoma cirratum PE=1 SV=1
evm.model.scaffold136529.2	4.79513	1.90371	1.33276	0.000820679	sp|Q9NVP4|DZAN1_HUMAN Double zinc ribbon and ankyrin repeat-containing protein 1 OS=Homo sapiens GN=DZANK1 PE=1 SV=3
evm.model.scaffold16445.4	12.2278	7.94583	0.621899	0.044934	--
evm.model.scaffold35045.5	5.39936	1.59026	1.76352	0.0379495	sp|Q6IFT6|ANO7_RAT Anoctamin-7 OS=Rattus norvegicus GN=Ano7 PE=2 SV=1
evm.model.scaffold156619.13	20.6272	12.7955	0.688913	0.0284609	sp|Q6GMV2|SMYD5_HUMAN SET and MYND domain-containing protein 5 OS=Homo sapiens GN=SMYD5 PE=1 SV=2
evm.model.scaffold88813.3	2.99323	1.09092	1.45616	0.0156116	sp|Q837K0|DEACT_ENTFA Deacetylase EF_0837 OS=Enterococcus faecalis (strain ATCC 700802 / V583) GN=EF_0837 PE=1 SV=1
evm.model.scaffold88813.8	6.47088	1.35732	2.2532	0.000820679	sp|Q0JC44|CML22_ORYSJ Probable calcium-binding protein CML22 OS=Oryza sativa subsp. japonica GN=CML22 PE=3 SV=1

evm.model.scaffold148433.98	31.7416	16.5974	0.935422	0.000820679	sp|Q3UHD3|MTUS2_MOUSE Microtubule-associated tumor suppressor candidate 2 homolog OS=Mus musculus GN=Mtus2 PE=2 SV=1
evm.model.scaffold175603.5.1	9.20886	5.62648	0.710789	0.0302156	sp|Q91VF6|COQA1_MOUSE Collagen alpha-1(XXVI) chain OS=Mus musculus GN=Col26a1 PE=1 SV=1
evm.model.scaffold158547.87	14.5638	8.73557	0.737412	0.0173453	sp|A5D8M0|TYDP2_XENLA Tyrosyl-DNA phosphodiesterase 2 OS=Xenopus laevis GN=tdp2 PE=2 SV=1
evm.model.scaffold29495.29	35.944	21.0506	0.771887	0.00480132	sp|B0W6M9|SUR8_CULQU Leucine-rich repeat protein soc-2 homolog OS=Culex quinquefasciatus GN=Sur-8 PE=3 SV=1
evm.model.scaffold26577.2	78.2836	39.2656	0.995444	0.000820679	sp|Q3UHU5|MTCL1_MOUSE Microtubule cross-linking factor 1 OS=Mus musculus GN=Mtcl1 PE=1 SV=1
evm.model.scaffold26577.1	276.729	128.76	1.10379	0.000820679	sp|Q6NZL0|SOGA3_MOUSE Protein SOGA3 OS=Mus musculus GN=Soga3 PE=2 SV=2
evm.model.scaffold176361.40.2	12.8323	6.70339	0.936814	0.000820679	sp|P17970|KCNAB_DROME Potassium voltage-gated channel

					protein Shab OS=Drosophila melanogaster GN=Shab PE=1 SV=2
evm.model.scaffold65307.21	16.8386	6.21099	1.43887	0.000820679	sp|Q9D8B6|F210B_MOUSE Protein FAM210B OS=Mus musculus GN=Fam210b PE=2 SV=3
evm.model.scaffold90855.11	19.973	12.392	0.688644	0.0222103	sp|P54281|ECLC_BOVIN Epithelial chloride channel protein OS=Bos taurus PE=2 SV=1
evm.model.scaffold73857.1	0.829025	0.355561	1.22132	0.00480132	sp|Q61982|NOTC3_MOUSE Neurogenic locus notch homolog protein 3 OS=Mus musculus GN=Notch3 PE=1 SV=1
evm.model.scaffold161513.27	5.80883	3.24223	0.84126	0.0484321	sp|Q95JD5|ST1B1_CANFA Sulfotransferase family cytosolic 1B member 1 OS=Canis familiaris GN=SULT1B1 PE=1 SV=1
evm.model.scaffold168231.23	114.922	63.9336	0.846004	0.00326205	sp|Q9NUV9|GIMA4_HUMAN GTPase IMAP family member 4 OS=Homo sapiens GN=GIMAP4 PE=1 SV=1
evm.model.scaffold142101.72	93.5984	51.3092	0.867266	0.0225564	--
evm.model.scaffold140461.2	67.6814	34.4277	0.97519	0.000820679	--
evm.model.scaffold98447.41	11.2281	6.64413	0.756964	0.0199213	sp|Q0P5B1|PEX13_BOVIN Peroxisomal membrane protein PEX13 OS=Bos taurus GN=PEX13 PE=2 SV=1

evm.model.scaffold95641.32	101.496	51.673	0.973938	0.000820679	sp|Q14117|DPYS_HUMAN Dihydropyrimidinase OS=Homo sapiens GN=DPYS PE=1 SV=1
evm.model.scaffold133281.1	1.16228	0.402871	1.52857	0.0432327	sp|Q86UC2|RSPH3_HUMAN Radial spoke head protein 3 homolog OS=Homo sapiens GN=RSPH3 PE=1 SV=1
evm.model.scaffold34899.2	35.6228	23.3874	0.607068	0.0391497	sp|P84025|SMAD3_RAT Mothers against decapentaplegic homolog 3 OS=Rattus norvegicus GN=Smad3 PE=1 SV=1
evm.model.scaffold54691.18	5.64494	3.21302	0.813029	0.0125549	sp|P54277|PMS1_HUMAN PMS1 protein homolog 1 OS=Homo sapiens GN=PMS1 PE=1 SV=1
evm.model.scaffold54691.12	19.3205	7.44666	1.37546	0.000820679	sp|P56695|WFS1_MOUSE Wolframin OS=Mus musculus GN=Wfs1 PE=1 SV=1
evm.model.scaffold73895.14	6.32868	3.579	0.822349	0.0316212	sp|Q92574|TSC1_HUMAN Hamartin OS=Homo sapiens GN=TSC1 PE=1 SV=2
evm.model.scaffold73895.15	6.16522	2.64711	1.21974	0.00380231	sp|Q9EP53|TSC1_MOUSE Hamartin OS=Mus musculus GN=Tsc1 PE=1 SV=1
evm.model.scaffold95051.32	34.6419	19.344	0.840633	0.00271512	sp|Q6P0C6|LHPL2_DANRE Lipoma HMGIC fusion partner-like 2 protein

					OS=Danio rerio GN=lhfpl2 PE=2 SV=1
evm.model.scaffold176063.9	140.572	86.6237	0.698477	0.0284609	sp|O73700|CAC1D_CHICK Voltage-dependent L-type calcium channel subunit alpha-1D OS=Gallus gallus GN=CACNA1D PE=2 SV=1
evm.model.scaffold176063.7	22.281	13.8083	0.690278	0.0141107	sp|Q9P291|ARMX1_HUMAN Armadillo repeat-containing X-linked protein 1 OS=Homo sapiens GN=ARMCX1 PE=1 SV=1
evm.model.scaffold51225.1	496.611	237.406	1.06476	0.000820679	--
evm.model.scaffold142905.41	1.74736	0.692516	1.33525	0.00326205	--
evm.model.scaffold123009.81	5.59521	3.24596	0.785549	0.0141107	sp|Q9UNK9|ANGE1_HUMAN Protein angel homolog 1 OS=Homo sapiens GN=ANGEL1 PE=2 SV=1
evm.model.scaffold142133.1	1051.66	314.862	1.73988	0.000820679	sp|Q9BRZ2|TRI56_HUMAN E3 ubiquitin-protein ligase TRIM56 OS=Homo sapiens GN=TRIM56 PE=1 SV=3
evm.model.scaffold3113.6	11.6044	6.46782	0.843326	0.0188471	sp|Q2YDC9|PDCD2_BOVIN Programmed cell death protein 2 OS=Bos taurus GN=PDCD2 PE=2 SV=1
evm.model.scaffold3113.5	123.752	57.4065	1.10816	0.000820679	sp|Q6INU7|FRRS1_XENLA Putative ferric-chelate reductase 1 OS=Xenopus laevis GN=frrs1 PE=2 SV=1
evm.model.scaffold91951.32	27.7434	12.5694	1.14223	0.000820679	sp|P13707|GPDA_MOUSE

					Glycerol-3-phosphate dehydrogenase [NAD(+)], cytoplasmic OS=Mus musculus GN=Gpd1 PE=1 SV=3
evm.model.scaffold91951.31	30.1089	14.2285	1.08141	0.000820679	sp|Q27928|GPDA_DROPS Glycerol-3-phosphate dehydrogenase [NAD(+)], cytoplasmic OS=Drosophila pseudoobscura pseudoobscura GN=Gpdh PE=3 SV=2
evm.model.scaffold110569.10	10.116	4.10891	1.29981	0.00380231	sp|P23416|GLRA2_HUMAN Glycine receptor subunit alpha-2 OS=Homo sapiens GN=GLRA2 PE=2 SV=1
evm.model.scaffold140631.11	12.6307	7.28368	0.794199	0.0152063	--
evm.model.scaffold80931.27	61.4081	35.7597	0.780095	0.00578348	sp|Q0ZM14|PCD15_CHICK Protocadherin-15 OS=Gallus gallus GN=Pcdh15 PE=1 SV=1
evm.model.scaffold174343.18	3.37448	1.78943	0.91517	0.0254597	sp|A0JNA8|PAXI1_BOVIN PAX-interacting protein 1 OS=Bos taurus GN=PAXIP1 PE=2 SV=1
evm.model.scaffold30547.1	5.43452	2.36078	1.20289	0.000820679	sp|Q9IBG7|KCP_XENLA Kielin/chordin-like protein OS=Xenopus laevis GN=kcp PE=2 SV=1
evm.model.scaffold53139.15	5.31399	2.74479	0.953099	0.0211683	sp|Q6GNV7|DIRC2_XENLA Disrupted in renal carcinoma protein 2 homolog OS=Xenopus laevis GN=dirc2

					PE=2 SV=1
evm.model.scaffold22505.19	16.9588	9.85415	0.783235	0.0222103	sp|Q8BIG4|FBX28_MOUSE F-box only protein 28 OS=Mus musculus GN=Fbxo28 PE=2 SV=1
evm.model.scaffold142971.11	78.0147	43.9042	0.829386	0.00429787	sp|B1H2N3|MICU1_XENTR Calcium uptake protein 1, mitochondrial OS=Xenopus tropicalis GN=micu1 PE=2 SV=2
evm.model.scaffold170995.52	3.501	1.01562	1.7854	0.000820679	sp|P42700|RO60_XENLA 60 kDa SS-A/Ro ribonucleoprotein OS=Xenopus laevis GN=trove2 PE=1 SV=1
evm.model.scaffold153897.11	55.3322	34.1161	0.697665	0.0152063	sp|P57103|NAC3_HUMAN Sodium/calcium exchanger 3 OS=Homo sapiens GN=SLC8A3 PE=2 SV=2
evm.model.scaffold173875.10	13.6996	8.8945	0.623145	0.0465917	--
evm.model.scaffold19117.13	51.6486	26.8164	0.945616	0.000820679	sp|Q8C033|ARHGA_MOUSE Rho guanine nucleotide exchange factor 10 OS=Mus musculus GN=Arhgef10 PE=2 SV=2
evm.model.scaffold19117.11	31.5541	12.5262	1.33288	0.000820679	sp|Q6IFT4|RHG20_MOUSE Rho GTPase-activating protein 20 OS=Mus musculus GN=Arhgap20 PE=2 SV=1
evm.model.scaffold78787.18	29.9064	17.572	0.767174	0.00755259	sp|Q03720|SLO_DROME

					Calcium-activated potassium channel slowpoke OS=Drosophila melanogaster GN=slo PE=1 SV=3
evm.model.scaffold51139.47	6.50923	2.62183	1.31191	0.000820679	sp|Q8IUS5|EPHX4_HUMAN Epoxide hydrolase 4 OS=Homo sapiens GN=EPHX4 PE=2 SV=2
evm.model.scaffold119983.31	147.916	80.947	0.869723	0.00213042	sp|Q8WPA2|AR_BOMMO Allatostatin-A receptor OS=Bombyx mori GN=AR PE=2 SV=1
evm.model.scaffold176133.37	34.2616	19.0768	0.844773	0.00213042	sp|Q8BGI4|FA13A_MOUSE Protein FAM13A OS=Mus musculus GN=Fam13a PE=2 SV=1
evm.model.scaffold132647.8	6.3908	2.37677	1.427	0.0125549	sp|Q5BN45|CI116_MOUSE UPF0691 protein C9orf116 homolog OS=Mus musculus PE=2 SV=1
evm.model.scaffold138569.38	7.93037	4.67711	0.761771	0.0218619	sp|Q3U4H6|HEXDC_MOUSE Hexosaminidase D OS=Mus musculus GN=Hexdc PE=1 SV=1
evm.model.scaffold117139.11	74.7043	39.886	0.905309	0.000820679	--
evm.model.scaffold117139.10	249.692	148.553	0.74917	0.00755259	sp|P98165|VLDLR_CHICK Very low-density lipoprotein receptor OS=Gallus gallus GN=VLDLR PE=1 SV=1
evm.model.scaffold72267.2	17.9951	4.93056	1.86778	0.000820679	--
evm.model.scaffold89289.5	16.9558	6.44442	1.39566	0.000820679	sp|Q10341|CYS2_SCHPO Probable

					serine-O-acetyltransferase cys2 OS=Schizosaccharomyces pombe (strain 972 / ATCC 24843) GN=cys2 PE=1 SV=1
evm.model.scaffold145623.3	128.289	51.6519	1.3125	0.000820679	sp|P08059|G6PI_PIG Glucose-6-phosphate isomerase OS=Sus scrofa GN=GPI PE=1 SV=3
evm.model.scaffold931.19	178.327	94.9246	0.909675	0.00271512	sp|P11708|MDHC_PIG Malate dehydrogenase, cytoplasmic OS=Sus scrofa GN=MDH1 PE=1 SV=4
evm.model.scaffold156619.1	35.1337	19.8367	0.82468	0.00213042	sp|Q9Y6M7|S4A7_HUMAN Sodium bicarbonate cotransporter 3 OS=Homo sapiens GN=SLC4A7 PE=1 SV=2
evm.model.scaffold104051.16	80.6154	29.0731	1.47137	0.000820679	--
evm.model.scaffold136189.2	424.851	145.87	1.54228	0.000820679	sp|Q9QYI5|DNJB2_MOUSE DnaJ homolog subfamily B member 2 OS=Mus musculus GN=Dnajb2 PE=1 SV=2
evm.model.scaffold23177.1	50.6881	28.1751	0.847225	0.0361439	sp|P48148|RHO1_DROME Ras-like GTP-binding protein Rho1 OS=Drosophila melanogaster GN=Rho1 PE=1 SV=1
evm.model.scaffold23177.4	3.9873	1.87039	1.09207	0.0211683	sp|Q5ZKW0|MTU1_CHICK Mitochondrial tRNA-specific 2-thiouridylase 1 OS=Gallus gallus

					GN=TRMU PE=2 SV=1
evm.model.scaffold142031.2	50.304	27.9791	0.846325	0.000820679	sp|Q6ZR37|PKHG7_HUMAN Pleckstrin homology domain-containing family G member 7 OS=Homo sapiens GN=PLEKHG7 PE=2 SV=1
evm.model.scaffold175645.55	36.3639	17.2263	1.07789	0.000820679	--
evm.model.scaffold119793.3	20.0444	11.1166	0.850481	0.00213042	sp|P83088|FUCTC_DROME Alpha-(1,3)-fucosyltransferase C OS=Drosophila melanogaster GN=FucTC PE=2 SV=4
evm.model.scaffold11819.2	13.4842	7.15991	0.913259	0.000820679	sp|Q9H0K1|SIK2_HUMAN Serine/threonine-protein kinase SIK2 OS=Homo sapiens GN=SIK2 PE=1 SV=1
evm.model.scaffold65971.63	707.892	433.371	0.707928	0.014476	sp|Q9U505|AT5G_MANSE ATP synthase lipid-binding protein, mitochondrial OS=Manduca sexta PE=2 SV=1
evm.model.scaffold143641.4	3.30042	0.676836	2.28577	0.000820679	sp|Q9EPH0|S26A5_RAT Prestin OS=Rattus norvegicus GN=Slc26a5 PE=1 SV=1
evm.model.scaffold61349.1	141.248	52.7568	1.42081	0.000820679	sp|Q6UPE0|CHDH_RAT Choline dehydrogenase, mitochondrial OS=Rattus norvegicus GN=Chdh PE=1

					SV=1
evm.model.scaffold176495.17	45.1768	21.6692	1.05993	0.000820679	sp|A8Y1P7|BRE4_CAEBR Beta-1,4-N-acetylgalactosaminyltransferase bre-4 OS=Caenorhabditis briggsae GN=bre-4 PE=3 SV=1
evm.model.scaffold88753.112	13.3664	8.6149	0.633705	0.0353697	sp|Q9VLT5|POE_DROME Protein purity of essence OS=Drosophila melanogaster GN=poe PE=1 SV=1
evm.model.scaffold76655.18	48.1309	21.3503	1.17271	0.000820679	sp|P25931|NPYR_DROME Neuropeptide Y receptor OS=Drosophila melanogaster GN=RYa-R PE=2 SV=2
evm.model.scaffold175495.32	19.4137	10.6793	0.862256	0.00888556	sp|A2AGA4|RHBL2_MOUSE Rhomboid-related protein 2 OS=Mus musculus GN=Rhbdl2 PE=1 SV=1
evm.model.scaffold83333.31	32.0158	17.4096	0.8789	0.00326205	--
evm.model.scaffold142971.7	52.0711	21.7517	1.25935	0.000820679	sp|Q5E9P9|GLYC_BOVIN Serine hydroxymethyltransferase, cytosolic OS=Bos taurus GN=SHMT1 PE=2 SV=3
evm.model.scaffold175113.4	65.8057	21.8972	1.58746	0.000820679	--
evm.model.scaffold76813.3	79.998	30.7737	1.37827	0.0125549	sp|Q9PUU6|FZD2_XENLA Frizzled-2 OS=Xenopus laevis GN=fzd2 PE=2 SV=1
evm.model.scaffold111781.24	10.8929	6.79302	0.681266	0.0439851	sp|Q9I8C7|ACH10_CHICK Neuronal acetylcholine receptor subunit

					alpha-10 OS=Gallus gallus GN=CHRNA10 PE=3 SV=1
evm.model.scaffold163753.29	20.5233	7.84742	1.38698	0.000820679	sp|Q8TCB7|METL6_HUMAN Methyltransferase-like protein 6 OS=Homo sapiens GN=METTL6 PE=2 SV=2
evm.model.scaffold171689.34	3.76219	1.95405	0.945105	0.0238645	sp|P70097|C560_CRIGR Succinate dehydrogenase cytochrome b560 subunit, mitochondrial OS=Cricetulus griseus GN=SDHC PE=2 SV=1
evm.model.scaffold71119.76	11.8312	7.45363	0.666585	0.0439851	--
evm.model.scaffold36337.3	78.1243	23.8168	1.71379	0.000820679	sp|Q5RKL5|STEA3_RAT Metalloreductase STEAP3 OS=Rattus norvegicus GN=Steap3 PE=2 SV=1
evm.model.scaffold110817.24	2.18704	0.498199	2.13419	0.000820679	sp|P24862|CCNB_PATVU G2/mitotic-specific cyclin-B OS=Patella vulgata PE=2 SV=1
evm.model.scaffold78701.7	8.82826	4.9537	0.833622	0.00480132	sp|A4II29|NRARP_XENTR Notch-regulated ankyrin repeat-containing protein OS=Xenopus tropicalis GN=nrarp PE=3 SV=1
evm.model.scaffold128661.3	7.76645	2.49056	1.64078	0.000820679	--
evm.model.scaffold93729.42	39.7141	21.7453	0.86895	0.000820679	sp|Q5RD08|OSER1_PONAB Oxidative stress-responsive serine-rich protein 1 OS=Pongo abelii GN=OSER1

					PE=2 SV=1
evm.model.scaffold103057.9	22.1361	12.8109	0.789035	0.0148208	sp|Q6GQ48|LIAS_XENLA Lipoyl synthase, mitochondrial OS=Xenopus laevis GN=lias PE=2 SV=1
evm.model.scaffold162531.3	1.73649	0.311063	2.4809	0.016685	sp|B3EWY9|MLP_ACRMI Mucin-like protein (Fragment) OS=Acropora millepora PE=1 SV=1
evm.model.scaffold76655.21	51.201	18.336	1.48149	0.000820679	sp|Q6P8Y1|CAPSL_MOUSE Calcyphosin-like protein OS=Mus musculus GN=Capsl PE=2 SV=4
evm.model.scaffold176609.4	6.67723	1.09359	2.61018	0.000820679	sp|P86729|EPDR2_HALAI Ependymin-related protein 2 OS=Haliotis asinina PE=1 SV=1
evm.model.scaffold106529.16	16.4084	3.795	2.11226	0.000820679	--
evm.model.scaffold146665.9	4.55612	1.60858	1.50202	0.000820679	--
evm.model.scaffold80507.13	65.9284	34.9441	0.915852	0.000820679	sp|Q8BGW8|VGLL2_MOUSE Transcription cofactor vestigial-like protein 2 OS=Mus musculus GN=Vgll2 PE=1 SV=1
evm.model.scaffold2907.10	37.5052	17.4713	1.10211	0.00621905	sp|Q9H3K2|GHITM_HUMAN Growth hormone-inducible transmembrane protein OS=Homo sapiens GN=GHITM PE=1 SV=2
evm.model.scaffold142869.10	3.28602	0.524518	2.64728	0.00213042	--
evm.model.scaffold146097.53_e	3.42295	1.62438	1.07535	0.00480132	sp|Q8N9F8|ZN454_HUMAN Zinc

vm.model.scaffold146097.54					finger protein 454 OS=Homo sapiens GN=ZNF454 PE=2 SV=2
evm.model.scaffold7467.29	13.0939	5.56823	1.23361	0.000820679	sp|Q2PC20|PPM1K_BOVIN Protein phosphatase 1K, mitochondrial OS=Bos taurus GN=PPM1K PE=2 SV=1
evm.model.scaffold175665.5	118.27	48.7511	1.27858	0.000820679	sp|P76536|YFEX_ECOLI Probable deferrochelatase/peroxidase YfeX OS=Escherichia coli (strain K12) GN=yfeX PE=1 SV=2
evm.model.scaffold147433.6	26.171	16.9695	0.625025	0.0314536	sp|Q921R4|DJC14_MOUSE DnaJ homolog subfamily C member 14 OS=Mus musculus GN=Dnajc14 PE=2 SV=2
evm.model.scaffold27129.3	2.18324	1.04044	1.06928	0.00888556	sp|A0JN53|RPAP1_BOVIN RNA polymerase II-associated protein 1 OS=Bos taurus GN=RPAP1 PE=2 SV=1
evm.model.scaffold42935.29.1	32.6591	19.6504	0.732925	0.0137473	sp|Q92673|SORL_HUMAN Sortilin-related receptor OS=Homo sapiens GN=SORL1 PE=1 SV=2
evm.model.scaffold2735.23	67.437	42.7077	0.659044	0.0195535	sp|B4KD90|NMDA1_DROMO Glutamate [NMDA] receptor subunit 1 OS=Drosophila mojavensis GN=Nmdar1 PE=3 SV=1
evm.model.scaffold117039.3	35.5134	10.2313	1.79538	0.000820679	sp|Q6NSN2|AOF_DANRE Amine oxidase [flavin-containing] OS=Danio

					rerio GN=mao PE=1 SV=1
evm.model.scaffold99641.19	9.55058	4.30489	1.14961	0.00532397	sp|Q8BZP8|NPSR1_MOUSE Neuropeptide S receptor OS=Mus musculus GN=Npsr1 PE=2 SV=1
evm.model.scaffold159927.5	29.4097	12.9391	1.18455	0.000820679	sp|Q9P2T1|GMPR2_HUMAN GMP reductase 2 OS=Homo sapiens GN=GMPR2 PE=1 SV=1
evm.model.scaffold175429.21	12.5211	5.1011	1.29548	0.0101381	sp|Q95M71|BIRC8_GORGO Baculoviral IAP repeat-containing protein 8 OS=Gorilla gorilla gorilla GN=BIRC8 PE=2 SV=1
evm.model.scaffold65937.7	21.8076	12.4923	0.803795	0.0274321	sp|B4F7A1|LYRM7_RAT Complex III assembly factor LYRM7 OS=Rattus norvegicus GN=Lyrm7 PE=3 SV=1
evm.model.scaffold4397.19	27.5741	14.3603	0.941224	0.00213042	sp|Q16891|MIC60_HUMAN MICOS complex subunit MIC60 OS=Homo sapiens GN=IMMT PE=1 SV=1
evm.model.scaffold66661.54	6.99019	3.43747	1.02398	0.0117709	--
evm.model.scaffold148345.7	27.9767	14.6983	0.928581	0.00213042	sp|P23286|CALM_CANAX Calmodulin OS=Candida albicans GN=CMD1 PE=3 SV=2
evm.model.scaffold176619.12.1	49.3413	28.191	0.807564	0.00326205	sp|P31319|KAPR_APLCA cAMP-dependent protein kinase regulatory subunit OS=Aplysia californica PE=2 SV=2

evm.model.scaffold3293.2.1	4.31953	2.49859	0.789762	0.0296885	sp|A1L253|F149B_DANRE Protein FAM149B1 OS=Danio rerio GN=fam149b1 PE=2 SV=1
evm.model.scaffold55277.10	6.35436	1.38075	2.20229	0.00213042	sp|Q8UWA5|CAH2_TRIHK Carbonic anhydrase 2 OS=Tribolodon hakonensis GN=ca2 PE=2 SV=3
evm.model.scaffold132451.9	25.0827	16.0066	0.648025	0.0327195	sp|P0CI75|BIRA_BACSU Bifunctional ligase/repressor BirA OS=Bacillus subtilis (strain 168) GN=birA PE=1 SV=1
evm.model.scaffold76647.2	0.843715	0.264435	1.67384	0.035577	sp|P22607|FGFR3_HUMAN Fibroblast growth factor receptor 3 OS=Homo sapiens GN=FGFR3 PE=1 SV=1
evm.model.scaffold172337.13	57.6714	33.2619	0.793985	0.00429787	--
evm.model.scaffold118449.28	17.9745	6.77848	1.40692	0.0113727	sp|P80276|ALDR_PIG Aldose reductase OS=Sus scrofa GN=AKR1B1 PE=1 SV=2
evm.model.scaffold32083.24	5.00714	2.35022	1.09119	0.0205228	sp|Q5ZLC8|ANR52_CHICK Serine/threonine-protein phosphatase 6 regulatory ankyrin repeat subunit C OS=Gallus gallus GN=ANKRD52 PE=2 SV=1
evm.model.scaffold32083.27	35.0125	15.4104	1.18396	0.000820679	sp|Q8CD19|LANC3_MOUSE LanC-like protein 3 OS=Mus musculus

					GN=Lancl3 PE=2 SV=2
evm.model.scaffold32083.29	60.3252	35.0595	0.782953	0.00480132	sp|Q9W770|SPON1_CHICK Spondin-1 OS=Gallus gallus GN=SPON1 PE=2 SV=1
evm.model.scaffold60115.12	66.9635	39.68	0.754965	0.00578348	--
evm.model.scaffold80593.7	62.7834	38.4766	0.706402	0.00712329	sp|Q12923|PTN13_HUMAN Tyrosine-protein phosphatase non-receptor type 13 OS=Homo sapiens GN=PTPN13 PE=1 SV=2
evm.model.scaffold54263.29	11.6603	5.00188	1.22106	0.000820679	sp|P97831|TWST2_RAT Twist-related protein 2 OS=Rattus norvegicus GN=Twist2 PE=2 SV=2
evm.model.scaffold154521.6	8.69289	5.63381	0.625725	0.0399665	sp|P91620|SIF2_DROME Protein still life, isoforms C/SIF type 2 OS=Drosophila melanogaster GN=sif PE=2 SV=2
evm.model.scaffold94027.1	33.6118	16.5963	1.01811	0.000820679	sp|P36544|ACHA7_HUMAN Neuronal acetylcholine receptor subunit alpha-7 OS=Homo sapiens GN=CHRNA7 PE=1 SV=5
evm.model.scaffold146247.21	8.67492	3.59486	1.27091	0.000820679	sp|P82987|ATL3_HUMAN ADAMTS-like protein 3 OS=Homo sapiens GN=ADAMTSL3 PE=1 SV=4
evm.model.scaffold52185.23	13.6534	7.18559	0.926084	0.000820679	sp|Q2V2K5|GNRHR_OCTVU Gonadotropin-releasing hormone

					receptor OS=Octopus vulgaris GN=GNRHR PE=2 SV=1
evm.model.scaffold110195.7.1	61.1697	34.9188	0.808814	0.00271512	sp|Q63312|PHLB1_RAT Pleckstrin homology-like domain family B member 1 (Fragment) OS=Rattus norvegicus GN=Phldb1 PE=2 SV=2
evm.model.scaffold173227.7	449.112	142.051	1.66067	0.000820679	sp|Q9H987|SYP2L_HUMAN Synaptopodin 2-like protein OS=Homo sapiens GN=SYNPO2L PE=2 SV=3
evm.model.scaffold147663.9	6.51722	3.45346	0.916215	0.0261282	sp|Q32PI8|RT27_BOVIN 28S ribosomal protein S27, mitochondrial OS=Bos taurus GN=MRPS27 PE=1 SV=1
evm.model.scaffold99267.4	30.2685	19.2225	0.655017	0.0494585	sp|Q8BSL4|HS3S5_MOUSE Heparan sulfate glucosamine 3-O-sulfotransferase 5 OS=Mus musculus GN=Hs3st5 PE=2 SV=1
evm.model.scaffold59477.9	27.5243	12.1743	1.17686	0.016685	sp|O76082|S22A5_HUMAN Solute carrier family 22 member 5 OS=Homo sapiens GN=SLC22A5 PE=1 SV=1
evm.model.scaffold30425.9	41.8473	26.1716	0.677134	0.0188471	sp|O55043|ARHG7_RAT Rho guanine nucleotide exchange factor 7 OS=Rattus norvegicus GN=Arhgef7 PE=1 SV=1
evm.model.scaffold170995.22	75.9549	48.073	0.659918	0.0324675	sp|Q28CH2|CPEB3_XENTR

					Cytoplasmic polyadenylation element-binding protein 3 OS=Xenopus tropicalis GN=cpeb3 PE=2 SV=1
evm.model.scaffold169113.5	9.25274	4.28717	1.10985	0.00380231	sp|P46437|GST_MUSDO Glutathione S-transferase OS=Musca domestica PE=2 SV=1
evm.model.scaffold56437.3	35.0204	19.8513	0.818961	0.00712329	sp|Q6NZS4|RTCB_DANRE tRNA-splicing ligase RtcB homolog OS=Danio rerio GN=rtcb PE=2 SV=1
evm.model.scaffold56437.5	91.6023	40.0334	1.19418	0.00213042	sp|P74148|Y1388_SYNY3 Universal stress protein Sll1388 OS=Synechocystis sp. (strain PCC 6803 / Kazusa) GN=sll1388 PE=3 SV=1
evm.model.scaffold119983.4	26.3346	0.942311	4.80461	0.000820679	sp|Q8IUS5|EPHX4_HUMAN Epoxide hydrolase 4 OS=Homo sapiens GN=EPHX4 PE=2 SV=2
evm.model.scaffold169189.17	5.90097	3.19759	0.883969	0.0254597	sp|Q9NTG7|SIR3_HUMAN NAD-dependent protein deacetylase sirtuin-3, mitochondrial OS=Homo sapiens GN=SIRT3 PE=1 SV=2
evm.model.scaffold146043.10	478.965	124.792	1.94039	0.000820679	sp|Q23551|UNC22_CAEEL Twitchin OS=Caenorhabditis elegans GN=unc-22 PE=1 SV=3
evm.model.scaffold146097.26	153.823	88.3075	0.800667	0.00755259	sp|Q6GQD3|RB24A_XENLA

					RNA-binding protein 24-A OS=Xenopus laevis GN=rbm24-a PE=2 SV=1
evm.model.scaffold1779.18	19.1978	11.596	0.727307	0.016685	--
evm.model.scaffold124683.37	31.4684	13.607	1.20955	0.00213042	--
evm.model.scaffold100239.22	52.4172	7.96541	2.71822	0.000820679	--
evm.model.scaffold118787.25	15.956	8.58583	0.894067	0.016685	sp|Q9NWT8|AKIP_HUMAN Aurora kinase A-interacting protein OS=Homo sapiens GN=AURKAIP1 PE=1 SV=1
evm.model.scaffold96499.8	72.9531	41.177	0.825132	0.00800162	sp|Q8WZ42|TITIN_HUMAN Titin OS=Homo sapiens GN=TTN PE=1 SV=4
evm.model.scaffold36941.4	81.8303	51.1285	0.678506	0.0148208	sp|Q13873|BMPR2_HUMAN Bone morphogenetic protein receptor type-2 OS=Homo sapiens GN=BMPR2 PE=1 SV=2
evm.model.scaffold43385.10	0.887234	0.134666	2.71992	0.00972356	sp|Q03526|ITK_MOUSE Tyrosine-protein kinase ITK/TSK OS=Mus musculus GN=Itk PE=1 SV=1
evm.model.scaffold147153.1	93.3613	51.4337	0.860112	0.00271512	sp|O08688|CAN5_MOUSE Calpain-5 OS=Mus musculus GN=Capn5 PE=2 SV=1
evm.model.scaffold34573.4	105.037	26.0072	2.01392	0.000820679	sp|P14618|KPYM_HUMAN Pyruvate kinase PKM OS=Homo sapiens GN=PKM PE=1 SV=4

evm.model.scaffold176133.44	17.188	6.64532	1.371	0.000820679	sp|O97148|MTH_DROME G-protein coupled receptor Mth OS=Drosophila melanogaster GN=mth PE=1 SV=1
evm.model.scaffold116211.17	7.29279	4.11485	0.825631	0.0129481	sp|Q8AVY1|ODF3A_XENLA Outer dense fiber protein 3 OS=Xenopus laevis GN=odf3 PE=2 SV=1
evm.model.scaffold6943.50	19.7777	8.41478	1.23287	0.0361439	sp|Q28107|FA5_BOVIN Coagulation factor V OS=Bos taurus GN=F5 PE=1 SV=1
evm.model.scaffold25605.29	186.166	121.625	0.614148	0.0299214	sp|Q94360|NDUS7_CAEEL Probable NADH dehydrogenase [ubiquinone] iron-sulfur protein 7, mitochondrial OS=Caenorhabditis elegans GN=nduf-7 PE=3 SV=1
evm.model.scaffold126619.7	66.1537	39.8301	0.731962	0.0105331	sp|Q8HXG6|NDUAB_BOVIN NADH dehydrogenase [ubiquinone] 1 alpha subcomplex subunit 11 OS=Bos taurus GN=NDUFA11 PE=1 SV=3
evm.model.scaffold38207.15	8.63719	4.0535	1.09139	0.000820679	sp|Q90YJ2|NGB_DANRE Neuroglobin OS=Danio rerio GN=ngb PE=1 SV=1
evm.model.scaffold165727.6.1	152.022	64.2856	1.24171	0.000820679	sp|P03372|ESR1_HUMAN Estrogen receptor OS=Homo sapiens GN=ESR1 PE=1 SV=2
evm.model.scaffold30535.13	20.0522	3.73448	2.42478	0.000820679	sp|A6NM10|AQ12B_HUMAN

					Aquaporin-12B OS=Homo sapiens GN=AQP12B PE=2 SV=1
evm.model.scaffold153405.13	5.07289	1.60775	1.65776	0.00578348	--
evm.model.scaffold73937.5	10.7691	3.72717	1.53075	0.000820679	sp|Q8SWR3|SPR_DROME Sex peptide receptor OS=Drosophila melanogaster GN=SPR PE=1 SV=1
evm.model.scaffold164773.67	20.9237	8.80443	1.24884	0.000820679	sp|P21251|CALM_STIJA Calmodulin OS=Stichopus japonicus PE=1 SV=2
evm.model.scaffold22223.4.3	7964.12	799.065	3.31713	0.000820679	--
evm.model.scaffold23897.18	15.3671	9.67247	0.667885	0.0404844	sp|Q9UPW0|FOXJ3_HUMAN Forkhead box protein J3 OS=Homo sapiens GN=FOXJ3 PE=1 SV=2
evm.model.scaffold98335.86	1.38397	0	inf	0.0276479	--
evm.model.scaffold32677.13	5.79609	3.22387	0.846284	0.0129481	sp|Q6PGE4|ZF316_MOUSE Zinc finger protein 316 OS=Mus musculus GN=Znf316 PE=2 SV=1
evm.model.scaffold104629.3	12.8679	7.22905	0.831903	0.00755259	sp|O35857|TIM44_MOUSE Mitochondrial import inner membrane translocase subunit TIM44 OS=Mus musculus GN=Timm44 PE=1 SV=2
evm.model.scaffold176361.16	41.6413	23.7311	0.81124	0.0117709	sp|Q80SY4|MIB1_MOUSE E3 ubiquitin-protein ligase MIB1 OS=Mus musculus GN=Mib1 PE=1 SV=1
evm.model.scaffold134329.9	5.29373	1.36599	1.95434	0.000820679	sp|Q8N9V6|ANR53_HUMAN Ankyrin repeat domain-containing

					protein 53 OS=Homo sapiens GN=ANKRD53 PE=2 SV=3
evm.model.scaffold93679.62	47.7535	23.2831	1.03632	0.000820679	sp|B4PZ52|NFU1_DROYA NFU1 iron-sulfur cluster scaffold homolog, mitochondrial OS=Drosophila yakuba GN=GE15286 PE=3 SV=1
evm.model.scaffold145865.10	2.26988	1.04398	1.12052	0.0318917	sp|Q6P8Y1|CAPSL_MOUSE Calcyphosin-like protein OS=Mus musculus GN=Capsl PE=2 SV=4
evm.model.scaffold71355.7	13.8051	5.75319	1.26277	0.000820679	sp|Q9JJK2|LANC2_MOUSE LanC-like protein 2 OS=Mus musculus GN=Lancl2 PE=1 SV=1
evm.model.scaffold20377.12	62.7206	37.7812	0.731273	0.00621905	sp|Q5NVR2|MDHM_PONAB Malate dehydrogenase, mitochondrial OS=Pongo abelii GN=MDH2 PE=2 SV=1
evm.model.scaffold162271.2	70.9088	42.7737	0.729241	0.013343	sp|P30622|CLIP1_HUMAN CAP-Gly domain-containing linker protein 1 OS=Homo sapiens GN=CLIP1 PE=1 SV=2
evm.model.scaffold162271.3	25.2853	12.9553	0.964758	0.000820679	sp|Q17DK5|CRY1_AEDAE Cryptochrome-1 OS=Aedes aegypti GN=cry PE=3 SV=1
evm.model.scaffold158547.61	120.513	69.49	0.794312	0.00532397	sp|P40423|SQH_DROME Myosin regulatory light chain sqh

					OS=Drosophila melanogaster GN=sqh PE=1 SV=1
evm.model.scaffold142905.54	66.9895	23.2313	1.52787	0.000820679	sp|Q9Z0E8|S22A5_MOUSE Solute carrier family 22 member 5 OS=Mus musculus GN=Slc22a5 PE=1 SV=1
evm.model.scaffold138629.150	2.05863	0.678213	1.60187	0.000820679	sp|Q6KEQ9|PC11X_PIG Protocadherin-11 X-linked OS=Sus scrofa GN=PCDH11X PE=2 SV=1
evm.model.scaffold4289.11	55.8476	23.3038	1.26093	0.000820679	sp|P0CR42|SET5_CRYNJ Potential protein lysine methyltransferase SET5 OS=Cryptococcus neoformans var. neoformans serotype D (strain JEC21 / ATCC MYA-565) GN=SET5 PE=3 SV=1
evm.model.scaffold121031.37	187.569	49.1101	1.93333	0.000820679	sp|Q6NVA9|ACTB_XENTR Actin, cytoplasmic 1 OS=Xenopus tropicalis GN=actb PE=2 SV=1
evm.model.scaffold121031.36	2.29271	0.651405	1.81543	0.027885	sp|Q93129|ACTC_BRABE Actin, cytoplasmic OS=Branchiostoma belcheri PE=2 SV=1
evm.model.scaffold121031.38	21294.8	3483.72	2.6118	0.000820679	sp|Q93129|ACTC_BRABE Actin, cytoplasmic OS=Branchiostoma belcheri PE=2 SV=1
evm.model.scaffold106559.11	12.878	4.16828	1.62738	0.000820679	sp|Q99LB6|MAT2B_MOUSE Methionine adenosyltransferase 2 subunit beta OS=Mus musculus

					GN=Mat2b PE=2 SV=1
evm.model.scaffold15403.28	62.3799	31.81	0.971601	0.000820679	sp|O75052|CAPON_HUMAN Carboxyl-terminal PDZ ligand of neuronal nitric oxide synthase protein OS=Homo sapiens GN=NOS1AP PE=1 SV=3
evm.model.scaffold15119.4	15.0876	7.08215	1.0911	0.000820679	sp|Q60HE9|MA2B1_MACFA Lysosomal alpha-mannosidase OS=Macaca fascicularis GN=MAN2B1 PE=2 SV=1
evm.model.scaffold15119.3	13.0814	6.3064	1.05263	0.00578348	sp|Q8VHC8|MA2B1_CAVPO Lysosomal alpha-mannosidase OS=Cavia porcellus GN=MAN2B1 PE=1 SV=1
evm.model.scaffold100379.30	21.0775	12.0554	0.806022	0.00755259	sp|Q24423|NOC_DROME Zinc finger protein Noc OS=Drosophila melanogaster GN=noc PE=1 SV=1
evm.model.scaffold56755.58	22.6242	9.5835	1.23924	0.000820679	sp|P78504|JAG1_HUMAN Protein jagged-1 OS=Homo sapiens GN=JAG1 PE=1 SV=3
evm.model.scaffold81801.14	43.3399	25.4931	0.765592	0.00670086	sp|Q92508|PIEZ1_HUMAN Piezo-type mechanosensitive ion channel component 1 OS=Homo sapiens GN=PIEZO1 PE=1 SV=4
evm.model.scaffold136775.13	15.2562	9.90705	0.622868	0.0437576	sp|O97571|CXCR2_CANFA C-X-C

					chemokine receptor type 2 OS=Canis familiaris GN=CXCR2 PE=2 SV=1
evm.model.scaffold136775.11	50.9279	13.2797	1.93924	0.000820679	sp|P53453|DRD2L_TAKRU D(2)-like dopamine receptor OS=Takifugu rubripes GN=d215 PE=3 SV=1
evm.model.scaffold107627.10	33.9721	21.4283	0.66483	0.0205228	--
evm.model.scaffold152989.22	312.556	170.803	0.871784	0.00800162	sp|Q76LL6|FHOD3_MOUSE FH1/FH2 domain-containing protein 3 OS=Mus musculus GN=Fhod3 PE=1 SV=1
evm.model.scaffold152989.23	251.344	150.251	0.742291	0.0148208	sp|Q2V2M9|FHOD3_HUMAN FH1/FH2 domain-containing protein 3 OS=Homo sapiens GN=FHOD3 PE=1 SV=2
evm.model.scaffold152989.24	391.705	229.248	0.772857	0.00842602	sp|Q2V2M9|FHOD3_HUMAN FH1/FH2 domain-containing protein 3 OS=Homo sapiens GN=FHOD3 PE=1 SV=2
evm.model.scaffold162731.25	49.6087	29.3101	0.759198	0.00429787	sp|Q3SWY2|ILK_BOVIN Integrin-linked protein kinase OS=Bos taurus GN=ILK PE=2 SV=1
evm.model.scaffold77121.12	11.1572	6.50995	0.777257	0.0129481	--
evm.model.scaffold111921.42	332.065	64.1811	2.37124	0.000820679	sp|Q57997|Y577_METJA Universal stress protein MJ0577 OS=Methanocaldococcus jannaschii

					(strain ATCC 43067 / DSM 2661 / JAL-1 / JCM 10045 / NBRC 100440) GN=MJ0577 PE=1 SV=1
evm.model.scaffold124269.103	39.6999	22.1887	0.839311	0.00326205	sp|Q9WV92|E41L3_MOUSE Band 4.1-like protein 3 OS=Mus musculus GN=Epb41l3 PE=1 SV=1
evm.model.scaffold119783.1	11.7861	5.82022	1.01794	0.00271512	sp|P51799|CLCN7_RAT H(+)/Cl(-) exchange transporter 7 OS=Rattus norvegicus GN=Clcn7 PE=2 SV=1
evm.model.scaffold78215.36	11.1768	5.49223	1.02504	0.00151774	sp|Q96CX2|KCD12_HUMAN BTB/POZ domain-containing protein KCTD12 OS=Homo sapiens GN=KCTD12 PE=1 SV=1
evm.model.scaffold142393.79	7.59589	3.65016	1.05726	0.000820679	sp|Q62210|BIRC2_MOUSE Baculoviral IAP repeat-containing protein 2 OS=Mus musculus GN=Birc2 PE=1 SV=1
evm.model.scaffold132179.2	24.8245	10.1512	1.29011	0.000820679	sp|P26150|3BHS3_MOUSE 3 beta-hydroxysteroid dehydrogenase/Delta 5-->4-isomerase type 3 OS=Mus musculus GN=Hsd3b3 PE=2 SV=3
evm.model.scaffold132179.7	33.9005	17.5087	0.953232	0.00578348	sp|Q2KIK3|SIM14_BOVIN Small integral membrane protein 14 OS=Bos taurus GN=SMIM14 PE=3 SV=1

evm.model.scaffold176119.79	400.385	207.398	0.948984	0.00532397	sp|Q27889|PP2B2_DROME Serine/threonine-protein phosphatase 2B catalytic subunit 2 OS=Drosophila melanogaster GN=Pp2B-14D PE=1 SV=2
evm.model.scaffold176119.71	32.7001	10.5399	1.63343	0.000820679	--
evm.model.scaffold176119.72	34.9186	19.5341	0.838	0.00151774	sp|D3YZP9|CCDC6_MOUSE Coiled-coil domain-containing protein 6 OS=Mus musculus GN=Ccdc6 PE=1 SV=1
evm.model.scaffold8273.13	4.77829	2.27651	1.06967	0.00532397	sp|Q6PII3|CC174_HUMAN Coiled-coil domain-containing protein 174 OS=Homo sapiens GN=CCDC174 PE=2 SV=3
evm.model.scaffold169321.5	22.1299	8.86681	1.31951	0.000820679	sp|Q5PQN9|RM38_RAT 39S ribosomal protein L38, mitochondrial OS=Rattus norvegicus GN=Mrpl38 PE=2 SV=2
evm.model.scaffold176577.33	227.16	92.4445	1.29705	0.000820679	sp|Q9HBL0|TENS1_HUMAN Tensin-1 OS=Homo sapiens GN=TNS1 PE=1 SV=2
evm.model.scaffold164239.19	81.1758	30.9423	1.39147	0.000820679	--
evm.model.scaffold136529.6	48.1129	21.6928	1.14921	0.000820679	sp|Q08C93|ABD12_DANRE Monoacylglycerol lipase ABHD12 OS=Danio rerio GN=abhd12 PE=2

					SV=1
evm.model.scaffold10799.8	2.10197	0.445635	2.23781	0.00670086	sp|A3KG59|P20D2_MOUSE Peptidase M20 domain-containing protein 2 OS=Mus musculus GN=Pm20d2 PE=2 SV=1
evm.model.scaffold51225.72	13.319	5.95533	1.16123	0.000820679	--
evm.model.scaffold76817.45	2.53418	0.674974	1.90861	0.000820679	--
evm.model.scaffold73223.15	127.131	53.0429	1.26109	0.000820679	sp|Q32PF3|PCNP_BOVIN PEST proteolytic signal-containing nuclear protein OS=Bos taurus GN=PCNP PE=2 SV=1
evm.model.scaffold116119.121	11.0582	5.80904	0.928747	0.00326205	sp|Q99814|EPAS1_HUMAN Endothelial PAS domain-containing protein 1 OS=Homo sapiens GN=EPAS1 PE=1 SV=3
evm.model.scaffold174971.4	211.812	55.3881	1.93513	0.000820679	sp|Q9D6P8|CALL3_MOUSE Calmodulin-like protein 3 OS=Mus musculus GN=Calml3 PE=2 SV=1
evm.model.scaffold151129.1	36.4724	6.31475	2.53001	0.000820679	sp|Q5F364|MRP1_CHICK Multidrug resistance-associated protein 1 OS=Gallus gallus GN=ABCC1 PE=2 SV=1
evm.model.scaffold81473.11	3.54246	0.869113	2.02714	0.000820679	sp|P18173|DHGL_DROME Glucose dehydrogenase [FAD, quinone] OS=Drosophila melanogaster GN=Gld

					PE=3 SV=3
evm.model.scaffold142869.41	45.8947	5.07986	3.17547	0.000820679	sp|Q9VCA2|ORCT_DROME Organic cation transporter protein OS=Drosophila melanogaster GN=Orct PE=1 SV=1
evm.model.scaffold82057.56	1.90866	0.830493	1.20052	0.0228478	sp|Q99741|CDC6_HUMAN Cell division control protein 6 homolog OS=Homo sapiens GN=CDC6 PE=1 SV=1
evm.model.scaffold50375.1	13.8564	7.97891	0.796289	0.00842602	sp|A9CB25|S22A4_PAPAN Solute carrier family 22 member 4 OS=Papio anubis GN=SLC22A4 PE=3 SV=1
evm.model.scaffold176409.6.1	1.56796	0.669759	1.22718	0.0396592	sp|Q8R216|SIR4_MOUSE NAD-dependent protein lipoamidase sirtuin-4, mitochondrial OS=Mus musculus GN=Sirt4 PE=1 SV=3
evm.model.scaffold90899.77	10.0605	1.23396	3.02733	0.000820679	sp|Q8BZG5|RRNAD_MOUSE Protein RRNAD1 OS=Mus musculus GN=Rrnad1 PE=2 SV=1
evm.model.scaffold132315.44	85.4652	32.7549	1.38363	0.000820679	sp|Q63421|PDE1C_RAT Calcium/calmodulin-dependent 3',5'-cyclic nucleotide phosphodiesterase 1C OS=Rattus norvegicus GN=Pde1c PE=2 SV=1
evm.model.scaffold16445.63	3246.4	450.859	2.84809	0.000820679	sp|Q5XFX0|TAGL2_RAT Transgelin-2

					OS=Rattus norvegicus GN=Tagln2 PE=2 SV=1
evm.model.scaffold16445.67	65.1366	39.2343	0.731353	0.00972356	sp|Q6AXM9|CL029_RAT Uncharacterized protein C12orf29 homolog OS=Rattus norvegicus PE=2 SV=2
evm.model.scaffold167889.16	169.877	101.495	0.743074	0.00755259	sp|O61492|FLOT2_DROME Flotillin-2 OS=Drosophila melanogaster GN=Flo-2 PE=2 SV=3
evm.model.scaffold175495.49	17.83	8.81394	1.01645	0.000820679	sp|P47934|CACP_MOUSE Carnitine O-acetyltransferase OS=Mus musculus GN=Crat PE=1 SV=3
evm.model.scaffold65455.5	85.6499	6.63689	3.68987	0.000820679	sp|Q8VDT1|SC5A9_MOUSE Sodium/glucose cotransporter 4 OS=Mus musculus GN=Slc5a9 PE=2 SV=3
evm.model.scaffold5455.6	485.748	304.476	0.673881	0.0177206	sp|Q20655|14332_CAEEL 14-3-3-like protein 2 OS=Caenorhabditis elegans GN=ftt-2 PE=1 SV=1
evm.model.scaffold19571.10	10.6631	5.22019	1.03045	0.00621905	sp|Q80TE4|SI1L2_MOUSE Signal-induced proliferation-associated 1-like protein 2 OS=Mus musculus GN=Sipa1l2 PE=1 SV=3
evm.model.scaffold170291.2	91.2908	56.6806	0.687614	0.0137473	sp|O15344|TRI18_HUMAN E3

					ubiquitin-protein ligase Midline-1 OS=Homo sapiens GN=MID1 PE=1 SV=1
evm.model.scaffold128949.5	260.044	80.0352	1.70005	0.000820679	--
evm.model.scaffold128949.6	2.38524	0.60838	1.97109	0.000820679	sp|P40189|IL6RB_HUMAN Interleukin-6 receptor subunit beta OS=Homo sapiens GN=IL6ST PE=1 SV=2
evm.model.scaffold163655.3	4.03624	0.486104	3.05367	0.00151774	sp|P86725|UP6_HALAI Uncharacterized protein 6 OS=Haliotis asinina PE=1 SV=1
evm.model.scaffold124911.26	12.1478	5.96277	1.02664	0.0125549	--
evm.model.scaffold124911.27	168.751	58.1227	1.53772	0.000820679	sp|O08730|GLYG_RAT Glycogenin-1 OS=Rattus norvegicus GN=Gyg1 PE=2 SV=4
evm.model.scaffold52321.53	51.617	16.7246	1.62587	0.000820679	--
evm.model.scaffold27973.9.1	26.0948	14.5594	0.841815	0.00151774	sp|Q07722|PLCB4_BOVIN 1-phosphatidylinositol 4,5-bisphosphate phosphodiesterase beta-4 (Fragment) OS=Bos taurus GN=PLCB4 PE=1 SV=1
evm.model.scaffold48043.2	2.03613	0.772471	1.39828	0.0169923	sp|P28658|ATX10_MOUSE Ataxin-10 OS=Mus musculus GN=Atxn10 PE=1 SV=2
evm.model.scaffold10785.19	24.2039	13.9436	0.795637	0.0314536	sp|Q9QYG6|CP2DR_MESAU

					Cytochrome P450 2D27 OS=Mesocricetus auratus GN=CYP2D27 PE=1 SV=1
evm.model.scaffold3565.5	4.18516	2.32921	0.845442	0.0420901	sp|Q5ZJ66|SYEM_CHICK Probable glutamate--tRNA ligase, mitochondrial OS=Gallus gallus GN=EARS2 PE=2 SV=1
evm.model.scaffold135119.48	23.9255	4.89503	2.28916	0.000820679	sp|Q8UWA5|CAH2_TRIHK Carbonic anhydrase 2 OS=Tribolodon hakonensis GN=ca2 PE=2 SV=3
evm.model.scaffold20967.22	16.3057	10.3019	0.662459	0.044934	sp|Q6P371|HOT_XENTR Hydroxyacid-oxoacid transhydrogenase, mitochondrial OS=Xenopus tropicalis GN=adhfe1 PE=2 SV=1
evm.model.scaffold94547.2	5.92829	3.50804	0.756952	0.044934	sp|P62510|ERR3_RAT Estrogen-related receptor gamma OS=Rattus norvegicus GN=Esrrg PE=2 SV=1
evm.model.scaffold72185.31	39.0921	24.7	0.662368	0.0457321	--
evm.model.scaffold14517.27	133.04	73.8204	0.849765	0.00213042	sp|P07552|QCR10_BOVIN Cytochrome b-c1 complex subunit 10 OS=Bos taurus GN=UQCR11 PE=1 SV=2
evm.model.scaffold14517.20	9.21814	5.54028	0.734515	0.0152063	sp|O95263|PDE8B_HUMAN High

					affinity cAMP-specific and IBMX-insensitive 3',5'-cyclic phosphodiesterase 8B OS=Homo sapiens GN=PDE8B PE=1 SV=2
evm.model.scaffold108283.4	58.748	33.5428	0.808535	0.00532397	sp|A0LMC1|PHNX_SYNFM Phosphonoacetaldehyde hydrolase OS=Syntrophobacter fumaroxidans (strain DSM 10017 / MPOB) GN=phnX PE=3 SV=1
evm.model.scaffold128557.1	10.9228	6.13807	0.831489	0.00933293	--
evm.model.scaffold138369.1	0.635412	0	inf	0.000820679	--
evm.model.scaffold148433.11	32.8299	19.7332	0.734383	0.0105331	sp|Q6NTV6|INF2_XENLA Inverted formin-2 OS=Xenopus laevis GN=inf2 PE=2 SV=1
evm.model.scaffold166273.6	337.693	80.1481	2.07497	0.000820679	--
evm.model.scaffold166273.5	442.66	163.216	1.43942	0.000820679	--
evm.model.scaffold91387.8_evm.model.scaffold91387.7	24.3669	13.0591	0.899869	0.000820679	sp|Q6ZV89|SH2D5_HUMAN SH2 domain-containing protein 5 OS=Homo sapiens GN=SH2D5 PE=1 SV=2
evm.model.scaffold30917.3	34.6793	16.1555	1.10205	0.000820679	sp|P16157|ANK1_HUMAN Ankyrin-1 OS=Homo sapiens GN=ANK1 PE=1 SV=3
evm.model.scaffold146623.42	19.1553	7.12067	1.42766	0.000820679	sp|P18762|ADRB2_MOUSE Beta-2 adrenergic receptor OS=Mus musculus

					GN=Adrb2 PE=2 SV=2
evm.model.scaffold68361.18	5.40692	3.34059	0.694705	0.0404844	sp|B0W2S0|CLU_CULQU Clustered mitochondria protein homolog OS=Culex quinquefasciatus GN=CPIJ001445 PE=3 SV=1
evm.model.scaffold115633.4	25.7044	14.3917	0.836772	0.0177206	sp|Q8BHY3|ANO1_MOUSE Anoctamin-1 OS=Mus musculus GN=Ano1 PE=1 SV=2
evm.model.scaffold115633.5	21.4874	11.6284	0.885837	0.00480132	sp|Q5XXA6|ANO1_HUMAN Anoctamin-1 OS=Homo sapiens GN=ANO1 PE=1 SV=1
evm.model.scaffold115633.9	4.02929	0.380426	3.40484	0.00380231	--
evm.model.scaffold47967.26	29.7695	18.8957	0.655771	0.0429196	sp|Q7ZVY5|CISY_DANRE Citrate synthase, mitochondrial OS=Danio rerio GN=cs PE=2 SV=1
evm.model.scaffold158263.5	124.183	39.0897	1.66761	0.000820679	sp|P34284|YKK7_CAEEL Uncharacterized F-box/LRR-repeat protein C02F5.7 OS=Caenorhabditis elegans GN=C02F5.7 PE=3 SV=3
evm.model.scaffold115551.1_evm.model.scaffold115551.2	37.0202	12.5133	1.56485	0.000820679	sp|P91931|DCAM_DROME S-adenosylmethionine decarboxylase proenzyme OS=Drosophila melanogaster GN=SamDC PE=2 SV=1
evm.model.scaffold80479.21	13.1303	4.32536	1.602	0.000820679	sp|Q93YS4|AB22G_ARATH ABC transporter G family member 22

					OS=Arabidopsis thaliana GN=ABCG22 PE=1 SV=1
evm.model.scaffold164745.23	9.19916	5.12633	0.843576	0.00480132	sp|P56719|OX2R_RAT Orexin receptor type 2 OS=Rattus norvegicus GN=Hcrtr2 PE=2 SV=1
evm.model.scaffold144319.6	57.0071	22.8988	1.31587	0.000820679	sp|P31637|SC5A3_CANFA Sodium/myo-inositol cotransporter OS=Canis familiaris GN=SLC5A3 PE=2 SV=1
evm.model.scaffold115227.35	27.92	14.9023	0.905765	0.00151774	sp|Q80VJ2|SRA1_MOUSE Steroid receptor RNA activator 1 OS=Mus musculus GN=Sra1 PE=1 SV=3
evm.model.scaffold161513.61	26.1628	15.6751	0.739041	0.00972356	sp|Q9VFC8|GYS_DROME Glycogen [starch] synthase OS=Drosophila melanogaster GN=GlyS PE=1 SV=2
evm.model.scaffold49899.6	26.8701	17.4847	0.619911	0.0420901	sp|A0JMV4|RBM5A_XENLA RNA-binding protein 5-A OS=Xenopus laevis GN=rbm5-a PE=2 SV=1
evm.model.scaffold118787.16	252.645	83.2639	1.60135	0.000820679	sp|P47843|GTR3_SHEEP Solute carrier family 2, facilitated glucose transporter member 3 OS=Ovis aries GN=SLC2A3 PE=2 SV=1
evm.model.scaffold146755.44	331.968	110.924	1.58147	0.000820679	sp|O70433|FHL2_MOUSE Four and a half LIM domains protein 2 OS=Mus

					musculus GN=Fhl2 PE=1 SV=1
evm.model.scaffold66661.28	15.0543	8.32769	0.854187	0.00712329	sp|Q17QH8|D39U1_BOVIN Epimerase family protein SDR39U1 OS=Bos taurus GN=SDR39U1 PE=2 SV=1
evm.model.scaffold66661.29	20.9567	13.1062	0.677161	0.0205228	sp|Q9R049|AMFR_MOUSE E3 ubiquitin-protein ligase AMFR OS=Mus musculus GN=Amfr PE=1 SV=2
evm.model.scaffold22011.17	38.0302	20.2625	0.908333	0.000820679	sp|Q9D0L6|BAMBI_MOUSE BMP and activin membrane-bound inhibitor homolog OS=Mus musculus GN=Bambi PE=2 SV=1
evm.model.scaffold22011.19	50.3394	29.5659	0.767752	0.00480132	sp|Q13145|BAMBI_HUMAN BMP and activin membrane-bound inhibitor homolog OS=Homo sapiens GN=BAMBI PE=1 SV=1
evm.model.scaffold176379.13	18.6259	11.5798	0.685693	0.0163478	sp|Q8IVL0|NAV3_HUMAN Neuron navigator 3 OS=Homo sapiens GN=NAV3 PE=1 SV=3
evm.model.scaffold78673.9	36.3709	20.7179	0.811903	0.00621905	--
evm.model.scaffold78673.1	11.9869	7.24156	0.727089	0.0141107	sp|Q9UPZ6|THS7A_HUMAN Thrombospondin type-1 domain-containing protein 7A OS=Homo sapiens GN=THSD7A PE=1

					SV=4
evm.model.scaffold3243.24	66.5562	19.1477	1.7974	0.000820679	sp|Q7K1V5|JAGN_DROME Protein jagunal OS=Drosophila melanogaster GN=jagn PE=1 SV=1
evm.model.scaffold170995.8	54.8678	22.4845	1.28703	0.000820679	sp|O70258|SGCE_MOUSE Epsilon-sarcoglycan OS=Mus musculus GN=Sgce PE=1 SV=2
evm.model.scaffold170995.7	104.93	50.4137	1.05754	0.000820679	sp|Q9SE95|FIP2_ARATH FH protein interacting protein FIP2 OS=Arabidopsis thaliana GN=FIP2 PE=1 SV=1
evm.model.scaffold136409.15	7.29903	1.66555	2.13171	0.000820679	sp|Q6GQJ7|SYRM_XENLA Probable arginine--tRNA ligase, mitochondrial OS=Xenopus laevis GN=rars2 PE=2 SV=1
evm.model.scaffold136409.16	58.5548	34.8573	0.748326	0.00712329	sp|P08907|AATM_HORSE Aspartate aminotransferase, mitochondrial OS=Equus caballus GN=GOT2 PE=1 SV=1
evm.model.scaffold151467.169	40.9811	25.463	0.686557	0.0117709	sp|Q58EX7|PKHG4_HUMAN Puratrophin-1 OS=Homo sapiens GN=PLEKHG4 PE=1 SV=1
evm.model.scaffold124269.82	2.35472	1.15937	1.02222	0.027885	sp|P00521|ABL_MLVAB Tyrosine-protein kinase transforming protein Abl OS=Abelson murine

					leukemia virus GN=ABL PE=3 SV=1
evm.model.scaffold137173.18	21.9744	11.5288	0.930577	0.00380231	sp|Q6DFQ7|TM198_XENTR Transmembrane protein 198 OS=Xenopus tropicalis GN=tmem198 PE=1 SV=1
evm.model.scaffold72763.118	2.85421	1.49173	0.936099	0.0137473	sp|Q3MHZ2|SPSB3_BOVIN SPRY domain-containing SOCS box protein 3 OS=Bos taurus GN=SPSB3 PE=2 SV=1
evm.model.scaffold99441.3	7.02769	1.49394	2.23393	0.000820679	sp|P87132|YFK5_SCHPO Uncharacterized protein C167.05 OS=Schizosaccharomyces pombe (strain 972 / ATCC 24843) GN=SPAC167.05 PE=1 SV=2
evm.model.scaffold247.12	2665.93	719.923	1.88872	0.000820679	sp|P02466|CO1A2_RAT Collagen alpha-2(I) chain OS=Rattus norvegicus GN=Col1a2 PE=1 SV=3
evm.model.scaffold247.10	2269.95	680.954	1.73703	0.00213042	sp|Q01149|CO1A2_MOUSE Collagen alpha-2(I) chain OS=Mus musculus GN=Col1a2 PE=2 SV=2
evm.model.scaffold247.19	1233.86	375.69	1.71556	0.000820679	--
evm.model.scaffold24225.3	20.7689	13.1077	0.664014	0.0231466	sp|P23825|GATA3_CHICK GATA-binding factor 3 OS=Gallus gallus GN=GATA3 PE=2 SV=1
evm.model.scaffold3113.10	61.4613	36.9635	0.733576	0.00888556	sp|Q9V9J3|SRC42_DROME Tyrosine-protein kinase Src42A

					OS=Drosophila melanogaster GN=Src42A PE=2 SV=1
evm.model.scaffold58357.10	8.11286	3.41116	1.24995	0.00933293	sp|Q7SYI5|C1GTB_DANRE Glycoprotein-N-acetylgalactosamine 3-beta-galactosyltransferase 1-B OS=Danio rerio GN=c1galt1b PE=2 SV=1
evm.model.scaffold112763.27	6.30735	1.65861	1.92706	0.000820679	sp|Q8CI59|STEA3_MOUSE Metalloreductase STEAP3 OS=Mus musculus GN=Steap3 PE=1 SV=1
evm.model.scaffold100629.11	15.1018	8.84446	0.771874	0.0109379	sp|Q8INF0|GCY8E_DROME Soluble guanylate cyclase 88E OS=Drosophila melanogaster GN=Gyc88E PE=1 SV=3
evm.model.scaffold42227.1	99.083	49.4314	1.00321	0.00151774	sp|Q96RL7|VP13A_HUMAN Vacuolar protein sorting-associated protein 13A OS=Homo sapiens GN=VPS13A PE=1 SV=2
evm.model.scaffold175741.11_evm.model.scaffold175741.8	16.8101	9.36256	0.844354	0.000820679	sp|P23508|CRCM_HUMAN Colorectal mutant cancer protein OS=Homo sapiens GN=MCC PE=1 SV=2
evm.model.scaffold123009.55.1	297.467	147.701	1.01005	0.0117709	sp|Q00963|SPTCB_DROME Spectrin beta chain OS=Drosophila melanogaster GN=beta-Spec PE=1 SV=2

evm.model.scaffold16245.7	2.07879	0.974477	1.09304	0.0254597	sp|Q80VP2|SPAT7_MOUSE Spermatogenesis-associated protein 7 homolog OS=Mus musculus GN=Spata7 PE=1 SV=1
evm.model.scaffold164745.4	358.62	40.118	3.16013	0.000820679	sp|Q6DG32|S2536_DANRE Solute carrier family 25 member 36-A OS=Danio rerio GN=slc25a36a PE=2 SV=1
evm.model.scaffold51225.82	38.4089	9.17096	2.0663	0.000820679	sp|Q17JQ7|CTLH1_AEDAE CTL-like protein 1 OS=Aedes aegypti GN=AAEL001935 PE=3 SV=1
evm.model.scaffold51225.83	35.0685	11.7876	1.5729	0.000820679	sp|Q6IR74|CTL1_XENLA Choline transporter-like protein 1 OS=Xenopus laevis GN=slc44a1 PE=2 SV=2
evm.model.scaffold146401.1	3.18502	1.38921	1.19703	0.0451727	sp|A4IGL7|PXDN_XENTR Peroxidasin OS=Xenopus tropicalis GN=pxdn PE=2 SV=1
evm.model.scaffold82395.20.1	212.858	127.608	0.738178	0.0141107	sp|Q9NQC3|RTN4_HUMAN Reticulon-4 OS=Homo sapiens GN=RTN4 PE=1 SV=2
evm.model.scaffold150473.5	75.3081	27.8019	1.43762	0.000820679	sp|P52899|ODPA_CAEEL Probable pyruvate dehydrogenase E1 component subunit alpha, mitochondrial OS=Caenorhabditis elegans GN=T05H10.6 PE=3 SV=1

evm.model.scaffold149755.20	7.6846	2.054	1.90353	0.000820679	--
evm.model.scaffold124003.1	3.25719	1.28612	1.3406	0.014476	sp|Q8N159|NAGS_HUMAN N-acetylglutamate synthase, mitochondrial OS=Homo sapiens GN=NAGS PE=1 SV=1
evm.model.scaffold154539.56	28.4568	17.8581	0.672191	0.0327195	sp|Q640V2|RMD5A_XENTR Protein RMD5 homolog A OS=Xenopus tropicalis GN=rmnd5a PE=2 SV=1
evm.model.scaffold147445.10	343.197	116.855	1.55432	0.000820679	--
evm.model.scaffold147445.11	75.3822	36.5217	1.04547	0.000820679	sp|Q16720|AT2B3_HUMAN Plasma membrane calcium-transporting ATPase 3 OS=Homo sapiens GN=ATP2B3 PE=1 SV=3
evm.model.scaffold41199.20	7.27769	3.3272	1.12917	0.00429787	sp|P56719|OX2R_RAT Orexin receptor type 2 OS=Rattus norvegicus GN=Hcrtr2 PE=2 SV=1
evm.model.scaffold88753.63	1.6104	0	inf	0.0457321	sp|P86854|PLCL_MYTGA Perlucin-like protein OS=Mytilus galloprovincialis PE=1 SV=1
evm.model.scaffold80593.12	2.12257	1.07675	0.979129	0.0308116	sp|O94812|BAIP3_HUMAN BAI1-associated protein 3 OS=Homo sapiens GN=BAIAP3 PE=1 SV=2
evm.model.scaffold143135.35	167.305	75.8983	1.14035	0.000820679	sp|O95835|LATS1_HUMAN Serine/threonine-protein kinase LATS1 OS=Homo sapiens GN=LATS1 PE=1

					SV=1
evm.model.scaffold150821.75	14.3959	7.63451	0.915047	0.00621905	sp|Q96HR3|MED30_HUMAN Mediator of RNA polymerase II transcription subunit 30 OS=Homo sapiens GN=MED30 PE=1 SV=1
evm.model.scaffold56671.3	15.7454	6.48333	1.28012	0.000820679	sp|Q04446|GLGB_HUMAN 1,4-alpha-glucan-branching enzyme OS=Homo sapiens GN=GBE1 PE=1 SV=3
evm.model.scaffold75347.13.1	41.7823	22.9385	0.865121	0.00213042	sp|P29503|NEUR_DROME Protein neuralized OS=Drosophila melanogaster GN=neur PE=1 SV=2
evm.model.scaffold65307.20	24.6595	10.6188	1.21553	0.000820679	sp|A7YVD7|NDUF6_BOVIN NADH dehydrogenase (ubiquinone) complex I, assembly factor 6 OS=Bos taurus GN=NDUFAF6 PE=2 SV=1
evm.model.scaffold51139.23	41.4994	22.8666	0.859849	0.000820679	sp|P25162|ACH4_DROME Acetylcholine receptor subunit beta-like 2 OS=Drosophila melanogaster GN=nAChRbeta2 PE=2 SV=3
evm.model.scaffold172313.7	14.1398	8.41652	0.748469	0.0184892	sp|Q96SW2|CRBN_HUMAN Protein cereblon OS=Homo sapiens GN=CRBN PE=1 SV=1
evm.model.scaffold142385.2	11.1639	5.29957	1.07489	0.000820679	sp|P56719|OX2R_RAT Orexin

					receptor type 2 OS=Rattus norvegicus GN=Hcrtr2 PE=2 SV=1
evm.model.scaffold119983.58	46.8091	24.2237	0.950367	0.000820679	sp|Q0VCA2|ARRD3_BOVIN Arrestin domain-containing protein 3 OS=Bos taurus GN=ARRDC3 PE=2 SV=1
evm.model.scaffold176133.15	329.738	182.478	0.853598	0.00151774	sp|Q5XG64|EMRE_XENLA Essential MCU regulator, mitochondrial OS=Xenopus laevis GN=smdt1 PE=3 SV=1
evm.model.scaffold22505.8	301.453	114.823	1.39253	0.000820679	sp|Q5F3N1|PIMT_CHICK Protein-L-isoaspartate(D-aspartate) O-methyltransferase OS=Gallus gallus GN=PCMT1 PE=2 SV=3
evm.model.scaffold104023.9	131.82	71.6208	0.880116	0.0241881	sp|Q9BW72|HIG2A_HUMAN HIG1 domain family member 2A, mitochondrial OS=Homo sapiens GN=HIGD2A PE=1 SV=1
evm.model.scaffold104023.4	133.95	31.7921	2.07495	0.000820679	sp|P70694|DHB5_MOUSE Estradiol 17 beta-dehydrogenase 5 OS=Mus musculus GN=Akr1c6 PE=1 SV=1
evm.model.scaffold175645.80	27.1201	10.2499	1.40375	0.00800162	sp|O18778|PAHX_BOVIN Phytanoyl-CoA dioxygenase, peroxisomal OS=Bos taurus GN=PHYH PE=2 SV=2
evm.model.scaffold147433.30	0.723052	0.170856	2.08132	0.0399665	sp|O88281|MEGF6_RAT Multiple

					epidermal growth factor-like domains protein 6 OS=Rattus norvegicus GN=Megf6 PE=1 SV=1
evm.model.scaffold2353.12	83.3692	51.1796	0.703944	0.0121758	sp|Q80V70|MEGF6_MOUSE Multiple epidermal growth factor-like domains protein 6 OS=Mus musculus GN=Megf6 PE=2 SV=3
evm.model.scaffold171603.10.1	4.25241	1.66924	1.34909	0.00151774	sp|P70324|TBX3_MOUSE T-box transcription factor TBX3 OS=Mus musculus GN=Tbx3 PE=1 SV=3
evm.model.scaffold161771.37	66.9595	43.2945	0.629107	0.0299214	sp|O08623|SQSTM_RAT Sequestosome-1 OS=Rattus norvegicus GN=Sqstm1 PE=1 SV=1
evm.model.scaffold25809.10	30800.4	7263.68	2.08418	0.0211683	sp|Q9GZ71|TPM_HALDV Tropomyosin OS=Haliotis diversicolor PE=2 SV=1
evm.model.scaffold77121.17	26.8567	11.0341	1.28331	0.000820679	sp|O16264|PEBPH_CAEEL Phosphatidylethanolamine-binding protein homolog F40A3.3 OS=Caenorhabditis elegans GN=F40A3.3 PE=3 SV=1
evm.model.scaffold176027.38	12.4389	5.81027	1.09818	0.000820679	sp|E1BD59|TRI56_BOVIN E3 ubiquitin-protein ligase TRIM56 OS=Bos taurus GN=TRIM56 PE=3 SV=1
evm.model.scaffold156031.1.2	40.3652	26.0419	0.632275	0.023485	--

evm.model.scaffold8221.4	7.2701	3.4951	1.05664	0.000820679	sp|Q9IBG7|KCP_XENLA Kielin/chordin-like protein OS=Xenopus laevis GN=kcp PE=2 SV=1
evm.model.scaffold175211.2	25177.3	1764.28	3.83497	0.000820679	sp|Q24799|MYPH_ECHGR Myophilin OS=Echinococcus granulosus PE=2 SV=1
evm.model.scaffold120493.3	35.82	15.6325	1.19622	0.000820679	sp|O75391|SPAG7_HUMAN Sperm-associated antigen 7 OS=Homo sapiens GN=SPAG7 PE=1 SV=2
evm.model.scaffold109361.14_evm.model.scaffold109361.15	745.643	320.428	1.21849	0.0248731	sp|Q5DTN8|JKIP3_MOUSE Janus kinase and microtubule-interacting protein 3 OS=Mus musculus GN=Jakmip3 PE=2 SV=2
evm.model.scaffold54691.1	4.38246	2.09524	1.06463	0.00271512	sp|Q25414|5HTR_LYMST 5-hydroxytryptamine receptor OS=Lymnaea stagnalis PE=2 SV=1
evm.model.scaffold93679.3	12.5481	6.22049	1.01237	0.00271512	--
evm.model.scaffold143175.3	4.90254	2.97007	0.72303	0.0474104	sp|Q8ITC7|CAPAR_DROME Neuropeptides capa receptor OS=Drosophila melanogaster GN=CapaR PE=2 SV=3
evm.model.scaffold175015.12	18.019	11.8388	0.605994	0.0476583	sp|H2A0M3|AMO_PINMG Putative amine oxidase [copper-containing] OS=Pinctada margaritifera PE=1 SV=1

evm.model.scaffold138423.4_evm.model.scaffold138423.5	29.8171	17.2797	0.787063	0.00480132	sp|P49748|ACADV_HUMAN Very long-chain specific acyl-CoA dehydrogenase, mitochondrial OS=Homo sapiens GN=ACADVL PE=1 SV=1
evm.model.scaffold160813.15	0.455526	0.0809547	2.49235	0.00151774	sp|Q03132|ERYA2_SACER Erythronolide synthase, modules 3 and 4 OS=Saccharopolyspora erythraea GN=eryA PE=1 SV=3
evm.model.scaffold146043.9	341.307	90.2315	1.91936	0.000820679	sp|O01761|UNC89_CAEEL Muscle M-line assembly protein unc-89 OS=Caenorhabditis elegans GN=unc-89 PE=1 SV=3
evm.model.scaffold103277.15	109.231	57.1139	0.935473	0.000820679	sp|Q9JMG6|TFPT_RAT TCF3 fusion partner homolog OS=Rattus norvegicus GN=Tfpt PE=1 SV=2
evm.model.scaffold79089.12	2.97798	1.42593	1.06243	0.0113727	sp|O88831|KKCC2_RAT Calcium/calmodulin-dependent protein kinase kinase 2 OS=Rattus norvegicus GN=Camkk2 PE=1 SV=1
evm.model.scaffold54277.3	21.9675	13.9541	0.65468	0.032187	sp|Q5EA25|SRPX2_BOVIN Sushi repeat-containing protein SRPX2 OS=Bos taurus GN=SRPX2 PE=2 SV=1
evm.model.scaffold18691.5	1.31506	0.214063	2.61902	0.00326205	sp|P29590|PML_HUMAN Protein PML OS=Homo sapiens GN=PML

					PE=1 SV=3
evm.model.scaffold164239.3	113.677	66.0084	0.784221	0.00326205	sp|Q6DRI1|EI3EA_DANRE Eukaryotic translation initiation factor 3 subunit E-A OS=Danio rerio GN=eif3ea PE=2 SV=2
evm.model.scaffold73127.70	51.2567	27.259	0.911008	0.00151774	sp|Q64640|ADK_RAT Adenosine kinase OS=Rattus norvegicus GN=Adk PE=1 SV=3
evm.model.scaffold175495.10	33.3446	20.3335	0.713599	0.0121758	sp|A7MBL8|PKN2_DANRE Serine/threonine-protein kinase N2 OS=Danio rerio GN=pkn2 PE=2 SV=1
evm.model.scaffold171655.16.1	749.479	141.105	2.40912	0.000820679	sp|P24733|MYS_ARGIR Myosin heavy chain, striated muscle OS=Argopecten irradians PE=1 SV=1
evm.model.scaffold69023.9	52.7018	25.5556	1.04421	0.000820679	sp|P53590|SUCB2_PIG Succinyl-CoA ligase [GDP-forming] subunit beta, mitochondrial (Fragment) OS=Sus scrofa GN=SUCLG2 PE=1 SV=2
evm.model.scaffold66661.79_evm.model.scaffold66661.80	16.33	8.12794	1.00657	0.000820679	sp|Q9HCJ6|VAT1L_HUMAN Synaptic vesicle membrane protein VAT-1 homolog-like OS=Homo sapiens GN=VAT1L PE=1 SV=2
evm.model.scaffold19571.21	147.083	58.4622	1.33106	0.000820679	sp|P30044|PRDX5_HUMAN Peroxiredoxin-5, mitochondrial OS=Homo sapiens GN=PRDX5 PE=1

					SV=4
evm.model.scaffold112961.13_evm.model.scaffold112961.17	5.38402	3.06608	0.812289	0.0339147	sp|Q09660|CC44_CAEEL Probable cytochrome P450 CYP44 OS=Caenorhabditis elegans GN=cyp-44A1 PE=3 SV=2
evm.model.scaffold168051.1	52.855	27.489	0.943184	0.000820679	sp|Q92828|COR2A_HUMAN Coronin-2A OS=Homo sapiens GN=CORO2A PE=2 SV=2
evm.model.scaffold139253.47	74.1659	31.9871	1.21327	0.000820679	sp|Q9Z2W9|GRIA3_MOUSE Glutamate receptor 3 OS=Mus musculus GN=Gria3 PE=1 SV=2
evm.model.scaffold30535.2	3.01771	1.06937	1.49669	0.00621905	sp|Q11082|YT66_CAEEL Probable G-protein coupled receptor B0563.6 OS=Caenorhabditis elegans GN=B0563.6 PE=3 SV=2
evm.model.scaffold50517.15	6.74002	3.67654	0.874401	0.00670086	sp|Q5ZEQ8|FJX1_XIPMA Four-jointed box protein 1 OS=Xiphophorus maculatus GN=fjx1 PE=2 SV=1
evm.model.scaffold124911.13	7.15604	1.67108	2.09838	0.00151774	--
evm.model.scaffold124911.12	61.504	34.1601	0.84837	0.000820679	--
evm.model.scaffold124911.11	21.0647	9.74539	1.11204	0.000820679	--
evm.model.scaffold47331.8	65.3236	30.9036	1.07983	0.00213042	sp|Q7PPU9|BND7A_ANOGA Band 7 protein AGAP004871 OS=Anopheles gambiae GN=AGAP004871 PE=3 SV=3

evm.model.scaffold47331.9	42.61	16.9686	1.32833	0.000820679	sp|Q27433|MEC2_CAEEL Mechanosensory protein 2 OS=Caenorhabditis elegans GN=mec-2 PE=1 SV=1
evm.model.scaffold162731.54	19.1285	9.18636	1.05816	0.000820679	sp|Q9UBQ7|GRHPR_HUMAN Glyoxylate reductase/hydroxypyruvate reductase OS=Homo sapiens GN=GRHPR PE=1 SV=1
evm.model.scaffold150821.83	4.96542	2.5095	0.984514	0.0274321	sp|Q28262|PAFA_CANFA Platelet-activating factor acetylhydrolase OS=Canis familiaris GN=PLA2G7 PE=2 SV=1
evm.model.scaffold150821.82	2.13193	0.917064	1.21706	0.0284609	sp|P0C872|JMJD7_MOUSE JmjC domain-containing protein 7 OS=Mus musculus GN=Jmjd7 PE=2 SV=1
evm.model.scaffold72185.35	38.9899	22.1334	0.816877	0.0129481	--
evm.model.scaffold174195.1	1.17875	0.450383	1.38804	0.00972356	sp|A2AVA0|SVEP1_MOUSE Sushi, von Willebrand factor type A, EGF and pentraxin domain-containing protein 1 OS=Mus musculus GN=Svep1 PE=1 SV=1
evm.model.scaffold71119.54	91.4689	45.4747	1.00822	0.000820679	sp|Q9VI93|RN_DROME Zinc finger protein rotund OS=Drosophila melanogaster GN=rn PE=1 SV=2

evm.model.scaffold88219.1	0.502788	0.106219	2.24291	0.023485	sp|Q56R14|TRI33_XENLA E3 ubiquitin-protein ligase TRIM33 OS=Xenopus laevis GN=trim33 PE=1 SV=1
evm.model.scaffold130715.8.1	31.5567	19.8345	0.669933	0.0222103	sp|Q07496|EPHA4_CHICK Ephrin type-A receptor 4 OS=Gallus gallus GN=EPHA4 PE=1 SV=2
evm.model.scaffold11195.6	6.214	3.05876	1.02257	0.00621905	sp|O14901|KLF11_HUMAN Krueppel-like factor 11 OS=Homo sapiens GN=KLF11 PE=1 SV=2
evm.model.scaffold108761.12	54.3843	34.2862	0.665561	0.016685	sp|P20070|NB5R3_RAT NADH-cytochrome b5 reductase 3 OS=Rattus norvegicus GN=Cyb5r3 PE=1 SV=2
evm.model.scaffold81773.46	8.26103	4.38317	0.914349	0.0113727	sp|O15315|RA51B_HUMAN DNA repair protein RAD51 homolog 2 OS=Homo sapiens GN=RAD51B PE=1 SV=2
evm.model.scaffold65829.4	44.1639	22.8828	0.948602	0.00271512	--
evm.model.scaffold169005.14	15.8368	8.23761	0.942988	0.0316212	--
evm.model.scaffold164073.8	17.7862	10.938	0.701418	0.0327195	sp|Q5RFQ0|FCF1_PONAB rRNA-processing protein FCF1 homolog OS=Pongo abelii GN=FCF1 PE=2 SV=1
evm.model.scaffold143471.13	11.5125	6.27064	0.876519	0.020248	--

evm.model.scaffold146805.11	45.2837	16.6646	1.4422	0.000820679	sp|Q9H330|TM245_HUMAN Transmembrane protein 245 OS=Homo sapiens GN=TMEM245 PE=1 SV=2
evm.model.scaffold9075.12.1	67.5464	38.8433	0.798214	0.00380231	sp|Q86XE3|MICU3_HUMAN Calcium uptake protein 3, mitochondrial OS=Homo sapiens GN=MICU3 PE=2 SV=1
evm.model.scaffold67785.32.2	49.069	29.6788	0.725379	0.00670086	sp|Q4VCS5|AMOT_HUMAN Angiomotin OS=Homo sapiens GN=AMOT PE=1 SV=1
evm.model.scaffold171655.24	93.4714	27.3113	1.77503	0.000820679	sp|P24733|MYS_ARGIR Myosin heavy chain, striated muscle OS=Argopecten irradians PE=1 SV=1
evm.model.scaffold70889.2	5.51232	3.20169	0.783827	0.0188471	sp|O43451|MGA_HUMAN Maltase-glucoamylase, intestinal OS=Homo sapiens GN=MGAM PE=1 SV=5
evm.model.scaffold169517.3	38.2356	14.8884	1.36072	0.000820679	sp|P24733|MYS_ARGIR Myosin heavy chain, striated muscle OS=Argopecten irradians PE=1 SV=1
evm.model.scaffold169517.1	211.19	64.5002	1.71116	0.000820679	sp|P24733|MYS_ARGIR Myosin heavy chain, striated muscle OS=Argopecten irradians PE=1 SV=1
evm.model.scaffold125983.8	520.463	263.083	0.984278	0.000820679	sp|O13082|COX6A_CYPCA Cytochrome c oxidase subunit 6A,

					mitochondrial OS=Cyprinus carpio PE=3 SV=1
evm.model.scaffold173893.31	105.81	54.6848	0.952264	0.00429787	--
evm.model.scaffold168071.4	7.13034	3.75541	0.925	0.0191834	sp|P18293|ANPRA_MOUSE Atrial natriuretic peptide receptor 1 OS=Mus musculus GN=Npr1 PE=2 SV=2
evm.model.scaffold147331.18	90.9242	47.2317	0.944908	0.00213042	sp|Q90370|MAFB_COTJA Transcription factor MafB OS=Coturnix coturnix japonica GN=MAFB PE=1 SV=1
evm.model.scaffold144475.14	45.4151	19.4589	1.22274	0.000820679	sp|Q06455|MTG8_HUMAN Protein CBFA2T1 OS=Homo sapiens GN=RUNX1T1 PE=1 SV=2
evm.model.scaffold2221.2	10.1011	3.80095	1.41008	0.000820679	sp|O17185|SUP9_CAEEL Two pore potassium channel protein sup-9 OS=Caenorhabditis elegans GN=sup-9 PE=1 SV=2
evm.model.scaffold98447.53	5.73986	2.93292	0.968679	0.0211683	sp|Q66JD1|ATPF1_XENTR ATP synthase mitochondrial F1 complex assembly factor 1 OS=Xenopus tropicalis GN=atpaf1 PE=2 SV=1
evm.model.scaffold71897.36	5.40037	2.80383	0.945662	0.00712329	sp|Q9ULJ7|ANR50_HUMAN Ankyrin repeat domain-containing protein 50 OS=Homo sapiens GN=ANKRD50 PE=1 SV=4

evm.model.scaffold95641.27	14.9409	4.95413	1.59256	0.0125549	--
evm.model.scaffold106649.13	41.618	24.9614	0.737505	0.0117709	--
evm.model.scaffold176379.27	5.47967	1.95778	1.48487	0.0156116	sp|A3KG59|P20D2_MOUSE Peptidase M20 domain-containing protein 2 OS=Mus musculus GN=Pm20d2 PE=2 SV=1
evm.model.scaffold152753.12	10.6981	2.85693	1.90482	0.0271297	sp|Q6DFQ5|TMM97_XENTR Transmembrane protein 97 OS=Xenopus tropicalis GN=tmem97 PE=2 SV=1
evm.model.scaffold143341.10	94.9615	28.9266	1.71495	0.000820679	sp|P52034|PFKA_DROME ATP-dependent 6-phosphofructokinase OS=Drosophila melanogaster GN=Pfk PE=2 SV=2
evm.model.scaffold169189.1	62.5102	32.5192	0.9428	0.00842602	--
evm.model.scaffold169189.6	485.766	242.841	1.00025	0.00271512	sp|Q60865|CAPR1_MOUSE Caprin-1 OS=Mus musculus GN=Caprin1 PE=1 SV=2
evm.model.scaffold88177.32	950.598	183.713	2.37138	0.000820679	sp|Q8C8H8|KY_MOUSE Kyphoscoliosis peptidase OS=Mus musculus GN=Ky PE=1 SV=1
evm.model.scaffold171767.7_evm.model.scaffold171767.8	30.8365	19.7426	0.643329	0.0251677	sp|P00516|KGP1_BOVIN cGMP-dependent protein kinase 1 OS=Bos taurus GN=PRKG1 PE=1 SV=2
evm.model.scaffold151227.15	9.17536	4.71094	0.961748	0.00271512	sp|Q28X44|OCAD1_DROPS OCIA

					domain-containing protein 1 OS=Drosophila pseudoobscura pseudoobscura GN=GA12348 PE=3 SV=1
evm.model.scaffold170995.56.1	4871.49	1037.77	2.23088	0.00151774	sp|Q9NFT7|HXK2_DROME Hexokinase type 2 OS=Drosophila melanogaster GN=Hex-t2 PE=2 SV=4
evm.model.scaffold16423.32	41.9322	26.4363	0.66554	0.0211683	sp|Q16981|NCS1_APLCA Neuronal calcium sensor 1 OS=Aplysia californica PE=2 SV=2
evm.model.scaffold84179.15	18.0397	10.9217	0.723979	0.0305208	sp|Q49B93|SC5AC_MOUSE Sodium-coupled monocarboxylate transporter 2 OS=Mus musculus GN=Slc5a12 PE=1 SV=1
evm.model.scaffold92767.10	24.8469	11.2151	1.14762	0.000820679	sp|Q9ULJ7|ANR50_HUMAN Ankyrin repeat domain-containing protein 50 OS=Homo sapiens GN=ANKRD50 PE=1 SV=4
evm.model.scaffold106897.3	2.26367	0.180832	3.64594	0.000820679	sp|Q9D5S7|LRGUK_MOUSE Leucine-rich repeat and guanylate kinase domain-containing protein OS=Mus musculus GN=Lrguk PE=2 SV=1
evm.model.scaffold60761.3	13.6371	5.85034	1.22094	0.000820679	--
evm.model.scaffold144121.13	4.7133	1.17789	2.00053	0.000820679	sp|Q15392|DHC24_HUMAN

					Delta(24)-sterol reductase OS=Homo sapiens GN=DHCR24 PE=1 SV=2
evm.model.scaffold142101.5	183.09	80.9845	1.17684	0.000820679	sp|Q02645|HTS_DROME Protein hu-li tai shao OS=Drosophila melanogaster GN=hts PE=1 SV=2
evm.model.scaffold162017.14	9.65935	2.59707	1.89504	0.00578348	sp|Q0P5V2|SOBP_MOUSE Sine oculis-binding protein homolog OS=Mus musculus GN=Sobp PE=2 SV=1
evm.model.scaffold117139.7	194.403	112.159	0.793501	0.0148208	sp|P98160|PGBM_HUMAN Basement membrane-specific heparan sulfate proteoglycan core protein OS=Homo sapiens GN=HSPG2 PE=1 SV=4
evm.model.scaffold169873.36	13.1191	6.43468	1.02773	0.0105331	--
evm.model.scaffold5455.16	90.3623	57.0639	0.663143	0.0305208	sp|P24802|PLOD1_CHICK Procollagen-lysine,2-oxoglutarate 5-dioxygenase 1 OS=Gallus gallus GN=PLOD1 PE=1 SV=1
evm.model.scaffold148899.16	30.3167	18.1472	0.740367	0.00972356	sp|Q8IDX6|RBP2A_PLAF7 Reticulocyte-binding protein 2 homolog a OS=Plasmodium falciparum (isolate 3D7) GN=PF13_0198 PE=3 SV=1
evm.model.scaffold41199.17	101.739	39.5831	1.36192	0.000820679	sp|O01761|UNC89_CAEEL Muscle M-line assembly protein unc-89

					OS=Caenorhabditis elegans GN=unc-89 PE=1 SV=3
evm.model.scaffold159865.3	63.0487	36.7767	0.777676	0.0121758	sp|Q96PH1|NOX5_HUMAN NADPH oxidase 5 OS=Homo sapiens GN=NOX5 PE=1 SV=1
evm.model.scaffold110183.17	61.6729	24.7734	1.31584	0.000820679	sp|Q6TXF1|ARRD3_RAT Arrestin domain-containing protein 3 OS=Rattus norvegicus GN=Arrdc3 PE=2 SV=1
evm.model.scaffold11307.34	71.2055	29.3421	1.27902	0.000820679	sp|Q99NB1|ACS2L_MOUSE Acetyl-coenzyme A synthetase 2-like, mitochondrial OS=Mus musculus GN=Acss1 PE=1 SV=1
evm.model.scaffold124199.8	41.4809	21.594	0.941816	0.000820679	sp|P54821|PRRX1_HUMAN Paired mesoderm homeobox protein 1 OS=Homo sapiens GN=PRRX1 PE=1 SV=2
evm.model.scaffold167705.6	29458.4	9346.73	1.65614	0.0274321	sp|Q32L92|CNN3_BOVIN Calponin-3 OS=Bos taurus GN=CNN3 PE=2 SV=1
evm.model.scaffold167705.4	0.799359	0.160611	2.31527	0.0251677	sp|Q08890|IDS_MOUSE Iduronate 2-sulfatase OS=Mus musculus GN=Ids PE=2 SV=3
evm.model.scaffold40311.60	122.186	38.6789	1.65946	0.000820679	sp|Q868Z9|PPN_DROME Papilin OS=Drosophila melanogaster GN=Ppn PE=1 SV=2

evm.model.scaffold54533.11	51.2454	29.9138	0.77661	0.00755259	sp|Q8SWR3|SPR_DROME Sex peptide receptor OS=Drosophila melanogaster GN=SPR PE=1 SV=1
evm.model.scaffold176495.42	482.796	78.8305	2.61459	0.000820679	sp|Q24799|MYPH_ECHGR Myophilin OS=Echinococcus granulosus PE=2 SV=1
evm.model.scaffold142393.86	9.45683	3.51459	1.428	0.016685	sp|Q13489|BIRC3_HUMAN Baculoviral IAP repeat-containing protein 3 OS=Homo sapiens GN=BIRC3 PE=1 SV=2
evm.model.scaffold109263.34	33.1953	14.6628	1.17881	0.000820679	--
evm.model.scaffold169189.30	639.481	255.611	1.32295	0.000820679	sp|Q9PW72|PDLI4_CHICK PDZ and LIM domain protein 4 OS=Gallus gallus GN=PDLIM4 PE=2 SV=1
evm.model.scaffold151467.50_evm.model.scaffold151467.51	10.9698	6.95724	0.656957	0.0439851	sp|A1L252|RANB9_DANRE Ran-binding protein 9 OS=Danio rerio GN=ranbp9 PE=2 SV=1
evm.model.scaffold167419.7	2.85825	1.39455	1.03533	0.00712329	sp|Q9ULJ7|ANR50_HUMAN Ankyrin repeat domain-containing protein 50 OS=Homo sapiens GN=ANKRD50 PE=1 SV=4
evm.model.scaffold167419.2	20.7351	10.79	0.942376	0.00712329	sp|Q6YP21|KAT3_HUMAN Kynurenine--oxoglutarate transaminase 3 OS=Homo sapiens GN=CCBL2 PE=1 SV=1

evm.model.scaffold124683.19	11.371	4.13338	1.45996	0.000820679	sp|Q9NX94|WBP1L_HUMAN WW domain binding protein 1-like OS=Homo sapiens GN=WBP1L PE=1 SV=2
evm.model.scaffold133599.5	13.7993	4.43982	1.63602	0.000820679	sp|O01393|UNC9_CAEEL Innexin unc-9 OS=Caenorhabditis elegans GN=unc-9 PE=2 SV=1
evm.model.scaffold133599.4	26.9301	14.5799	0.885241	0.00578348	sp|Q03412|UNC7_CAEEL Innexin unc-7 OS=Caenorhabditis elegans GN=unc-7 PE=2 SV=1
evm.model.scaffold176307.34	305.601	176.434	0.79252	0.00326205	sp|Q9D3D9|ATPD_MOUSE ATP synthase subunit delta, mitochondrial OS=Mus musculus GN=Atp5d PE=1 SV=1
evm.model.scaffold161513.31	1.31759	0.603831	1.12568	0.0199213	sp|Q8CDK2|CBPC2_MOUSE Cytosolic carboxypeptidase 2 OS=Mus musculus GN=Agbl2 PE=1 SV=1
evm.model.scaffold168127.2	114.568	29.4405	1.96034	0.000820679	sp|Q9VT65|CANB_DROME Calpain-B OS=Drosophila melanogaster GN=CalpB PE=1 SV=2
evm.model.scaffold4085.9	45.9778	30.5531	0.589621	0.0439851	sp|A3KQ55|MEPCE_DANRE 7SK snRNA methylphosphate capping enzyme OS=Danio rerio GN=mepce PE=3 SV=2
evm.model.scaffold66879.8	144.191	83.5199	0.787789	0.00578348	--

evm.model.scaffold119983.22	0.430737	0.0476823	3.17528	0.0471091	sp|P0C0W8|GP139_RAT Probable G-protein coupled receptor 139 OS=Rattus norvegicus GN=Gpr139 PE=2 SV=1
evm.model.scaffold146097.5	5.51581	2.75041	1.00393	0.0327195	sp|Q17QJ7|P5CR2_BOVIN Pyrroline-5-carboxylate reductase 2 OS=Bos taurus GN=PYCR2 PE=2 SV=1
evm.model.scaffold176617.8	287.604	161.611	0.831555	0.00213042	--
evm.model.scaffold128495.4	21.1654	11.1217	0.928325	0.00380231	sp|Q21355|GST4_CAEEL Glutathione S-transferase 4 OS=Caenorhabditis elegans GN=gst-4 PE=2 SV=1
evm.model.scaffold161771.46	4.18732	2.49951	0.744381	0.035577	sp|P39087|GRIK2_MOUSE Glutamate receptor ionotropic, kainate 2 OS=Mus musculus GN=Grik2 PE=1 SV=4
evm.model.scaffold161771.42	58.6992	38.2978	0.616078	0.040273	sp|A7E300|RHG07_BOVIN Rho GTPase-activating protein 7 OS=Bos taurus GN=DLC1 PE=2 SV=1
evm.model.scaffold12723.6	20.6218	12.1028	0.768832	0.016685	sp|Q3MHV6|SLAI2_BOVIN SLAIN motif-containing protein 2 OS=Bos taurus GN=SLAIN2 PE=2 SV=1
evm.model.scaffold139039.7	632.027	140.493	2.16949	0.000820679	sp|Q9VEN1|FLNA_DROME Filamin-A OS=Drosophila melanogaster GN=cher PE=1 SV=2
evm.model.scaffold7483.21	27.6003	17.8364	0.629859	0.0296885	sp|Q02962|PAX2_HUMAN Paired box protein Pax-2 OS=Homo sapiens

					GN=PAX2 PE=1 SV=4
evm.model.scaffold176307.6	225.635	132.78	0.76495	0.00755259	sp|P82198|BGH3_MOUSE Transforming growth factor-beta-induced protein ig-h3 OS=Mus musculus GN=Tgfbi PE=2 SV=1
evm.model.scaffold167725.18	148.375	28.5922	2.37555	0.000820679	sp|P35573|GDE_HUMAN Glycogen debranching enzyme OS=Homo sapiens GN=AGL PE=1 SV=3
evm.model.scaffold97101.9	92.9718	55.0167	0.756923	0.00480132	sp|P30883|TBB4_XENLA Tubulin beta-4 chain OS=Xenopus laevis GN=tubb4 PE=2 SV=1
evm.model.scaffold16203.6	11.0364	4.18953	1.39741	0.000820679	sp|A7Y2W8|SC6A9_XENLA Sodium- and chloride-dependent glycine transporter 1 OS=Xenopus laevis GN=slc6a9 PE=2 SV=1
evm.model.scaffold97101.4	16.0994	9.48397	0.763447	0.0211683	sp|P11833|TBB_PARLI Tubulin beta chain OS=Paracentrotus lividus PE=2 SV=1
evm.model.scaffold100453.4_evm.model.scaffold100453.5	5.37661	2.10538	1.35261	0.000820679	--
evm.model.scaffold169321.35	2.36752	0.978459	1.27479	0.000820679	--
evm.model.scaffold169321.37	9.63579	5.09967	0.918	0.0163478	--
evm.model.scaffold77121.37_evm.model.scaffold77121.38	59.4483	38.3023	0.634206	0.0327195	sp|Q9P0J7|KCMF1_HUMAN E3 ubiquitin-protein ligase KCMF1

					OS=Homo sapiens GN=KCMF1 PE=1 SV=2
evm.model.scaffold44571.4	13.8335	8.94392	0.629184	0.0329966	sp|Q28GD4|ELAV2_XENTR ELAV-like protein 2 OS=Xenopus tropicalis GN=elavl2 PE=2 SV=2
evm.model.scaffold153989.3	52.2355	34.7929	0.586238	0.0471091	sp|P13623|NFIX_MESAU Nuclear factor 1 X-type OS=Mesocricetus auratus GN=NFIX PE=2 SV=1
evm.model.scaffold92151.7	0.950497	0.333233	1.51215	0.0404844	sp|Q9CZR2|NALD2_MOUSE N-acetylated-alpha-linked acidic dipeptidase 2 OS=Mus musculus GN=Naalad2 PE=1 SV=2
evm.model.scaffold93679.40	3.55278	0.931595	1.93117	0.000820679	sp|Q95334|AMPE_PIG Glutamyl aminopeptidase OS=Sus scrofa GN=ENPEP PE=1 SV=1
evm.model.scaffold38707.7	1.12622	0.124507	3.17719	0.0282044	sp|Q6NZQ0|CP093_MOUSE Coiled-coil domain-containing protein C16orf93 homolog OS=Mus musculus GN=Gm166 PE=2 SV=2
evm.model.scaffold116119.99	28.3488	10.4243	1.44333	0.000820679	sp|Q3LXA3|DHAK_HUMAN Bifunctional ATP-dependent dihydroxyacetone kinase/FAD-AMP lyase (cyclizing) OS=Homo sapiens GN=DAK PE=1 SV=2
evm.model.scaffold128461.12	21.3277	11.585	0.880472	0.00213042	sp|P55199|ELL_HUMAN RNA

					polymerase II elongation factor ELL OS=Homo sapiens GN=ELL PE=1 SV=1
evm.model.scaffold128461.10	3.75585	1.72841	1.11969	0.00326205	sp|Q8IS44|DRD2L_DROME Dopamine D2-like receptor OS=Drosophila melanogaster GN=D2R PE=2 SV=1
evm.model.scaffold93729.1.1	22.8104	5.16888	2.14177	0.000820679	sp|Q5UQ50|COLL6_MIMIV Collagen-like protein 6 OS=Acanthamoeba polyphaga mimivirus GN=MIMI_L668 PE=4 SV=1
evm.model.scaffold119539.13	7.49373	4.54253	0.72219	0.039382	sp|Q5ZLC6|ANR10_CHICK Ankyrin repeat domain-containing protein 10 OS=Gallus gallus GN=ANKRD10 PE=2 SV=1
evm.model.scaffold166247.12	8.48032	4.87113	0.799861	0.0225564	sp|Q6UWP2|DHR11_HUMAN Dehydrogenase/reductase SDR family member 11 OS=Homo sapiens GN=DHRS11 PE=1 SV=1
evm.model.scaffold173703.2_evm.model.scaffold173703.3	9.94907	5.59669	0.829988	0.0169923	sp|O76082|S22A5_HUMAN Solute carrier family 22 member 5 OS=Homo sapiens GN=SLC22A5 PE=1 SV=1
evm.model.scaffold133759.4	11186.7	2224.49	2.33024	0.00755259	sp|P08051|MLR_SPISA Myosin regulatory light chain, smooth muscle OS=Spisula sachalinensis PE=1 SV=1
evm.model.scaffold133759.3	143.747	55.1191	1.3829	0.000820679	sp|P08051|MLR_SPISA Myosin

					regulatory light chain, smooth muscle OS=Spisula sachalinensis PE=1 SV=1
evm.model.scaffold173383.41	6.83347	3.71758	0.878253	0.0444534	sp|Q9NP81|SYSM_HUMAN Serine--tRNA ligase, mitochondrial OS=Homo sapiens GN=SARS2 PE=1 SV=1
evm.model.scaffold128139.15	36.4259	19.3635	0.911624	0.000820679	sp|Q8BL65|ABLM2_MOUSE Actin-binding LIM protein 2 OS=Mus musculus GN=Ablim2 PE=1 SV=1
evm.model.scaffold93615.2	9.11102	4.37837	1.05722	0.0287519	--
evm.model.scaffold173603.19	199.802	75.9015	1.39637	0.000820679	sp|P11217|PYGM_HUMAN Glycogen phosphorylase, muscle form OS=Homo sapiens GN=PYGM PE=1 SV=6
evm.model.scaffold139253.18	32.2928	15.1205	1.09471	0.000820679	sp|Q8BUZ1|ABRA_MOUSE Actin-binding Rho-activating protein OS=Mus musculus GN=Abra PE=1 SV=1
evm.model.scaffold70251.15	16.2702	10.3629	0.650796	0.0351039	sp|Q75J93|CPAS1_DICDI Circularly permutated Ras protein 1 OS=Dictyostelium discoideum GN=cpras1 PE=3 SV=1
evm.model.scaffold176119.100.1	33.1033	20.9298	0.661416	0.0228478	sp|Q3B7D5|RASF2_RAT Ras association domain-containing protein 2 OS=Rattus norvegicus GN=Rassf2

					PE=2 SV=1
evm.model.scaffold133265.1	2.71228	0.963776	1.49274	0.00271512	sp|Q29043|FUT1_PIG Galactoside 2-alpha-L-fucosyltransferase 1 OS=Sus scrofa GN=FUT1 PE=2 SV=2
evm.model.scaffold169587.4	57.8186	31.9629	0.855136	0.00151774	sp|P41827|HSP74_ANOAL Heat shock protein 70 B2 OS=Anopheles albimanus GN=HSP70B2 PE=3 SV=1
evm.model.scaffold95909.26	6.49862	2.03765	1.67323	0.000820679	--
evm.model.scaffold77533.3	4.01461	1.803	1.15486	0.0299214	sp|Q5TYS5|SCRN2_DANRE Secernin-2 OS=Danio rerio GN=scrn2 PE=2 SV=1
evm.model.scaffold163753.13	5.26974	2.06457	1.35189	0.00429787	sp|Q502K1|EXOG_DANRE Nuclease EXOG, mitochondrial OS=Danio rerio GN=exog PE=2 SV=2
evm.model.scaffold163753.16	74.7976	10.7938	2.79279	0.000820679	--
evm.model.scaffold163753.17	32.6827	20.5874	0.666765	0.0169923	sp|P26009|ITA8_CHICK Integrin alpha-8 OS=Gallus gallus GN=ITGA8 PE=1 SV=1
evm.model.scaffold163753.18	292.537	149.927	0.964365	0.00151774	sp|Q25117|ATPB_HEMPU ATP synthase subunit beta, mitochondrial OS=Hemicentrotus pulcherrimus PE=2 SV=1
evm.model.scaffold4289.36.1	20.1747	12.5134	0.689071	0.0188471	sp|Q6YHK3|CD109_HUMAN CD109 antigen OS=Homo sapiens GN=CD109 PE=1 SV=2

evm.model.scaffold168521.1	3.48208	1.13281	1.62004	0.0225564	--
evm.model.scaffold176577.14	10.7213	5.59591	0.938033	0.00151774	sp|Q95N78|PPARA_CANFA Peroxisome proliferator-activated receptor alpha OS=Canis familiaris GN=PPARA PE=2 SV=1
evm.model.scaffold80479.8	12.6628	7.0706	0.84069	0.00621905	sp|Q9UPR0|PLCL2_HUMAN Inactive phospholipase C-like protein 2 OS=Homo sapiens GN=PLCL2 PE=1 SV=2
evm.model.scaffold80479.7	7.40952	4.22514	0.810381	0.0413639	--
evm.model.scaffold72861.6	6.6874	3.20401	1.06157	0.0308116	sp|P79760|CP1A4_CHICK Cytochrome P450 1A4 OS=Gallus gallus GN=CYP1A4 PE=2 SV=1
evm.model.scaffold139771.9	36.0629	16.2582	1.14935	0.000820679	sp|Q803W1|SCRN3_DANRE Secernin-3 OS=Danio rerio GN=scrn3 PE=2 SV=1
evm.model.scaffold26189.74	10.7486	6.74463	0.672331	0.0465917	sp|O15973|OPSD1_MIZYE Rhodopsin, GQ-coupled OS=Mizuhopecten yessoensis GN=SCOP1 PE=1 SV=1
evm.model.scaffold4289.80	2.34791	0.57407	2.03208	0.00670086	sp|Q9NXG6|P4HTM_HUMAN Transmembrane prolyl 4-hydroxylase OS=Homo sapiens GN=P4HTM PE=1 SV=2
evm.model.scaffold4289.82	16.4335	8.09147	1.02217	0.00380231	sp|Q5RDW1|MTG2_PONAB

					Mitochondrial ribosome-associated GTPase 2 OS=Pongo abelii GN=MTG2 PE=2 SV=1
evm.model.scaffold134743.55	5.20891	1.83442	1.50566	0.000820679	sp|O42222|GDF8_DANRE Growth/differentiation factor 8 OS=Danio rerio GN=mstnb PE=2 SV=2
evm.model.scaffold142393.2	12.1934	2.79453	2.12542	0.0109379	--
evm.model.scaffold158907.14	25.699	14.8921	0.787166	0.0180929	sp|Q8LA32|LUL4_ARATH Probable E3 ubiquitin-protein ligase LUL4 OS=Arabidopsis thaliana GN=LUL4 PE=2 SV=1
evm.model.scaffold174343.31	9.31308	5.79064	0.685537	0.0408047	sp|Q62396|ZFP92_MOUSE Zinc finger protein 92 OS=Mus musculus GN=Zfp92 PE=2 SV=2
evm.model.scaffold115633.38	41.2877	26.3592	0.647408	0.0316212	sp|A1L224|CR3L2_DANRE Cyclic AMP-responsive element-binding protein 3-like protein 2 OS=Danio rerio GN=creb3l2 PE=2 SV=1
evm.model.scaffold115633.31	32.6919	20.7354	0.656833	0.032187	sp|Q13370|PDE3B_HUMAN cGMP-inhibited 3',5'-cyclic phosphodiesterase B OS=Homo sapiens GN=PDE3B PE=1 SV=2
evm.model.scaffold16445.45	7.10659	3.31092	1.10192	0.0271297	sp|Q9JIQ3|DBLOH_MOUSE Diablo homolog, mitochondrial OS=Mus musculus GN=Diablo PE=1 SV=2

evm.model.scaffold82339.9	51.4675	21.0407	1.29048	0.000820679	sp|P00949|PGM1_RABIT Phosphoglucomutase-1 OS=Oryctolagus cuniculus GN=PGM1 PE=1 SV=2
evm.model.scaffold81801.56.1	22.3299	12.1733	0.875255	0.00213042	sp|Q8BH79|ANO10_MOUSE Anoctamin-10 OS=Mus musculus GN=Ano10 PE=2 SV=1
evm.model.scaffold69685.7	34.3819	16.8484	1.02904	0.000820679	sp|Q3UTY6|THSD4_MOUSE Thrombospondin type-1 domain-containing protein 4 OS=Mus musculus GN=Thsd4 PE=1 SV=2
evm.model.scaffold132893.16	29.1206	13.8035	1.07701	0.000820679	sp|Q3MHJ7|EEPD1_BOVIN Endonuclease/exonuclease/phosphatase family domain-containing protein 1 OS=Bos taurus GN=EEPD1 PE=2 SV=1
evm.model.scaffold24095.2_evm.model.scaffold24095.3	26.5282	14.8278	0.839219	0.00213042	sp|Q8UVC3|INVS_CHICK Inversin OS=Gallus gallus GN=INVS PE=2 SV=2
evm.model.scaffold148899.5	92.1164	52.0923	0.822389	0.00271512	--
evm.model.scaffold43895.14	24.376	15.2785	0.673962	0.0429196	sp|P30568|GSTA_PLEPL Glutathione S-transferase A OS=Pleuronectes platessa PE=2 SV=1
evm.model.scaffold104051.15.1	177.722	81.4305	1.12598	0.000820679	sp|Q8VC31|CCDC9_MOUSE Coiled-coil domain-containing protein 9 OS=Mus musculus GN=Ccdc9 PE=2 SV=1

evm.model.scaffold97101.6_evm.model.scaffold97101.7	9.89394	3.99719	1.30756	0.000820679	sp|P30883|TBB4_XENLA Tubulin beta-4 chain OS=Xenopus laevis GN=tubb4 PE=2 SV=1
evm.model.scaffold45153.3	70.4834	26.1128	1.43253	0.000820679	sp|Q13639|5HT4R_HUMAN 5-hydroxytryptamine receptor 4 OS=Homo sapiens GN=HTR4 PE=1 SV=2
evm.model.scaffold123417.37	9.85085	5.75898	0.774434	0.0141107	sp|Q924H0|NPFF2_MOUSE Neuropeptide FF receptor 2 OS=Mus musculus GN=Npffr2 PE=2 SV=2
evm.model.scaffold417.4	65.2088	35.4168	0.880633	0.00213042	sp|Q6AZV0|DHSDB_XENLA Succinate dehydrogenase [ubiquinone] cytochrome b small subunit B, mitochondrial OS=Xenopus laevis GN=sdhd-b PE=2 SV=1
evm.model.scaffold54263.31	59.3827	30.2266	0.974226	0.00621905	sp|P26687|TWST1_MOUSE Twist-related protein 1 OS=Mus musculus GN=Twist1 PE=1 SV=1
evm.model.scaffold49771.29	20.742	11.8673	0.805559	0.00480132	sp|Q6ZN30|BNC2_HUMAN Zinc finger protein basonuclin-2 OS=Homo sapiens GN=BNC2 PE=1 SV=1
evm.model.scaffold43975.5	238.156	62.5883	1.92794	0.000820679	sp|O08722|UNC5B_RAT Netrin receptor UNC5B OS=Rattus norvegicus GN=Unc5b PE=1 SV=1
evm.model.scaffold127055.3	20.6974	13.3407	0.633615	0.0302156	sp|P20241|NRG_DROME Neuroglian

					OS=Drosophila melanogaster GN=Nrg PE=1 SV=2
evm.model.scaffold146247.30	10.4901	4.44625	1.23836	0.000820679	sp|Q6ZMM2|ATL5_HUMAN ADAMTS-like protein 5 OS=Homo sapiens GN=ADAMTSL5 PE=1 SV=3
evm.model.scaffold162503.12	1.28821	0.288152	2.16046	0.0308116	sp|Q9W740|NOGG2_DANRE Noggin-2 OS=Danio rerio GN=nog2 PE=2 SV=1
evm.model.scaffold175457.32.1	99.104	46.7458	1.08411	0.000820679	sp|Q8WP23|BOLL_MACFA Protein boule-like OS=Macaca fascicularis GN=BOLL PE=2 SV=2
evm.model.scaffold127921.38_evm.model.scaffold127921.39_evm.model.scaffold127921.40	54.8208	22.2136	1.30328	0.000820679	sp|O95714|HERC2_HUMAN E3 ubiquitin-protein ligase HERC2 OS=Homo sapiens GN=HERC2 PE=1 SV=2
evm.model.scaffold175759.9	39.9417	24.8704	0.683469	0.0180929	sp|O46629|ECHB_BOVIN Trifunctional enzyme subunit beta, mitochondrial OS=Bos taurus GN=HADHB PE=2 SV=1
evm.model.scaffold93071.4	24.8593	13.8373	0.84522	0.00578348	sp|P0AC33|FUMA_ECOLI Fumarate hydratase class I, aerobic OS=Escherichia coli (strain K12) GN=fumA PE=1 SV=2
evm.model.scaffold146109.6	33.2195	20.9983	0.661758	0.0222103	sp|Q3SZB4|ACADM_BOVIN Medium-chain specific acyl-CoA

					dehydrogenase, mitochondrial OS=Bos taurus GN=ACADM PE=2 SV=1
evm.model.scaffold132315.14	57.9116	20.3803	1.50668	0.000820679	sp|Q6PD31|TRAK1_MOUSE Trafficking kinesin-binding protein 1 OS=Mus musculus GN=Trak1 PE=1 SV=1
evm.model.scaffold98601.12	1.7096	0.589274	1.53665	0.00326205	sp|Q93126|GPR9_AMPAM Probable G-protein coupled receptor No9 OS=Amphibalanus amphitrite PE=3 SV=1
evm.model.scaffold82395.12	31.181	10.0643	1.63141	0.000820679	sp|P37167|ACTP_ACACA Actophorin OS=Acanthamoeba castellanii PE=1 SV=2
evm.model.scaffold11019.9	20.8707	9.8394	1.08484	0.0251677	sp|Q8WXF0|SRS12_HUMAN Serine/arginine-rich splicing factor 12 OS=Homo sapiens GN=SRSF12 PE=2 SV=1
evm.model.scaffold761.2.1	3.73054	0.950627	1.97243	0.00480132	--
evm.model.scaffold155537.5	6.43047	3.12623	1.0405	0.032187	--
evm.model.scaffold155537.4	7.05244	3.47937	1.0193	0.00429787	sp|Q92536|YLAT2_HUMAN Y+L amino acid transporter 2 OS=Homo sapiens GN=SLC7A6 PE=1 SV=3
evm.model.scaffold168725.19	34.1021	7.04203	2.2758	0.000820679	sp|Q5TJF5|DHB8_CANFA Estradiol 17-beta-dehydrogenase 8 OS=Canis familiaris GN=HSD17B8 PE=3 SV=1

evm.model.scaffold29495.6	666.124	174.816	1.92995	0.000820679	sp|Q16082|HSPB2_HUMAN Heat shock protein beta-2 OS=Homo sapiens GN=HSPB2 PE=1 SV=2
evm.model.scaffold116119.67	65.0098	40.766	0.673292	0.0148208	sp|Q07407|FGFR1_DROME Fibroblast growth factor receptor homolog 1 OS=Drosophila melanogaster GN=htl PE=1 SV=3
evm.model.scaffold152335.11	5.79959	3.33749	0.797187	0.0437576	sp|Q9EP89|LACTB_MOUSE Serine beta-lactamase-like protein LACTB, mitochondrial OS=Mus musculus GN=Lactb PE=1 SV=1
evm.model.scaffold173785.18	54.4217	31.8604	0.772419	0.00326205	sp|Q9DBL1|ACDSB_MOUSE Short/branched chain specific acyl-CoA dehydrogenase, mitochondrial OS=Mus musculus GN=Acadsb PE=1 SV=1
evm.model.scaffold173785.19	42.6399	10.9859	1.95655	0.0163478	sp|P70584|ACDSB_RAT Short/branched chain specific acyl-CoA dehydrogenase, mitochondrial OS=Rattus norvegicus GN=Acadsb PE=1 SV=1
evm.model.scaffold176495.79	24.6818	14.7819	0.739616	0.0382321	sp|Q2VPU4|MLXIP_MOUSE MLX-interacting protein OS=Mus musculus GN=Mlxip PE=1 SV=1
evm.model.scaffold82079.4	9.13173	4.73324	0.948061	0.0117709	sp|Q2KIQ4|COQ2_BOVIN

					4-hydroxybenzoate polyprenyltransferase, mitochondrial OS=Bos taurus GN=COQ2 PE=2 SV=1
evm.model.scaffold30711.26	6.81824	3.33468	1.03185	0.0101381	sp|O88281|MEGF6_RAT Multiple epidermal growth factor-like domains protein 6 OS=Rattus norvegicus GN=Megf6 PE=1 SV=1
evm.model.scaffold104945.24	723.497	206.535	1.8086	0.000820679	sp|P18091|ACTN_DROME Alpha-actinin, sarcomeric OS=Drosophila melanogaster GN=Actn PE=1 SV=2
evm.model.scaffold157929.25	24.8751	14.8902	0.74034	0.027885	--
evm.model.scaffold65937.5_evm.model.scaffold65937.6	7.53055	1.82799	2.04249	0.000820679	sp|C9JQI7|TM232_HUMAN Transmembrane protein 232 OS=Homo sapiens GN=TMEM232 PE=2 SV=2
evm.model.scaffold107225.82	818.735	369.126	1.14928	0.000820679	sp|Q04831|GLNA_PANAR Glutamine synthetase OS=Panulirus argus PE=2 SV=1
evm.model.scaffold14507.17	51.2284	21.1283	1.27776	0.000820679	sp|C1C4R8|BDH2_LITCT 3-hydroxybutyrate dehydrogenase type 2 OS=Lithobates catesbeiana GN=bdh2 PE=2 SV=1
evm.model.scaffold84953.16_evm.model.scaffold84953.15	7.54442	4.26859	0.821651	0.0105331	sp|P48764|SL9A3_HUMAN Sodium/hydrogen exchanger 3 OS=Homo sapiens GN=SLC9A3 PE=1

					SV=2
evm.model.scaffold171767.4	59.2027	32.6766	0.857404	0.00213042	sp|P20073|ANXA7_HUMAN Annexin A7 OS=Homo sapiens GN=ANXA7 PE=1 SV=3
evm.model.scaffold124281.7	41.7905	6.18427	2.7565	0.000820679	sp|Q9Y617|SERC_HUMAN Phosphoserine aminotransferase OS=Homo sapiens GN=PSAT1 PE=1 SV=2
evm.model.scaffold124281.5	8.50299	3.95335	1.1049	0.000820679	sp|P50747|BPL1_HUMAN Biotin--protein ligase OS=Homo sapiens GN=HLCS PE=1 SV=1
evm.model.scaffold167793.42	275.492	118.399	1.21836	0.000820679	sp|O00151|PDLI1_HUMAN PDZ and LIM domain protein 1 OS=Homo sapiens GN=PDLIM1 PE=1 SV=4
evm.model.scaffold161497.7	474.836	144.728	1.71408	0.000820679	sp|Q6P5H6|FRMD5_MOUSE FERM domain-containing protein 5 OS=Mus musculus GN=Frmd5 PE=2 SV=1
evm.model.scaffold56755.107	68.4368	12.4991	2.45295	0.000820679	--
evm.model.scaffold151467.147	71.2954	18.4235	1.95226	0.000820679	sp|Q9D687|S6A19_MOUSE Sodium-dependent neutral amino acid transporter B(0)AT1 OS=Mus musculus GN=Slc6a19 PE=1 SV=1
evm.model.scaffold25605.39	32.7504	14.701	1.1556	0.000820679	sp|A2VDZ3|MEF2A_BOVIN Myocyte-specific enhancer factor 2A OS=Bos taurus GN=MEF2A PE=2 SV=1

evm.model.scaffold138423.8	4.6777	1.27005	1.88092	0.000820679	sp|Q6ZSM3|MOT12_HUMAN Monocarboxylate transporter 12 OS=Homo sapiens GN=SLC16A12 PE=1 SV=2
evm.model.scaffold158547.47.1	133.757	85.8105	0.640384	0.0353697	sp|O96102|CALM_PHYPO Calmodulin OS=Physarum polycephalum PE=2 SV=3
evm.model.scaffold105045.4	184.903	64.8935	1.51062	0.000820679	sp|Q6DFV3|RHG21_MOUSE Rho GTPase-activating protein 21 OS=Mus musculus GN=Arhgap21 PE=1 SV=1
evm.model.scaffold105045.6	134.39	53.6452	1.3249	0.000820679	sp|Q9P227|RHG23_HUMAN Rho GTPase-activating protein 23 OS=Homo sapiens GN=ARHGAP23 PE=1 SV=2
evm.model.scaffold38207.22	3.25271	0.187603	4.11589	0.00578348	sp|P10280|VKT52_ANESU Kunitz-type proteinase inhibitor 5 II OS=Anemonia sulcata PE=1 SV=2
evm.model.scaffold64247.39	208.772	126.735	0.720113	0.00842602	sp|O75439|MPPB_HUMAN Mitochondrial-processing peptidase subunit beta OS=Homo sapiens GN=PMPCB PE=1 SV=2
evm.model.scaffold82339.26	8.65511	4.19953	1.04332	0.00670086	sp|Q8KSC8|RDPA_SPHHM (R)-phenoxypropionate/alpha-ketoglutarate-dioxygenase OS=Sphingobium herbicidovorans (strain ATCC 700291 /

					DSM 11019 / NBRC 16415 / MH) GN=rdpA PE=1 SV=2
evm.model.scaffold172145.25	12.6813	4.55243	1.478	0.00842602	sp|O69060|HTXA_PSEST Probable alpha-ketoglutarate-dependent hypophosphite dioxygenase OS=Pseudomonas stutzeri GN=htxA PE=3 SV=1
evm.model.scaffold172145.29	58.8283	27.3767	1.10356	0.000820679	sp|O15374|MOT5_HUMAN Monocarboxylate transporter 5 OS=Homo sapiens GN=SLC16A4 PE=2 SV=1
evm.model.scaffold139039.22	422.273	137.859	1.61499	0.000820679	sp|O00151|PDLI1_HUMAN PDZ and LIM domain protein 1 OS=Homo sapiens GN=PDLIM1 PE=1 SV=4
evm.model.scaffold158265.20	20.1732	10.8305	0.897342	0.00380231	sp|Q5ZJ87|BMT2_CHICK Probable methyltransferase BTM2 homolog OS=Gallus gallus GN=RCJMB04_20b4 PE=2 SV=1
evm.model.scaffold158265.24	1033.08	186.288	2.47135	0.000820679	sp|Q8BTM8|FLNA_MOUSE Filamin-A OS=Mus musculus GN=Flna PE=1 SV=5
evm.model.scaffold158265.26	6.95274	4.24715	0.711087	0.0257834	sp|Q5UQ35|YR811_MIMIV Putative ariadne-like RING finger protein R811 OS=Acanthamoeba polyphaga mimivirus GN=MIMI_R811 PE=3 SV=1

evm.model.scaffold120675.1	57.2037	34.5264	0.728409	0.0169923	sp|Q24270|CAC1D_DROME Voltage-dependent calcium channel type D subunit alpha-1 OS=Drosophila melanogaster GN=Ca-alpha1D PE=1 SV=2
evm.model.scaffold126527.21	12.2155	7.24042	0.754573	0.0316212	sp|Q54KA7|SECG_DICDI Ankyrin repeat, PH and SEC7 domain containing protein secG OS=Dictyostelium discoideum GN=secG PE=2 SV=1
evm.model.scaffold80359.16	5936.41	664.696	3.15882	0.000820679	sp|P51544|KARG_HALMK Arginine kinase OS=Haliotis madaka PE=2 SV=1
evm.model.scaffold107183.6	49.6673	25.616	0.95525	0.00151774	sp|P00348|HCDH_PIG Hydroxyacyl-coenzyme A dehydrogenase, mitochondrial OS=Sus scrofa GN=HADH PE=1 SV=2
evm.model.scaffold107183.1	82.5914	47.7293	0.791116	0.00151774	sp|Q14517|FAT1_HUMAN Protocadherin Fat 1 OS=Homo sapiens GN=FAT1 PE=1 SV=2
evm.model.scaffold139039.24.1	291.007	78.6696	1.88717	0.000820679	sp|Q8CI51|PDLI5_MOUSE PDZ and LIM domain protein 5 OS=Mus musculus GN=Pdlim5 PE=1 SV=4
evm.model.scaffold173495.19	28.7485	17.3943	0.724874	0.0113727	sp|Q5R1P3|NMDE2_CANFA Glutamate receptor ionotropic, NMDA 2B OS=Canis familiaris GN=GRIN2B

					PE=2 SV=1
evm.model.scaffold161075.24	26.958	16.5408	0.704687	0.0268133	sp|Q9CZR2|NALD2_MOUSE N-acetylated-alpha-linked acidic dipeptidase 2 OS=Mus musculus GN=Naalad2 PE=1 SV=2
evm.model.scaffold161075.25	4.58177	1.30194	1.81524	0.000820679	sp|Q8WT51|YL4M_CAEEL Uncharacterized protein F35H12.5 OS=Caenorhabditis elegans GN=F35H12.5 PE=4 SV=1
evm.model.scaffold52185.4	24.8337	12.1302	1.0337	0.000820679	sp|O62640|PIAP_PIG Putative inhibitor of apoptosis OS=Sus scrofa GN=PIAP PE=2 SV=1
evm.model.scaffold68265.5	21.374	11.1909	0.933537	0.000820679	sp|Q9BZF1|OSBL8_HUMAN Oxysterol-binding protein-related protein 8 OS=Homo sapiens GN=OSBPL8 PE=1 SV=3
evm.model.scaffold26071.12	10.4335	5.0215	1.05503	0.000820679	--
evm.model.scaffold4431.2	15.9746	5.2309	1.61065	0.00151774	--
evm.model.scaffold35585.5	1.17304	0.577805	1.02159	0.0418385	sp|Q07008|NOTC1_RAT Neurogenic locus notch homolog protein 1 OS=Rattus norvegicus GN=Notch1 PE=1 SV=3
evm.model.scaffold176235.2	0.957193	0	inf	0.0271297	--
evm.model.scaffold121775.31	1874.06	365.059	2.35996	0.000820679	sp|Q9BLG0|TNNC_TODPA Troponin C OS=Todarodes pacificus PE=1 SV=3

evm.model.scaffold69419.38	8.4808	4.70469	0.850102	0.00621905	sp|Q9W4Y2|PDFR_DROME PDF receptor OS=Drosophila melanogaster GN=Pdfr PE=1 SV=2
evm.model.scaffold115693.28	155.553	90.1034	0.787756	0.00326205	sp|O35143|ATIF1_MOUSE ATPase inhibitor, mitochondrial OS=Mus musculus GN=Atpif1 PE=1 SV=2
evm.model.scaffold104507.8	36.3861	22.5729	0.688794	0.0254597	sp|O54975|XPP1_RAT Xaa-Pro aminopeptidase 1 OS=Rattus norvegicus GN=Xpnpep1 PE=1 SV=1
evm.model.scaffold139253.26	33.0733	17.5438	0.914702	0.00151774	sp|Q6P7Q4|LGUL_RAT Lactoylglutathione lyase OS=Rattus norvegicus GN=Glo1 PE=1 SV=3
evm.model.scaffold151467.77	143.741	60.5656	1.24691	0.000820679	sp|Q9ET80|JPH1_MOUSE Junctophilin-1 OS=Mus musculus GN=Jph1 PE=2 SV=1
evm.model.scaffold44291.3	3.30927	1.8167	0.865191	0.0101381	--
evm.model.scaffold148595.5	36.7117	10.9192	1.74937	0.000820679	sp|F1RA39|MCA2B_DANRE Protein-methionine sulfoxide oxidase mical2b OS=Danio rerio GN=mical2b PE=2 SV=2
evm.model.scaffold78215.23	11.6231	3.91478	1.56999	0.000820679	sp|Q06317|PBP4_AMYLA Penicillin-binding protein 4 OS=Amycolatopsis lactamdurans GN=pbp PE=3 SV=1
evm.model.scaffold60115.6	0.604704	0.136403	2.14835	0.0482225	sp|Q14831|GRM7_HUMAN

					Metabotropic glutamate receptor 7 OS=Homo sapiens GN=GRM7 PE=1 SV=1
evm.model.scaffold13107.2	122.11	54.564	1.16216	0.000820679	sp|Q66HD0|ENPL_RAT Endoplasmin OS=Rattus norvegicus GN=Hsp90b1 PE=1 SV=2
evm.model.scaffold51225.68	186.727	27.7965	2.74796	0.000820679	sp|Q8N0N9|ODH_HALDH Opine dehydrogenase OS=Haliotis discus hannai GN=tadh PE=2 SV=1
evm.model.scaffold51225.69	88.9421	23.8265	1.9003	0.000820679	sp|Q8N0N9|ODH_HALDH Opine dehydrogenase OS=Haliotis discus hannai GN=tadh PE=2 SV=1
evm.model.scaffold93729.83	25.1255	12.4567	1.01224	0.000820679	sp|Q502M6|ANR29_DANRE Ankyrin repeat domain-containing protein 29 OS=Danio rerio GN=ankrd29 PE=2 SV=1
evm.model.scaffold65971.20	10.6279	5.81763	0.869351	0.00972356	sp|Q5BKC6|HBAP1_RAT HSPB1-associated protein 1 OS=Rattus norvegicus GN=Hspbap1 PE=1 SV=1
evm.model.scaffold95641.21	240.107	118.505	1.01872	0.000820679	sp|Q71U00|SKP1_XENLA S-phase kinase-associated protein 1 OS=Xenopus laevis GN=skp1 PE=1 SV=3
evm.model.scaffold95641.22	4.24166	0.916801	2.20995	0.000820679	sp|Q96BM1|ANKR9_HUMAN Ankyrin repeat domain-containing

					protein 9 OS=Homo sapiens GN=ANKRD9 PE=2 SV=1
evm.model.scaffold155579.8	124.639	63.4519	0.974026	0.000820679	sp|Q9YI37|SUCB1_COLLI Succinyl-CoA ligase [ADP-forming] subunit beta, mitochondrial (Fragment) OS=Columba livia GN=SUCLA2 PE=1 SV=1
evm.model.scaffold144071.1	60.187	37.6603	0.676408	0.0125549	sp|Q3UMR5|MCU_MOUSE Calcium uniporter protein, mitochondrial OS=Mus musculus GN=Mcu PE=1 SV=2
evm.model.scaffold105515.3	5.15496	2.70691	0.929315	0.0231466	sp|Q8K1C7|MOT14_MOUSE Monocarboxylate transporter 14 OS=Mus musculus GN=Slc16a14 PE=2 SV=1
evm.model.scaffold14507.4	7.70119	3.97299	0.954858	0.0388548	--
evm.model.scaffold90899.65	5.73148	2.90468	0.980529	0.00800162	sp|Q9C0G0|ZN407_HUMAN Zinc finger protein 407 OS=Homo sapiens GN=ZNF407 PE=1 SV=2
evm.model.scaffold170995.68.1	2.5321	0.609454	2.05475	0.00151774	sp|Q9H5P4|PDZD7_HUMAN PDZ domain-containing protein 7 OS=Homo sapiens GN=PDZD7 PE=1 SV=1
evm.model.scaffold175929.9	15.7079	2.56372	2.61518	0.000820679	sp|P26221|GUN4_THEFU Endoglucanase E-4 OS=Thermobifida

					fusca GN=celD PE=1 SV=2
evm.model.scaffold33673.13	61.4572	30.0297	1.03319	0.00151774	sp|P70122|SBDS_MOUSE Ribosome maturation protein SBDS OS=Mus musculus GN=Sbds PE=1 SV=4
evm.model.scaffold156687.4	4.62654	0.926526	2.32003	0.000820679	sp|Q6IWZ0|OXLA_APLCA L-amino-acid oxidase OS=Aplysia californica PE=1 SV=1
evm.model.scaffold33673.17	1.46042	0.643532	1.1823	0.0491986	sp|Q96DY2|IQCD_HUMAN IQ domain-containing protein D OS=Homo sapiens GN=IQCD PE=2 SV=2
evm.model.scaffold129863.3	135.267	42.7222	1.66275	0.000820679	sp|Q6UPE0|CHDH_RAT Choline dehydrogenase, mitochondrial OS=Rattus norvegicus GN=Chdh PE=1 SV=1
evm.model.scaffold96453.5	16.1344	7.5911	1.08776	0.000820679	sp|O49923|ADK_PHYPA Adenosine kinase OS=Physcomitrella patens subsp. patens GN=ADK PE=2 SV=1
evm.model.scaffold96453.1	36.2543	19.8452	0.869363	0.00429787	sp|Q5ZI34|F213A_CHICK Redox-regulatory protein FAM213A OS=Gallus gallus GN=FAM213A PE=2 SV=2
evm.model.scaffold175495.18.1	32.8862	14.2432	1.2072	0.000820679	sp|Q9H4G4|GAPR1_HUMAN Golgi-associated plant pathogenesis-related protein 1

					OS=Homo sapiens GN=GLIPR2 PE=1 SV=3
evm.model.scaffold160425.1	32.7418	8.79821	1.89585	0.000820679	sp|P82125|AKCL2_PIG 1,5-anhydro-D-fructose reductase OS=Sus scrofa GN=AKR1E2 PE=1 SV=2
evm.model.scaffold48809.8	100.346	35.4447	1.50134	0.000820679	sp|P11167|GTR1_RAT Solute carrier family 2, facilitated glucose transporter member 1 OS=Rattus norvegicus GN=Slc2a1 PE=1 SV=1
evm.model.scaffold133259.4_evm.model.scaffold133259.5	56.9485	23.2617	1.2917	0.000820679	sp|Q674X7|KAZRN_HUMAN Kazrin OS=Homo sapiens GN=KAZN PE=1 SV=2
evm.model.scaffold41199.1	7.07194	2.33598	1.59808	0.000820679	sp|D3ZAT9|FAXC_RAT Failed axon connections homolog OS=Rattus norvegicus GN=Faxc PE=3 SV=1
evm.model.scaffold60761.16	157.281	35.8663	2.13265	0.000820679	sp|Q7ZVP8|TM38B_DANRE Trimeric intracellular cation channel type B OS=Danio rerio GN=tmem38b PE=2 SV=1
evm.model.scaffold8823.4	12.6764	0.635393	4.31835	0.000820679	sp|Q8CEE6|PASK_MOUSE PAS domain-containing serine/threonine-protein kinase OS=Mus musculus GN=Pask PE=2 SV=3
evm.model.scaffold106529.6	12.7007	6.4505	0.977429	0.0264598	--

evm.model.scaffold33513.6_evm.model.scaffold33513.7	23.1574	12.6154	0.876283	0.00151774	sp|P97879|GRIP1_RAT Glutamate receptor-interacting protein 1 OS=Rattus norvegicus GN=Grip1 PE=1 SV=1
evm.model.scaffold128037.8	12.6856	4.73681	1.4212	0.000820679	sp|Q8K1C7|MOT14_MOUSE Monocarboxylate transporter 14 OS=Mus musculus GN=Slc16a14 PE=2 SV=1
evm.model.scaffold154027.31	50.5761	28.2437	0.840531	0.000820679	sp|Q99MK9|RASF1_MOUSE Ras association domain-containing protein 1 OS=Mus musculus GN=Rassf1 PE=2 SV=1
evm.model.scaffold162875.2	39.1042	23.8156	0.715413	0.0208422	sp|Q01433|AMPD2_HUMAN AMP deaminase 2 OS=Homo sapiens GN=AMPD2 PE=1 SV=2
evm.model.scaffold162875.3	35.9165	19.0854	0.912178	0.00326205	sp|Q02356|AMPD2_RAT AMP deaminase 2 OS=Rattus norvegicus GN=Ampd2 PE=2 SV=2
evm.model.scaffold26269.3	175.445	81.4019	1.10789	0.000820679	sp|P34416|LASP1_CAEEL LIM and SH3 domain protein F42H10.3 OS=Caenorhabditis elegans GN=F42H10.3 PE=3 SV=3
evm.model.scaffold2735.7	39.2194	22.108	0.826998	0.00578348	--
evm.model.scaffold25635.1	301.852	66.8559	2.17471	0.000820679	sp|Q80X80|C2C2L_MOUSE C2 domain-containing protein 2-like

					OS=Mus musculus GN=C2cd2l PE=1 SV=3
evm.model.scaffold54713.44	19.1178	7.66903	1.3178	0.000820679	sp|Q9NX36|DJC28_HUMAN DnaJ homolog subfamily C member 28 OS=Homo sapiens GN=DNAJC28 PE=1 SV=2
evm.model.scaffold119493.2	139.047	70.8477	0.972777	0.000820679	--
evm.model.scaffold146623.57	10.0392	3.60903	1.47597	0.000820679	sp|Q1IY56|KYNB_DEIGD Kynurenine formamidase OS=Deinococcus geothermalis (strain DSM 11300) GN=kynB PE=3 SV=1
evm.model.scaffold88177.30	1195.85	219.444	2.44611	0.000820679	sp|B3QVL0|GPMA_CHLT3 2,3-bisphosphoglycerate-dependent phosphoglycerate mutase OS=Chloroherpeton thalassium (strain ATCC 35110 / GB-78) GN=gpmA PE=3 SV=1
evm.model.scaffold88177.34	140.673	25.7049	2.45223	0.000820679	sp|Q8C8H8|KY_MOUSE Kyphoscoliosis peptidase OS=Mus musculus GN=Ky PE=1 SV=1
evm.model.scaffold75453.2	1.60658	0.818767	0.972468	0.0420901	sp|Q6B9X6|VWKA_DICDI Alpha-protein kinase vwkA OS=Dictyostelium discoideum GN=vwkA PE=1 SV=1
evm.model.scaffold70561.44.1	4.94119	2.7809	0.829308	0.0314536	sp|Q9NR55|BATF3_HUMAN Basic

					leucine zipper transcriptional factor ATF-like 3 OS=Homo sapiens GN=BATF3 PE=1 SV=1
evm.model.scaffold168171.8	1.89289	0.720201	1.39412	0.00800162	sp|Q8R0M8|MOT5_MOUSE Monocarboxylate transporter 5 OS=Mus musculus GN=Slc16a4 PE=2 SV=1
evm.model.scaffold19291.11	6304.92	1281.61	2.29852	0.000820679	sp|P24733|MYS_ARGIR Myosin heavy chain, striated muscle OS=Argopecten irradians PE=1 SV=1
evm.model.scaffold19291.12	2167.33	247.149	3.13246	0.000820679	sp|P24733|MYS_ARGIR Myosin heavy chain, striated muscle OS=Argopecten irradians PE=1 SV=1
evm.model.scaffold132315.33	6.46124	3.44211	0.908517	0.0296885	sp|Q969T7|5NT3B_HUMAN 7-methylguanosine phosphate-specific 5'-nucleotidase OS=Homo sapiens GN=NT5C3B PE=1 SV=4
evm.model.scaffold167909.3_evm.model.scaffold167909.4	24.6994	14.6749	0.751124	0.00578348	sp|Q61214|DYR1A_MOUSE Dual specificity tyrosine-phosphorylation-regulated kinase 1A OS=Mus musculus GN=Dyrk1a PE=1 SV=1
evm.model.scaffold61349.3	126.46	40.2335	1.65222	0.000820679	sp|P18172|DHGL_DROPS Glucose dehydrogenase [FAD, quinone] OS=Drosophila pseudoobscura

					pseudoobscura GN=Gld PE=3 SV=4
evm.model.scaffold61349.2	1.66437	0.492686	1.75623	0.013343	sp|P18172|DHGL_DROPS Glucose dehydrogenase [FAD, quinone] OS=Drosophila pseudoobscura pseudoobscura GN=Gld PE=3 SV=4
evm.model.scaffold138193.5	7.7976	3.75813	1.05302	0.0177206	sp|A5WUX7|CL065_DANRE Probable peptide chain release factor C12orf65 homolog, mitochondrial OS=Danio rerio GN=si:ch211-275j6.5 PE=3 SV=1
evm.model.scaffold65307.31	6.82865	4.38268	0.639786	0.0447111	sp|Q9U3V5|TIPT_DROME Protein tiptop OS=Drosophila melanogaster GN=tio PE=2 SV=2
evm.model.scaffold105705.4	6.21848	3.58388	0.79504	0.0327195	sp|Q3TI53|SCHI1_MOUSE Schwannomin-interacting protein 1 OS=Mus musculus GN=Schip1 PE=1 SV=2
evm.model.scaffold176621.18	0.489097	0.181636	1.42907	0.0332993	sp|Q8BQB6|SAXO2_MOUSE Stabilizer of axonemal microtubules 2 OS=Mus musculus GN=Saxo2 PE=2 SV=2
evm.model.scaffold96499.18	30.6713	19.9426	0.62104	0.029403	sp|O88327|CTNL1_MOUSE Alpha-catulin OS=Mus musculus GN=Ctnnal1 PE=2 SV=1
evm.model.scaffold136165.68	1.74354	0.403736	2.11053	0.0261282	--

evm.model.scaffold823.22	76.5083	47.4803	0.688286	0.0173453	sp|Q9MYP6|DHB14_BOVIN 17-beta-hydroxysteroid dehydrogenase 14 OS=Bos taurus GN=HSD17B14 PE=2 SV=1
evm.model.scaffold176495.82	22.534	14.7895	0.607532	0.0359113	sp|Q5R9B8|DCAF6_PONAB DDB1- and CUL4-associated factor 6 OS=Pongo abelii GN=DCAF6 PE=2 SV=2
evm.model.scaffold144121.9	4.25001	0.349741	3.60311	0.00213042	sp|P60707|ACTB_TRIVU Actin, cytoplasmic 1 OS=Trichosurus vulpecula GN=ACTB PE=2 SV=1
evm.model.scaffold161513.57	9.76166	5.41445	0.850312	0.0148208	sp|Q9JLJ0|LITAF_MOUSE Lipopolysaccharide-induced tumor necrosis factor-alpha factor homolog OS=Mus musculus GN=Litaf PE=1 SV=1
evm.model.scaffold23155.1	3.0803	0.847481	1.86182	0.00380231	sp|P41044|CAB32_DROME Calbindin-32 OS=Drosophila melanogaster GN=Cbp53E PE=2 SV=1
evm.model.scaffold66661.14	3.52439	1.51402	1.21899	0.0218619	sp|Q9VZW5|FMAR_DROME FMRFamide receptor OS=Drosophila melanogaster GN=FR PE=2 SV=1
evm.model.scaffold174343.53	20.7206	11.4766	0.852377	0.00271512	sp|Q9UQB3|CTND2_HUMAN Catenin delta-2 OS=Homo sapiens GN=CTNND2 PE=1 SV=3

evm.model.scaffold54691.27	8.22119	3.83782	1.09906	0.000820679	sp|Q9NS40|KCNH7_HUMAN Potassium voltage-gated channel subfamily H member 7 OS=Homo sapiens GN=KCNH7 PE=2 SV=2
evm.model.scaffold138465.2	129.429	53.2527	1.28124	0.000820679	sp|Q32LP4|S4A10_BOVIN Sodium-driven chloride bicarbonate exchanger OS=Bos taurus GN=SLC4A10 PE=2 SV=1
evm.model.scaffold138465.1	104.761	42.0835	1.31577	0.000820679	sp|Q32LP4|S4A10_BOVIN Sodium-driven chloride bicarbonate exchanger OS=Bos taurus GN=SLC4A10 PE=2 SV=1
evm.model.scaffold110941.13	131.926	45.4197	1.53834	0.000820679	sp|P16257|TSPO_RAT Translocator protein OS=Rattus norvegicus GN=Tspo PE=1 SV=1
evm.model.scaffold173487.5	5267.35	648.312	3.02232	0.000820679	--
evm.model.scaffold173487.4	6803.25	3694.85	0.880708	0.0163478	--
evm.model.scaffold90803.16	58.5761	28.5947	1.03456	0.000820679	--
evm.model.scaffold106529.19_evm.model.scaffold106529.20	103.53	55.3882	0.902393	0.00213042	sp|P36876|2ABA_RAT Serine/threonine-protein phosphatase 2A 55 kDa regulatory subunit B alpha isoform OS=Rattus norvegicus GN=Ppp2r2a PE=2 SV=1
evm.model.scaffold52831.9	131.17	35.5725	1.88261	0.000820679	sp|P53814|SMTN_HUMAN Smoothelin OS=Homo sapiens

					GN=SMTN PE=1 SV=7
evm.model.scaffold12535.11	56.7304	33.1676	0.774346	0.00621905	sp|Q641Y2|NDUS2_RAT NADH dehydrogenase [ubiquinone] iron-sulfur protein 2, mitochondrial OS=Rattus norvegicus GN=Ndufs2 PE=1 SV=1
evm.model.scaffold76053.25.1	11.2996	7.14185	0.6619	0.0287519	sp|P30886|BMP7_XENLA Bone morphogenetic protein 7 OS=Xenopus laevis GN=bmp7 PE=1 SV=1
evm.model.scaffold91437.10.1	16.9559	7.98436	1.08654	0.000820679	sp|P50464|UNC97_CAEEL LIM domain-containing protein unc-97 OS=Caenorhabditis elegans GN=unc-97 PE=1 SV=1
evm.model.scaffold173227.8.22	119.994	49.9759	1.26366	0.000820679	sp|O60504|VINEX_HUMAN Vinexin OS=Homo sapiens GN=SORBS3 PE=1 SV=2
evm.model.scaffold162017.7	145.016	85.653	0.759632	0.00621905	--
evm.model.scaffold142905.6	14.6233	7.60556	0.943141	0.00380231	sp|Q9H9E1|ANRA2_HUMAN Ankyrin repeat family A protein 2 OS=Homo sapiens GN=ANKRA2 PE=1 SV=1
evm.model.scaffold91387.12	37.6214	15.3716	1.29129	0.000820679	sp|Q8BXK9|CLIC5_MOUSE Chloride intracellular channel protein 5 OS=Mus musculus GN=Clic5 PE=1 SV=1
evm.model.scaffold91387.18	1694.18	671.73	1.33464	0.000820679	sp|P41824|YBOXH_APLCA Y-box

					factor homolog OS=Aplysia californica PE=2 SV=1
evm.model.scaffold161075.14	106.327	62.8726	0.758004	0.0105331	--
evm.model.scaffold27973.166.1	3.47204	1.52626	1.18578	0.014476	sp|Q5RD07|KCNK1_PONAB Potassium channel subfamily K member 1 OS=Pongo abelii GN=KCNK1 PE=2 SV=1
evm.model.scaffold169873.12	8921.44	2212.52	2.01159	0.00380231	sp|Q9XZ71|TNNT_PERAM Troponin T OS=Periplaneta americana GN=TNT PE=2 SV=1
evm.model.scaffold98043.9.1	3.19024	1.33412	1.25778	0.00151774	sp|Q8N2E2|VWDE_HUMAN von Willebrand factor D and EGF domain-containing protein OS=Homo sapiens GN=VWDE PE=2 SV=4
evm.model.scaffold9075.16	84.2148	50.1465	0.747924	0.00429787	sp|O00303|EIF3F_HUMAN Eukaryotic translation initiation factor 3 subunit F OS=Homo sapiens GN=EIF3F PE=1 SV=1
evm.model.scaffold40311.81	13.1128	6.63847	0.982056	0.00326205	sp|Q0VCJ7|RERG_BOVIN Ras-related and estrogen-regulated growth inhibitor OS=Bos taurus GN=RERG PE=2 SV=1
evm.model.scaffold153583.1	3.74099	0.501341	2.89956	0.0284609	--
evm.model.scaffold176409.1	49.248	21.621	1.18763	0.000820679	sp|P15388|KCNC1_MOUSE Potassium voltage-gated channel

					subfamily C member 1 OS=Mus musculus GN=Kcnc1 PE=2 SV=1
evm.model.scaffold126605.1	17.6305	7.50498	1.23215	0.000820679	sp|Q8BTK5|SMYD4_MOUSE SET and MYND domain-containing protein 4 OS=Mus musculus GN=Smyd4 PE=2 SV=2
evm.model.scaffold133775.48	5036.39	2160.74	1.22087	0.000820679	sp|Q7M3Y3|TNNI_CHLNI Troponin I OS=Chlamys nipponensis akazara PE=1 SV=2
evm.model.scaffold53705.13	18.8683	10.7589	0.810438	0.00712329	sp|O77793|PA24A_HORSE Cytosolic phospholipase A2 OS=Equus caballus GN=PLA2G4A PE=2 SV=1
evm.model.scaffold64163.1_evm.model.scaffold64163.2	9.25245	5.82747	0.666964	0.0251677	sp|Q9TTK0|CDKL2_RABIT Cyclin-dependent kinase-like 2 OS=Oryctolagus cuniculus GN=CDKL2 PE=2 SV=1
evm.model.scaffold127921.28	51.7515	19.774	1.38799	0.000820679	--
evm.model.scaffold176007.21	8.20311	4.9393	0.731862	0.0199213	sp|O70511|ANK3_RAT Ankyrin-3 OS=Rattus norvegicus GN=Ank3 PE=1 SV=3
evm.model.scaffold45047.18	2.38492	0.856525	1.47738	0.0199213	sp|Q8JIF5|ERAL1_CHICK GTPase Era, mitochondrial OS=Gallus gallus GN=ERAL1 PE=1 SV=1
evm.model.scaffold64407.38	24.8319	15.3965	0.689591	0.0163478	sp|Q5R7N3|CLPX_PONAB ATP-dependent Clp protease

					ATP-binding subunit clpX-like, mitochondrial OS=Pongo abelii GN=CLPX PE=2 SV=1
evm.model.scaffold169873.3	23.6092	7.47431	1.65933	0.000820679	sp|P0CC03|ST6B1_MOUSE Sulfotransferase 6B1 OS=Mus musculus GN=Sult6b1 PE=1 SV=1
evm.model.scaffold56755.17	85.1355	40.6916	1.06503	0.000820679	sp|P22006|SVS2_RAT Seminal vesicle secretory protein 2 OS=Rattus norvegicus GN=Svs2 PE=1 SV=1
evm.model.scaffold154705.73	9.79668	5.05037	0.955903	0.00429787	sp|O81884|GALDH_ARATH L-galactose dehydrogenase OS=Arabidopsis thaliana GN=LGALDH PE=1 SV=1
evm.model.scaffold51225.21	79.1246	36.759	1.10603	0.000820679	sp|Q96D70|R3HD4_HUMAN R3H domain-containing protein 4 OS=Homo sapiens GN=R3HDM4 PE=1 SV=3
evm.model.scaffold117139.8	202.329	115.853	0.804399	0.00380231	sp|P98160|PGBM_HUMAN Basement membrane-specific heparan sulfate proteoglycan core protein OS=Homo sapiens GN=HSPG2 PE=1 SV=4
evm.model.scaffold77239.25	87.7102	47.0608	0.89822	0.00213042	sp|G5E8K5|ANK3_MOUSE Ankyrin-3 OS=Mus musculus GN=Ank3 PE=1 SV=1
evm.model.scaffold77239.24	60.1644	34.5336	0.800911	0.00271512	--

evm.model.scaffold168151.11	6.3898	1.73151	1.88374	0.00271512	--
evm.model.scaffold136555.19	193.058	43.6856	2.14381	0.000820679	sp|P16641|KTRC_SCHMA Taurocyamine kinase OS=Schistosoma mansoni GN=Smp_194770 PE=1 SV=3
evm.model.scaffold122901.20	18.2591	10.1512	0.846964	0.00326205	sp|Q149C3|LIGO4_MOUSE Leucine-rich repeat and immunoglobulin-like domain containing-NOGO receptor-interacting protein 4 OS=Mus musculus GN=Lingo4 PE=2 SV=2
evm.model.scaffold45687.7	9.65868	5.10852	0.918921	0.0228478	sp|P81908|CHLE_HORSE Cholinesterase OS=Equus caballus GN=BCHE PE=1 SV=1
evm.model.scaffold52321.54	66.8505	42.2047	0.663536	0.0222103	sp|Q0MQI5|NDUV1_GORGO NADH dehydrogenase [ubiquinone] flavoprotein 1, mitochondrial OS=Gorilla gorilla gorilla GN=NDUFV1 PE=2 SV=1
evm.model.scaffold142393.6	1.14372	0.25248	2.1795	0.0427039	--
evm.model.scaffold141215.7	12.846	8.04791	0.674631	0.0413639	sp|Q91453|STXB_SYNHO Stonustoxin subunit beta OS=Synanceia horrida PE=1 SV=3
evm.model.scaffold50517.9	23.7669	15.6555	0.602286	0.0486765	sp|Q00492|SUM1_LYTVA Transcription factor SUM-1 OS=Lytechinus variegatus GN=SUM-1

					PE=2 SV=1
evm.model.scaffold175037.6	452.511	235.352	0.943132	0.0105331	sp|Q9I7U4|TITIN_DROME Titin OS=Drosophila melanogaster GN=sls PE=1 SV=3
evm.model.scaffold175037.3	1231.06	600.739	1.03509	0.0113727	sp|Q28824|MYLK_BOVIN Myosin light chain kinase, smooth muscle OS=Bos taurus GN=MYLK PE=1 SV=1
evm.model.scaffold81473.59	4.39064	2.20905	0.991006	0.000820679	sp|Q12912|LRMP_HUMAN Lymphoid-restricted membrane protein OS=Homo sapiens GN=LRMP PE=1 SV=3
evm.model.scaffold81473.55	20.9516	9.38257	1.159	0.000820679	sp|P9WKC5|TGS3_MYCTU Probable diacyglycerol O-acyltransferase tgs3 OS=Mycobacterium tuberculosis (strain ATCC 25618 / H37Rv) GN=tgs3 PE=1 SV=1
evm.model.scaffold3225.10	23.5504	10.9703	1.10216	0.000820679	sp|Q22328|LEV9_CAEEL Protein lev-9 OS=Caenorhabditis elegans GN=lev-9 PE=1 SV=3
evm.model.scaffold74869.9	65.6854	30.1072	1.12546	0.000820679	sp|Q9Y663|HS3SA_HUMAN Heparan sulfate glucosamine 3-O-sulfotransferase 3A1 OS=Homo sapiens GN=HS3ST3A1 PE=1 SV=1
evm.model.scaffold158547.27	398.407	222.319	0.841615	0.0121758	sp|P29341|PABP1_MOUSE Polyadenylate-binding protein 1

					OS=Mus musculus GN=Pabpc1 PE=1 SV=2
evm.model.scaffold54817.4	55.4542	32.8189	0.756768	0.00429787	sp|P49432|ODPB_RAT Pyruvate dehydrogenase E1 component subunit beta, mitochondrial OS=Rattus norvegicus GN=Pdhb PE=1 SV=2
evm.model.scaffold26913.6	170.66	95.4679	0.838034	0.00151774	sp|Q8BU85|MSRB3_MOUSE Methionine-R-sulfoxide reductase B3, mitochondrial OS=Mus musculus GN=Msrb3 PE=1 SV=2
evm.model.scaffold164003.1	0.51563	0	inf	0.0218619	--
evm.model.scaffold33117.12.1	64.2575	41.7621	0.621671	0.0345537	sp|Q9UGJ0|AAKG2_HUMAN 5'-AMP-activated protein kinase subunit gamma-2 OS=Homo sapiens GN=PRKAG2 PE=1 SV=1
evm.model.scaffold112763.13_evm.model.scaffold112763.14	1211.55	80.0244	3.92027	0.000820679	--
evm.model.scaffold88753.100	5.86436	3.47342	0.755615	0.0373071	sp|P22488|IFEA_HELAS Non-neuronal cytoplasmic intermediate filament protein OS=Helix aspersa PE=3 SV=2
evm.model.scaffold163411.13	2.01533	0.457911	2.13788	0.0287519	sp|Q15399|TLR1_HUMAN Toll-like receptor 1 OS=Homo sapiens GN=TLR1 PE=1 SV=3
evm.model.scaffold163411.12	5.81778	2.06719	1.4928	0.023485	--

evm.model.scaffold103525.15	41.6342	25.3137	0.717847	0.00842602	sp|Q8TBG4|AT2L1_HUMAN Ethanolamine-phosphate phospho-lyase OS=Homo sapiens GN=ETNPPL PE=1 SV=1
evm.model.scaffold171579.8	156.008	89.3282	0.804434	0.00213042	sp|P17081|RHOQ_HUMAN Rho-related GTP-binding protein RhoQ OS=Homo sapiens GN=RHOQ PE=1 SV=2
evm.model.scaffold173383.28	43.647	24.0504	0.859821	0.000820679	sp|Q8IVE3|PKHH2_HUMAN Pleckstrin homology domain-containing family H member 2 OS=Homo sapiens GN=PLEKHH2 PE=1 SV=2
evm.model.scaffold176479.5	199.6	50.812	1.97387	0.000820679	sp|Q99JR5|TINAL_MOUSE Tubulointerstitial nephritis antigen-like OS=Mus musculus GN=Tinagl1 PE=1 SV=1
evm.model.scaffold4289.52	4.58013	2.00554	1.1914	0.0113727	sp|Q96T60|PNKP_HUMAN Bifunctional polynucleotide phosphatase/kinase OS=Homo sapiens GN=PNKP PE=1 SV=1
evm.model.scaffold147433.16	8.27084	4.06934	1.02324	0.0373071	sp|P20072|ANXA7_BOVIN Annexin A7 OS=Bos taurus GN=ANXA7 PE=1 SV=2
evm.model.scaffold2221.15	4.33757	2.15748	1.00754	0.0404844	sp|Q9W6I0|UQCC1_XENLA

					Ubiquinol-cytochrome-c reductase complex assembly factor 1 OS=Xenopus laevis GN=uqcc1 PE=2 SV=1
evm.model.scaffold143487.32_evm.model.scaffold143487.31	4.10761	1.7655	1.21822	0.00151774	sp|Q4R744|HEAT9_MACFA Protein HEATR9 OS=Macaca fascicularis GN=HEATR9 PE=2 SV=1
evm.model.scaffold104247.2	19.9566	12.4097	0.685395	0.014476	sp|Q6P9K8|CSKI1_MOUSE Caskin-1 OS=Mus musculus GN=Caskin1 PE=1 SV=2
evm.model.scaffold146665.16	76.4317	7.91274	3.27192	0.000820679	sp|P86789|GIGA6_CRAGI Gigasin-6 OS=Crassostrea gigas PE=1 SV=1
evm.model.scaffold176559.7	60.6134	29.5438	1.03678	0.000820679	sp|A1XQX0|NR1AA_DANRE Neurexin-1a OS=Danio rerio GN=nrxn1a PE=2 SV=1
evm.model.scaffold112475.5	126.713	75.4702	0.747581	0.0113727	sp|O01479|TMOD_CAEEL Tropomodulin OS=Caenorhabditis elegans GN=unc-94 PE=1 SV=2
evm.model.scaffold112475.6	10.7652	5.9203	0.862635	0.00213042	sp|Q9C0G6|DYH6_HUMAN Dynein heavy chain 6, axonemal OS=Homo sapiens GN=DNAH6 PE=2 SV=3
evm.model.scaffold163753.34	51.6556	32.279	0.678329	0.0148208	sp|P91929|NDUAA_DROME NADH dehydrogenase [ubiquinone] 1 alpha subcomplex subunit 10, mitochondrial OS=Drosophila melanogaster

					GN=ND42 PE=2 SV=2
evm.model.scaffold82287.19	12.3015	6.01634	1.03188	0.00271512	sp|Q9P255|ZN492_HUMAN Zinc finger protein 492 OS=Homo sapiens GN=ZNF492 PE=2 SV=2
evm.model.scaffold31507.6	78.4864	30.3573	1.3704	0.000820679	sp|P52564|MP2K6_HUMAN Dual specificity mitogen-activated protein kinase kinase 6 OS=Homo sapiens GN=MAP2K6 PE=1 SV=1
evm.model.scaffold62683.4	20.9037	9.28502	1.17078	0.000820679	sp|Q6P963|GLO2_DANRE Hydroxyacylglutathione hydrolase, mitochondrial OS=Danio rerio GN=hagh PE=2 SV=2
evm.model.scaffold104967.2.1	38.711	24.3976	0.666005	0.0208422	sp|Q9WVM7|AIMP2_CRIGR Aminoacyl tRNA synthase complex-interacting multifunctional protein 2 OS=Cricetulus griseus GN=AIMP2 PE=2 SV=1
evm.model.scaffold114775.4	1.02459	0.44392	1.20667	0.0494585	sp|Q9YHB5|CND3_XENLA Condensin complex subunit 3 OS=Xenopus laevis GN=ncapg PE=1 SV=1
evm.model.scaffold60387.16	53.9845	32.5246	0.731013	0.00578348	sp|Q8N3T6|T132C_HUMAN Transmembrane protein 132C OS=Homo sapiens GN=TMEM132C PE=2 SV=3

evm.model.scaffold64407.36	20.935	11.895	0.815568	0.00578348	sp|Q6PB70|ANO8_MOUSE Anoctamin-8 OS=Mus musculus GN=Ano8 PE=2 SV=3
evm.model.scaffold64407.34	66.2846	39.1691	0.758958	0.0159979	sp|O94788|AL1A2_HUMAN Retinal dehydrogenase 2 OS=Homo sapiens GN=ALDH1A2 PE=1 SV=3
evm.model.scaffold109989.15	73.9919	43.2781	0.77373	0.00326205	sp|P62958|HINT1_BOVIN Histidine triad nucleotide-binding protein 1 OS=Bos taurus GN=HINT1 PE=1 SV=2
evm.model.scaffold74253.3	13.8917	7.53247	0.883031	0.00755259	sp|Q969R8|ITFG2_HUMAN Integrin-alpha FG-GAP repeat-containing protein 2 OS=Homo sapiens GN=ITFG2 PE=1 SV=1
evm.model.scaffold14199.7	8.59222	5.14502	0.739853	0.039382	--
evm.model.scaffold14199.4	32.2089	16.3307	0.979869	0.00151774	sp|Q5U4T7|BIC1B_XENLA Protein bicaudal C homolog 1-B OS=Xenopus laevis GN=bicc1-b PE=2 SV=1
evm.model.scaffold14199.5	29.265	11.9424	1.29308	0.000820679	sp|Q5U4T7|BIC1B_XENLA Protein bicaudal C homolog 1-B OS=Xenopus laevis GN=bicc1-b PE=2 SV=1
evm.model.scaffold14199.2	21.161	12.2239	0.791707	0.0141107	sp|Q503W7|ATD1B_DANRE ATPase family AAA domain-containing protein 1-B OS=Danio rerio GN=atad1b PE=2 SV=2
evm.model.scaffold64407.22	248.561	156.144	0.67072	0.0342138	sp|A2AX52|CO6A4_MOUSE

					Collagen alpha-4(VI) chain OS=Mus musculus GN=Col6a4 PE=1 SV=2
evm.model.scaffold7987.11	198.278	36.022	2.46057	0.000820679	--
evm.model.scaffold18403.3	5.40455	2.45233	1.14002	0.0276479	sp|P11833|TBB_PARLI Tubulin beta chain OS=Paracentrotus lividus PE=2 SV=1
evm.model.scaffold137721.7	1.68922	0.807138	1.06547	0.0222103	sp|P87061|TEA1_SCHPO Tip elongation aberrant protein 1 OS=Schizosaccharomyces pombe (strain 972 / ATCC 24843) GN=tea1 PE=1 SV=1
evm.model.scaffold156619.17	22.8262	12.2361	0.899543	0.00429787	sp|P0C7B7|ELIC_DICCH Cys-loop ligand-gated ion channel OS=Dickeya chrysanthemi PE=1 SV=1
evm.model.scaffold156619.10	33.2808	21.2469	0.647441	0.0228478	sp|Q32PA4|PHP14_BOVIN 14 kDa phosphohistidine phosphatase OS=Bos taurus GN=PHPT1 PE=2 SV=1
evm.model.scaffold23897.23	708.077	120.306	2.5572	0.000820679	sp|O02654|ENO_DORPE Enolase OS=Doryteuthis pealeii PE=2 SV=1

Table S4 Primers used for qRT-PCR

gene	Forward	Reverse
XLOC_033661	TGCAAGTGAATGTGAAGCTG	TATGGCGGTCAGTATCCTCG
XLOC_014032	GCTGTCCCCTAGTTTCCTGT	CCGCCGTGATATTGCTGAAA
XLOC_019974	CCGTGTGTGTCTTTTGGTGT	TTGCGATGAGGAAACGTTGG
XLOC_044392	GTGTCGTCGTCTTTCTCGTG	GCAGAAGTCTTTGGTTGGCA
XLOC_030357	CGCGTGAAGTCAGTCAGAAG	GGTTAGCGTATCCTGTCCCA
XLOC_047280	TCATTTGCCAACACAGCCAA	CAGGTATCTTCGCCACAACG
XLOC_011639	TACCCAGAGTGACTTCCCTT	TTTGGACCAGGACGCAGC
Gdf8	TGAGTCGGGAGATTCTTCGC	TGATGATGTCGGTTGTCGTG
Mef2A	GTCATGCAGCAGAACGTCAT	ACCGGCATAGTGTTGTGGTA
Smad3	GTTTGCCGAGTGTCTCAGTG	CCCTGGTGGTATCTTGCAGA
β-actin	GGTATCCTCACCCTCAAGT	GGGTCATCTTTTCACGGTTG
